# Kdm2a histone demethylase ablation restores anti-tumor immunogenicity in liver cancer

**DOI:** 10.1038/s41416-026-03566-z

**Published:** 2026-08-19

**Authors:** Laura Gragnani, Matteo Lulli, Patrizio Caini, Pinuccia Faviana, Rossella Del Frate, Enrico Desideri, Katia De Ieso, Francesco Bartoli, Andrew Hall, Tu Vinh Luong, Krista Rombouts, Vinicio Carloni

**Affiliations:** 1 Department of Translational Research and New Medical and Surgical Technologies, University of Pisa, Pisa, Italy; 2 Department of Experimental and Clinical Biomedical Sciences “Mario Serio”, University of Florence, Florence, Italy; 3 Department of Experimental and Clinical Medicine, University of Florence, Florence, Italy; 4 Department of Surgical, Medical, Molecular Pathology and Critical Area, University of Pisa, Pisa, Italy; 5 Department of Life Sciences, Health and Health Professions Link Campus University, Rome, Italy; 6 Azienda Ospedaliero-Universitaria Pisana, Pisa, Italy; 7 The Sheila Sherlock Liver Centre, Royal Free Hospital, London, UK; 8 Department of Cellular Pathology, Royal Free Hospital, London, UK; 9 University College London (UCL), Institute for Liver & Digestive Health, London, UK

**Keywords:** Cancer, Oncogenesis, Gastrointestinal cancer

## Abstract

**Background:**

Liver cancer remains a major global challenge, with limited benefit from immune checkpoint inhibitors due to strong immune evasion. Emerging evidence indicates that chromatin modifiers shape tumor immunogenicity, but their roles in liver cancer immunity are not fully understood.

**Methods:**

We investigated the role of the histone demethylase KDM2A in liver cancer using genetic silencing in vitro and in vivo. We evaluated mitochondrial function, redox balance, genomic stability, and performed transcriptomic and epigenomic analyses to assess immune pathway changes and H3K36me2 distribution. Immune infiltration was analyzed in human liver tumors and syngeneic mouse models.

**Results:**

KDM2A silencing caused mitochondrial dysfunction, redox imbalance, and DNA damage, leading to activation of innate and adaptive immune pathways. It also reshaped H3K36me2 deposition at loci regulating chemokine signaling, metabolism, and T-cell recruitment. In human liver tumors, high KDM2A expression correlated with poor differentiation, macrophage enrichment, and exclusion of CD8⁺ T cells, consistent with an immune-excluded phenotype. In syngeneic models, KDM2A loss increased intratumoral CD8⁺ T-cell infiltration.

**Conclusions:**

KDM2A acts as a key epigenetic regulator of mitochondrial function, genomic stability, and the tumor immune microenvironment in liver cancer. Targeting KDM2A may enhance tumor immunogenicity and improve responses to immunotherapy, representing a promising therapeutic strategy.

**Figure:**
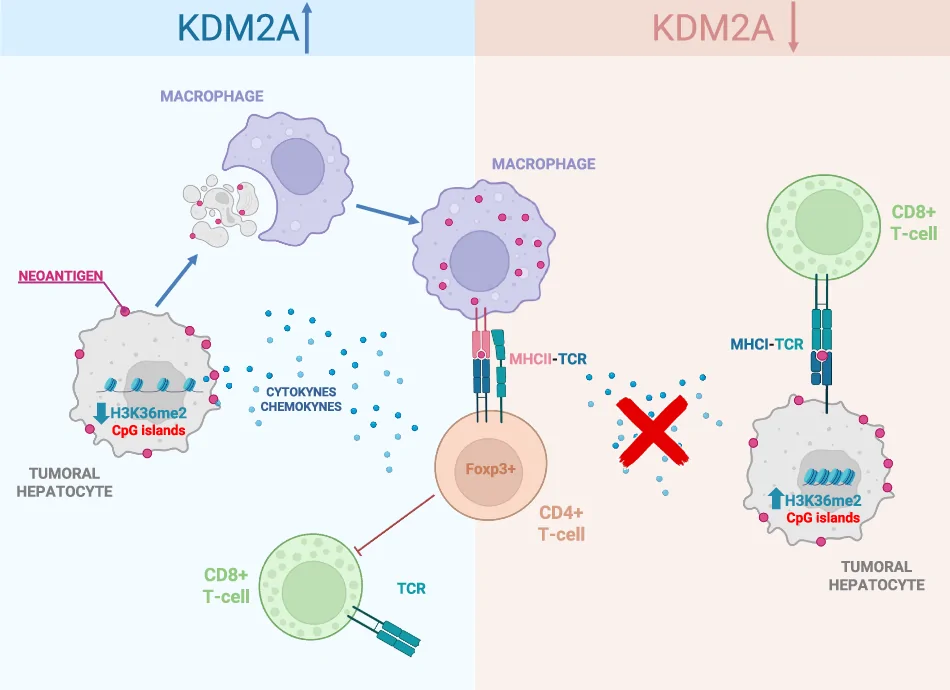

## Introduction

Liver cancer is one of the leading causes of cancer-related death worldwide [1, 2]. Despite advancements in research, the burden of hepatocellular carcinoma (HCC) continues to escalate, necessitating a deeper understanding of its complexities [1, 2]. Chromosomal instability (CIN), i.e. the genomic imbalance in chromosome number and structure, plays a crucial role in the progression of liver cancer by promoting genetic diversity that drives the emergence of tumor clones with increasing malignancy. Recently, it has become evident that DNA damage response (DDR) is closely linked to CIN. Our previous findings showed that checkpoint kinase 2 (CHK2), a central effector of DDR, is linked to CIN in HCC, and contributes to a metabolic switch towards glycolysis for ATP production due to dysfunctional mitochondria [3]. HCC progression is driven not only by CIN but also by epigenetic modifications [4, 5] whose dysregulation is a hallmark of cancer [6]. In fact, epigenetic modifications, such as histone methylation, can alter chromatin structure and gene transcription without changing the underlying genomic sequence [4]. These modifications play a significant role in regulating DNA damage levels and genomic instability to preserve cell survival [5]. In this context, studies conducted in various cancers have suggested a role for histone demethylases in the DDR, further reinforcing the link between epigenetic regulation and genome stability in cancer [7–11]. In addition, recent studies have uncovered a critical connection between DDR and the activation of anti-tumor immunity. For instance, genotoxic stress activates innate immune pathways such as cyclic-GMP-AMP synthase (cGAS)-stimulator of interferon genes (STING) signaling [12]. This triggers the production of type I interferons and chemokines that enhance immune cell recruitment, particularly CD8⁺ cytotoxic T lymphocytes, into the tumor microenvironment [13]. Moreover, unresolved or chronic DNA damage has been shown to upregulate immune checkpoint molecules such as programmed death-ligand 1 (PD-L1), contributing to immune escape [14]. These insights highlight the DDR as a key modulator of tumor immunogenicity as well. On the other side, according to recent research, histone methylation modification can also regulate immune cells affecting anti-tumor immune response [15, 16]. For instance, lysine demethylase 5 A (KDM5A) has been shown to repress interferon-stimulated genes and limit tumor immune surveillance [17], and histone demethylases contribute to the regulation of PD-L1 expression [15, 18], highlighting their potential as targets to sensitize tumors to immune checkpoint blockade therapies. Lysine demethylase 2 A (KDM2A) is a protein with histone H3 lysine 36 demethylase activity [19]. In addition, it contains a ZF-CxxC DNA-binding domain that enables it to interact with unmethylated CpG islands in the genome, where it exerts its enzymatic function. Approximately 70% of mammalian gene promoters are found within CpG islands [20]. KDM2A catalyzes removal of methylation from histone H3 lysine 36 with a preference for H3K36me2, while it has weak or no activity for H3K36me1 and H3K36me3 [21, 22]. KDM2A has been found involved in tumorigenesis, however the reports describing its role in cancer are often at odds and poorly understood [21, 23–25]. Regarding the involvement of KDM2A in HCC limited studies are currently available, showing a correlation between KDM2A levels and poor prognosis [25]. This combined clinical and biological problem prompted us to ask whether KDM2A might select for HCC with a common set of survival advantage mechanisms.

## Materials and methods

### Cell cultures and antibodies

Huh7 and HCT116 were previously characterized by karyotyping [3, 26] and analyzed by RNA-seq. Human hepatocytes immortalized with TERT gene (HuS) were provided by Makoto Hijikata (Laboratory of Human Tumor Viruses, Institute of Virus Research, Kyoto University, Japan) and characterized by karyotyping [3, 26, 27] and RNA-seq. Murine HCC cell line Hepa1-6 cells was acquired by ATCC. HCT116 and Huh7 were grown in DMEM medium supplemented with 10% fetal bovine serum, 1% penicillin/streptomycin, and 1% glutamine at 37 °C and 95% humidity and 5% CO_2_. HuS cells were cultured in DMEM medium supplemented with 5% fetal bovine serum, 5% normal human serum from male type AB serum (Sigma-Aldrich), 1% DMSO, 0.1 µL/mL insulin, 0.1 µL/mL hydrocortisone, and 2 ng/mL EGF. Cells were routinely assayed for mycoplasma contamination. Antibodies employed were anti-KDM2A (Abcam, ab191387), anti-H3K36me2 (Abcam, ab176921), anti-HP1 (Santa Cruz Biotechnology, sc-515341), anti-PD-L1 (Origene, #TA374430), anti-Sting (Origene, #TA382116), anti-IFI16 (Cell Signaling, #14970), anti-cGas (Cell Signaling #15102), anti-MTHFD1 (Abcam, ab103698), anti-SHMT1 (Abcam, ab186130), anti-SHMT2 (Abcam, ab180786), anti-Histone H3 (Cell Signaling, #9715),anti-phospho-Histone H2AX (Ser139)(Cell Signaling, #9718), anti-CHK2 (Cell Signaling #3440), anti-phospho-CHK2 (Thr68) (Abcam, ab278548), anti-β-actin (Cell Signaling, #3700), anti-H-2Db(BioRad, MCA2189), anti-HLA-A/B (Santa Cruz Biotechnology, sc-32235), normal rabbit IgG (Cell Signaling #2729S). Secondary antibodies conjugated to AlexaFluor 488 and 594 (Molecular Probes) were used in immunofluorescence analysis. Secondary antibodies used in immunoblot analysis were HRP anti-rabbit (BioRad, #1706515) and HRP anti-mouse (ThermoFisher Scientific,#G21040). DNA was stained with Hoechst 33342 (ThermoFisher Scientific). Ponceau S stain was used for total protein normalization [28, 29].

### Retroviral infections

The following shRNAs (OriGene Technologies) were stably delivered into cells by retroviral infection. TR315641 is a set of plasmids containing four shRNA constructs in retroviral vector (pRSvector, TR30012) to knockdown human KDM2A. TR506710 is a set of plasmids containing four shRNA constructs in retroviral vector (pRS vector) to knock down mouse KDM2A. TR30015 is a non-effective 29mer scrambled shRNA used as control. Retroviral particles were generated by transient transfection of 293 T cells (ATCC, CRL-3216) with plasmid pUMVC (Addgene #8449) in conjunction with the envelope plasmid pCMV-VSV-G.293 T cells plated onto 100 mm dishes were transfected at approximately 80% confluence with 20 µg of plasmid using Trans-IT-X2 (Mirus) transfection reagent. After 48 h, virus-containing supernatant was recovered and centrifuged, filtered (0.45 µm filters), and used immediately or aliquoted and frozen at −80 °C. Retrovirally infected cells were selected in puromycin-containing medium and, after 2 weeks, reduced expression of proteins were assessed by immunoblotting.

### Tissue Samples and Immunohistochemistry Assays

*Pisa cohort, University Hospital of Pisa;* formalin-fixed, paraffin-embedded tissue were obtained from an archive of surgical specimens from patients diagnosed with HCC. Ethical was given by the Institutional Review Board Comitato Etico Regione Toscana-Area vasta nord ovest-Protocol code: # 9989. All of the H&E-stained sections were reviewed, and areas of HCC were identified. Tissue sections of 42 patients were deparaffinized and hydrated in graded ethanols. The 42 HCC specimens were distributed as follows: G1, *n* = 14; G2, *n* = 14; G3, *n* = 14. Antigen retrieval was performed by heating in citrate buffer pH 6.0 at 98 °C for 30 minutes. Non-specific signal was eliminated by peroxidase block for 10 min at RT. Primary antibody was incubated at room temperature overnight in a humidified chamber, followed by HRP-conjugated secondary antibody incubation for 30 min at room temperature. Antibody binding was revealed by DAB. Appropriate positive and negative controls were included for each run of immunohistochemistry. Tissue sections were used for immunohistochemical analysis and stained with anti-KDM2A antibody (EPR18602 anti-KDM2A, Abcam; 1:50); anti-CD163 antibody (MRQ26 anti-CD163, Cell Marque 1:400); anti-CD80 antibody (EPR-1157 anti-CD80, Abcam 1:400); anti-CD4 antibody (SP35 anti-CD4 antibody, Ventana), prediluted; anti-CD8 antibody (SP57 anti-CD8α chain antibody, Ventana), prediluted. The colours for KDM2A, CD163, CD80, CD4 and CD8A were performed with Benchmark Ultra, Ventana Medical Systems (Tucson, AZ). All IHC staining slides were assessed with a light microscope, and tissue sections were scored by a pathologist and two researchers independently.

*London cohort, Royal Free Hospital;* ethical approval was given for use of materials within guidelines of the Royal Free Hospital, London, UK (07/Q0501/50, IRAS ID 254793). Formalin-fixed, paraffin-embedded surgical specimens from patients diagnosed with cirrhotic-related HCC were retrieved from Royal Free Hospital archives. All samples were stained with H&E, and the sections were reviewed, and suitable areas of cirrhosis and HCC were identified in 20 samples. The primary antibody was incubated at room temperature for 1 h (1:300 KDM2A). Antibody detection was carried out using the ImmPRESS polymer detection system (Vector) and developed using DAB (Abcam). Tissue sections were counterstained with Mayer’s hematoxylin.

### Mice

Six- to eight-wk-old female mice were used for all experiments. C57BL/6 mice were purchased from Charles River Laboratories. Animal experimentation was carried out in accordance with the Guide for the Care and Use of Laboratory Animals. Animal numbers were estimated on the basis of pilot studies of tumor growth. The protocol of animal study were approved by the responsible administration of the Italian Ministry of Health. Mice were anesthetized with isofluorane, and skin was sterilized with 70% ethanol. A 1 cm horizontal incision was made from the midline toward the upper abdomen, and the left lobe of the liver exposed. A total of 5 × 10^5^GFP-labeled Hepa 1-6 cells (scramble and KDM2A knockdown) were suspended in 50 µl of PBS and injected into the left lobe of the liver using a 30 G needle. After the injection, the needle was removed, and gentle pressure was applied using a cotton-tipped applicator. The skin was closed with 5-0 Vicryl sutures. All animals received a standard laboratory diet and were weighed each week. No randomization was used. No blinding was done. At the beginning of the 4th week the animal were euthanized, the livers harvested and tumors were sized with caliper.

### Native chromatin immunoprecipitation and sequencing

GFP-labeled Hepa 1-6shCTL and GFP-labeled Hepa 1-6shKDM2A depleted cells cells were excised on day 14 post injection and plated in 60 mm dishes. The growth of GFP-labeled Hepa 1-6shCTL and shKDM2A cells was evaluated by epifluorescence microscopy. Cells grown were washed with ice-cold PBS twice to completely remove residual medium and scraped from culture dishes and collected in PBS, then centrifuged for 3 min,4°C at 2500 rpm. Cells were re-suspended on ice 15 min in lysis buffer 1 mM CaCl_2_, 0.5% Triton X-100, 20 mM Tris-HCl pH 7.6, 1 mM DTT and protease inhibitors. Cells were centrifuged for 3 min, 4°C at 2500 rpm to collect nuclei. Nuclei were suspended in lysis buffer 15 min and chromatin shearing performed with a syringe needle 30 G, then centrifuged for 3 min, 4°C at 2500 rpm to collect chromatin. Chromatin was suspended in the reaction buffer with restriction enzyme HinP1I at 37 °C for 2 h (New England BioLabs, R0124S). Dynabeads-protein A (Invitrogen, cat#10001D) were transferred to small tubes and washed two times with IP buffer (50 mM Tris-HCl pH 7.5, 500 mM NaCl, 1 mM CaCl_2,_ 1 mM MgCl_2_ and 0.1% Triton X-100). Normal rabbit IgG, anti-KDM2A, anti-H3K36me2 antibodies were incubated with Dynabeads-protein A on a rotator at 4 °C for 2 h. Cell lysates (100 µl) transferred to small tubes were precleared for 1 h with 10 µl of Dynabeads-protein A. Thereafter, cell lysates were incubated with Dynabeads-protein A conjugated antibodies and chromatin immunoprecipitated overnight on a rotator at 4 °C. IP beads were then washed with IP buffer five times, and boiled in SDS loading buffer at 95 °C for 5 min for SDS-PAGE and immunoblot analysis. For DNA sequencing, proteins of IP beads were digested with thermolabile proteinase K (New England Biolabs, #P8111S) at 37 °C for 2 h. DNA was purified using PCR cleanup kit (Qiagen). Purified DNA was used for ChIP-seq library construction with NEB Next ChIP-Seq Library Prep Master Mix Set. ChIP DNA was end-repaired and added with dA tail using Klenow fragment. Sequencing adaptors were ligated to the dA-tailed libraries, and the libraries raging around 280 bp were selected using AMPure XP beads (Beckman Coulter). Size-selected libraries were enriched by PCR with index primers. The quantity and quality of libraries were checked with Qubit and real-time PCR for quantification and bioanalyzer for size distribution detection. Quantified libraries were pooled and sequenced on Illumina platforms, according to effective library concentration and data amount required.

### ChiP-seq analysis

ChIPseeker software was used to calculate the distribution of peaks in various functional areas. The correspondence between peaks and various functional areas is based on the priority order of promoter, 5’UTR, 3’UTR, Exon, Intron, Downstream, and Intergenic. If the position of a peak is both an exon of one gene and an intron of another gene, according to the above priority, it was considered that the peak is annotated onto the exon rather than the intron. Peak region motif identification analysis was conducted by using Homer software based on genome regions of the peak. We conducted motif identification with lengths of 8, 10, 12, and 14 bp. The folder’s name are the motif analysis results of each experiment. Homer can detect known motifs, but also predict potential unknown (de novo) motifs. De novo predicts the motif by first removing redundancy from the generated motif, and then converting the motif into vector values to calculate the Pearson correlation coefficient. The motif results generated by Homer are displayed using a web page named homerResults.html, and the directory homerResults/ stores all the files and images that the web page depends on; homerResults.html formatted output of de novo motif finding, knownResults.html formatted output of known motif enrichment finding (data relative to Homer analysis are available as Supplementary data).

### RT-qPCR

Total RNA was extracted from GFP-labeled Hepa1-6shCTL and Hepa1-6shKDM2A isolated from tumor-bearing mice using the RNeasy Mini kit (QIAGEN) according to the manufacturer’s instructions and quantified using a Nanodrop 2000 UV-visible spectrophotometer. cDNA was prepared using 20–50 ng/ml total RNA by a RT-PCR using a high-capacity cDNA reverse transcription kit (Applied Biosystems), according to the manufacturer’s instructions. qPCR was performed on cDNA using TaqMan probes specific for G6pdx (Mm00656735_g1, ThermoFisher Scientific), Shmt1 (Mm07296291_g1), Shmt2 (Mm00659512_g1), Mthfd1 (Mm00507092_m1), qPCR was performed on a 7900 HT Fast Real-Time PCR System (Applied Biosystems). Fold changes in expression were calculated by the Delta Delta Ct method using 18S rRNA (ThermoFisher, #AM1716) as an endogenous control for mRNA expression. We considered samples value negative, those samples with a value of 2^−^^ΔCt[CtCHK2-Ct18S]^ ≤ to mean 2^-ΔCt^ of controls.

### Protein extraction and immunoblot analysis

Cells were washed with ice-cold PBS twice to completely remove residual medium. RIPA lysis buffer (150 mM NaCl, 1%NP-40, 0.5% sodium deoxycholate, 0.1% SDS and 50 mM Tris pH 8.0) supplemented with protease inhibitors was directly added to cell layers and scraped on ice. Cell lysates were transferred to small tubes and lysed on ice for 20 min before being cleared by top-speed centrifugation(20,000 g) at 4 °C. Protein concentrations in lysates were measured by Bio-Rad protein assay and adjusted equally between samples, followed by the addition of SDS loading buffer (5X) and boiling at 95 °C for 5 min. Equal volume and equal quantity of protein samples were subjected to SDS-PAGE and transferred to a PVDF membrane. The membrane was blocked in EveryBlot blocking buffer (BioRad#12010020) at room temperature for 5 min and incubated with appropriate antibodies at 4 °C overnight. On the next day, the membrane was washed with PBS-T three times and incubated with appropriate secondary HRP antibodies at room temperature for 1 h. The membrane was washed again with PBS-T three times, and chemiluminescent substrate was applied for development.

### Immunofluorescence

Cells grown on glass coverslips were fixed in ice-cold methanol for 5 min at −20 °C, washed twice with PBS at RT, blocked 60 min in 3% BSA with 0.05% NP-40, and immunostained with appropriate antibodies. Coverslips were mounted in MOWIOL 4-88 (Calbiochem) containing 1 μg/ml Hoechst 33342. Fluorescence images were taken by using a widefield fluorescence microscope (Axiovert 200 M; Carl Zeiss) equipped with a 100x Plan Apochromat 1.4 NA oil immersion lens (Carl Zeiss) and a xenon arc lamp with monochromator.

### Flow cytometry

Liver tumors were excised on day 14 post injection and cut into small pieces and incubated with 1 mg/ml collagenase (Thermofisher) and 50 U/ml DNase I in PBS for 30 min at 37 °C. Samples were filtered through 70 µm nylon strainers, and red blood cells were lysed using 0.85% ammonium chloride before resuspension in FACS buffer (PBS with CaCl_2_ and MgCl_2_Sigma-Aldrich D8662) supplemented with 4% FBS. Cells were incubated in FACS buffer for 30 min on ice with fluorescently labeled antibodies to F4/80 PE-rat monoclonal (BM8, BioLegend 123110), CD4 PE-rat monoclonal (RM4-5, BioLegend, 100512), CD8b PE-rat monoclonal (H35-17.2, Abcam, ab95772), Foxp3 PE-rat monoclonal (FJK-16s, ThermoFisher 12-5773-82) intracelluar staining using the eBioscience Foxp3 transcription factor staining buffer set (cat #00-5523-00), normal rat IgG (N104, Sigma-Aldrich).

### NADP^+^/NADPH, Lactate and H_2_O_2_ measurements

Cells were plated at 0.1 × 10^6^ cells/ml in DMEM containing 10% FBS and treated as required. NADP^+^/NADPH, Lactate and H_2_O_2_ were assayed using a quantification colorimetric kit (BioVision) according to the manufacturer’s instructions. NADP^+^/NADPH colorimetric assay kit #K347. Lactate assay kit #K607. H_2_O_2_ colorimetric assay kit #K265.

### Mitochondrial membrane potential

Cells were plated at 0.1 × 10^6^ cells/ml in DMEM containing 10% FBS. TMRM cell-permeable fluorescent dye (Molecular Probes, cat#T668) was use. DMEM/PBS was removed and replaced with TMRM-containing media (100 nM) and incubated at 37 °C in the dark for 30 min. The cell-permeable MitoTracker Green FM (Molecular Probes, cat#M7514) was employed to label mitochondria. Cells were imaged on a wide-field fluorescence microscope (Axiovert 200 M; Carl Zeiss).

### Cell growth assay

Cell growth was done by plating 2 × 10^4^ cells on 12-well plates, in DMEM with 10%FBS. The number of cells was determined at each time point by counting with a BioRad TC20 automated cell counter every 2 days. Cell viability was measured by trypan blue dye exclusion.

### RNA-seq

Total RNA was isolated from Huh7shCTL and Huh7shCHK2 cultured in vitro (*n* = 1), or GFP-labeled Hepa1-6shCTL and Hepa1-6shKDM2A isolated from tumor-bearing mice (*n* = 1) using the RNeasy Mini kit (QIAGEN) according to the manufacturer’s instructions and quantified using a Nanodrop 2000 UV-visible spectrophotometer. Messenger RNA was purified from total RNA using poly-T oligo-attached magnetic beads. After fragmentation, the first strand cDNA was synthesized using random hexamer primers, followed by the second strand cDNA synthesis using either dUTP for directional library or dTTP for non-directional library. For the non-directional library, it was ready after end repair, A-tailing, adapter ligation, size selection, amplification, and purification. For the directional library, it was ready after end repair, A-tailing, adapter ligation, size selection, USER enzyme digestion, amplification, and purification. The library was checked with Qubit and real-time PCR for quantification and bioanalyzer for size distribution detection. Quantified libraries were pooled and sequenced on Illumina NovaSeq X plus (25B), according to effective library concentration and data amount. Data relative to RNA-seq analysis are available as Supplementary data.

### Bioinformatics Analysis

#### RNA-seq analysis

Raw data (raw reads) of fastq format were firstly processed through in house perl scripts. In this step, clean data (clean reads) were obtained by removing reads containing adapter, reads containing ploy-N and low-quality reads from raw data. At the same time, Q20, Q30 and GC content the clean data were calculated. All the downstream analyses were based on the clean data with high quality. To quantify gene expression levels, featureCounts [30] v1.5.0-p3 was used to count the reads numbers mapped to each gene. And then FPKM of each gene was calculated based on the length of the gene and reads count mapped to this gene. FPKM, expected number of Fragments Per Kilobase of transcript sequence per Millions base pairs sequenced, the effect of sequencing depth and gene length fort the reads count at the same time, and is currently the most commonly used method for estimating gene expression levels.

#### Differential expression analysis

Differential expression [31] analysis of conditions was performed using the DESeq2 R package (1.20.0). DESeq2 provide statistical routines for determining differential expression in digital gene expression data using a model based on the negative binomial distribution. The resulting *p*-values were adjusted using the Benjamini and Hochberg’s approach for controlling the false discovery rate. Genes with an adjusted *p*-value < 0.05 found by DESeq2 were assigned as differentially expressed. Prior to differential gene expression analysis, for each sequenced library, the read counts were adjusted by edge R program package through one scaling normalized factor. Differential expression analysis of two conditions was performed using the edge RR package (3.22.5). The *P*-values were adjusted using the Benjamini and Hochberg method. Corrected *p*-value of 0.05 and absolute foldchange of 2 were set as the threshold for significantly differential expression.

#### Enrichment analysis of differentially expressed genes

Gene Ontology [32] (GO) enrichment analysis of differentially expressed genes was implemented by the clusterProfiler R package, in which gene length bias was corrected. GO terms with corrected *P*-values less than 0.05 were considered significantly enriched by differential expressed genes. KEGG is a database resource for understanding high-level functions and utilities of the biological system, (http://www.genome.jp/kegg/). We used clusterProfiler R package to test the statistical enrichment of differential expression genes in KEGG [33] pathways.

#### Gene Set enrichment analysis

The genes were ranked according to the degree of differential expression in the two samples, and then the predefined Gene Set were tested to see if they were enriched at the top or bottom of the list. Gene set enrichment analysis can include subtle expression changes. We used the local version of the GSEA analysis tool http://www.broadinstitute.org/gsea/index.jsp, GO, KEGG data sets were used for GSEA independently.

#### Cancer Genome Atlas (TCGA) data analysis

The TCGA-LIHC cohort, containing 371 cases, was used to validate KDM2A expression levels in distinct subtypes of HCC. The TCGA database (https://gdc-portal.nci.nih.gov) was accessed through the cbioportal website (http://www.cbioportal.org/) to retrieve patients. The TCGA_participant_barcode was used to download KDM2A expression from RNA-seq data using the firebrowse portal (http://firebrowse.org). Statistical analysis was performed using the Mann-Whitney test with GraphPad 7 software.

#### Statistical analysis

The data are presented mean ± SEM from at least three independent experiments with similar results. All presented immunofluorescence images are representative images from three representative experiments. For results encompassing multiple groups, one-way ANOVA was employed. For other experiments, statistical significance was determined using *t* -test. *P* values of less than 0.05 were considered significant. Plots and statistical analysis were constructed using software GraphPad Prism Version 7.0.

## Results

### KDM2A overexpression correlates with intratumoral CD163^+^ macrophages and CD8^+^ T cells peritumoral localization

**Fig. 1:**
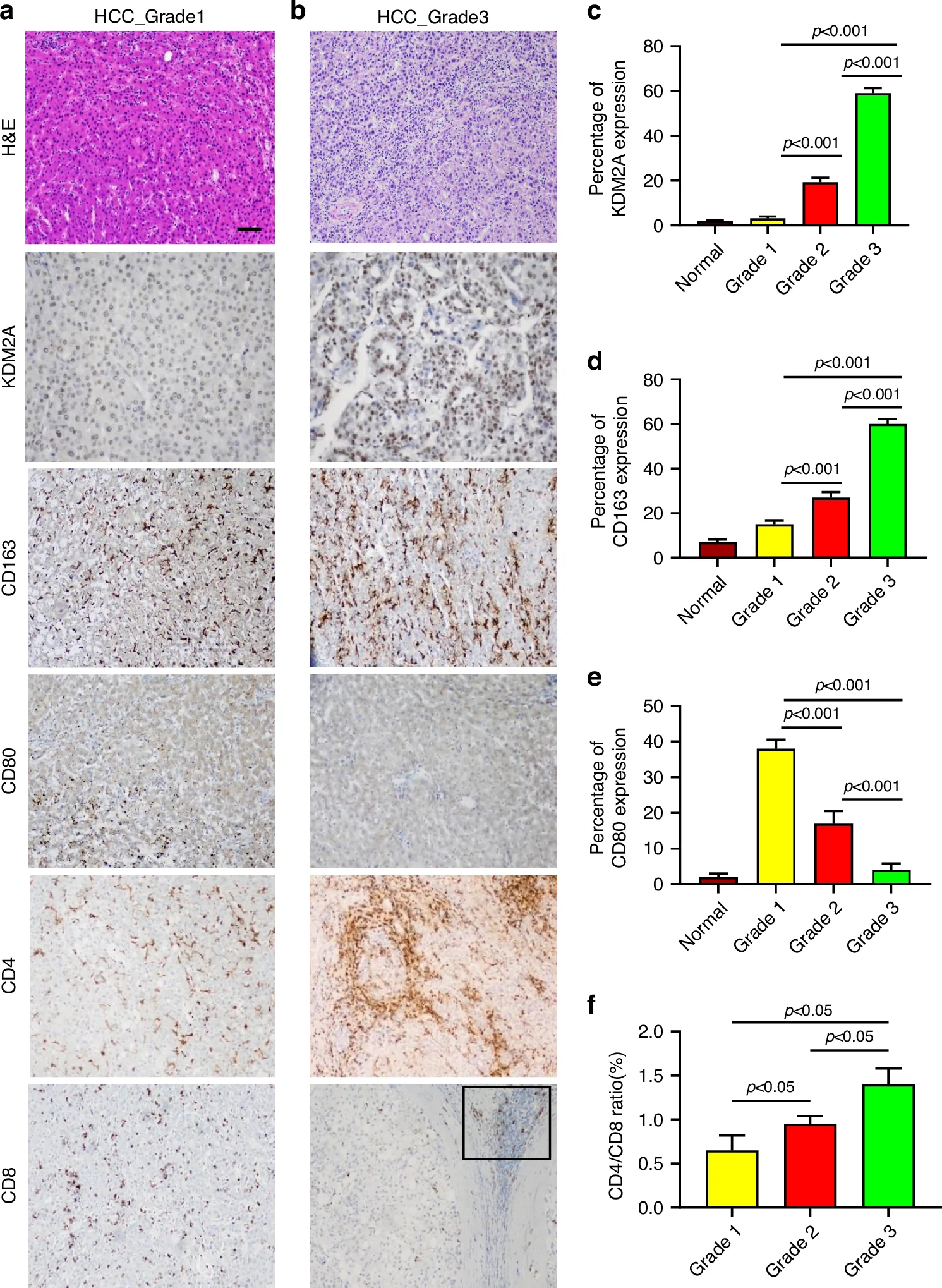
KDM2A overexpression is associated with a M2-macrophages intratumoral infiltration and a peritumoral CD8^+^T cells localization. **a**, **b** Representative images of histological staining of human hepatocellular carcinoma (HCC) grade 1 (**a**) and grade 3 (**b**) with H&E and antibodies against KDM2A, CD163, CD80, CD4, CD8. Scale bar, 500 µm. **c** Quantification of HCC cells nuclei expressing KDM2A, (HCC patients, *n* = 42). **d** Quantification of CD163^+^ cells infiltrating HCC tissue, (HCC patients, *n* = 42). **e** Quantification of CD80^+^ cells infiltrating HCC tissue, (HCC patients, *n* = 42). **f** CD4^+^and CD8^+^percentage ratio of cells infiltrating HCC tissue, (HCC patients, *n* = 42). The frame indicates peritumoral stroma. All values are shown as mean ± SD. The data were analyzed by one-way ANOVA and Shapiro-Wilk test to evaluate the presence a normal distribution of data.

To address the significance of KDM2A in hepatocellular carcinoma, we examined the distribution of KDM2A protein and leukocytes infiltrate in an Italian cohort of human HCC specimens (Fig. 1a, b and Suppl. Fig. 1A, B). We found that KDM2A expression increased significantly with HCC grading (low grade G1 3.2 ± 1.6%, versus high grade G3 59.1 ± 2.2%, *p* < 0.001) (Fig. 1c). To assess the link between KDM2A expression and anti-tumor immunity, we quantified leukocyte infiltration in intra- and peritumoral areas, finding an increased expression of CD163^+^ cells, a marker of M2-like macrophages, infiltrating intratumoral tissue (G1 16.0 ± 1.6% versus G3 56.0 ± 8.6%, *p* < 0.001) (Fig. 1d). Interestingly, increased expression of KDM2A correlated with reduced intratumoral infiltration of CD80^+^ cells, a marker of activated antigen-presenting cells (APCs) (G1 39.3 ± 1.64% versus G3 3.0 ± 1.4%, *p* < 0.001) (Fig. 1e). Consistently with these latter findings, we observed a significant increase of CD4^+^/CD8^+^ T cells ratio (G1 0.8 ± 0.02 versus G3 1.3 ± 0.06, *p* < 0.05) (Fig. 1f). Of note, CD8^+^ T cells, rarely represented into intratumoral tissue were accumulated prevalently in the peritumoral stroma in G3 hepatocellular carcinoma.

**Fig. 2:**
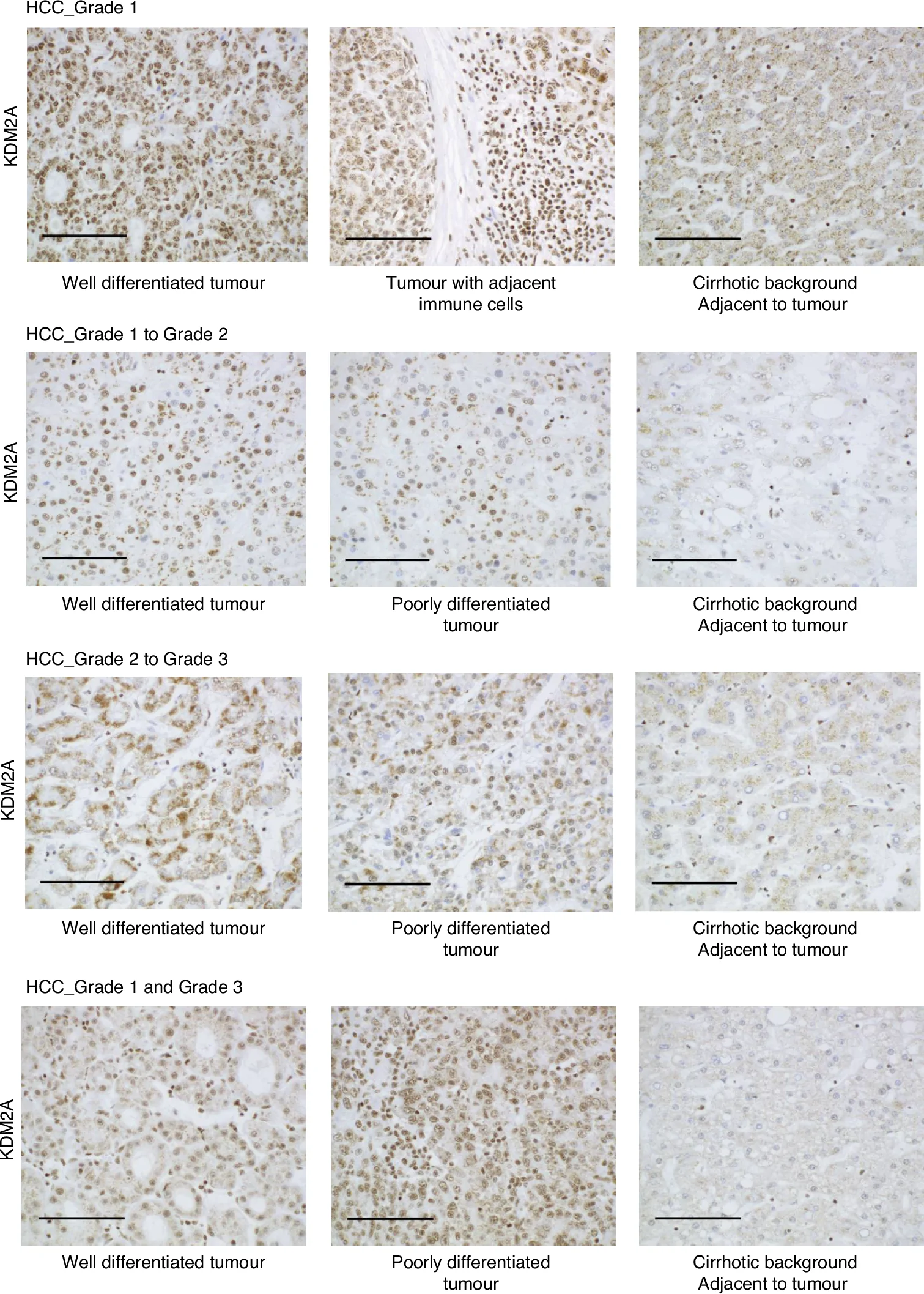
KDM2A increased expression is associated to HCC histological grading (UK cohort). Representative images of histological staining of human HCC grade 1, 2 and grade 3, all with cirrhotic background adjacent to tumor tissue (*n* = 20) scale bar, 100 µm. Slide were viewed on a Zeiss Axioskop 50 using the 40x objective and images taken with an attached Zeiss Axiocam ICC5 digital camera.

To determine whether the association between KDM2A and immune infiltration was maintained within tumors of the same histological grade, we performed a grade-stratified analysis of the Italian cohort. Within each grade, tumors were divided into KDM2A-low and KDM2A-high groups using the grade-specific median KDM2A value. This analysis revealed limited intra-grade variability of KDM2A expression, with low-grade tumors showing uniformly low KDM2A levels and high-grade tumors showing consistently elevated KDM2A levels (data not shown). Therefore, a statistically robust comparison of immune infiltrates between KDM2A-low and KDM2A-high tumors within the same grade was not feasible. These findings indicate that KDM2A expression is strongly associated with histological grade in this cohort. The UK validation cohort confirmed that KDM2A expression increased with HCC grading, i.e., well differentiated versus poorly differentiated tumors, with a strong expression intratumorally versus the cirrhotic background adjacent to the tumor (Fig. 2).

**Fig. 3:**
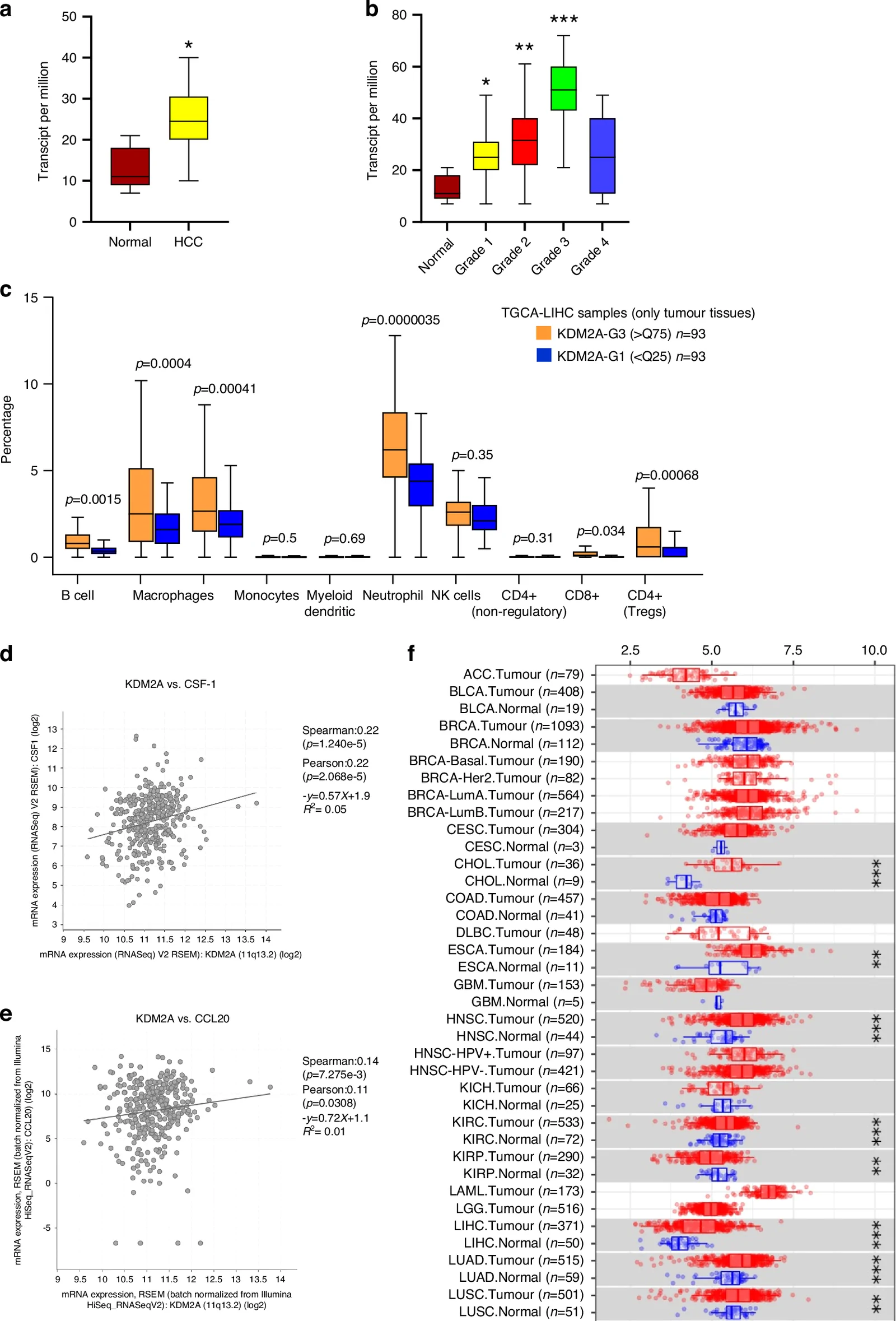
KDM2A expression level is associated with macrophages and Treg cells infiltrate in liver cancer. **a** Analysis of KDM2A RNA expression in HCC (*n* = 371) and normal (*n* = 40) tissues from patients of TGCA dataset. **b** Expression of KDM2A RNA in HCC tissues from patients of TGCA dataset based on tumor grade. Grade 1, *n* = 54; grade 2, *n* = 173; grade 3, *n* = 118; grade 4, *n* = 10. *p < 0.05,**p < 0.001, ***p < 0.0001. **c** Quantification of tumor-infiltrating immune cells by using quanTIseq. Analysis is performed on RNA-seq data from tumor samples of HCC patients collected from TCGA dataset. **d**, **e** Correlation analysis of mRNA levels of KDM2A vs CSF1 and KDM2A vs CCL20 in HCC patients using TGCA dataset. **f** Analysis of KDM2A mRNA expression in tumors and normal tissues from patients with indicated types of cancers in TCGA dataset,**p < 0.001, ***p < 0.0001.

Analysis of TCGA datasets showed that *KDM2A* is rarely mutated but its mRNA is overexpressed in several tumors, including HCC (Fig. 3a). TCGA dataset query also showed that, in HCC patients, *KDM2A* mRNA levels increased with histological grade (Fig. 3b), in agreement with our experimental data (Suppl. Fig. 1). Using quanTIseq on TCGA hepatocarcinoma datasets, we found that *KDM2A* overexpression was significantly associated with increased infiltration of macrophages, neutrophils, and regulatory T cells (Tregs) in grade-1 and grade-3 tumors (Fig. 3c). Additionally, we assessed the expression of CSF1, a cytokine involved in macrophage recruitment and maintenance, and CCL20, a chemokine implicated in T17h and Treg cell recruitment. *KDM2A* mRNA levels positively correlated with that of both *CSF1* mRNA (Spearman’s *ρ* = 0.22, *p* = 0.00002) and *CCL20* mRNA (Spearman’s *ρ* = 0.14, *p* = 0.007) (Fig. 3d, e). Moreover, *KDM2A* was found to be overexpressed in a variety of human cancers, including cholangiocarcinoma (Fig. 3f).

### KDM2A maintains genomic stability by regulating mitochondrial function and antioxidant capacity

**Fig. 4:**
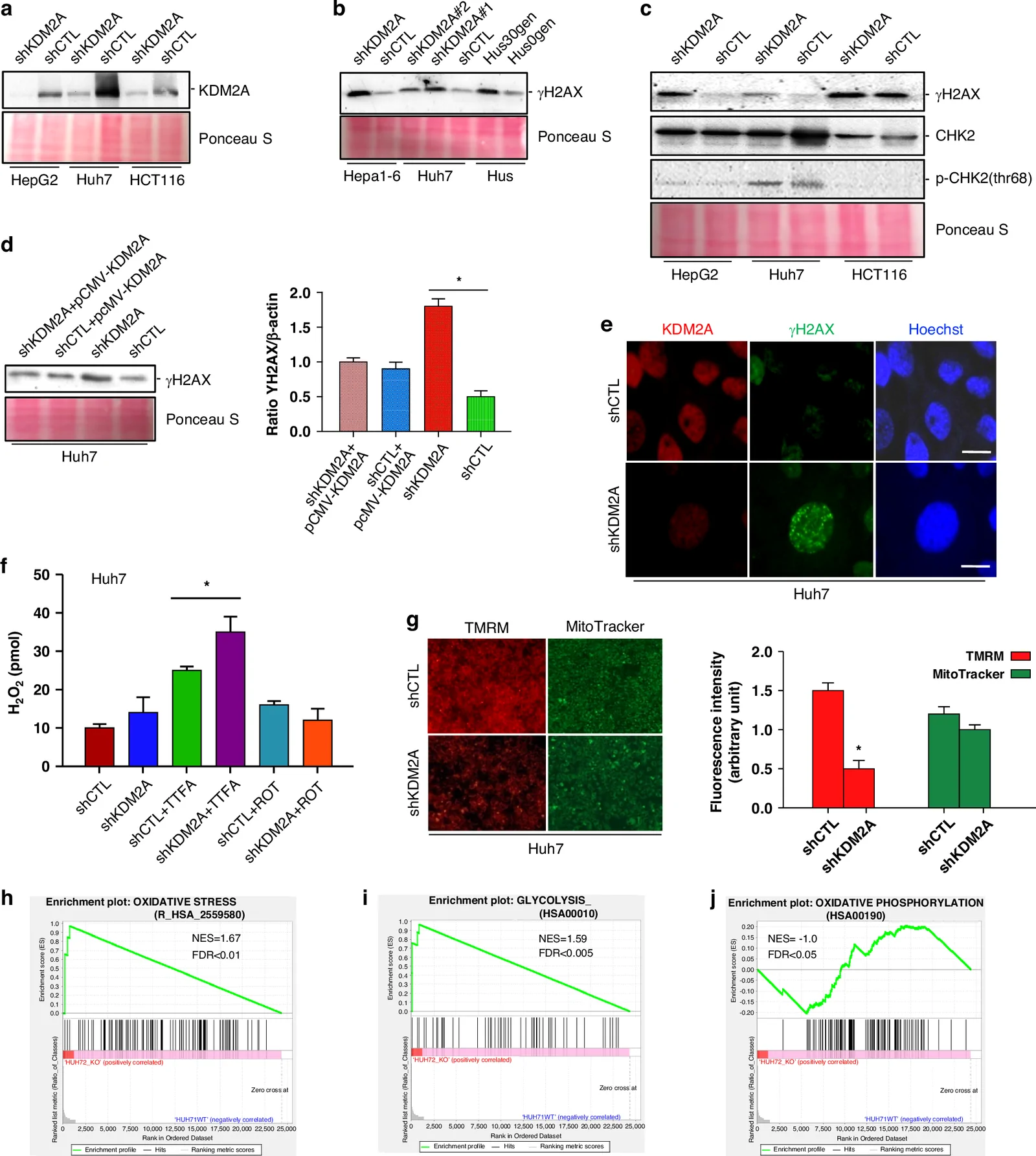
KDM2A depletion induces mitochondrial dysfunction and DNA damage. **a** Representative immunoblots of KDM2A in HCT116, Huh7 and HepG2 transduced with shRNA against KDM2A (shKDM2A) and scramble (shCTL), *n *= 3. **b** Representative immunoblots of DNA damage marker γH2AX in immortalized hepatocytes (Hus0gen, low passage in culture; Hus30gen, high passage in culture), Huh7 transduced with shRNA against KDM2A (shKDM2A) and scramble (shCTL), murine Hepa1-6 transduced with shRNA against KDM2A (shKDM2A) and scramble (shCTL), *n* = 5. **c** Representative immunoblots of HepG2, Huh7, HCT116 cells depleted for KDM2A. Huh7 and HepG2 cells show increased expression of γH2AX and activation of CHK2 (p-CHK2thr68), HCT116 cells show constitutive expression of γH2AX and are unable to activate CHK2, *n* = 3. **d** Representative immunoblots of KDM2A-depleted Huh7 cells transfected with a plasmid expressing a KDM2A cDNA resistant to RNA interfering *n* = 3. Quantitative analysis of band intensities and statistical significance of expression levels were performed *p < 0.05. **e** Immunofluorescent images show increased setting up of γH2AX foci in the nuclei of KDM2A depleted Huh7 cells, scale bar 10 µm. **f** Generation of H_2_O_2_ in basal condition and after treatment with electron transport chain complex II inhibitor 2-thenoyl-trifluoroacetone (TTFA), and complex I inhibitor rotenone (ROT); mean ± SEM.*p < 0.01, as determined by unpaired Student’s t-test. **g** Cells were stained with TMRM (red) and MitoTracker (green) for 30 min and analyzed by fluorescent microscopy. The intensity of TMRM staining reflects the mitochondrial membrane potential. The fluorescent signal of MitoTracker reflects the mitochondrial mass. Quantification of fluorescence intensity was performed with Image J software. *p < 0.01 vs Huh7shCTL. **h–j** GSEA analysis, gene list was ranked with signed (from log_2_ FC) probability ratio from KDM_2_A depleted Huh7 cells versus control comparison. FDR false discovery rate, NSE normalized enrichment score.

To test the impact of KDM2A in maintaining genome integrity, we depleted KDM2A in HepG2, Huh7 and HCT116 cells (Fig. 4a), and measured γH2AX, a marker of DNA damage, through immunoblotting and immunofluorescence (Fig. 4b–e). Figure 4b shows that γH2AX increased in immortalized hepatocytes Hus after 30 culture passages as well as after *KDM2A* knock-down in Huh7 and in the murine cell line Hepa1-6. KDM2A loss induced DNA damage also in HepG2 cells, while genomically unstable HCT116 cells showed constitutively high γH2AX (Fig. 4c). Importantly, KDM2A-depleted Huh7 and HepG2 cells also showed activation of phospho-CHK2 (Thr68), a key effector of the DDR (Fig. 4c). Expression of a KDM2A variant resistant to RNA interference prevented this damage, confirming KDM2A’s protective role (Fig. 4d).

To investigate the source of DNA damage, we assessed mitochondrial function, as mitochondria generate reactive oxygen species (ROS) and antioxidants during oxidative phosphorylation to maintain redox balance. Thus, measuring intracellular levels of H_2_O_2_ in Huh7 we did not find a significant increase in KDM2A-depleted cells (Fig. 4f). To evaluate if H_2_O_2_ production was directly due to mitochondria, Huh7 cells were treated with rotenone and 2-thenoyl-trifluoroacetone (TTFA), inhibitors of electron transport chain (ETC) complex I and complex II, respectively (4). TTFA treatment only increased significantly intracellular levels of H_2_O_2_ in control and consistently in KDM2A-depleted cells. Mitochondrial function assessed via Δψm (mitochondrial membrane potential) analysis using TMRM fluorescence showed a reduction in Δψm in KDM2A-depleted Huh7 cells, indicating mitochondrial dysfunction (Fig. 4g).

Transcriptomic analysis further revealed that *KDM2A* knockdown altered metabolism-related gene expression in Huh7 cells. GSEA analysis showed that KDM2A depletion determined the upregulation of oxidative stress and glycolysis pathways (Fig. 4h, i), and downregulation of electron transport chain complex I genes (Fig. 4j), supporting impaired mitochondrial function.

**Fig. 5:**
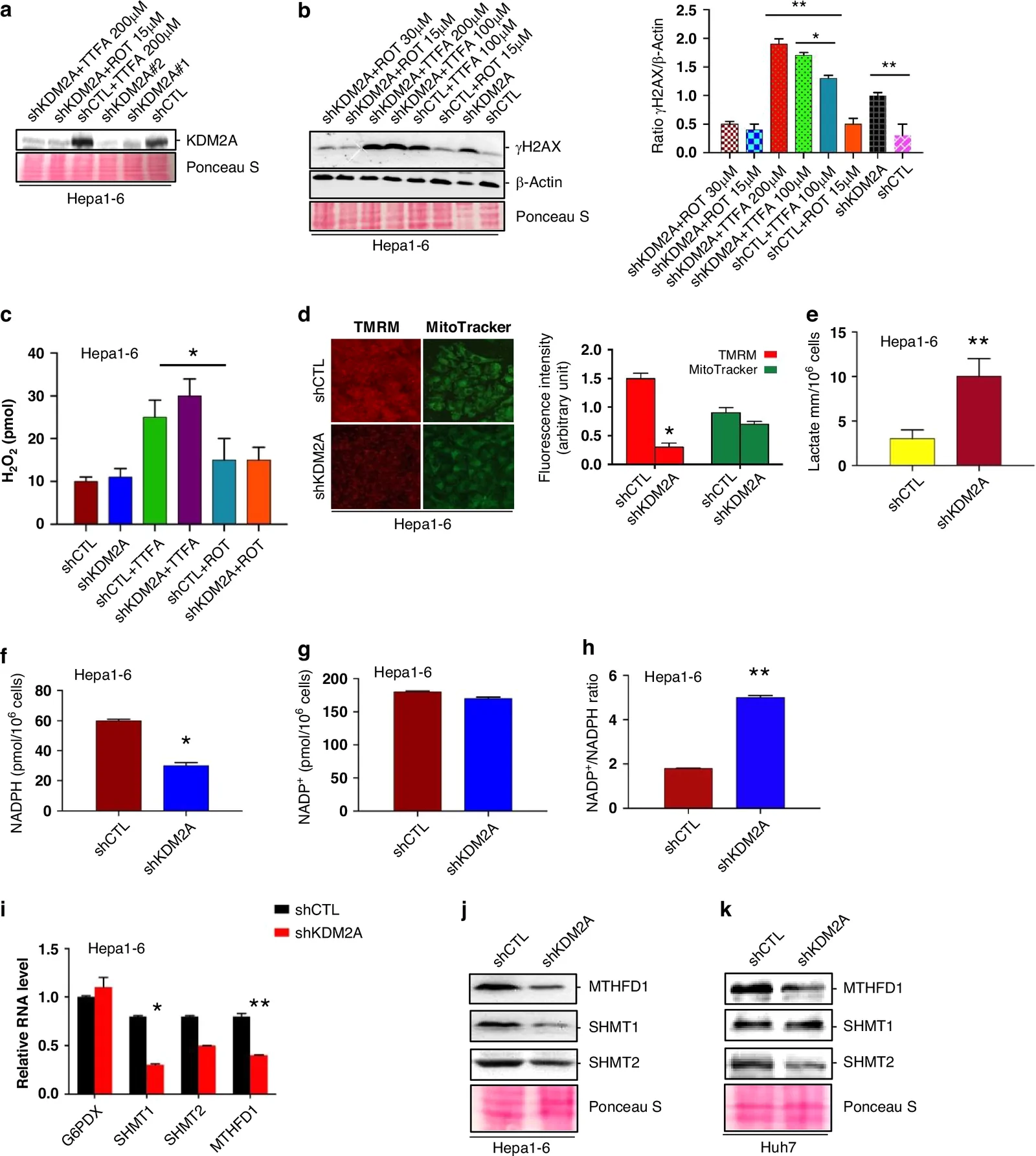
KDM2A depletion causes defective one carbon folate pathway and enhances glycolysis. **a** Representative immunoblots of KDM2A expression level in murine Hepa1-6 cells transduced with shRNA against KDM2A (shKDM2A) and scramble (shCTL), treated with 2-thenoyl-trifluoroacetone (TTFA) and rotenone (ROT), *n* = 3. **b** Representative immunoblots of γH2AX expression in Hepa1-6 transduced with shRNA against KDM2A (shKDM2A) and scramble (shCTL) treated with 2-thenoyl-trifluoroacetone (TTFA) and rotenone (ROT), *n* = 3. Quantitative analysis of band intensities and statistical significance were performed *p < 0.05, **p < 0.01. **c** Generation of H_2_O_2_ in KDM2A depleted Hepa1-6 cells and after treatment with complex II inhibitor TTFA and complex I inhibitor rotenone (ROT); mean ± SEM.*p < 0.01 as determined by unpaired Student’s t -test. **d** Cells were stained with TMRM (red) and MitoTracker (green) for 30 min and analyzed by fluorescent microscopy. The intensity of TMRM staining reflects the mitochondrial membrane potential. The fluorescent signal of MitoTracker reflects the mitochondrial mass. Quantification of fluorescence intensity was performed with Image J software. *p < 0.01 vs Hepa1-6shCTL. **e** The lactate production in cell lysates was determined using a colorimetric assay kit. The data represent mean ± SEM, *n* = 3 **p < 0.01. **f–h** The NAPD^+^/NADPH production in cell lysates was determined using a colorimetric assay kit. The data represent mean ± SEM, *n* = 3 **p < 0.01. **i** The real time qPCR analysis of transcripts of G6PDX, SHMT1, SHMT2, MTHFD1 in Hepa1-6 cells transduced with shRNA control (shCTL) and shRNA against KDM2A (shKDM2A). The RT-qPCR data were normalized to 18S rRNA and presented as fold changes of gene expression in the sample compared to the control. The data represent mean ± SEM, *n* = 3 *p < 0.05, **p < 0.01. **j**, **k** Representative immunoblots of one-carbon folate pathway enzymes expression in Hepa1-6 and Huh7 cells transduced with shRNA control and shRNA against KDM2A, *n* = 3.

To confirm these findings in another experimental model, as already mentioned, we generated Hepa1-6 mouse hepatocarcinoma cells stably depleted of KDM2A (shKDM2A). Rotenone and TTFA did not affect shRNA activity (Fig. 5a). *KDM2A* knockdown again induced an increase of γH2AX levels, and TTFA modulated its levels (Fig. 5b, see also Fig. 5a and Fig. 4b). However, H₂O₂ levels were not significantly increased (Fig. 5c). Still, Δψm was reduced, and lactate production was elevated, both markers of dysfunctional mitochondria (Fig. 5d, e and Suppl. Fig. 2).

To explain the DNA damage in the absence of increased H₂O₂ production, we hypothesized a defective antioxidant system. We measured NADPH, a key redox cofactor [34], and found significantly reduced levels in KDM2A-depleted Hepa1-6 cells (Fig. 5f–h). Since NADPH is generated via the pentose phosphate pathway (PPP) and one-carbon (1 C) metabolism, we performed qPCR analysis for *G6PD*, *SHMT1/2* and *MTHFD1/MTHFDL1* (1 C enzymes) [35, 36]. KDM2A depletion caused a notable reduction in *SHMT1* and *MTHFD1* expression, both at mRNA and protein levels in Hepa1-6 cells (Fig. 5I, J), and down-regulation of SHMT2 and MTHFDL1 in Huh7 cells (Fig. 5k). These findings demonstrate a clear link between KDM2A and mitochondrial function.

### KDM2A depletion modifies molecular determinants of immune response

**Fig. 6:**
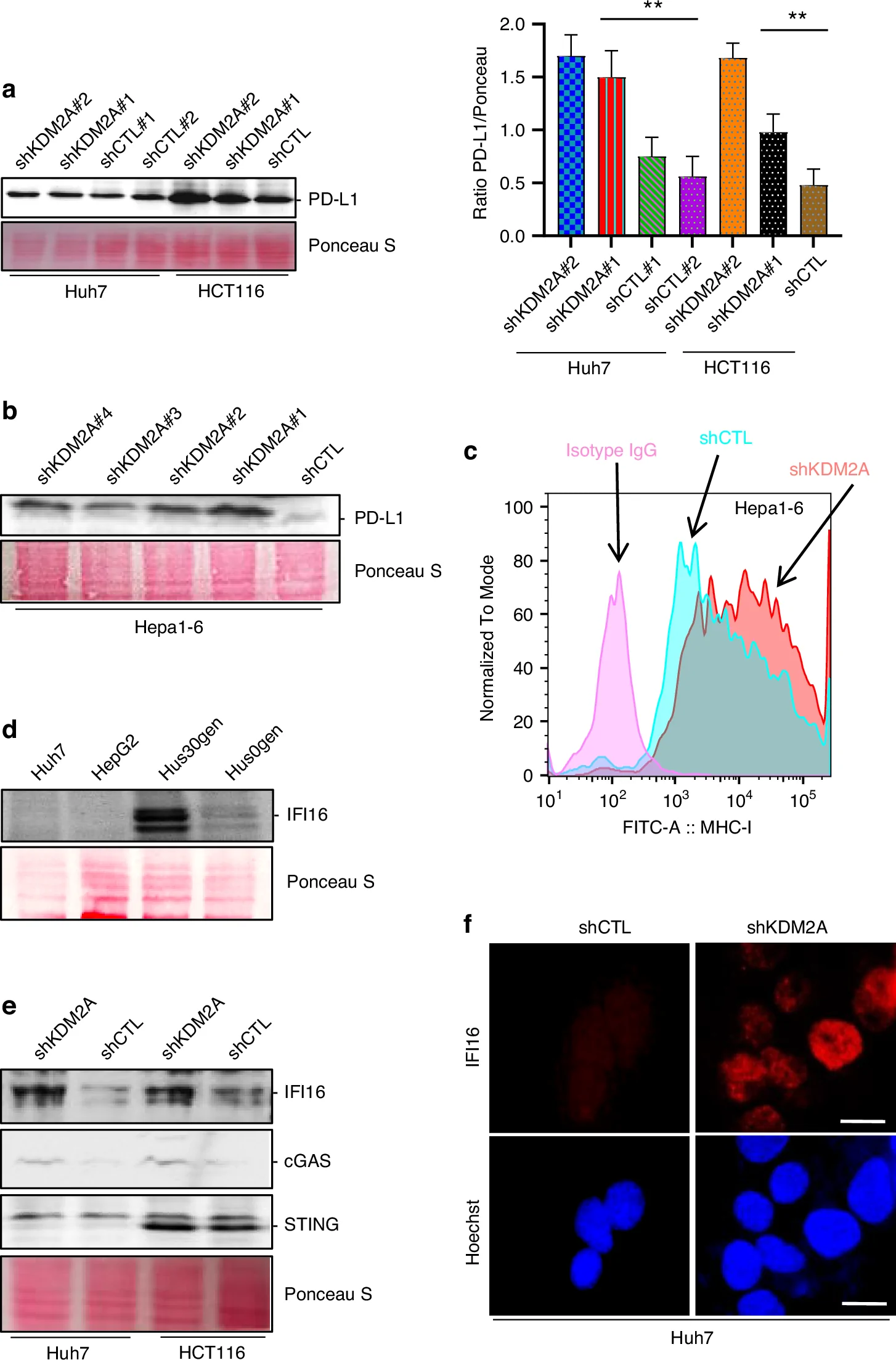
DNA damage associated to KDM2A depletion modifies molecular determinants of immunity. **a** Measurement of PD-L1 protein expression by immunoblot in HCT116 and Huh7 cells with indicated manipulations; PVDF membrane stained with Ponceau is shown as the loading control for all proteins, *n* = 3. Quantification of protein bands was performed after normalization by using Image J software, **p < 0.01. **b** Representative immunoblots of PD-L1 expression in Hepa1-6 transduced with different shRNA against KDM2A (shKDM2A) and scramble (shCTL). **c** Flow cytometry analysis of MHC-I expression in scrambled and KDM2A-depleted Hepa1-6 cells. **d** Immunoblot analysis of expression of dsDNA sensor IFI16 in Hus0gen, Hus30gen, and parental cell lines HepG2, Huh7, HCT116. **e** Representative immunoblots of IFI16, cGAS, STING1 expression in cells transduced with scramble or KDM2A shRNA. **f** Representative immunofluorescent images show increased expression and nuclear localization of dsDNA sensor IFI16 in Huh7 cells with indicated manipulations.

We next investigated biological consequences of KDM2A depletion-induced DNA damage, and if endogenous DNA damage could trigger a cell-intrinsic immune response. To address this issue, we first analyzed transcriptomic data in KDM2A-depleted Huh7 cells. Gene ontology (GO) enrichment analysis of differential expressed genes showed that the downregulated genes in GO terms were significantly related to humoral inflammatory response, regulation of inflammatory response and acute inflammatory response further confirmed by GSEA analysis (Suppl. Fig. 3). We then evaluated the expression of PD-L1, the target of several anti-cancer treatments, and MHC class I molecules involved in the adaptive branch of the immune system. We found an upregulation of PD-L1 in KDM2A-depleted HuH7 and HCT116 cells (Fig. 6a), conversely MHC class I components were not significantly modified (data not shown). KDM2A depletion in Hepa1-6 cells caused an upregulation of both PD-L1 and MHC class I (Fig. 6b, c). Thus, to investigate the mechanism by which DNA damage caused a cell intrinsic-immune response, we speculated that DNA damage sensors could be involved. The key intracellular DNA sensors are cyclic guanosine monophosphate (GMP)-AMP synthase (cGAS), interferon gamma inducible protein 16 (IFI16) and absent in melanoma 2 (AIM2). IFI16 and AIM2 bind DNA in a non-sequence-specific manner using a 200-aminoacid domain, furthermore they are localized in the nucleus and able to translocate in the cytoplasm where they interact with adaptor protein STING, a stimulator of interferon genes. IFI16 has also been described as a protein that associates with DNA damage-related proteins BRCA1, p53 and acts to promote cellular senescence. Therefore, we verified its expression levels in Huh7, HepG2, Hus and HCT116 cells (Fig. 6d, e). Next, we tested if DNA damage linked to KDM2A depletion was able to stimulate IFI16 expression. KDM2A depletion in HCT116 and Huh7 induced IFI16 expression and nuclear localization (Fig. 6E, F). Cells were also evaluated for cytosolic DNA sensor cGAS, but they did not express the protein in basal condition and after KDM2A depletion (Fig. 6e). It is unknown if IFI16 is involved in the recognition of DNA damage in the nucleus. Our results showed that KDM2A was implicated in the expression of IFI16.

### KDM2A depletion enhances anti-tumor immune response in vivo

**Fig. 7:**
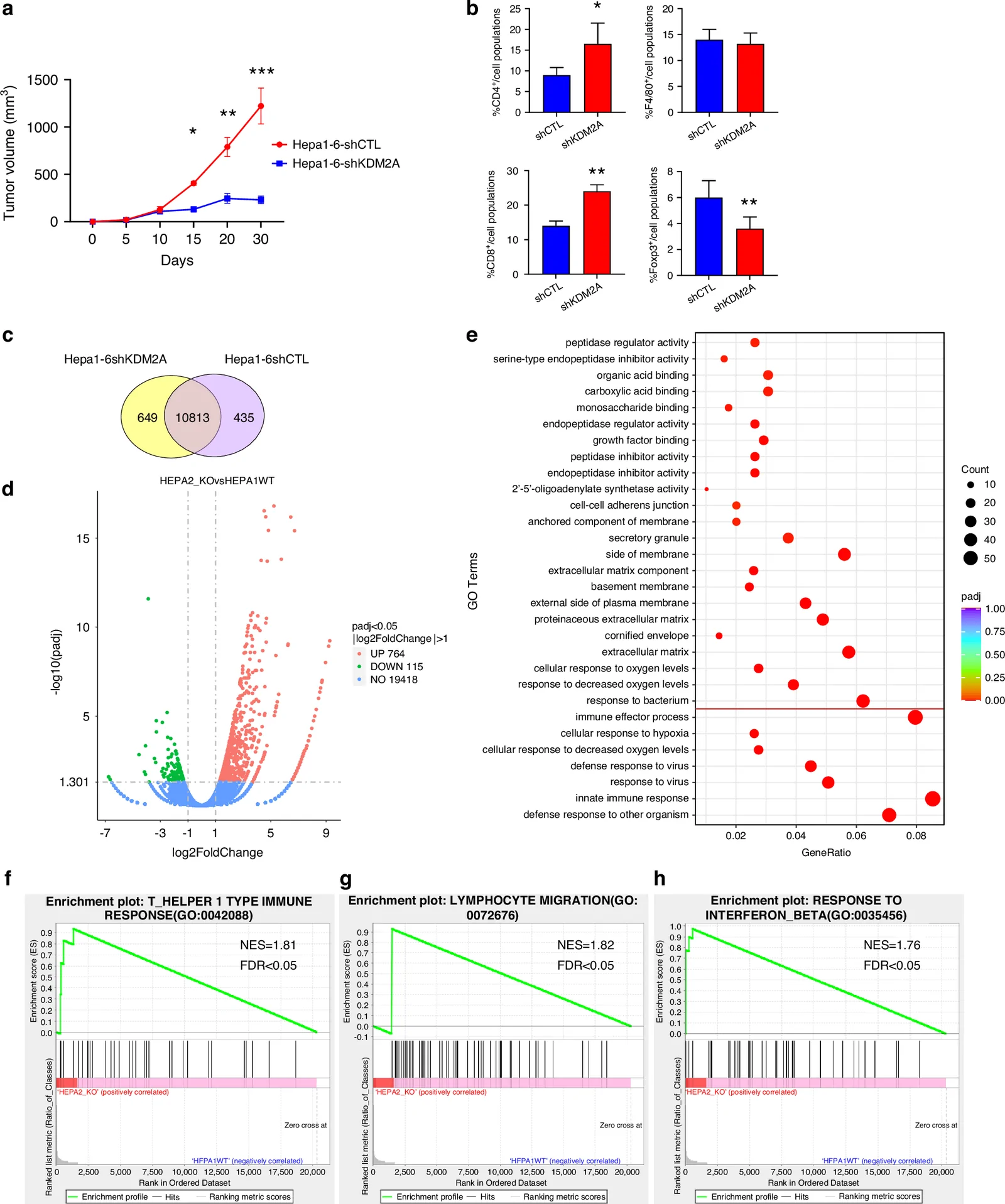
KDM2A depletion enhances anti-tumor immune response. **a** Growth of orthotopic Hepa1-6 tumors in mice inoculated with shRNACTL and shRNAKDM2A HEPA1-6 transduced cells. **b** Tumor infiltrating lymphocytes and macrophages from orthotopic Hepa1-6 tumors (*n* = 5 shRNACTL; *n* = 5 shRNAKDM2A) were analyzed by flow cytometry at day 21 post implantation. **c**, **d** GFP-labeled Hepa1-6 cells (shRNACTL and shRNAKDM2A) were isolated from tumor-bearing mice and subjected to RNA-seq analysis. Venn diagram presents the number of genes that are uniquely expressed within Hepa1-6shKDM2A and Hepa1-6shCTL, with the overlapping regions showing the number of genes that are co-expressed in two cell lines (**c**). Differential gene expression is shown as volcano plot. Dots in red represent increased genes (log_2_FC > 1 and padj < 0.05) and dots in green represent decreased genes (log_2_FC <-1 and padj < 0.05) in KDM2A depleted cells versus control (**d**). **e** Gene Ontology (GO) enrichment analysis of up-regulated genes in KDM2A depleted Hepa1-6 cells versus control, the most significant 30 GO terms were selected for display. In the figure, the abscissa is the ratio of the number of differential genes linked with the GO terms to the total number of differential genes, and the ordinate is GO terms. The size of a point represents the number of genes annotated to a specific GO terms, and the color from red to purple represents the significant level of the enrichment. The GO terms relative to immune response pathways are identified below a red line. **f–h** GSEA analysis for positive regulation of immune response in KDM2A depleted Hepa1-6 cells versus control. FDR false discovery rate, NES normalized enrichment score. All values are shown as mean ± SEM of individual mice per group in one experiment. *p < 0.05, **p < 0.01, ***p < 0.001, as determined by unpaired t -test.

Based on our in vitro findings that KDM2A potentially regulates cellular process related to anti-tumor immune response and to expand these observations we used a syngeneic tumor model by orthotopic liver injection of Hepa 1-6 in mice. KDM2A-depleted Hepa 1-6 cells significantly reduced growth in comparison to control cells measured as tumor size (Fig. 7a). To assess if KDM2A depletion determined a change of immune cell populations infiltrating the tumors, we evaluated by flow cytometry the percentage of immune cell subpopulations infiltrating the tumors. We found in the cell populations isolated from tumors a significant increase in the numbers of CD8^+^T cells concurrent with a significant reduction of Foxp3^+^cells, representing regulatory T cells (Treg cells) (Fig. 7b).

We then in vivo investigated if KDM2A depletion modified tumor cell characteristic associated with anti-tumor immune response. RNA-seq analysis showed that KDM2A depletion significantly altered the gene expression profile in Hepa1-6 cells (Fig. 7c, d). Differential expression analysis showed that immune response processes, including innate immune response, immune effector process and defense response to other organism, were ranked among the top three GO terms in ex vivo KDM2A depleted Hepa1-6 cells, providing evidence for increased tumor immunogenicity (Fig. 7e). In addition, genes associated with immune responses were enriched in KDM2A depleted Hepa1-6 as analyzed by GSEA (Fig. 7f–h).

All together, these findings highlighted how tumor cells can orchestrate immune response by modulating KDM2A expression/function.

### Depletion of KDM2A causes increases in H3K36me2 at CpG islands and redistribution at gene promoters

**Fig. 8:**
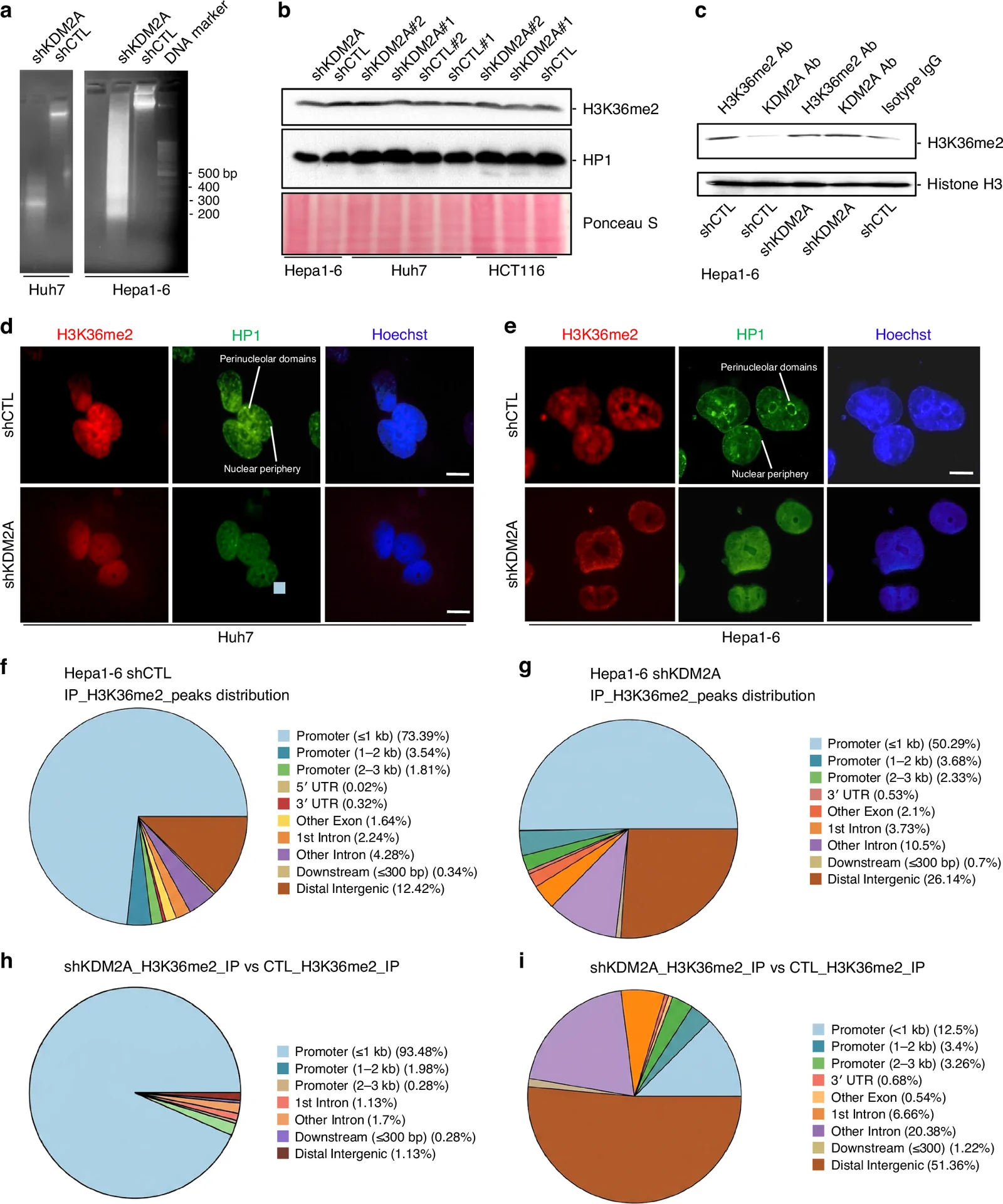
KDM2A depletion causes redistribution of H3K36me2 at gene promoters. **a** DNA analysis on agarose gel showed that chromatin isolated from Huh7shKDM2A and Hepa1-6shKDM2A cells was efficiently digested by restriction enzyme HinP1I, which selectively cleaves unmethylated CpG sites. In contrast, chromatin from Huh7shCTL and Hepa1-6shCTL cells was resistant to HinP1I digestion, indicating a predominance of CpG methylation in control cells. **b** Immunoblot of bulk chromatin released from purified nuclei using antibodies against H3K36me2 and heterochromatin P1 (HP1). Ponceau staining served as a loading control, *n* = 3. **c** Immunoprecipitation of CpG island chromatin isolated from Hepa1-6shCTL and Hepa1-6shKDM2A by using an antibody against H3K36me2 or KDM2A. Precipitated proteins were blotted against H3K36me2. An antibody against histone H3 served as a loading control. **d**, **e** Immunofluorescence analysis of H3K36me2 and HP1 localization in nuclei of Huh7 and Hepa1-6. Depletion of KDM2A causes disappearance of HP1 and H3K36me2 bright-staining foci marking regions of pericentromeric heterochromatin indicating a more relaxed chromatin. DNA is stained by using Hoechst 33342. **f**, **g** Annotation of peaks in gene functional regions, ChIPseeker software was used to calculate the annotation of peaks in various functional areas. The correspondence between peaks and various functional areas is based on the priority order of promoter, 5’UTR, 3’UTR, exon, intron, downstream, and intergenic. **h**, **i** ChIPseeker was used to annotate percentage of downregulated peaks (**h**) and upregulated peaks (**i**) in each functional area of Hepa1-6shKDM2A (shKDM2A) vs Hepa1-6shCTL (CTL).

To identify the chromatin fragments linked to CpG islands we used the methyl CpG sensitive restriction endonuclease HinP1I (cleavage site GCGC). The sites for this enzyme in bulk chromatin are generally uncleavable owing to DNA methylation, thus the released fractions contained prevalently unmethylated CpG islands. KDM2A depleted Huh7 and Hepa 1-6 cells expressed cleavable chromatin by HinP1I and produced unmethylated CpG chromatin fragments, the process was hindered in control Huh7 and Hepa1-6 cells (Fig. 8a). We evaluated the expression levels and localization inside the nuclei of H3K36me2 by comparing it with heterochromatin protein 1 (HP1), a marker of heterochromatin (40). Immunoblotting of proteins isolated from whole nuclei did not show significant differences in the levels of H3K36me2 (Fig. 8b). Immunoprecipitation of CpG chromatin fragments followed by western blotting showed that CpG chromatin fragments were enriched in H3K36me2 in the absence of KDM2A, conversely H3K36me2 was absent in the presence of KDM2A (Fig. 8c). Furthermore, H3K36me2 exhibited a distinctive spatial localization in the nuclei of KDM2A depleted cells as revealed by immunofluorescence analysis (Fig. 8d, e). All together, these data suggested that differences in H3K36me2 expression could be only determined in the CpG chromatin fragments. Strikingly, the observed re-localization of H3K36me2 in KDM2A-depleted cells was confirmed by ChIP-sequencing. Indeed, 73.39% of H3K36me2 DNA binding peaks were localized within gene promoters ( < 1 kb) and TSS (transcription start site) in control Hepa1-6cells, in comparison 50.29% of H3K36me2 DNA binding peaks were localized within gene promoters and TSS in KDM2A depleted cells (Fig. 8f–i and Suppl. Fig. 4). It is known that H3K36me2 bound CTCF motif, CTCF gene encodes the CCCTC-binding factor that plays a crucial role in regulating the spatial structure of chromatin and/or in defining the boundaries between active chromatin and heterochromatin. Homer analysis showed a loss of H3K36me2-CTCF gene binding in KDM2A-depleted Hepa1-6. Furthermore, GO and KEGG enrichment analysis of differentially expressed peak-associated genes showed that among the downregulated transcription factors genes, in KDM2A-depleted cells, there were linked to cellular metabolic processes, chemokine signaling pathways and T helper cell differentiation (Suppl. Fig. 5).

## Discussion

We identified KDM2A as a chromatin modifier that regulates key aspects of liver cancer behavior and its interaction with the immune microenvironment. KDM2A depletion induces DNA damage, disrupts mitochondrial function, and activates immune responses, that are multiple features of the cellular senescence program. Given the dynamic distribution of KDM2A at the CpG islands, this axis may act as a regulatory module controlling transcription in a tissue-specific manner [21].

Given the established link between epigenetic regulation of DDR [7–11] and immune responses[15–18], we reanalyzed our RNA-seq data with a focus on chromatin remodeling factors revealing an inverse correlation between immune activation and KDM2A.

As the in vitro findings suggested a role for KDM2A in repressing DNA damage-induced immune responses, we performed an IHC analysis of human HCC specimens. KDM2A expression significantly increased with tumor grade, correlating with the enrichment of CD163⁺ M2-like macrophages within the tumor core and a reduced presence of CD80⁺ M1-like macrophages/APCs, which are typically associated with anti-tumor immunity [37, 38]. Additionally, high-grade tumors exhibited a higher CD4⁺/CD8⁺ T cell ratio and a striking exclusion of CD8⁺ T cells from the tumor core, with their accumulation restricted to peritumoral regions. These features indicate an immune-excluded phenotype, linked to poor prognosis and immunotherapy resistance in solid tumors [39–41]. The correlation of KDM2A expression and HCC grading was further validated in an independent UK cohort, where high KDM2A levels were localized predominantly within poorly differentiated tumor areas, but not in the adjacent cirrhotic liver. TCGA data confirmed frequent KDM2A overexpression across cancers, including HCC, without common mutations or copy number changes, suggesting transcriptional regulation. In HCC patients, high KDM2A expression correlated with immune infiltrates enriched in macrophages, neutrophils, and regulatory T cells, all of which contribute to immune evasion. Furthermore, KDM2A levels positively correlated with CSF1 and CCL20 expression, which are cytokines critical for macrophage recruitment [42] and Treg trafficking [43], respectively suggesting a mechanistic role for KDM2A in shaping a pro-tumor immune landscape. Because KDM2A expression closely paralleled tumor grade, our patient cohort does not allow us to conclusively establish whether KDM2A is associated with immune infiltration independently of differentiation status. For this reason, the human data should be interpreted as showing that KDM2A-high poorly differentiated tumors display an immune-excluded phenotype, while the functional contribution of KDM2A to immune regulation is supported by the experimental KDM2A-depletion models. Taken together, these findings implicate KDM2A as a central epigenetic regulator of immune exclusion in HCC, promoting tumor progression by fostering an immunologically permissive niche.

We uncovered a novel epigenetic role of KDM2A in HCC, showing that its downregulation redistributes H3K36me2 at promoters of genes in metabolism, chemokine signaling, and T cell recruitment, thereby reorganizing chromatin and driving tumor progression and immune evasion.

We then explored the cause of DNA damage in KDM2A-depleted cells. Based on GO and KEGG enrichment analyses, a plausible explanation involved cellular metabolism, particularly mitochondrial function, which relies on the coordinated activity of both nuclear and mitochondrial genomes. In addition, mitochondrial dysfunction promotes DNA damage under conditions of increased apoptotic priming [44]. Interestingly, Merkwirth and colleagues demonstrated that conserved histone lysine demethylases were able to promote longevity and coordinate transcriptional outputs in response to mitochondrial dysfunction [45]. In other words, they revealed an epigenetic mode for the regulation of stress signaling that promotes cell life by overcoming mitochondrial defects. In line with these previous reports, our data point to mitochondrial dysfunction, supported by reduced membrane potential and altered oxidative phosphorylation. Our findings showed that KDM2A controls redox homeostasis by regulating folate-derived NADPH, a key antioxidant produced via one-carbon metabolism [34]. KDM2A contributes to the maintenance of the cellular antioxidant reservoir, primarily by influencing folate-derived NADPH production, therefore protecting the cells from damage and genomic instability caused by dysfunctional mitochondria.

As previously mentioned, mounting evidence links DNA damage to anti-tumor immunity [12, 46], and genotoxic therapies (e.g., platinum-based drugs) are often combined with anti-PD-1/PD-L1 molecules [47]. Notably, PD-L1 expression is known to increase in response to DNA double-strand breaks (DSBs) [14]. Our study adds that, in HCC, KDM2A regulates endogenous DNA damage and PD-L1 expression through an epigenetic mechanism. This supports the rationale for combining KDM2A-targeted therapies with immune checkpoint inhibitors to enhance therapeutic efficacy with potentially lower toxicity. As drug discovery efforts for chromatin modifiers advance, a detailed understanding of KDM2A’s function becomes increasingly important, mostly for cancers for which highly effective treatments are not yet available [48].

We further explored how DNA damage leads to immune activation. Although the DNA sensor cGAS was not expressed in our models, IFI16, a nuclear DNA sensor, was upregulated and localized to the nucleus after KDM2A depletion. IFI16 belongs to the PYHIN family, binds non-sequence-specific DNA, and activates STING-mediated immune signaling [49]. We propose that chromatin relaxation caused by KDM2A depletion may expose DNA stretches that activate IFI16, triggering downstream immune responses. Under immune pressure, cancer cells may silence this pathway through chromatin remodeling to avoid detection.

Immune checkpoint inhibitors targeting PD-(L)1 have shown promise in liver cancer, but responses are limited by immune evasion, including poor CD8⁺ T cell infiltration [50]. Our genetic models demonstrate that KDM2A overexpression promotes an immunosuppressive environment, partly by recruiting or retaining macrophages, which are key mediators of T cell suppression. In particular, macrophages may restrict intratumoral CD8⁺ T cell localization or indirectly suppress them via Treg cell recruitment [37, 51–53].

Although we did not directly map KDM2A genomic binding, our combined H3K36me2 and transcriptomic analyses suggest that KDM2A regulates key gene networks in HCC through promoter-associated chromatin remodeling. In particular, candidate downstream targets include immune modulators such as CSF1 and CCL20, antigen presentation and checkpoint molecules like PD-L1 and MHC class I genes, as well as metabolic regulators involved in one-carbon metabolism (e.g., SHMT1/2, MTHFD1). The induction of IFI16 and immune-related pathways upon KDM2A depletion further supports a role in restraining DNA damage–driven immune activation. Collectively, these findings raise the possibility that KDM2A functions as a central epigenetic hub integrating metabolic fitness, genome stability, and immune evasion.

Taken together, our findings can be framed within a broader senescence-associated program, integrating each of the molecular mechanisms described above. The dynamic interaction between KDM2A and CpG islands suggests the existence of a chromatin-based regulatory module capable of encoding tissue-specific transcriptional outputs, a property consistent with the epigenetic reprogramming observed during cellular senescence [54, 55]. KDM2A depletion induces chromatin remodeling, including redistribution of H3K36me2 and loss of heterochromatin integrity, a hallmark of senescent cells that has been directly linked to DNA damage accumulation and genomic instability [6, 56, 57]. In this context, the increased DNA damage observed upon KDM2A loss, particularly the persistence of double-strand breaks, represents a well-established trigger of senescence programs [11, 58]. Mechanistically, this is further supported by mitochondrial dysfunction and altered cellular metabolism, as impaired coordination between nuclear and mitochondrial genomes and defective oxidative phosphorylation are known drivers of senescence-associated stress responses. Notably, KDM2A regulation of folate-mediated one-carbon metabolism and NADPH production highlights its role in maintaining redox balance, whose disruption promotes oxidative stress and reinforces senescence induction [34]. Consistent with the link between DNA damage and immune activation, the upregulation of PD-L1 and activation of innate immune sensors such as IFI16 further connect KDM2A depletion to senescence-associated inflammatory signaling. IFI16-mediated activation of STING has been implicated in senescence and innate immune responses, while its frequent loss in cancer supports its role as a barrier to tumor progression [46, 49, 59–61]. Moreover, the immune landscape associated with high KDM2A expression, including macrophage recruitment, Treg enrichment, and CD8⁺ T cell exclusion, resembles the effects of the senescence-associated secretory phenotype (SASP), which can paradoxically promote tumor progression by shaping an immunosuppressive microenvironment [62]. Altogether, these observations position KDM2A as a key epigenetic regulator at the intersection of chromatin organization, metabolic control, DNA damage response, and immune signaling, all converging on the establishment and modulation of senescence-associated programs in HCC.

The findings might provide a rational to position KDM2A as a promising epigenetic target in HCC therapy, as drug discovery for chromatin modifiers advances elucidating the biological functions of epigenetic component like KDM2A becomes increasingly critical for designing rational, combination-based cancer treatments.

## Supplementary information

Supplementary figures and immunoblotting raw data

Homer analysis

RNAseq Hepa1-6 data

RNAseq Huh7 data

## Data Availability

All relevant data related to this study are included within the article or in the supplementary materials. Further data will be provided upon reasonable request to the corresponding authors.
