# Supplementary material for: Kdm2a histone demethylase ablation restores anti-tumor immunogenicity in liver cancer
Source: Br J Cancer. 2026 Aug 19;135(10):1587–602. doi: 10.1038/s41416-026-03566-z (PMC13740323; doi:10.1038/s41416-026-03566-z)
Supplement: Supplementary file 1 [file 41416_2026_3566_MOESM2_ESM.pdf]

Homer *de novo* Motif Results (WT_H3K36me2/)

Known Motif Enrichment Results
Gene Ontology Enrichment Results
If Homer is having trouble matching a motif to a known motif, try copy/pasting the matrix file into STAMP
More information on motif finding results: HOMER | Description of Results | Tips
Total target sequences = 4742
Total background sequences = 810
* - possible false positive

Rank	Motif	P-value	log P-pvalue	% of Targets	% of Background	STD(Bg STD)	Best Match/Details	Motif File
1	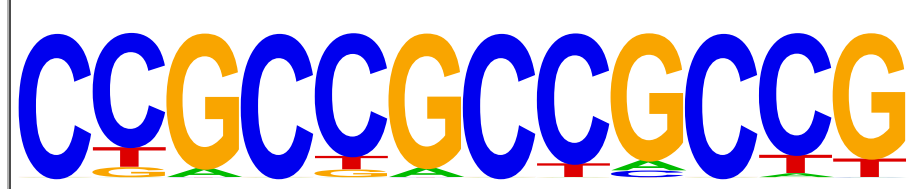	1e-3707	-8.536e+03	36.65%	0.19%	111.7bp (15.3bp)	CRF4/MA0976.2/Jaspar(0.906) More Information | Similar Motifs Found	motif file (matrix)
2	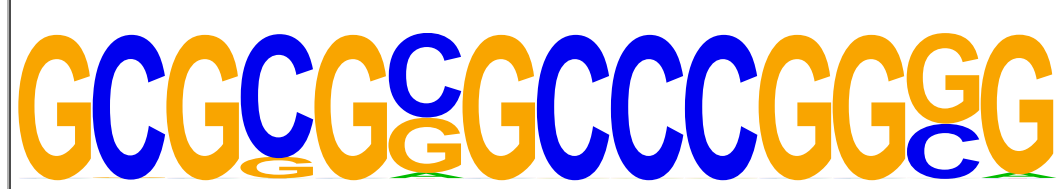	1e-3046	-7.015e+03	31.34%	0.10%	127.6bp (8.9bp)	SeqBias: CG-repeat(0.724) More Information | Similar Motifs Found	motif file (matrix)
3	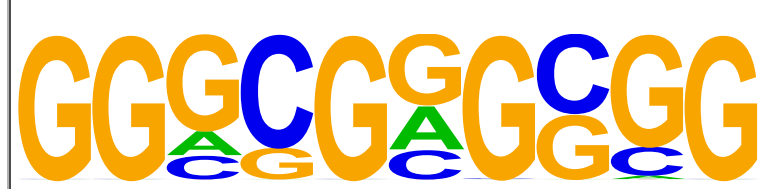	1e-2400	-5.527e+03	33.91%	0.51%	124.7bp (70.0bp)	SeqBias: polyC-repeat(0.818) More Information | Similar Motifs Found	motif file (matrix)
4	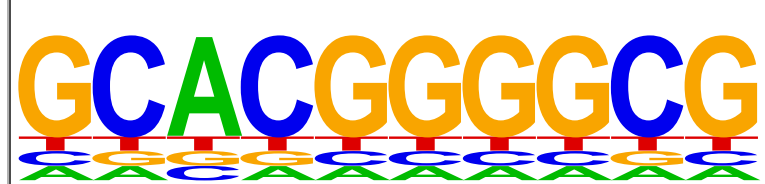	1e-2233	-5.142e+03	24.46%	0.23%	126.5bp (97.7bp)	CTCFL/MA1102.2/Jaspar(0.776) More Information | Similar Motifs Found	motif file (matrix)
5	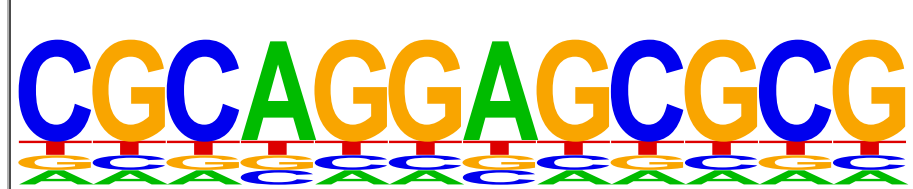	1e-2157	-4.967e+03	31.34%	0.59%	133.8bp (33.8bp)	MYB88(MYB)/col-MYB88-DAP-Seq(GSE60143)/Homer(0.652) More Information | Similar Motifs Found	motif file (matrix)
6	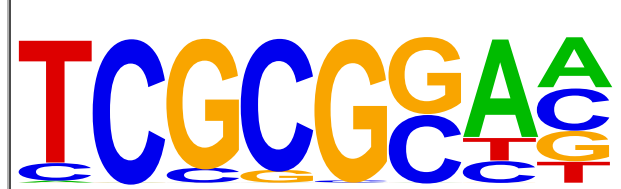	1e-2122	-4.887e+03	42.62%	1.85%	139.7bp (195.0bp)	RSC3/MA0374.1/Jaspar(0.828) More Information | Similar Motifs Found	motif file (matrix)
7	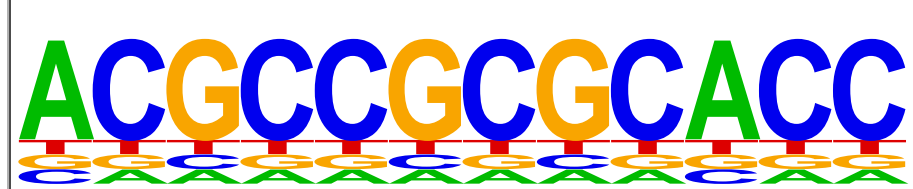	1e-2091	-4.816e+03	26.49%	0.30%	130.7bp (78.8bp)	ZBTB14/MA1650.1/Jaspar(0.838) More Information | Similar Motifs Found	motif file (matrix)
8	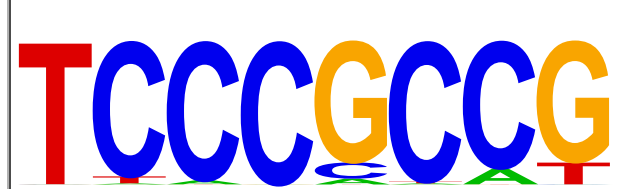	1e-2076	-4.782e+03	23.09%	0.20%	134.3bp (41.7bp)	UGA3/MA0410.1/Jaspar(0.936) More Information | Similar Motifs Found	motif file (matrix)
9	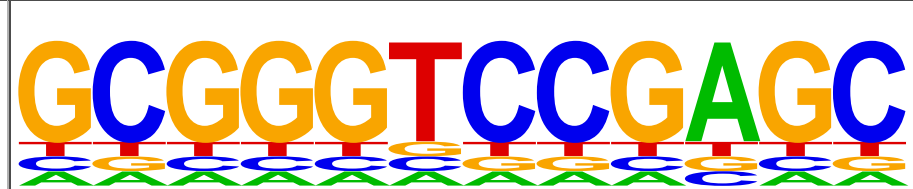	1e-2012	-4.634e+03	27.96%	0.41%	128.8bp (123.7bp)	TBS1/MA0404.1/Jaspar(0.723) More Information | Similar Motifs Found	motif file (matrix)
10	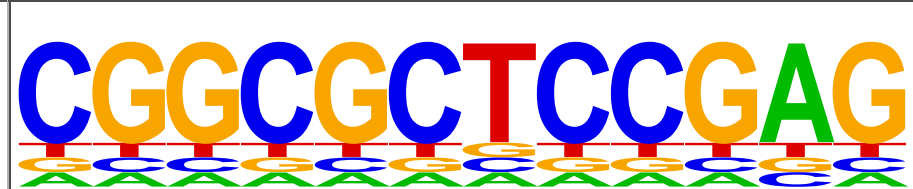	1e-1980	-4.559e+03	27.63%	0.44%	133.7bp (85.4bp)	POL013.1_MED-1/Jaspar(0.687) More Information | Similar Motifs Found	motif file (matrix)
11	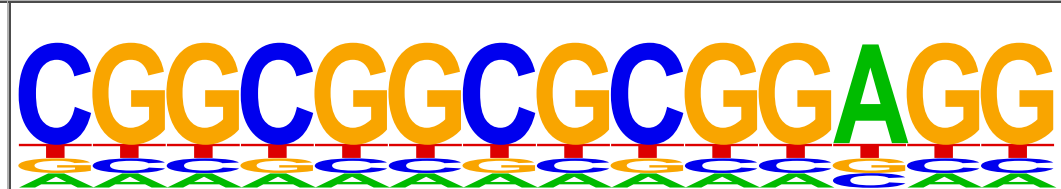	1e-1906	-4.390e+03	21.57%	0.01%	131.1bp (1.8bp)	RAP2-6/MA1052.1/Jaspar(0.744) More Information | Similar Motifs Found	motif file (matrix)
12	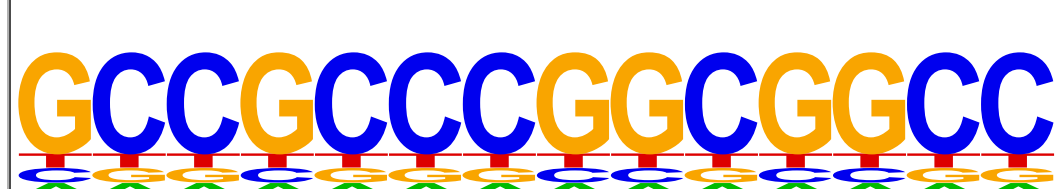	1e-1729	-3.982e+03	19.97%	0.11%	115.1bp (44.0bp)	STP1/Literature(Harbison)/Yeast(0.666) More Information | Similar Motifs Found	motif file (matrix)
13	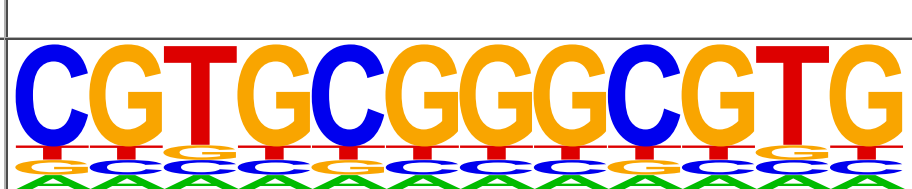	1e-1621	-3.734e+03	23.79%	0.39%	126.5bp (64.7bp)	KLF11/MA1512.1/Jaspar(0.735) More Information |	motif file (matrix)

							Similar Motifs Found	
14	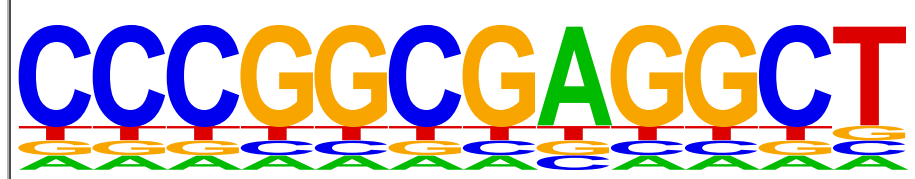	1e-1613	-3.716e+03	23.70%	0.42%	126.5bp (32.2bp)	IME1(MacIsaac)/ Yeast(0.664) More Information | Similar Motifs Found	motif file (matrix)
15	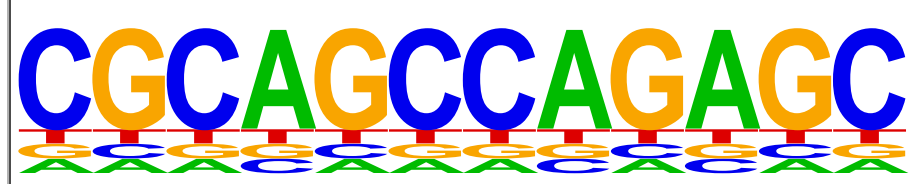	1e-1363	-3.139e+03	24.76%	0.80%	128.6bp (42.9bp)	CRF4(AP2EREBP)/ colamp-CRF4-DAP-Seq(GSE60143)/ Homer(0.645) More Information | Similar Motifs Found	motif file (matrix)
16	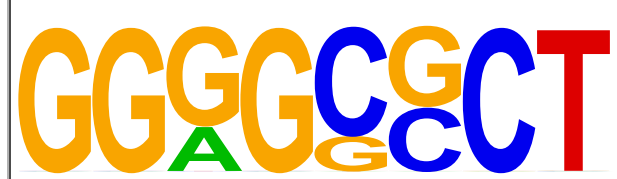	1e-1343	-3.094e+03	28.13%	1.25%	139.3bp (77.0bp)	PLAGL2/MA1548.1/ Jaspar(0.776) More Information | Similar Motifs Found	motif file (matrix)
17	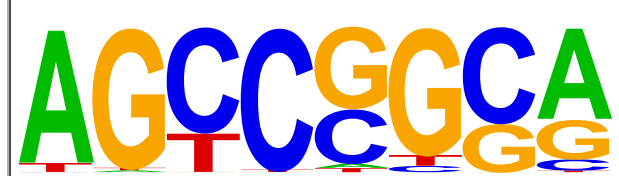	1e-1324	-3.050e+03	21.91%	0.53%	131.3bp (51.3bp)	ERF6/MA1006.1/ Jaspar(0.803) More Information | Similar Motifs Found	motif file (matrix)
18	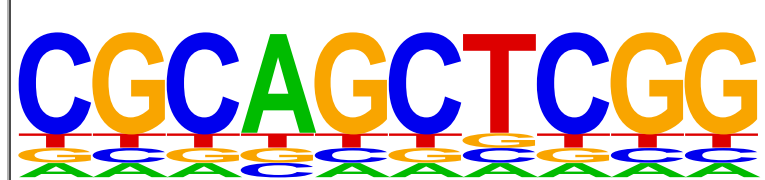	1e-1298	-2.989e+03	21.59%	0.60%	132.0bp (151.0bp)	NHLH1/MA0048.2/ Jaspar(0.679) More Information | Similar Motifs Found	motif file (matrix)
19	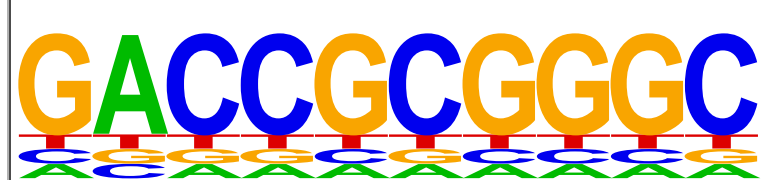	1e-1270	-2.926e+03	22.48%	0.65%	124.5bp (94.7bp)	Adf1/dmmpmm(Pollard)/ fly(0.731) More Information | Similar Motifs Found	motif file (matrix)
20	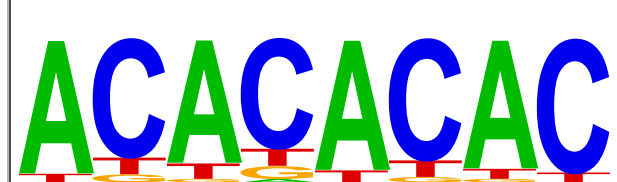	1e-210	-4.845e+02	21.83%	7.52%	125.9bp (156.8bp)	ARET(RRM)/ Drosophila_melanogaster-RNCMPT00270-PBM/ HughesRNA(0.891) More Information | Similar Motifs Found	motif file (matrix)
21	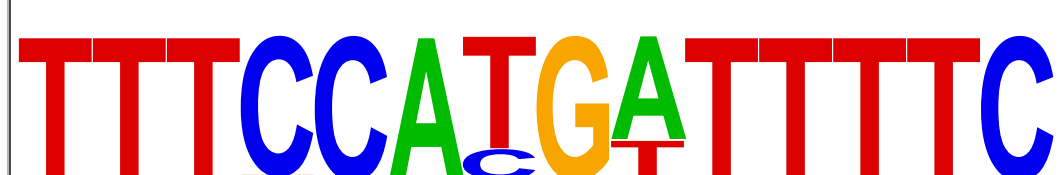	1e-152	-3.516e+02	3.18%	0.07%	136.3bp (30.7bp)	HSF1/HSF1_H2O2Lo/11-HSF1(Harbison)/ Yeast(0.686) More Information | Similar Motifs Found	motif file (matrix)
22	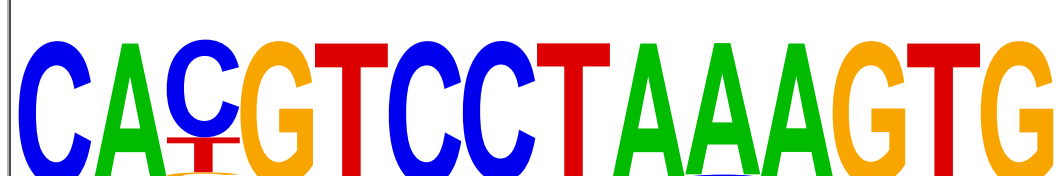	1e-119	-2.748e+02	2.68%	0.24%	134.2bp (43.9bp)	tin/ dmmpmm(SeSiMCMC)/ fly(0.646) More Information | Similar Motifs Found	motif file (matrix)
23	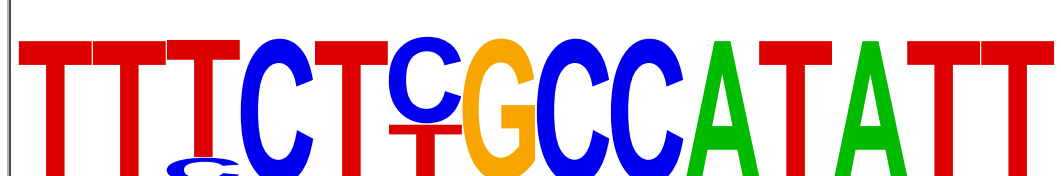	1e-95	-2.202e+02	2.30%	0.19%	135.8bp (0.0bp)	E2F8/MA0865.1/ Jaspar(0.678) More Information | Similar Motifs Found	motif file (matrix)

Homer Known Motif Enrichment Results (WT_H3K36me2)

Homer *de novo* Motif Results
Gene Ontology Enrichment Results
Known Motif Enrichment Results (txt file)
Total Target Sequences = 4743, Total Background Sequences = 811

Rank	Motif	Name	P-value	log P-pvalue	q-value (Benjamini)	# Target Sequences with Motif	% of Targets Sequences with Motif	# Background Sequences with Motif	% of Background Sequences with Motif	Motif File	SVG
1	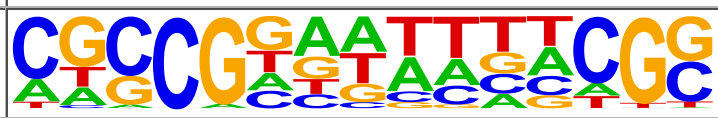	LOB(LOBAS2)/col-LOB-DAP-Seq(GSE60143)/Homer	1e-489	-1.127e+03	0.0000	1152.0	24.29%	36.1	4.45%	motif file (matrix)	svg
2	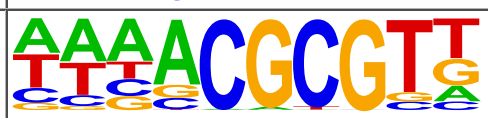	CAMTA1(CAMTA)/col-CAMTA1-DAP-Seq(GSE60143)/Homer	1e-381	-8.785e+02	0.0000	1031.0	21.74%	37.3	4.59%	motif file (matrix)	svg
3	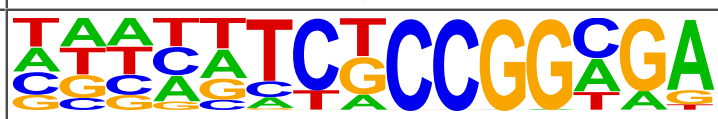	AT5G05550(Trihelix)/col-AT5G05550-DAP-Seq(GSE60143)/Homer	1e-307	-7.072e+02	0.0000	2509.0	52.91%	220.3	27.12%	motif file (matrix)	svg
4	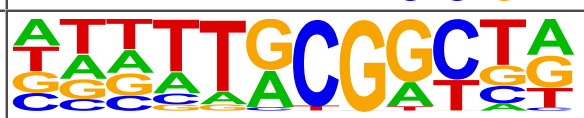	At5g08750(C3H)/col-At5g08750-DAP-Seq(GSE60143)/Homer	1e-281	-6.488e+02	0.0000	696.0	14.68%	22.5	2.77%	motif file (matrix)	svg
5	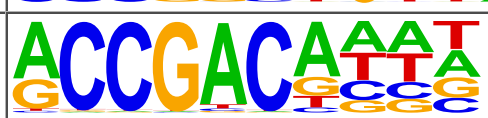	AT1G77200(AP2EREBP)/colamp-AT1G77200-DAP-Seq(GSE60143)/Homer	1e-245	-5.651e+02	0.0000	1177.0	24.82%	69.9	8.61%	motif file (matrix)	svg
6	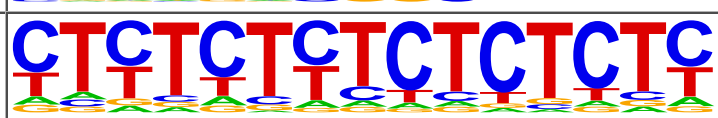	GAGA-repeat/SacCer-Promoters/Homer	1e-239	-5.518e+02	0.0000	2574.0	54.28%	253.0	31.15%	motif file (matrix)	svg
7	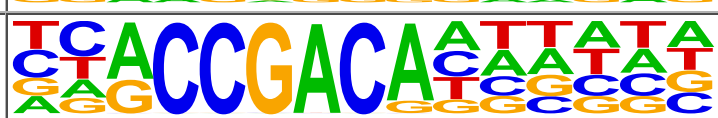	At2g44940(AP2EREBP)/colamp-At2g44940-DAP-Seq(GSE60143)/Homer	1e-222	-5.125e+02	0.0000	242.0	5.10%	2.3	0.28%	motif file (matrix)	svg
8	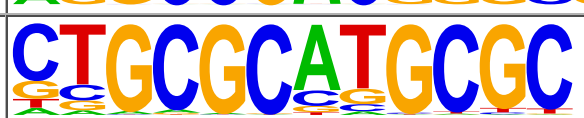	NRF1(NRF)/MCF7-NRF1-ChIP-Seq(Unpublished)/Homer	1e-220	-5.073e+02	0.0000	789.0	16.64%	36.6	4.50%	motif file (matrix)	svg
9	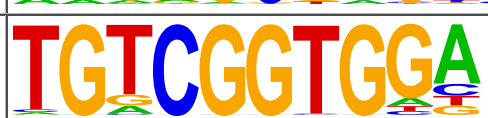	At1g77640(AP2EREBP)/col-At1g77640-DAP-Seq(GSE60143)/Homer	1e-212	-4.898e+02	0.0000	404.0	8.52%	9.2	1.14%	motif file (matrix)	svg
10	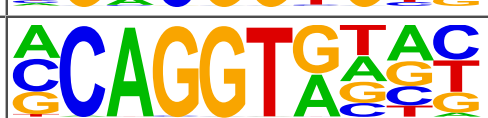	ZEB1(Zf)/PDAC-ZEB1-ChIP-Seq(GSE64557)/Homer	1e-211	-4.876e+02	0.0000	1683.0	35.49%	136.4	16.79%	motif file (matrix)	svg
11	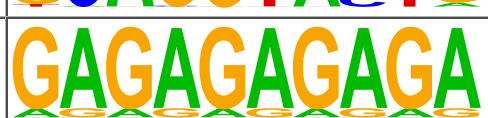	SeqBias: GA-repeat	1e-200	-4.627e+02	0.0000	4087.0	86.19%	544.6	67.05%	motif file (matrix)	svg
12	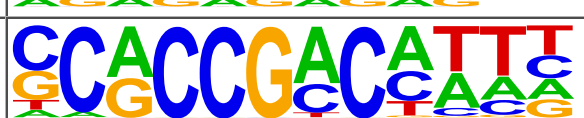	At5g65130(AP2EREBP)/colamp-At5g65130-DAP-Seq(GSE60143)/Homer	1e-189	-4.374e+02	0.0000	888.0	18.73%	50.9	6.26%	motif file (matrix)	svg
13	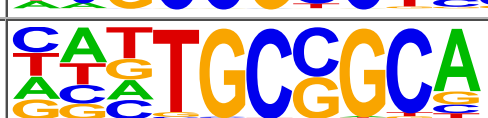	Zfp57(Zf)/H1-ZFP57.HA-ChIP-Seq(GSE115387)/Homer	1e-183	-4.223e+02	0.0000	1491.0	31.44%	120.8	14.87%	motif file (matrix)	svg
14	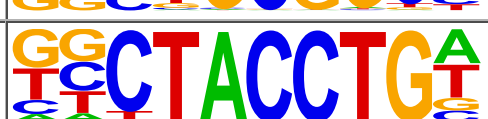	Zelda(Zf)/Embryo-zld-ChIP-Seq(GSE65441)/Homer	1e-164	-3.785e+02	0.0000	456.0	9.62%	16.6	2.05%	motif file (matrix)	svg
15	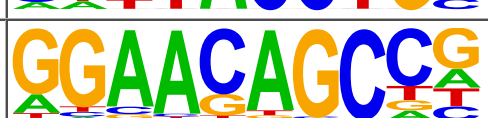	ZNF341(Zf)/EBV-ZNF341-ChIP-Seq(GSE113194)/Homer	1e-155	-3.591e+02	0.0000	794.0	16.74%	47.1	5.80%	motif file (matrix)	svg
16	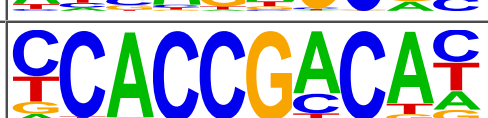	DREB26(AP2EREBP)/col-DREB26-DAP-Seq(GSE60143)/Homer	1e-151	-3.486e+02	0.0000	725.0	15.29%	41.9	5.16%	motif file (matrix)	svg
17	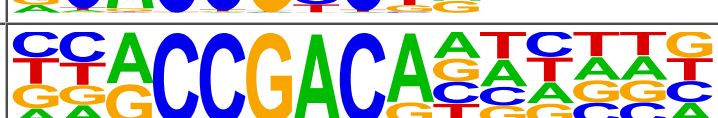	AT3G60490(AP2EREBP)/colamp-AT3G60490-DAP-Seq(GSE60143)/Homer	1e-150	-3.467e+02	0.0000	423.0	8.92%	15.2	1.87%	motif file (matrix)	svg
18	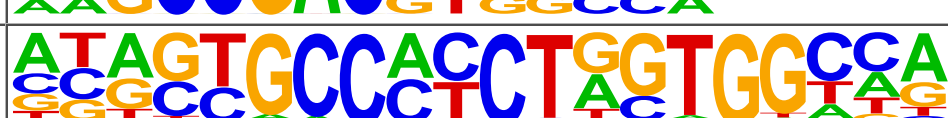	CTCF(Zf)/CD4+-CTCF-ChIP-Seq(Barski_et_al.)/Homer	1e-149	-3.444e+02	0.0000	185.0	3.90%	2.2	0.27%	motif file (matrix)	svg
19	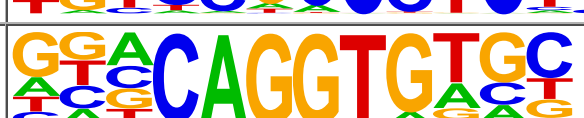	ZEB2(Zf)/SNU398-ZEB2-ChIP-Seq(GSE103048)/Homer	1e-146	-3.380e+02	0.0000	1050.0	22.14%	77.6	9.56%	motif file (matrix)	svg
20	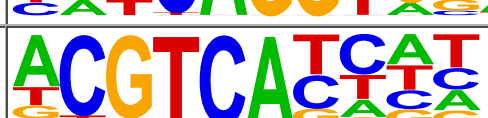	TGA2(bZIP)/colamp-TGA2-DAP-Seq(GSE60143)/Homer	1e-144	-3.325e+02	0.0000	454.0	9.57%	18.8	2.31%	motif file (matrix)	svg
21	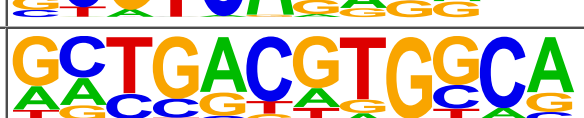	O2(bZIP)/Corn-O2-ChIP-Seq(GSE63991)/Homer	1e-141	-3.249e+02	0.0000	178.0	3.75%	2.2	0.28%	motif file (matrix)	svg
22	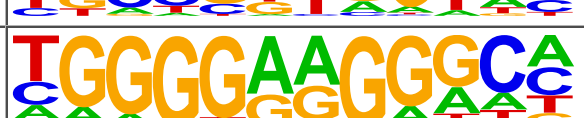	ZNF467(Zf)/HEK293-ZNF467.GFP-ChIP-Seq(GSE58341)/Homer	1e-134	-3.099e+02	0.0000	1474.0	31.08%	135.0	16.62%	motif file (matrix)	svg
23	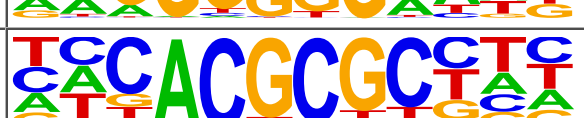	FHY3(FAR1)/Arabidopsis-FHY3-ChIP-Seq(GSE30711)/Homer	1e-132	-3.054e+02	0.0000	1198.0	25.26%	99.2	12.21%	motif file (matrix)	svg
24	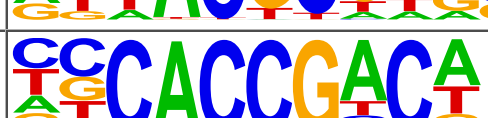	AT1G44830(AP2EREBP)/col-AT1G44830-DAP-Seq(GSE60143)/Homer	1e-131	-3.028e+02	0.0000	716.0	15.10%	44.1	5.42%	motif file (matrix)	svg
25	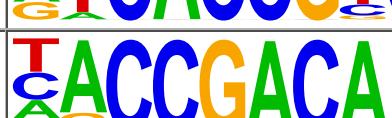	bHLH10(bHLH)/colamp-bHLH10-DAP-Seq(GSE60143)/Homer	1e-129	-2.984e+02	0.0000	393.0	8.29%	15.3	1.88%	motif file (matrix)	svg
26	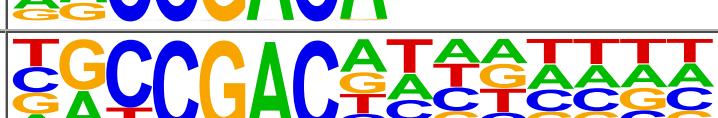	CBF4(AP2EREBP)/colamp-CBF4-DAP-Seq(GSE60143)/Homer	1e-125	-2.882e+02	0.0000	1032.0	21.76%	81.3	10.00%	motif file (matrix)	svg
27	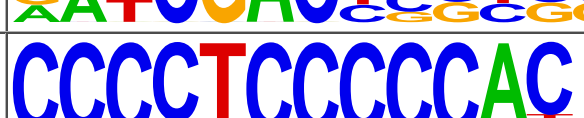	Zfp281(Zf)/ES-Zfp281-ChIP-Seq(GSE81042)/Homer	1e-122	-2.823e+02	0.0000	629.0	13.26%	37.3	4.59%	motif file (matrix)	svg
28	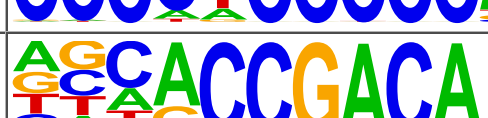	TINY(AP2EREBP)/col-TINY-DAP-Seq(GSE60143)/Homer	1e-120	-2.782e+02	0.0000	380.0	8.01%	15.2	1.87%	motif file (matrix)	svg
29	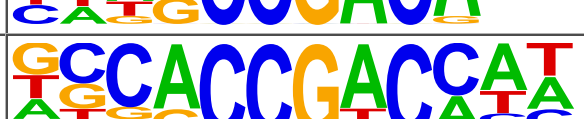	At4g28140(AP2EREBP)/colamp-At4g28140-DAP-Seq(GSE60143)/Homer	1e-118	-2.734e+02	0.0000	718.0	15.14%	47.3	5.82%	motif file (matrix)	svg
30	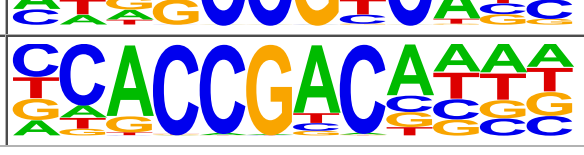	At1g22810(AP2EREBP)/colamp-At1g22810-DAP-Seq(GSE60143)/Homer	1e-118	-2.727e+02	0.0000	736.0	15.52%	50.0	6.16%	motif file (matrix)	svg

31	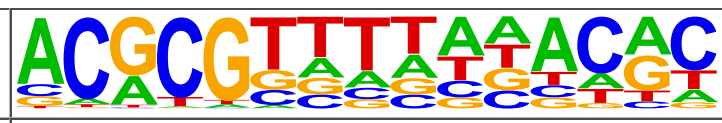	CAMTA5(CAMTA)/col-CAMTA5-DAP-Seq(GSE60143)/Homer	1e-111	-2.575e+02	0.0000	269.0	5.67%	8.2	1.01%	motif file (matrix)	svg
32	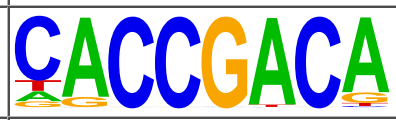	ERF38(AP2EREBP)/col-ERF38-DAP-Seq(GSE60143)/Homer	1e-108	-2.494e+02	0.0000	803.0	16.93%	59.6	7.33%	motif file (matrix)	svg
33	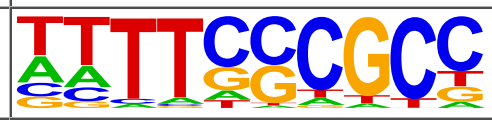	DEL2(E2FDP)/col-DEL2-DAP-Seq(GSE60143)/Homer	1e-107	-2.477e+02	0.0000	757.0	15.96%	54.2	6.67%	motif file (matrix)	svg
34	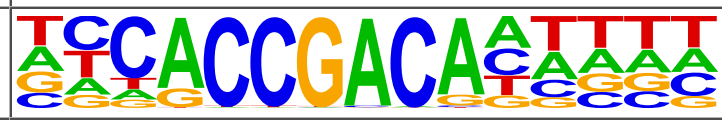	At4g32800(AP2EREBP)/colamp-At4g32800-DAP-Seq(GSE60143)/Homer	1e-107	-2.471e+02	0.0000	118.0	2.49%	1.7	0.21%	motif file (matrix)	svg
35	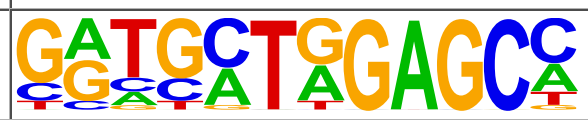	ZNF415(Zf)/HEK293-ZNF415.GFP-ChIP-Seq(GSE58341)/Homer	1e-107	-2.465e+02	0.0000	826.0	17.42%	62.2	7.66%	motif file (matrix)	svg
36	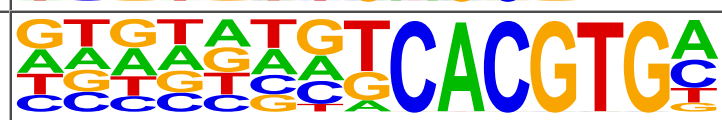	BIM1(bHLH)/colamp-BIM1-DAP-Seq(GSE60143)/Homer	1e-100	-2.321e+02	0.0000	113.0	2.38%	0.9	0.11%	motif file (matrix)	svg
37	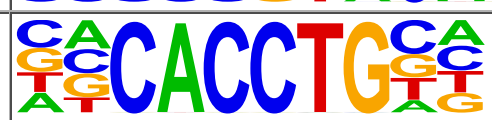	E2A(bHLH),near_PU.1/Bcell-PU.1-ChIP-Seq(GSE21512)/Homer	1e-99	-2.294e+02	0.0000	1428.0	30.11%	143.0	17.60%	motif file (matrix)	svg
38	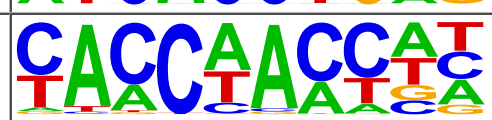	MYB83(MYB)/colamp-MYB83-DAP-Seq(GSE60143)/Homer	1e-98	-2.272e+02	0.0000	922.0	19.44%	76.7	9.45%	motif file (matrix)	svg
39	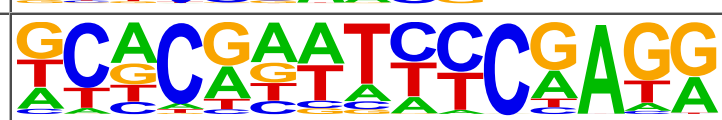	AIL7(AP2EREBP)/colamp-AIL7-DAP-Seq(GSE60143)/Homer	1e-98	-2.267e+02	0.0000	381.0	8.03%	18.4	2.26%	motif file (matrix)	svg
40	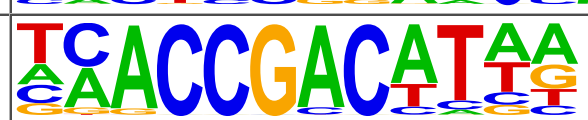	DREB2(AP2EREBP)/col-DREB2-DAP-Seq(GSE60143)/Homer	1e-98	-2.257e+02	0.0000	588.0	12.40%	38.3	4.72%	motif file (matrix)	svg
41	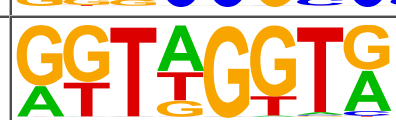	MYB92(MYB)/colamp-MYB92-DAP-Seq(GSE60143)/Homer	1e-91	-2.108e+02	0.0000	611.0	12.88%	42.7	5.25%	motif file (matrix)	svg
42	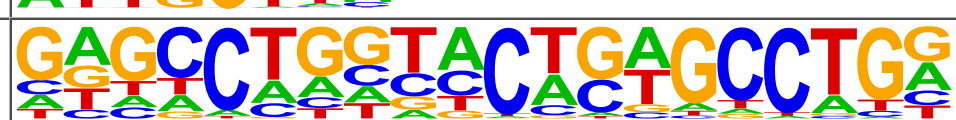	ZNF322(Zf)/HEK293-ZNF322.GFP-ChIP-Seq(GSE58341)/Homer	1e-91	-2.096e+02	0.0000	296.0	6.24%	12.1	1.49%	motif file (matrix)	svg
43	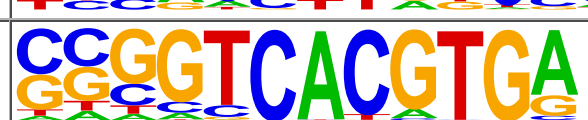	E-box(bHLH)/Promoter/Homer	1e-86	-1.997e+02	0.0000	130.0	2.74%	3.0	0.37%	motif file (matrix)	svg
44	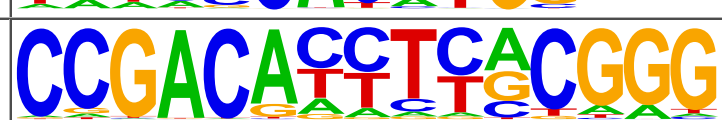	GEI-11(Myb?)/cElegans-L4-GEI11-ChIP-Seq(modEncode)/Homer	1e-86	-1.997e+02	0.0000	130.0	2.74%	2.3	0.28%	motif file (matrix)	svg
45	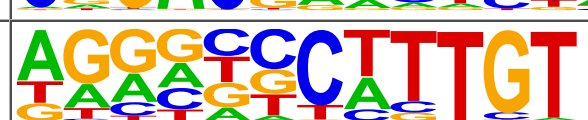	Sox9(HMG)/Limb-SOX9-ChIP-Seq(GSE73225)/Homer	1e-86	-1.982e+02	0.0000	465.0	9.81%	28.2	3.47%	motif file (matrix)	svg
46	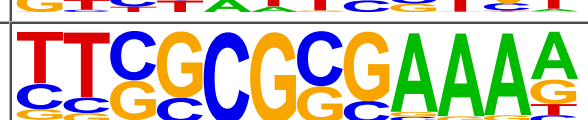	E2F(E2F)/Hela-CellCycle-Expression/Homer	1e-84	-1.944e+02	0.0000	169.0	3.56%	4.6	0.56%	motif file (matrix)	svg
47	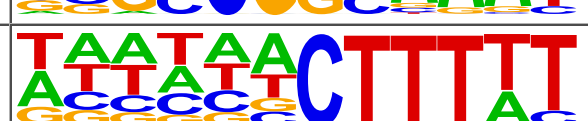	At5g62940(C2C2dof)/col-At5g62940-DAP-Seq(GSE60143)/Homer	1e-83	-1.922e+02	0.0000	1479.0	31.19%	157.8	19.43%	motif file (matrix)	svg
48	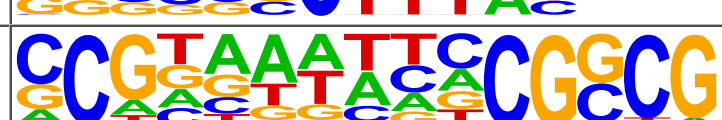	AS2(LOBAS2)/col-AS2-DAP-Seq(GSE60143)/Homer	1e-81	-1.872e+02	0.0000	513.0	10.82%	34.4	4.23%	motif file (matrix)	svg
49	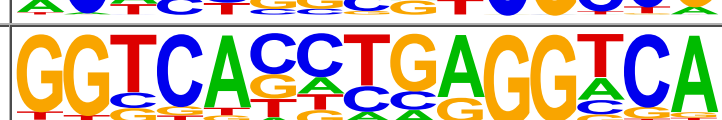	THRb(NR)/HepG2-THRb.Flag-ChIP-Seq(Encode)/Homer	1e-79	-1.829e+02	0.0000	644.0	13.58%	49.1	6.05%	motif file (matrix)	svg
50	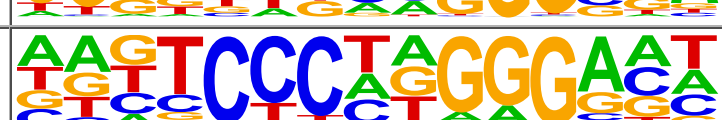	EBF2(EBF)/BrownAdipose-EBF2-ChIP-Seq(GSE97114)/Homer	1e-78	-1.819e+02	0.0000	802.0	16.91%	68.2	8.39%	motif file (matrix)	svg
51	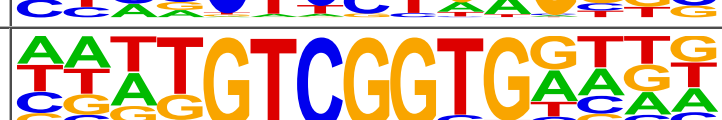	DEAR5(AP2EREBP)/col-DEAR5-DAP-Seq(GSE60143)/Homer	1e-76	-1.758e+02	0.0000	221.0	4.66%	8.3	1.03%	motif file (matrix)	svg
52	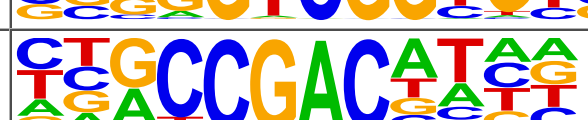	CBF3(AP2EREBP)/colamp-CBF3-DAP-Seq(GSE60143)/Homer	1e-73	-1.692e+02	0.0000	696.0	14.68%	57.7	7.11%	motif file (matrix)	svg
53	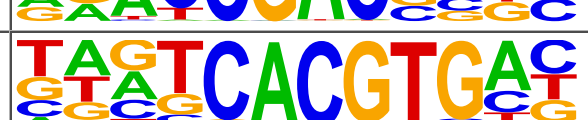	BIM3(bHLH)/col-BIM3-DAP-Seq(GSE60143)/Homer	1e-73	-1.687e+02	0.0000	117.0	2.47%	2.4	0.30%	motif file (matrix)	svg
54	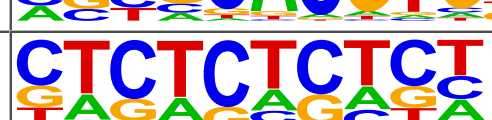	GAGA-repeat/Arabidopsis-Promoters/Homer	1e-72	-1.667e+02	0.0000	854.0	18.01%	77.3	9.52%	motif file (matrix)	svg
55	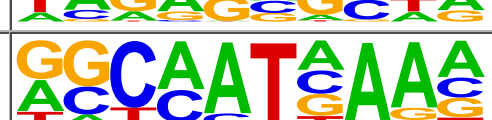	Hoxa9(Homeobox)/ChickenMSG-Hoxa9.Flag-ChIP-Seq(GSE86088)/Homer	1e-72	-1.664e+02	0.0000	1140.0	24.04%	115.7	14.24%	motif file (matrix)	svg
56	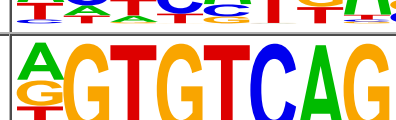	CELF2(RRM)/JSL1-CELF2-CLIP-Seq(GSE71264)/Homer	1e-72	-1.661e+02	0.0000	201.0	4.24%	7.8	0.97%	motif file (matrix)	svg
57	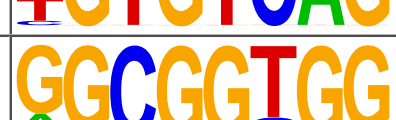	AT3G57600(AP2EREBP)/col-AT3G57600-DAP-Seq(GSE60143)/Homer	1e-70	-1.623e+02	0.0000	3284.0	69.25%	460.9	56.75%	motif file (matrix)	svg
58	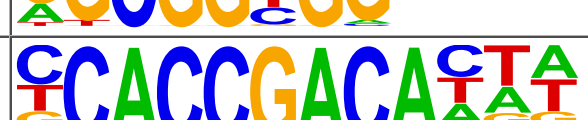	AT1G01250(AP2EREBP)/col-AT1G01250-DAP-Seq(GSE60143)/Homer	1e-68	-1.588e+02	0.0000	133.0	2.80%	3.2	0.39%	motif file (matrix)	svg
59	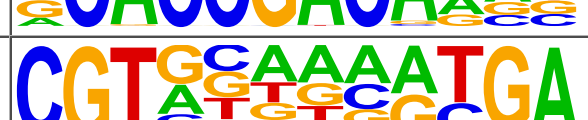	ABF1/SacCer-Promoters/Homer	1e-66	-1.542e+02	0.0000	365.0	7.70%	22.4	2.75%	motif file (matrix)	svg
60	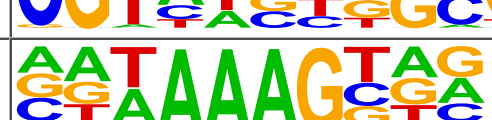	Adof1(C2C2dof)/col-Adof1-DAP-Seq(GSE60143)/Homer	1e-63	-1.467e+02	0.0000	1286.0	27.12%	140.5	17.30%	motif file (matrix)	svg
61	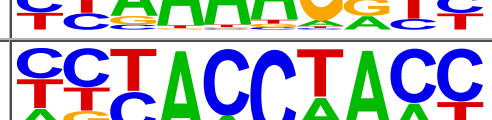	MYB61(MYB)/colamp-MYB61-DAP-Seq(GSE60143)/Homer	1e-62	-1.432e+02	0.0000	852.0	17.97%	82.0	10.09%	motif file (matrix)	svg
62	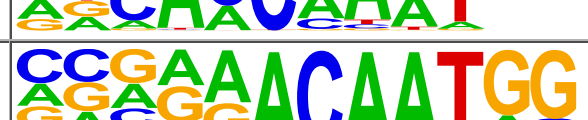	Sox7(HMG)/ESC-Sox7-ChIP-Seq(GSE133899)/Homer	1e-61	-1.418e+02	0.0000	236.0	4.98%	11.6	1.43%	motif file (matrix)	svg
63	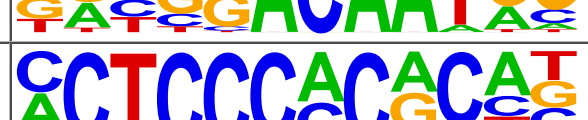	WT1(Zf)/Kidney-WT1-ChIP-Seq(GSE90016)/Homer	1e-60	-1.390e+02	0.0000	1437.0	30.30%	164.5	20.26%	motif file (matrix)	svg
64	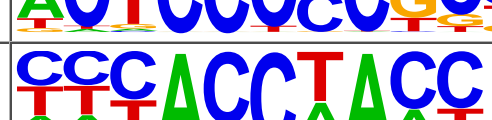	MYB58(MYB)/colamp-MYB58-DAP-Seq(GSE60143)/Homer	1e-59	-1.362e+02	0.0000	469.0	9.89%	36.0	4.43%	motif file (matrix)	svg

65	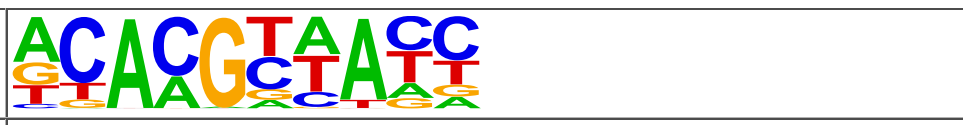	ANAC046(NAC)/colamp-ANAC046-DAP-Seq(GSE60143)/Homer	1e-58	-1.357e+02	0.0000	901.0	19.00%	90.0	11.08%	motif file (matrix)	svg
66	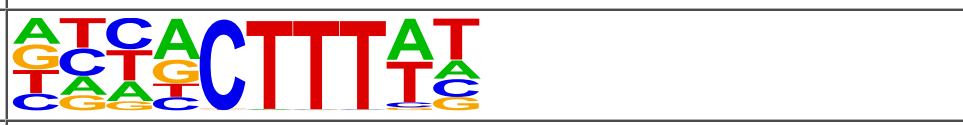	OBP1(C2C2dof)/col-OBP1-DAP-Seq(GSE60143)/Homer	1e-58	-1.352e+02	0.0000	1231.0	25.96%	135.7	16.70%	motif file (matrix)	svg
67	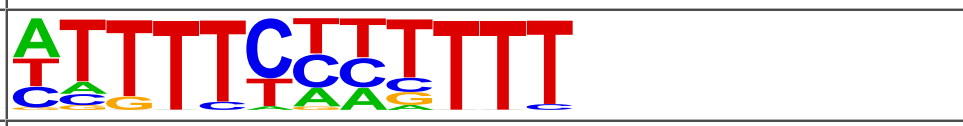	RLR1?/SacCer-Promoters/Homer	1e-57	-1.333e+02	0.0000	439.0	9.26%	32.3	3.98%	motif file (matrix)	svg
68	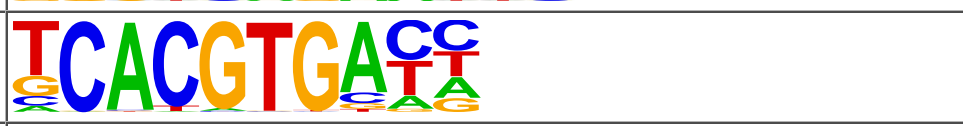	Cbf1(bHLH)/Yeast-Cbf1-ChIP-Seq(GSE29506)/Homer	1e-57	-1.329e+02	0.0000	263.0	5.55%	14.2	1.75%	motif file (matrix)	svg
69	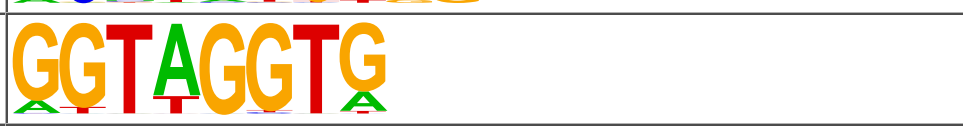	MYB51(MYB)/col-MYB51-DAP-Seq(GSE60143)/Homer	1e-57	-1.324e+02	0.0000	447.0	9.43%	33.3	4.10%	motif file (matrix)	svg
70	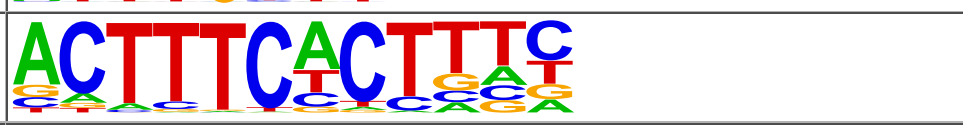	PRDM1(Zf)/Hela-PRDM1-ChIP-Seq(GSE31477)/Homer	1e-56	-1.305e+02	0.0000	381.0	8.03%	26.9	3.32%	motif file (matrix)	svg
71	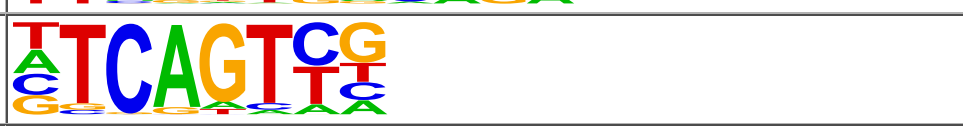	Initiator/Drosophila-Promoters/Homer	1e-54	-1.259e+02	0.0000	1067.0	22.50%	114.1	14.05%	motif file (matrix)	svg
72	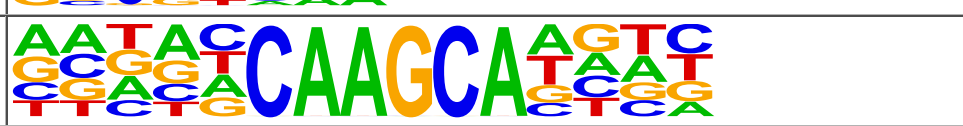	AT5G25475(ABI3VP1)/col-AT5G25475-DAP-Seq(GSE60143)/Homer	1e-53	-1.235e+02	0.0000	597.0	12.59%	52.1	6.41%	motif file (matrix)	svg
73	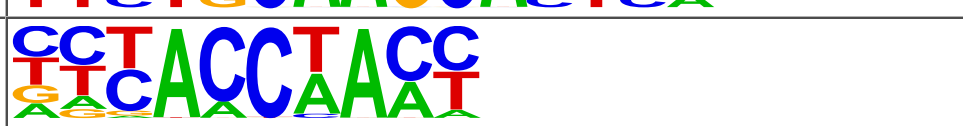	AT4G26030(C2H2)/col-AT4G26030-DAP-Seq(GSE60143)/Homer	1e-53	-1.228e+02	0.0000	471.0	9.93%	37.5	4.61%	motif file (matrix)	svg
74	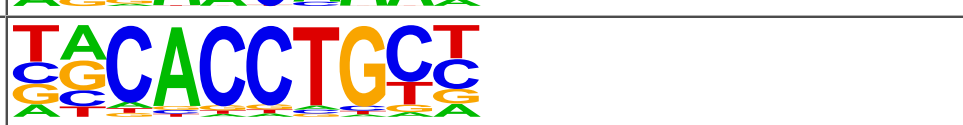	Snail1(Zf)/LS174T-SNAIL1.HA-ChIP-Seq(GSE127183)/Homer	1e-53	-1.228e+02	0.0000	932.0	19.65%	96.9	11.93%	motif file (matrix)	svg
75	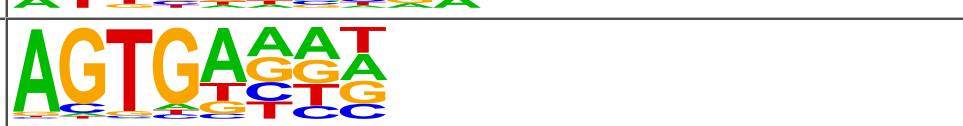	At5g04390(C2H2)/col200-At5g04390-DAP-Seq(GSE60143)/Homer	1e-53	-1.226e+02	0.0000	2621.0	55.27%	358.2	44.11%	motif file (matrix)	svg
76	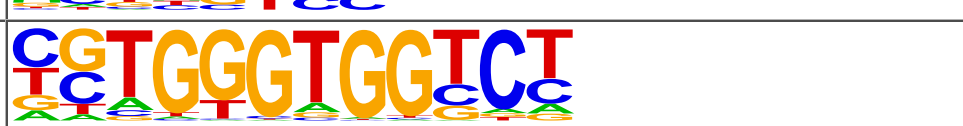	Gli2(Zf)/GM2-Gli2-ChIP-Chip(GSE112702)/Homer	1e-52	-1.207e+02	0.0000	242.0	5.10%	13.2	1.63%	motif file (matrix)	svg
77	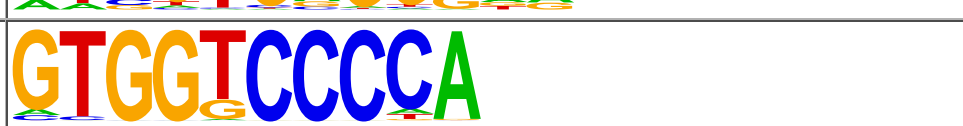	TCP17(TCP)/col-TCP17-DAP-Seq(GSE60143)/Homer	1e-52	-1.207e+02	0.0000	73.0	1.54%	1.8	0.22%	motif file (matrix)	svg
78	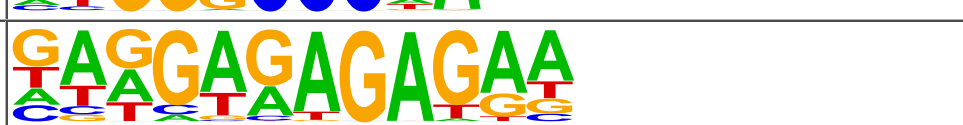	BPC1(BBRBPC)/colamp-BPC1-DAP-Seq(GSE60143)/Homer	1e-51	-1.183e+02	0.0000	457.0	9.64%	37.0	4.56%	motif file (matrix)	svg
79	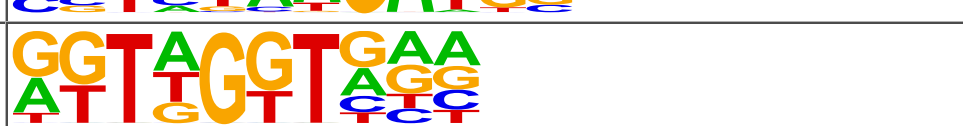	MYB107(MYB)/col-MYB107-DAP-Seq(GSE60143)/Homer	1e-50	-1.155e+02	0.0000	898.0	18.94%	93.6	11.52%	motif file (matrix)	svg
80	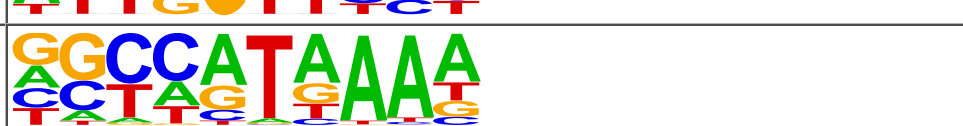	Hoxd11(Homeobox)/ChickenMSG-Hoxd11.Flag-ChIP-Seq(GSE86088)/Homer	1e-49	-1.147e+02	0.0000	1040.0	21.93%	113.3	13.95%	motif file (matrix)	svg
81	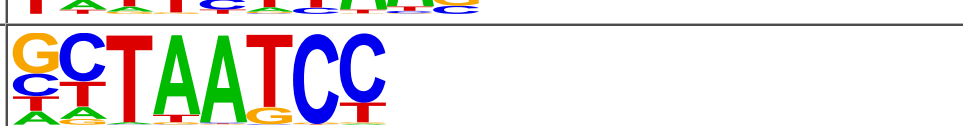	CRX(Homeobox)/Retina-Crx-ChIP-Seq(GSE20012)/Homer	1e-49	-1.144e+02	0.0000	997.0	21.02%	107.7	13.26%	motif file (matrix)	svg
82	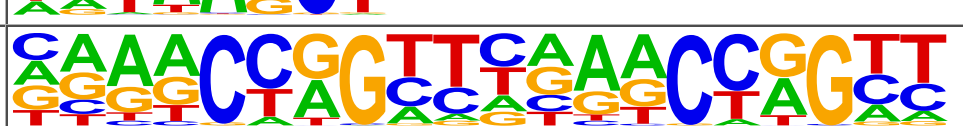	Tcfcp2l1(CP2)/mES-Tcfcp2l1-ChIP-Seq(GSE11431)/Homer	1e-49	-1.131e+02	0.0000	140.0	2.95%	5.1	0.62%	motif file (matrix)	svg
83	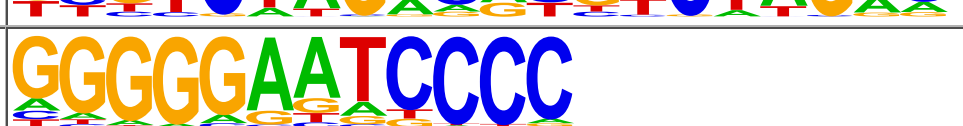	NFkB-p50,p52(RHD)/Monocyte-p50-ChIP-Chip(Schreiber_et_al.)/Homer	1e-48	-1.119e+02	0.0000	178.0	3.75%	8.7	1.08%	motif file (matrix)	svg
84	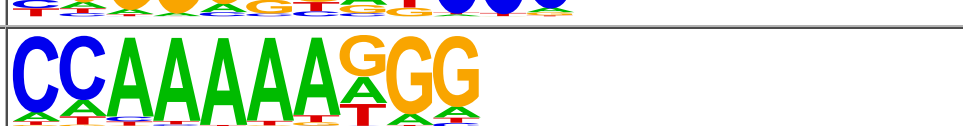	SEP3(MADS)/Arabidoposis-Flower-Sep3-ChIP-Seq/Homer	1e-47	-1.086e+02	0.0000	608.0	12.82%	56.6	6.97%	motif file (matrix)	svg
85	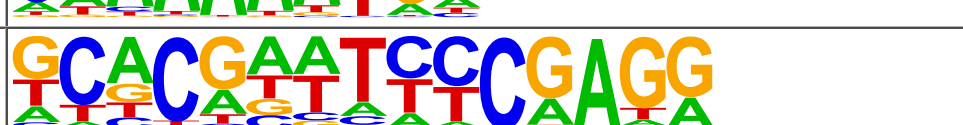	PLT1(AP2EREBP)/colamp-PLT1-DAP-Seq(GSE60143)/Homer	1e-46	-1.071e+02	0.0000	89.0	1.88%	2.1	0.25%	motif file (matrix)	svg
86	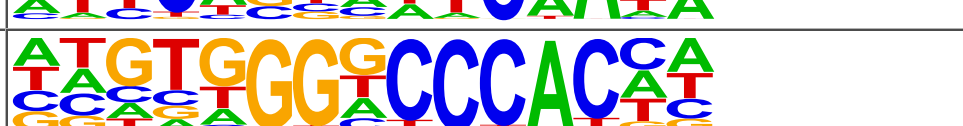	At1g69690(TCP)/colamp-At1g69690-DAP-Seq(GSE60143)/Homer	1e-45	-1.055e+02	0.0000	229.0	4.83%	13.4	1.65%	motif file (matrix)	svg
87	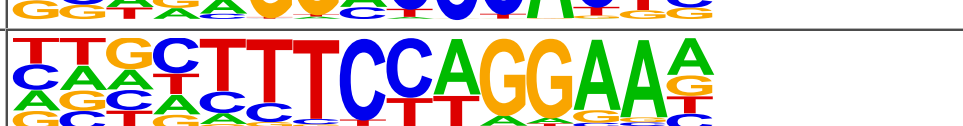	Bcl6(Zf)/Liver-Bcl6-ChIP-Seq(GSE31578)/Homer	1e-45	-1.043e+02	0.0000	694.0	14.64%	68.5	8.44%	motif file (matrix)	svg
88	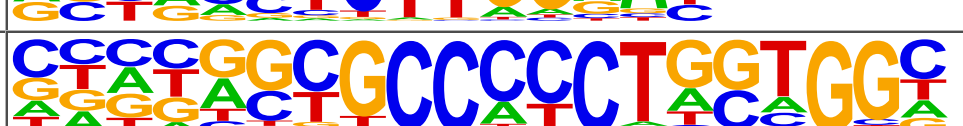	BORIS(Zf)/K562-CTCFL-ChIP-Seq(GSE32465)/Homer	1e-45	-1.041e+02	0.0000	671.0	14.15%	65.4	8.05%	motif file (matrix)	svg
89	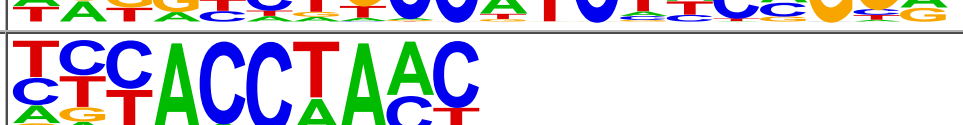	MYB67(MYB)/col-MYB67-DAP-Seq(GSE60143)/Homer	1e-44	-1.033e+02	0.0000	472.0	9.95%	40.1	4.93%	motif file (matrix)	svg
90	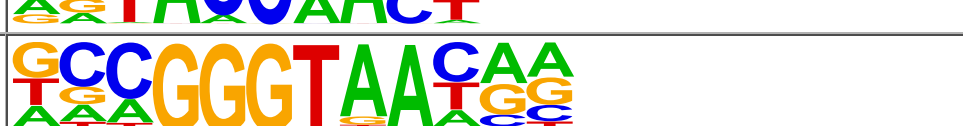	REB1/SacCer-Promoters/Homer	1e-44	-1.033e+02	0.0000	147.0	3.10%	6.8	0.83%	motif file (matrix)	svg
91	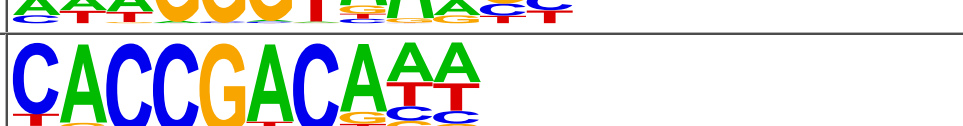	At4g31060(AP2EREBP)/colamp-At4g31060-DAP-Seq(GSE60143)/Homer	1e-44	-1.026e+02	0.0000	536.0	11.30%	48.9	6.03%	motif file (matrix)	svg
92	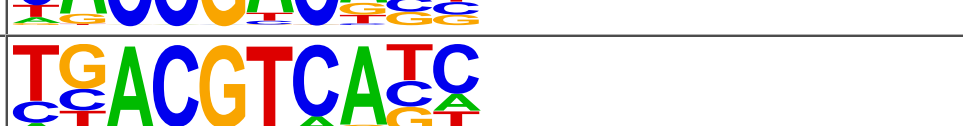	TGA6(bZIP)/colamp-TGA6-DAP-Seq(GSE60143)/Homer	1e-43	-1.013e+02	0.0000	376.0	7.93%	29.1	3.59%	motif file (matrix)	svg
93	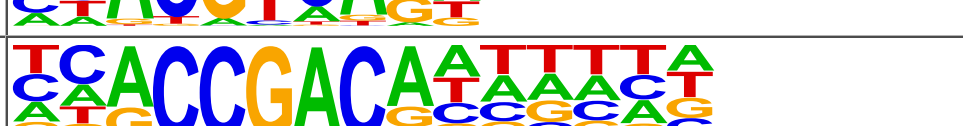	AT3G16280(AP2EREBP)/colamp-AT3G16280-DAP-Seq(GSE60143)/Homer	1e-43	-9.940e+01	0.0000	475.0	10.02%	41.9	5.16%	motif file (matrix)	svg
94	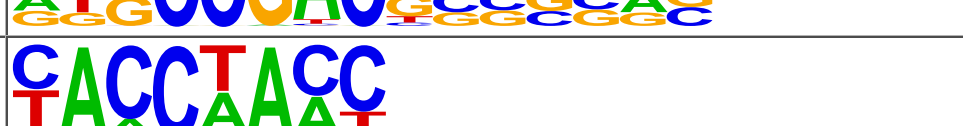	MYB55(MYB)/colamp-MYB55-DAP-Seq(GSE60143)/Homer	1e-43	-9.910e+01	0.0000	499.0	10.52%	44.4	5.46%	motif file (matrix)	svg
95	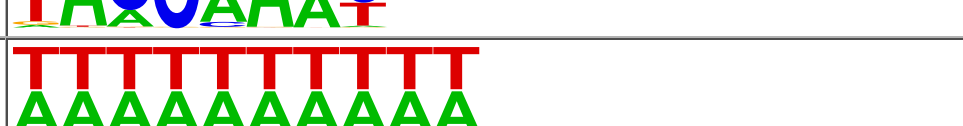	SeqBias: A/T bias	1e-42	-9.808e+01	0.0000	2409.0	50.80%	332.1	40.89%	motif file (matrix)	svg
96	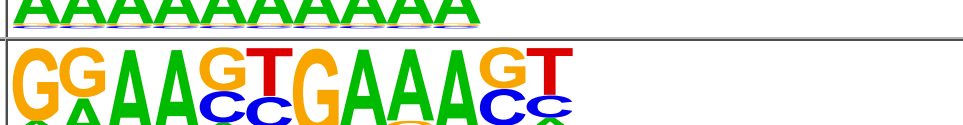	IRF8(IRF)/BMDM-IRF8-ChIP-Seq(GSE77884)/Homer	1e-42	-9.798e+01	0.0000	272.0	5.74%	18.8	2.31%	motif file (matrix)	svg
97	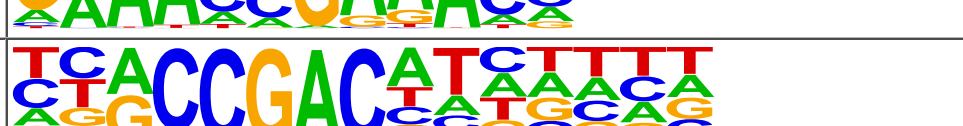	CBF2(AP2EREBP)/colamp-CBF2-DAP-Seq(GSE60143)/Homer	1e-41	-9.668e+01	0.0000	644.0	13.58%	63.2	7.78%	motif file (matrix)	svg
98	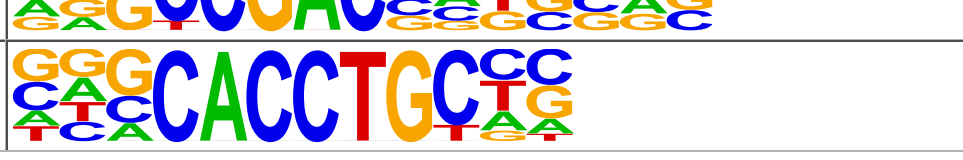	Slug(Zf)/Mesoderm-Snai2-ChIP-Seq(GSE61475)/Homer	1e-41	-9.562e+01	0.0000	695.0	14.66%	71.0	8.74%	motif file (matrix)	svg

99	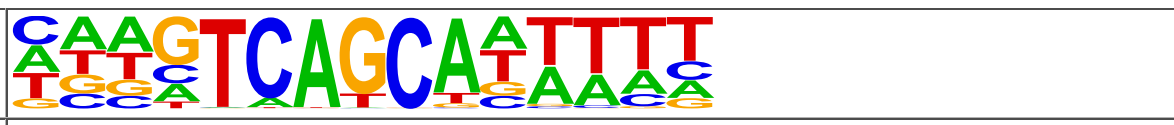	MafF(bZIP)/HepG2-MafF-ChIP-Seq(GSE31477)/Homer	1e-41	-9.545e+01	0.0000	100.0	2.11%	3.6	0.45%	motif file (matrix)	svg
100	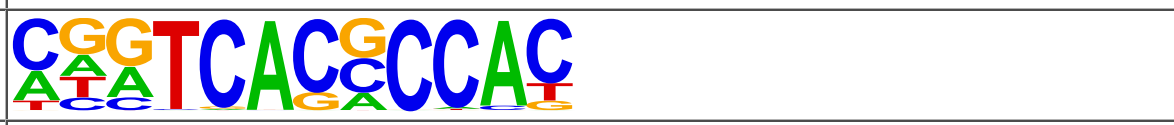	Srebp2(bHLH)/HepG2-Srebp2-ChIP-Seq(GSE31477)/Homer	1e-41	-9.545e+01	0.0000	100.0	2.11%	4.0	0.49%	motif file (matrix)	svg
101	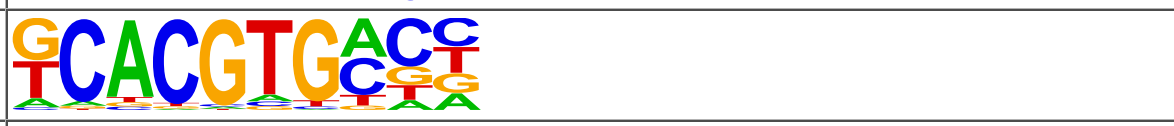	bHLHE41(bHLH)/proB-Bhlhe41-ChIP-Seq(GSE93764)/Homer	1e-41	-9.499e+01	0.0000	1756.0	37.03%	227.3	27.98%	motif file (matrix)	svg
102	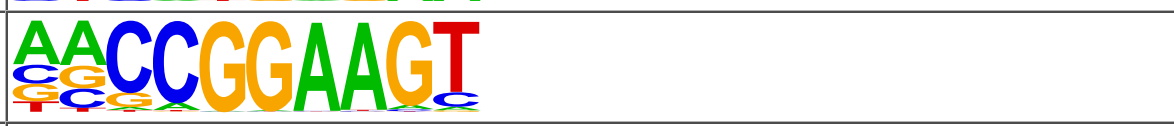	ETS(ETS)/Promoter/Homer	1e-41	-9.499e+01	0.0000	469.0	9.89%	41.8	5.14%	motif file (matrix)	svg
103	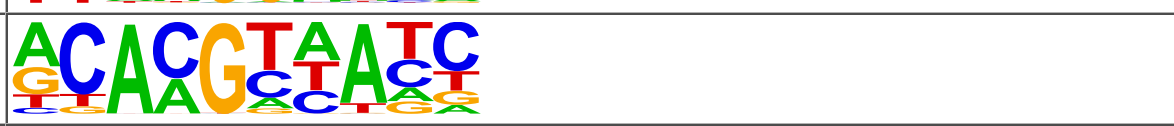	ANAC038(NAC)/col-ANAC038-DAP-Seq(GSE60143)/Homer	1e-40	-9.327e+01	0.0000	1103.0	23.26%	128.2	15.78%	motif file (matrix)	svg
104	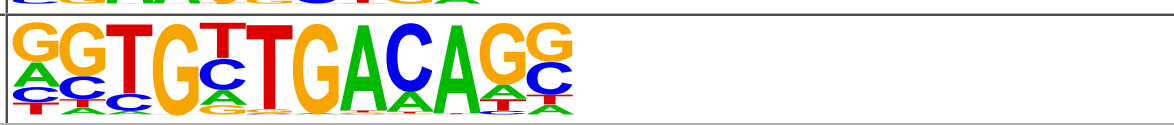	Tbx20(T-box)/Heart-Tbx20-ChIP-Seq(GSE29636)/Homer	1e-40	-9.324e+01	0.0000	140.0	2.95%	6.0	0.74%	motif file (matrix)	svg
105	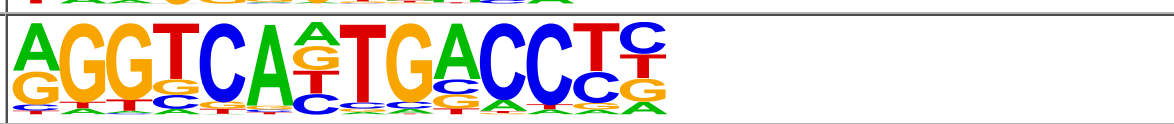	FXR(NR),IR1/Liver-FXR-ChIP-Seq(Chong_et_al.)/Homer	1e-40	-9.287e+01	0.0000	322.0	6.79%	24.5	3.01%	motif file (matrix)	svg
106	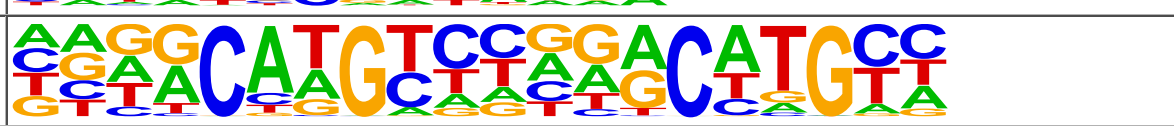	p63(p53)/Keratinocyte-p63-ChIP-Seq(GSE17611)/Homer	1e-39	-9.107e+01	0.0000	216.0	4.56%	13.5	1.66%	motif file (matrix)	svg
107	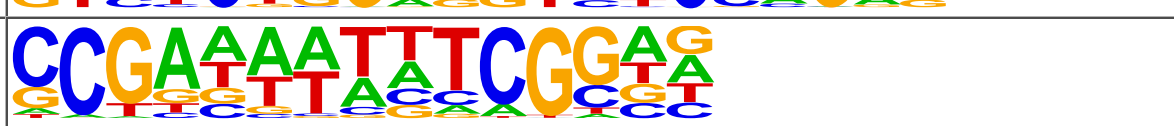	CDM1(C3H)/colamp-CDM1-DAP-Seq(GSE60143)/Homer	1e-39	-9.101e+01	0.0000	81.0	1.71%	2.4	0.29%	motif file (matrix)	svg
108	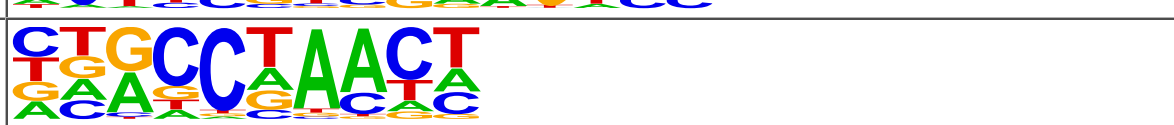	BOS1(MYB)/col-BOS1-DAP-Seq(GSE60143)/Homer	1e-39	-9.060e+01	0.0000	487.0	10.27%	44.3	5.45%	motif file (matrix)	svg
109	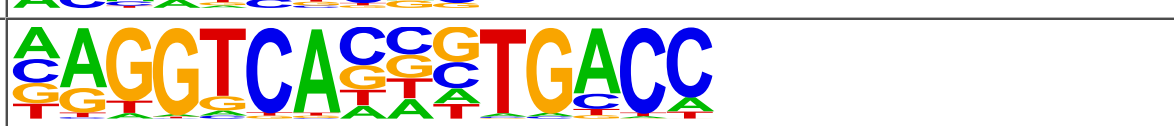	ERE(NR),IR3/MCF7-ERa-ChIP-Seq(Unpublished)/Homer	1e-39	-9.040e+01	0.0000	184.0	3.88%	10.5	1.30%	motif file (matrix)	svg
110	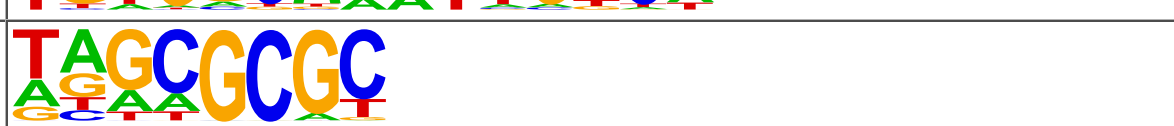	DPL-1(E2F)/cElegans-Adult-ChIP-Seq(modEncode)/Homer	1e-38	-8.851e+01	0.0000	2991.0	63.07%	436.4	53.73%	motif file (matrix)	svg
111	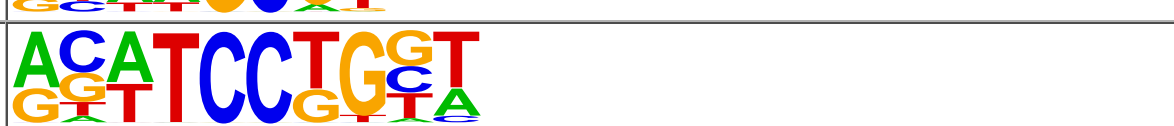	SPDEF(ETS)/VCaP-SPDEF-ChIP-Seq(SRA014231)/Homer	1e-38	-8.781e+01	0.0000	784.0	16.53%	84.0	10.35%	motif file (matrix)	svg
112	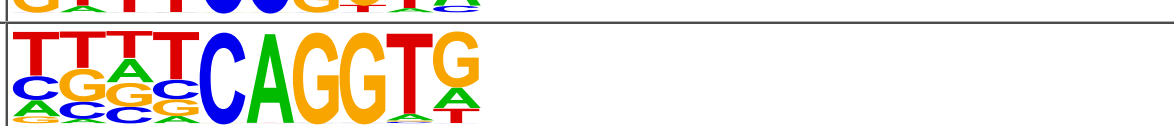	NGA4(ABI3VP1)/col-NGA4-DAP-Seq(GSE60143)/Homer	1e-37	-8.596e+01	0.0000	2008.0	42.35%	271.1	33.38%	motif file (matrix)	svg
113	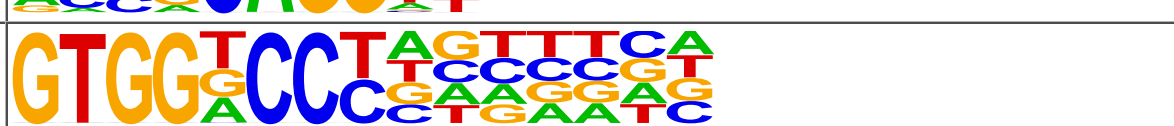	TCP16(TCP)/colamp-TCP16-DAP-Seq(GSE60143)/Homer	1e-36	-8.485e+01	0.0000	684.0	14.42%	71.8	8.84%	motif file (matrix)	svg
114	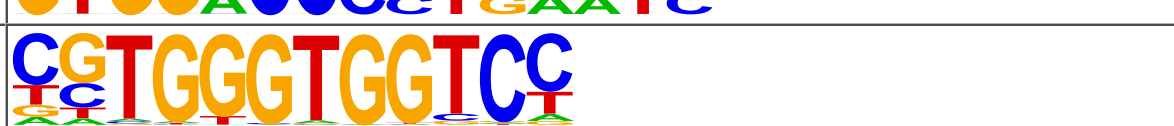	GLI3(Zf)/Limb-GLI3-ChIP-Chip(GSE11077)/Homer	1e-36	-8.334e+01	0.0000	93.0	1.96%	3.7	0.46%	motif file (matrix)	svg
115	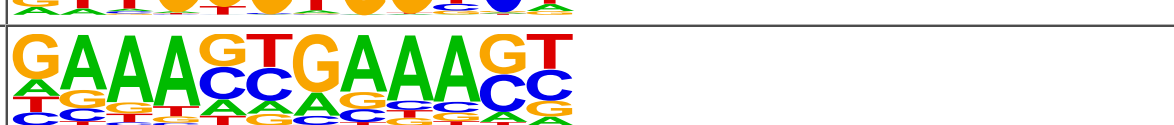	IRF2(IRF)/Erythroblas-IRF2-ChIP-Seq(GSE36985)/Homer	1e-36	-8.334e+01	0.0000	93.0	1.96%	4.0	0.49%	motif file (matrix)	svg
116	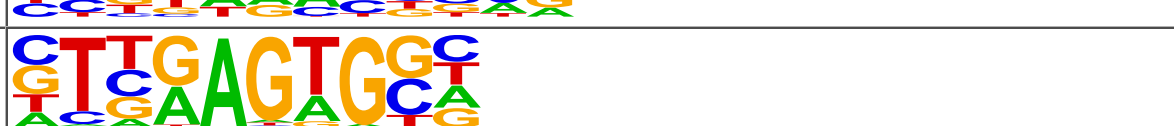	Nkx2.2(Homeobox)/NPC-Nkx2.2-ChIP-Seq(GSE61673)/Homer	1e-36	-8.298e+01	0.0000	1295.0	27.31%	160.3	19.74%	motif file (matrix)	svg
117	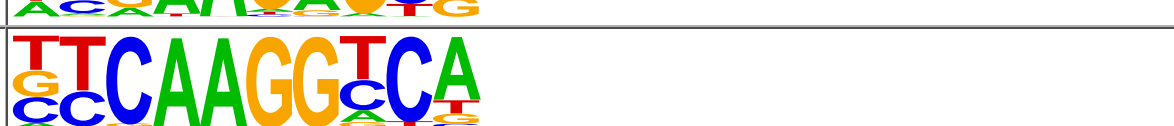	Nr5a2(NR)/mES-Nr5a2-ChIP-Seq(GSE19019)/Homer	1e-35	-8.282e+01	0.0000	319.0	6.73%	26.0	3.20%	motif file (matrix)	svg
118	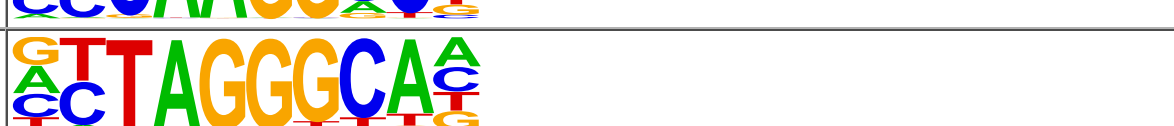	TBP3(MYBrelated)/col-TBP3-DAP-Seq(GSE60143)/Homer	1e-35	-8.189e+01	0.0000	521.0	10.99%	50.5	6.22%	motif file (matrix)	svg
119	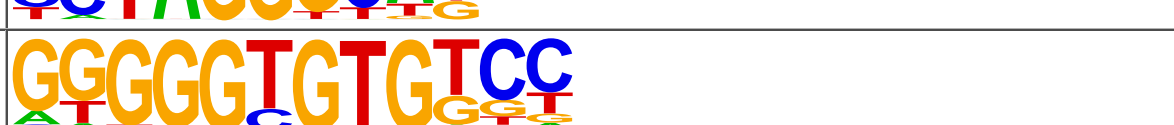	KLF10(Zf)/HEK293-KLF10.GFP-ChIP-Seq(GSE58341)/Homer	1e-35	-8.169e+01	0.0000	1058.0	22.31%	125.3	15.43%	motif file (matrix)	svg
120	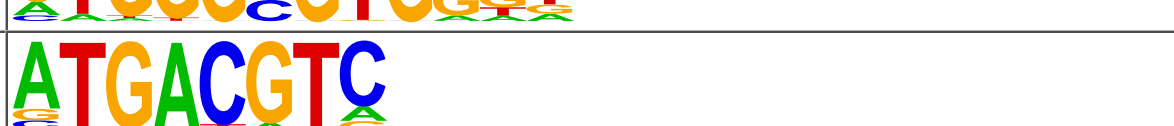	TGA10(bZIP)/colamp-TGA10-DAP-Seq(GSE60143)/Homer	1e-35	-8.066e+01	0.0000	325.0	6.85%	26.2	3.23%	motif file (matrix)	svg
121	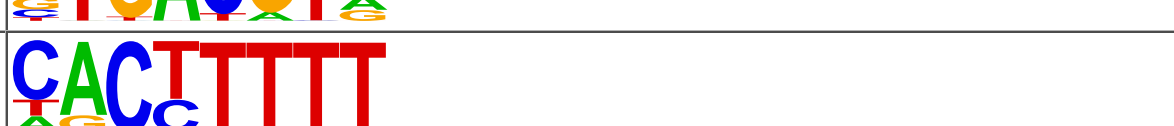	At1g64620(C2C2dof)/colamp-At1g64620-DAP-Seq(GSE60143)/Homer	1e-34	-7.903e+01	0.0000	803.0	16.93%	89.3	11.00%	motif file (matrix)	svg
122	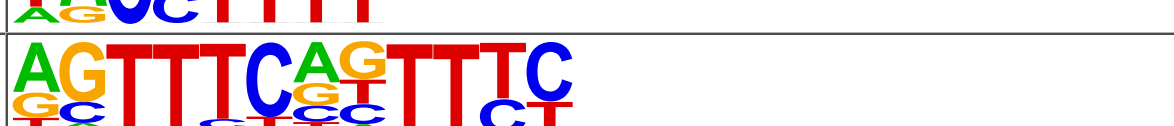	IRF3(IRF)/BMDM-Irf3-ChIP-Seq(GSE67343)/Homer	1e-33	-7.790e+01	0.0000	269.0	5.67%	20.7	2.55%	motif file (matrix)	svg
123	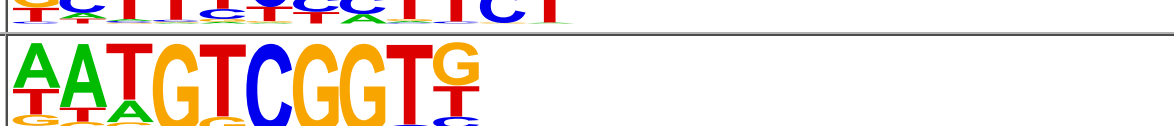	DREB19(AP2EREBP)/colamp-DREB19-DAP-Seq(GSE60143)/Homer	1e-33	-7.624e+01	0.0000	762.0	16.07%	85.0	10.46%	motif file (matrix)	svg
124	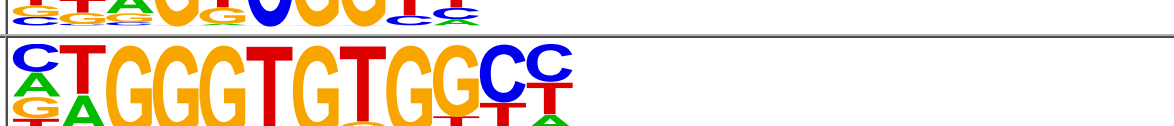	EKLF(Zf)/Erythrocyte-Klf1-ChIP-Seq(GSE20478)/Homer	1e-33	-7.606e+01	0.0000	139.0	2.93%	7.1	0.87%	motif file (matrix)	svg
125	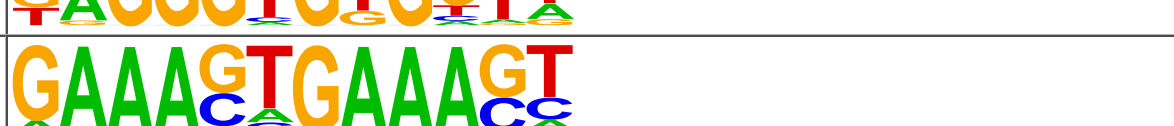	IRF1(IRF)/PBMC-IRF1-ChIP-Seq(GSE43036)/Homer	1e-32	-7.481e+01	0.0000	138.0	2.91%	7.6	0.94%	motif file (matrix)	svg
126	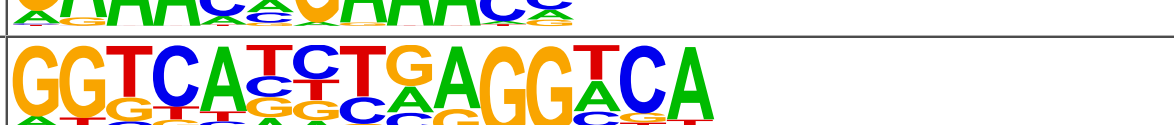	THRa(NR)/C17.2-THRa-ChIP-Seq(GSE38347)/Homer	1e-32	-7.434e+01	0.0000	317.0	6.68%	26.1	3.21%	motif file (matrix)	svg
127	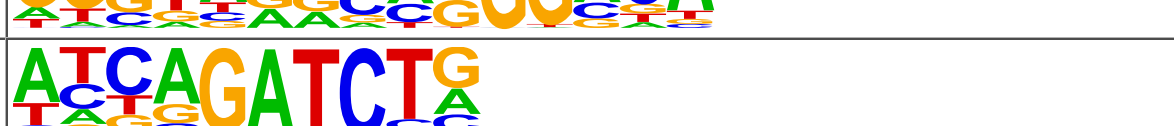	GATA14(C2C2gata)/col-GATA14-DAP-Seq(GSE60143)/Homer	1e-31	-7.344e+01	0.0000	382.0	8.06%	34.9	4.30%	motif file (matrix)	svg
128	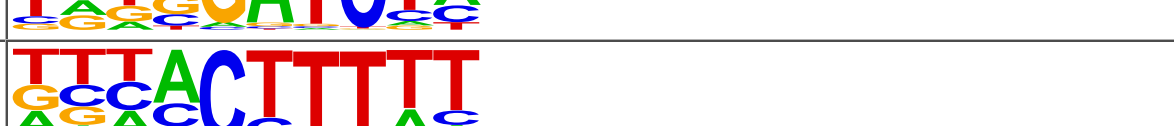	AT3G52440(C2C2dof)/colamp-AT3G52440-DAP-Seq(GSE60143)/Homer	1e-31	-7.257e+01	0.0000	997.0	21.02%	119.7	14.73%	motif file (matrix)	svg
129	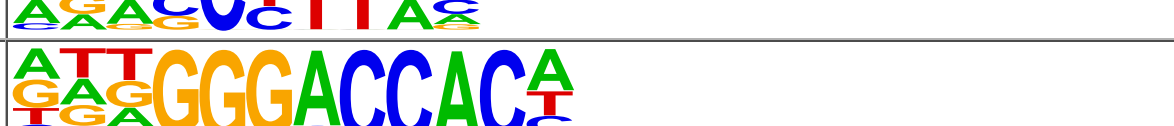	PTF1(TCP)/colamp-PTF1-DAP-Seq(GSE60143)/Homer	1e-31	-7.236e+01	0.0000	100.0	2.11%	4.5	0.55%	motif file (matrix)	svg
130	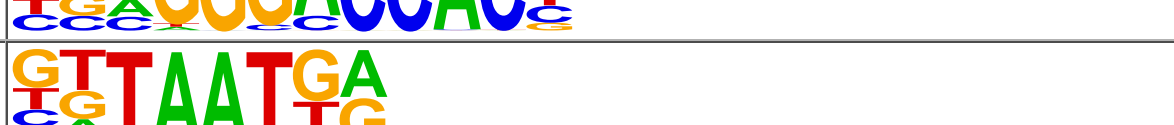	Nkx6.1(Homeobox)/Islet-Nkx6.1-ChIP-Seq(GSE40975)/Homer	1e-31	-7.196e+01	0.0000	948.0	19.99%	112.3	13.82%	motif file (matrix)	svg
131	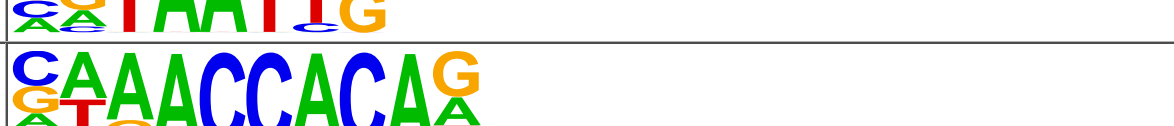	RUNX(Runt)/HPC7-Runx1-ChIP-Seq(GSE22178)/Homer	1e-31	-7.161e+01	0.0000	355.0	7.49%	31.5	3.88%	motif file (matrix)	svg
132	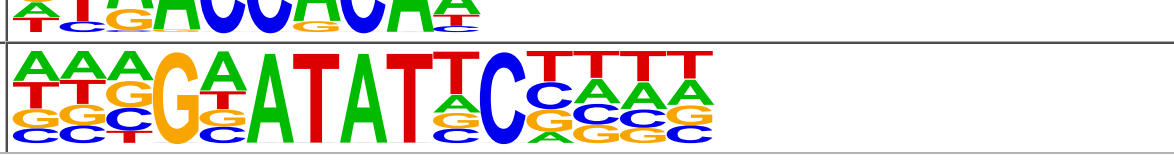	AT2G20400(G2like)/colamp-AT2G20400-DAP-Seq(GSE60143)/Homer	1e-30	-7.054e+01	0.0000	52.0	1.10%	0.7	0.09%	motif file (matrix)	svg

133	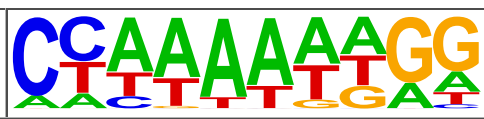	RIN(MADS)/Tomato-RIN-ChIP-Seq(GSE116581)/Homer	1e-30	-6.938e+01	0.0000	493.0	10.40%	49.4	6.08%	motif file (matrix)	svg
134	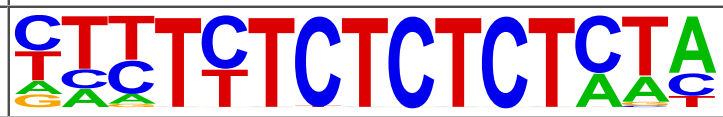	BPC6(BBRBPC)/col-BPC6-DAP-Seq(GSE60143)/Homer	1e-29	-6.875e+01	0.0000	110.0	2.32%	5.3	0.65%	motif file (matrix)	svg
135	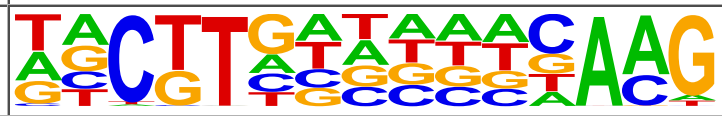	ANAC028(NAC)/col-ANAC028-DAP-Seq(GSE60143)/Homer	1e-29	-6.875e+01	0.0000	267.0	5.63%	21.5	2.65%	motif file (matrix)	svg
136	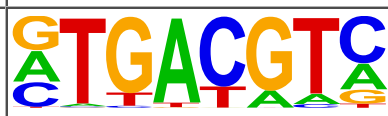	TGA9(bZIP)/colamp-TGA9-DAP-Seq(GSE60143)/Homer	1e-29	-6.871e+01	0.0000	537.0	11.32%	56.0	6.89%	motif file (matrix)	svg
137	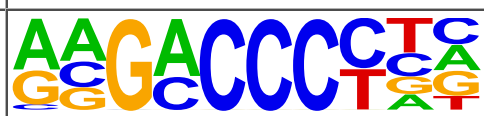	LRF(Zf)/Erythroblasts-ZBTB7A-ChIP-Seq(GSE74977)/Homer	1e-29	-6.823e+01	0.0000	2809.0	59.24%	414.1	50.98%	motif file (matrix)	svg
138	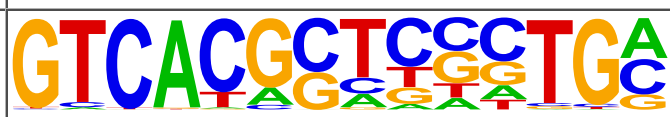	PAX5(Paired,Homeobox),condensed/GM12878-PAX5-ChIP-Seq(GSE32465)/Homer	1e-29	-6.776e+01	0.0000	121.0	2.55%	6.1	0.75%	motif file (matrix)	svg
139	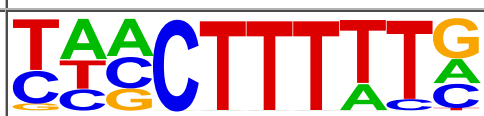	dof24(C2C2dof)/col-dof24-DAP-Seq(GSE60143)/Homer	1e-29	-6.763e+01	0.0000	1026.0	21.64%	125.3	15.43%	motif file (matrix)	svg
140	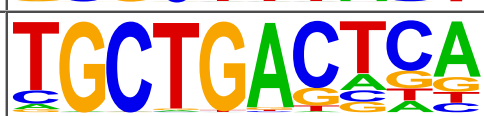	MafA(bZIP)/Islet-MafA-ChIP-Seq(GSE30298)/Homer	1e-29	-6.754e+01	0.0000	520.0	10.97%	53.9	6.64%	motif file (matrix)	svg
141	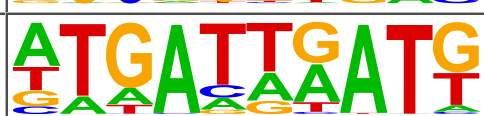	LIN-39(Homeobox)/cElegans.L3-LIN39-ChIP-Seq(modEncode)/Homer	1e-29	-6.707e+01	0.0000	451.0	9.51%	44.8	5.52%	motif file (matrix)	svg
142	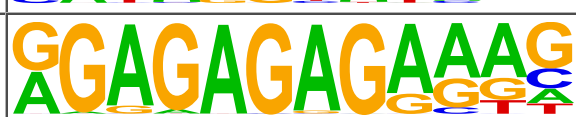	FRS9(ND)/col-FRS9-DAP-Seq(GSE60143)/Homer	1e-28	-6.619e+01	0.0000	281.0	5.93%	23.4	2.88%	motif file (matrix)	svg
143	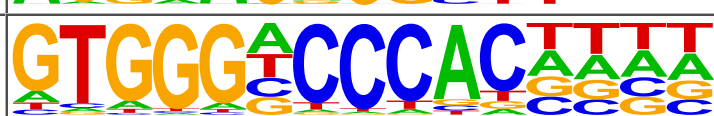	At2g45680(TCP)/colamp-At2g45680-DAP-Seq(GSE60143)/Homer	1e-28	-6.616e+01	0.0000	50.0	1.05%	1.6	0.20%	motif file (matrix)	svg
144	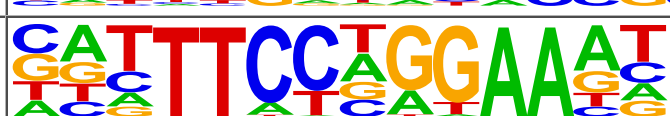	STAT1(Stat)/HelaS3-STAT1-ChIP-Seq(GSE12782)/Homer	1e-28	-6.592e+01	0.0000	172.0	3.63%	11.1	1.36%	motif file (matrix)	svg
145	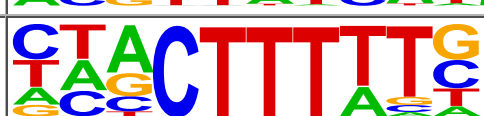	AT1G47655(C2C2dof)/colamp-AT1G47655-DAP-Seq(GSE60143)/Homer	1e-28	-6.512e+01	0.0000	1590.0	33.53%	213.9	26.33%	motif file (matrix)	svg
146	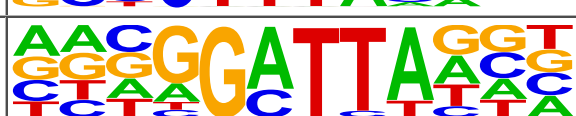	bcd(Homeobox)/Embryo-Bcd-ChIP-Seq(GSE86966)/Homer	1e-27	-6.436e+01	0.0000	544.0	11.47%	57.4	7.06%	motif file (matrix)	svg
147	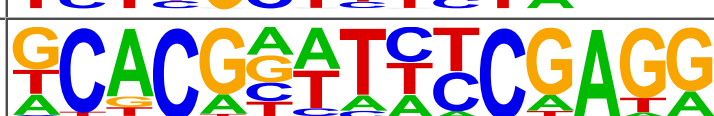	PLT3(AP2EREBP)/col-PLT3-DAP-Seq(GSE60143)/Homer	1e-27	-6.400e+01	0.0000	49.0	1.03%	1.9	0.23%	motif file (matrix)	svg
148	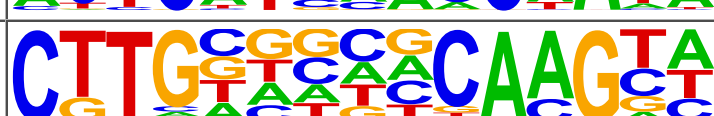	AT1G19040(NAC)/col-AT1G19040-DAP-Seq(GSE60143)/Homer	1e-27	-6.311e+01	0.0000	66.0	1.39%	2.8	0.34%	motif file (matrix)	svg
149	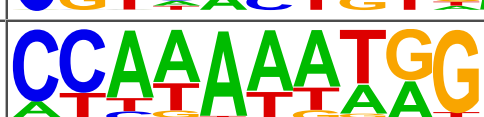	TAGL1(MADS)/Tomato-TAGL1-ChIP-Seq(GSE116581)/Homer	1e-27	-6.242e+01	0.0000	496.0	10.46%	51.9	6.39%	motif file (matrix)	svg
150	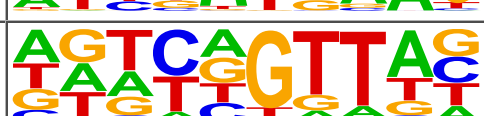	MYB33(MYB)/col-MYB33-DAP-Seq(GSE60143)/Homer	1e-26	-6.082e+01	0.0000	674.0	14.21%	76.5	9.42%	motif file (matrix)	svg
151	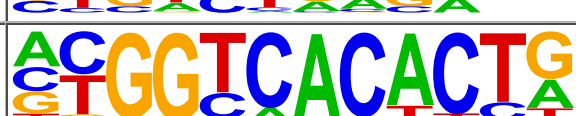	Unknown1(NR/Ini-like)/Drosophila-Promoters/Homer	1e-26	-6.043e+01	0.0000	126.0	2.66%	7.2	0.89%	motif file (matrix)	svg
152	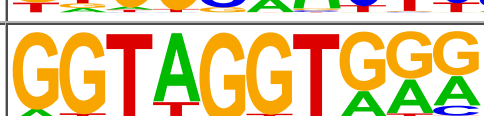	MYB17(MYB)/colamp-MYB17-DAP-Seq(GSE60143)/Homer	1e-26	-5.990e+01	0.0000	346.0	7.30%	32.5	4.01%	motif file (matrix)	svg
153	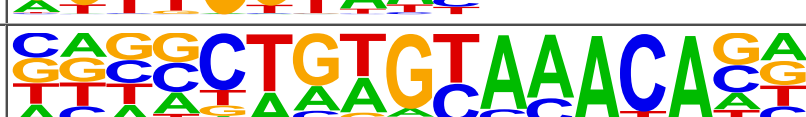	Fox:Ebox(Forkhead,bHLH)/Panc1-Foxa2-ChIP-Seq(GSE47459)/Homer	1e-25	-5.944e+01	0.0000	400.0	8.44%	39.4	4.85%	motif file (matrix)	svg
154	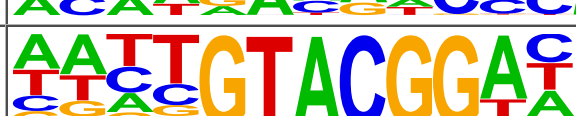	SPL13(SBP)/col-SPL13-DAP-Seq(GSE60143)/Homer	1e-25	-5.924e+01	0.0000	78.0	1.64%	3.3	0.41%	motif file (matrix)	svg
155	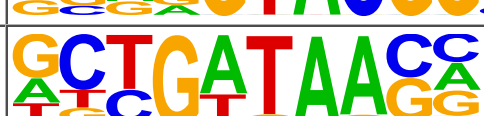	Unknown5/Drosophila-Promoters/Homer	1e-25	-5.921e+01	0.0000	337.0	7.11%	31.1	3.83%	motif file (matrix)	svg
156	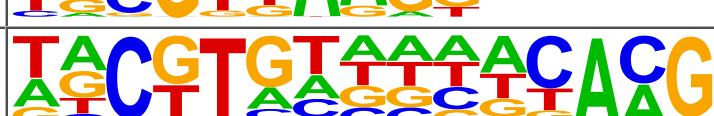	CUC2(NAC)/colamp-CUC2-DAP-Seq(GSE60143)/Homer	1e-25	-5.867e+01	0.0000	155.0	3.27%	10.4	1.28%	motif file (matrix)	svg
157	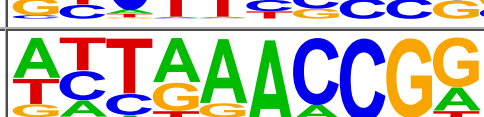	Unknown3/Arabidopsis-Promoters/Homer	1e-25	-5.824e+01	0.0000	102.0	2.15%	5.9	0.72%	motif file (matrix)	svg
158	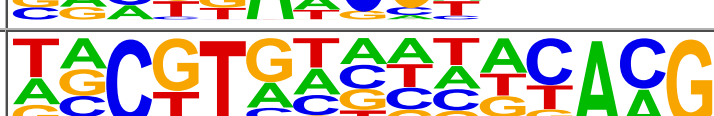	ANAC087(NAC)/col-ANAC087-DAP-Seq(GSE60143)/Homer	1e-25	-5.799e+01	0.0000	164.0	3.46%	11.2	1.38%	motif file (matrix)	svg
159	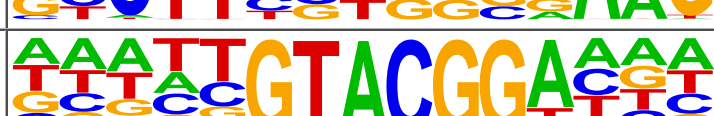	SPL14(SBP)/col-SPL14-DAP-Seq(GSE60143)/Homer	1e-25	-5.767e+01	0.0000	134.0	2.83%	8.9	1.10%	motif file (matrix)	svg
160	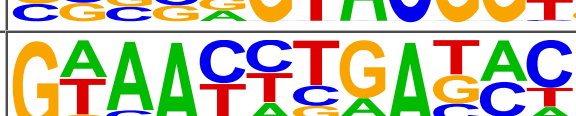	Six2(Homeobox)/NephronProgenitor-Six2-ChIP-Seq(GSE39837)/Homer	1e-24	-5.743e+01	0.0000	509.0	10.73%	54.4	6.69%	motif file (matrix)	svg
161	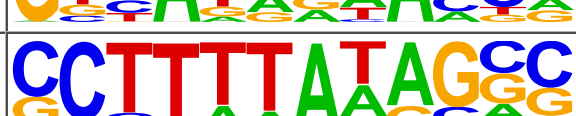	TATA-Box(TBP)/Promoter/Homer	1e-24	-5.709e+01	0.0000	523.0	11.03%	56.3	6.94%	motif file (matrix)	svg
162	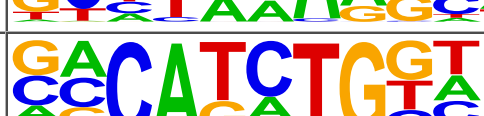	TCF4(bHLH)/SHSY5Y-TCF4-ChIP-Seq(GSE96915)/Homer	1e-24	-5.693e+01	0.0000	1026.0	21.64%	129.1	15.90%	motif file (matrix)	svg
163	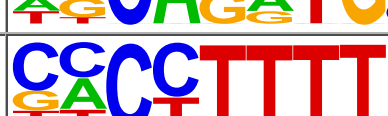	AT5G02460(C2C2dof)/col-AT5G02460-DAP-Seq(GSE60143)/Homer	1e-24	-5.627e+01	0.0000	1175.0	24.78%	153.0	18.84%	motif file (matrix)	svg
164	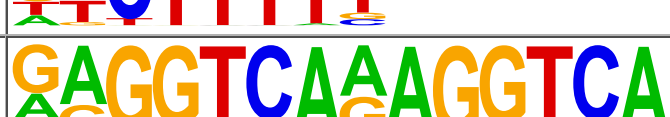	TR4(NR),DR1/Hela-TR4-ChIP-Seq(GSE24685)/Homer	1e-24	-5.622e+01	0.0000	62.0	1.31%	2.6	0.32%	motif file (matrix)	svg
165	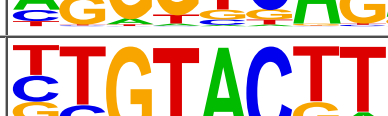	SPL9(SBP)/colamp-SPL9-DAP-Seq(GSE60143)/Homer	1e-24	-5.564e+01	0.0000	1283.0	27.06%	169.6	20.88%	motif file (matrix)	svg
166	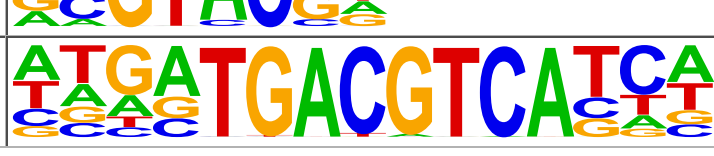	TGA3(bZIP)/colamp-TGA3-DAP-Seq(GSE60143)/Homer	1e-24	-5.558e+01	0.0000	45.0	0.95%	1.6	0.20%	motif file (matrix)	svg

167	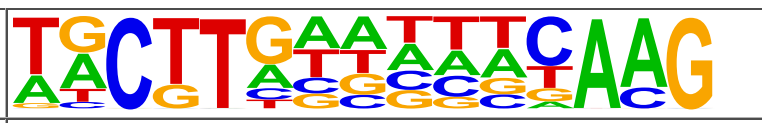	VND3(NAC)/colamp-VND3-DAP-Seq(GSE60143)/Homer	1e-24	-5.544e+01	0.0000	233.0	4.91%	19.1	2.35%	motif file (matrix)	svg
168	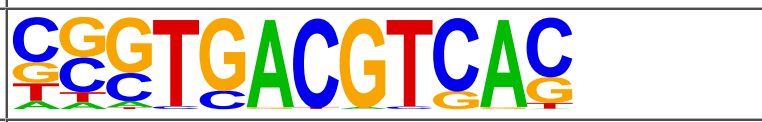	CRE(bZIP)/Promoter/Homer	1e-24	-5.539e+01	0.0000	283.0	5.97%	25.6	3.15%	motif file (matrix)	svg
169	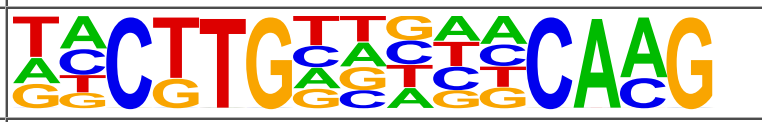	ANAC017(NAC)/colamp-ANAC017-DAP-Seq(GSE60143)/Homer	1e-24	-5.537e+01	0.0000	88.0	1.86%	4.9	0.61%	motif file (matrix)	svg
170	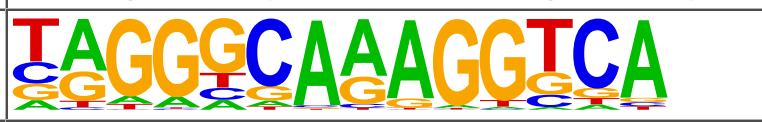	RXR(NR),DR1/3T3L1-RXR-ChIP-Seq(GSE13511)/Homer	1e-23	-5.470e+01	0.0000	751.0	15.84%	89.9	11.07%	motif file (matrix)	svg
171	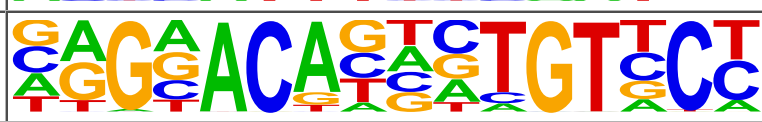	GRE(NR),IR3/A549-GR-ChIP-Seq(GSE32465)/Homer	1e-23	-5.454e+01	0.0000	61.0	1.29%	2.4	0.29%	motif file (matrix)	svg
172	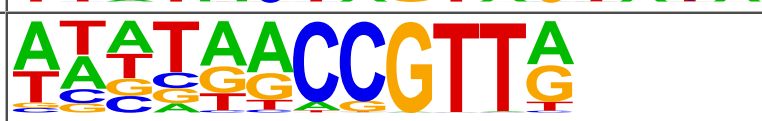	MYB3R4(MYB)/col-MYB3R4-DAP-Seq(GSE60143)/Homer	1e-23	-5.454e+01	0.0000	61.0	1.29%	2.4	0.30%	motif file (matrix)	svg
173	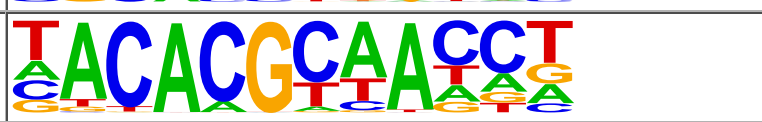	ANAC079(NAC)/colamp-ANAC079-DAP-Seq(GSE60143)/Homer	1e-23	-5.418e+01	0.0000	160.0	3.37%	11.4	1.41%	motif file (matrix)	svg
174	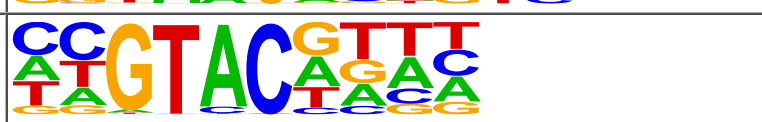	SPL1(SBP)/colamp-SPL1-DAP-Seq(GSE60143)/Homer	1e-23	-5.402e+01	0.0000	865.0	18.24%	106.0	13.05%	motif file (matrix)	svg
175	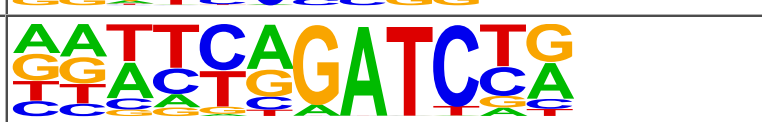	GATA4(C2C2gata)/col-GATA4-DAP-Seq(GSE60143)/Homer	1e-23	-5.329e+01	0.0000	382.0	8.06%	38.9	4.79%	motif file (matrix)	svg
176	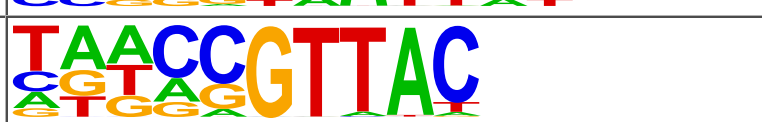	MYB118(MYB)/colamp-MYB118-DAP-Seq(GSE60143)/Homer	1e-23	-5.320e+01	0.0000	109.0	2.30%	6.9	0.85%	motif file (matrix)	svg
177	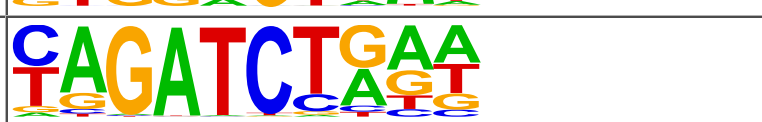	GATA12(C2C2gata)/col-GATA12-DAP-Seq(GSE60143)/Homer	1e-22	-5.196e+01	0.0000	310.0	6.54%	29.9	3.68%	motif file (matrix)	svg
178	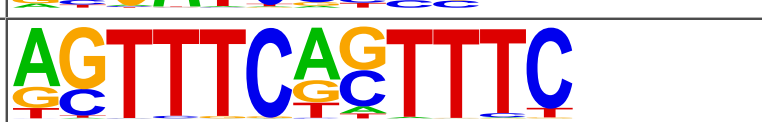	ISRE(IRF)/ThioMac-LPS-Expression(GSE23622)/Homer	1e-22	-5.182e+01	0.0000	73.0	1.54%	3.2	0.39%	motif file (matrix)	svg
179	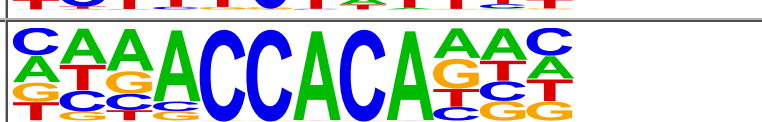	RUNX2(Runt)/PCa-RUNX2-ChIP-Seq(GSE33889)/Homer	1e-22	-5.133e+01	0.0000	348.0	7.34%	34.0	4.19%	motif file (matrix)	svg
180	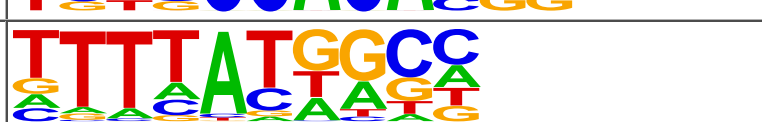	Hoxa11(Homeobox)/ChickenMSG-Hoxa11.Flag-ChIP-Seq(GSE86088)/Homer	1e-22	-5.103e+01	0.0000	964.0	20.33%	122.1	15.03%	motif file (matrix)	svg
181	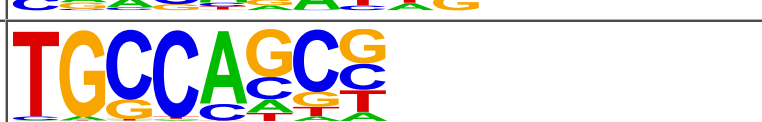	HIC1(Zf)/Treg-ZBTB29-ChIP-Seq(GSE99889)/Homer	1e-22	-5.087e+01	0.0000	2444.0	51.54%	361.2	44.47%	motif file (matrix)	svg
182	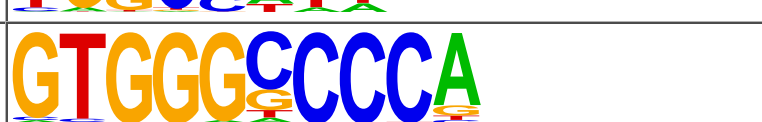	ZNF692(Zf)/HEK293-ZNF692.GFP-ChIP-Seq(GSE58341)/Homer	1e-21	-5.058e+01	0.0000	284.0	5.99%	26.3	3.24%	motif file (matrix)	svg
183	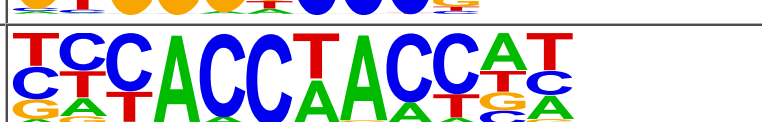	ATY19(MYB)/col-ATY19-DAP-Seq(GSE60143)/Homer	1e-21	-5.053e+01	0.0000	362.0	7.63%	36.2	4.45%	motif file (matrix)	svg
184	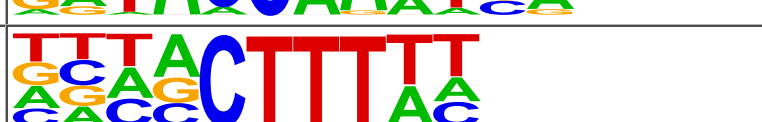	AT5G66940(C2C2dof)/col-AT5G66940-DAP-Seq(GSE60143)/Homer	1e-21	-5.031e+01	0.0000	889.0	18.75%	111.1	13.68%	motif file (matrix)	svg
185	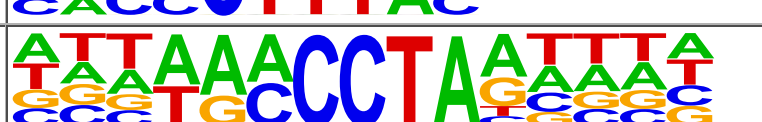	AT1G72740(MYBrelated)/colamp-AT1G72740-DAP-Seq(GSE60143)/Homer	1e-21	-5.014e+01	0.0000	502.0	10.59%	55.8	6.87%	motif file (matrix)	svg
186	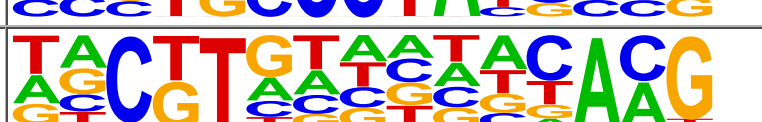	ANAC092(NAC)/colamp-ANAC092-DAP-Seq(GSE60143)/Homer	1e-21	-4.982e+01	0.0000	200.0	4.22%	16.3	2.01%	motif file (matrix)	svg
187	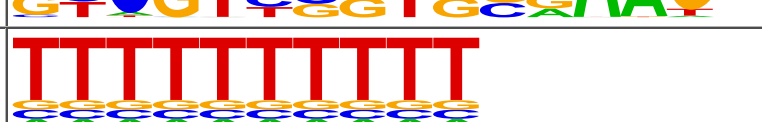	VRN1(ABI3VP1)/col-VRN1-DAP-Seq(GSE60143)/Homer	1e-21	-4.939e+01	0.0000	164.0	3.46%	12.3	1.52%	motif file (matrix)	svg
188	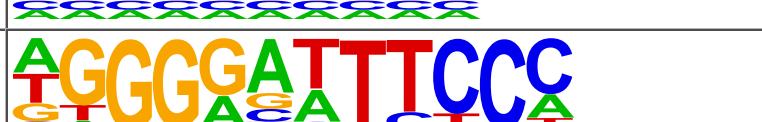	NFkB-p65(RHD)/GM12787-p65-ChIP-Seq(GSE19485)/Homer	1e-21	-4.929e+01	0.0000	290.0	6.12%	27.7	3.41%	motif file (matrix)	svg
189	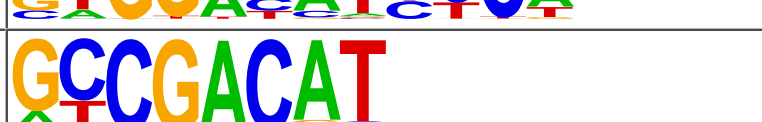	DDF1(AP2EREBP)/col-DDF1-DAP-Seq(GSE60143)/Homer	1e-21	-4.878e+01	0.0000	492.0	10.38%	54.4	6.70%	motif file (matrix)	svg
190	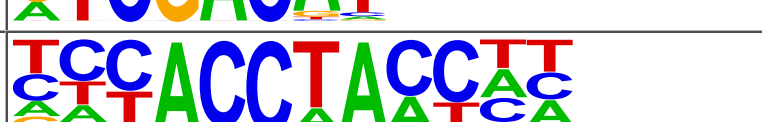	MYB10(MYB)/col-MYB10-DAP-Seq(GSE60143)/Homer	1e-21	-4.859e+01	0.0000	181.0	3.82%	14.3	1.76%	motif file (matrix)	svg
191	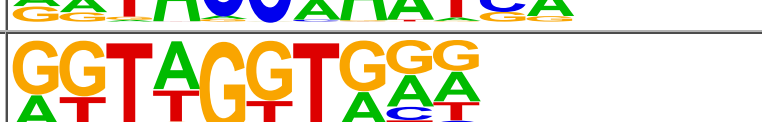	MYB93(MYB)/colamp-MYB93-DAP-Seq(GSE60143)/Homer	1e-21	-4.857e+01	0.0000	640.0	13.50%	75.3	9.27%	motif file (matrix)	svg
192	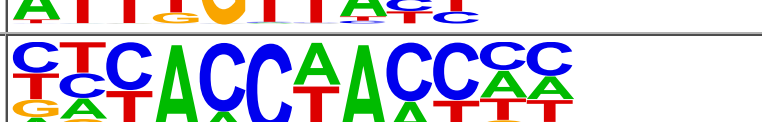	MYB63(MYB)/col-MYB63-DAP-Seq(GSE60143)/Homer	1e-21	-4.841e+01	0.0000	232.0	4.89%	20.7	2.55%	motif file (matrix)	svg
193	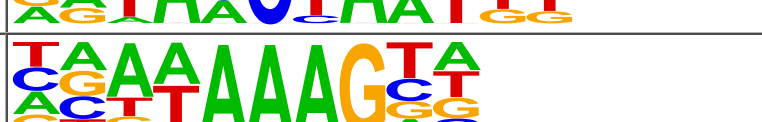	dof45(C2C2dof)/col-dof45-DAP-Seq(GSE60143)/Homer	1e-20	-4.818e+01	0.0000	1216.0	25.64%	162.4	20.00%	motif file (matrix)	svg
194	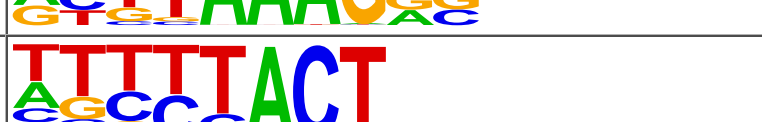	At3g60580(C2H2)/col-At3g60580-DAP-Seq(GSE60143)/Homer	1e-20	-4.808e+01	0.0000	2440.0	51.46%	362.5	44.63%	motif file (matrix)	svg
195	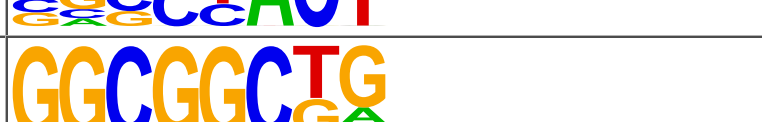	ERF2(AP2EREBP)/colamp-ERF2-DAP-Seq(GSE60143)/Homer	1e-20	-4.776e+01	0.0000	3439.0	72.52%	537.2	66.14%	motif file (matrix)	svg
196	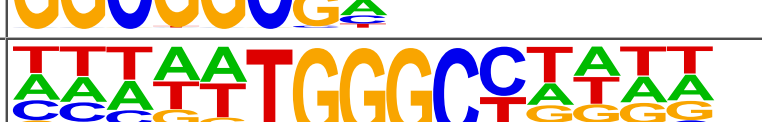	PCF/Arabidopsis-Promoters/Homer	1e-20	-4.753e+01	0.0000	70.0	1.48%	3.2	0.40%	motif file (matrix)	svg
197	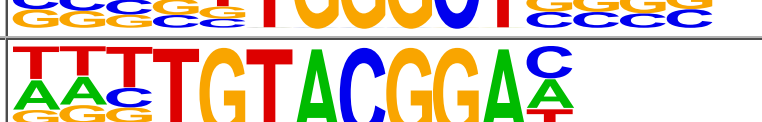	SPL3(SBP)/colamp-SPL3-DAP-Seq(GSE60143)/Homer	1e-20	-4.750e+01	0.0000	41.0	0.86%	0.6	0.07%	motif file (matrix)	svg
198	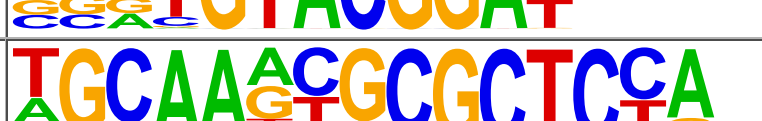	EFL-1(E2F)/cElegans-L1-EFL1-ChIP-Seq(modEncode)/Homer	1e-20	-4.750e+01	0.0000	41.0	0.86%	0.0	0.00%	motif file (matrix)	svg
199	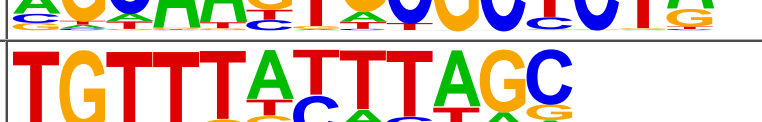	FoxD3(forkhead)/ZebrafishEmbryo-Foxd3.biotin-ChIP-seq(GSE106676)/Homer	1e-20	-4.677e+01	0.0000	378.0	7.97%	39.7	4.88%	motif file (matrix)	svg
200	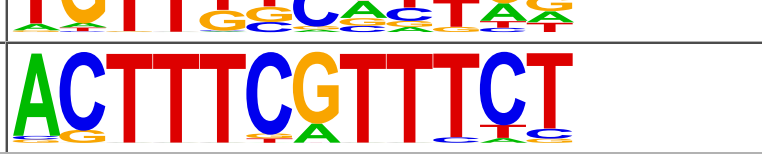	T1ISRE(IRF)/ThioMac-Ifnb-Expression/Homer	1e-20	-4.637e+01	0.0000	56.0	1.18%	3.0	0.37%	motif file (matrix)	svg

201	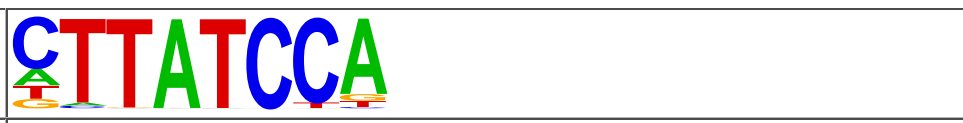	At5g47390(MYBrelated)/col-At5g47390-DAP-Seq(GSE60143)/Homer	1e-19	-4.588e+01	0.0000	599.0	12.63%	70.5	8.68%	motif file (matrix)	svg
202	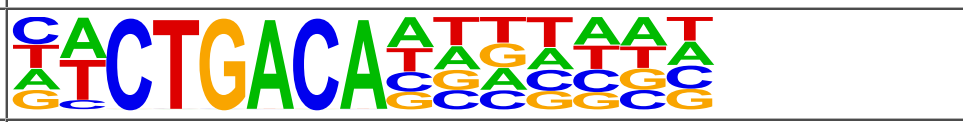	GRF9(GRF)/colamp-GRF9-DAP-Seq(GSE60143)/Homer	1e-19	-4.585e+01	0.0000	406.0	8.56%	43.8	5.39%	motif file (matrix)	svg
203	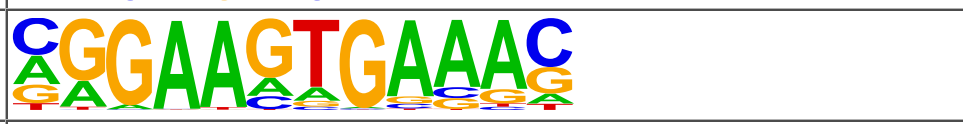	PU.1-IRF(ETS:IRF)/Bcell-PU.1-ChIP-Seq(GSE21512)/Homer	1e-19	-4.570e+01	0.0000	850.0	17.92%	107.3	13.21%	motif file (matrix)	svg
204	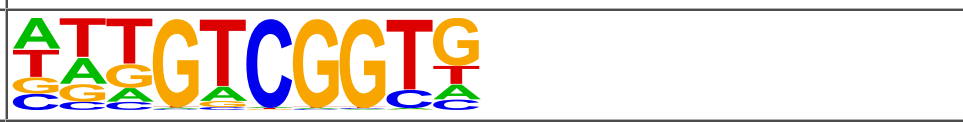	CEJ1(AP2EREBP)/col-CEJ1-DAP-Seq(GSE60143)/Homer	1e-19	-4.568e+01	0.0000	1367.0	28.83%	187.9	23.14%	motif file (matrix)	svg
205	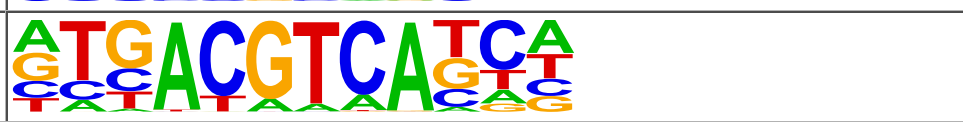	TGA4(bZIP)/colamp-TGA4-DAP-Seq(GSE60143)/Homer	1e-19	-4.508e+01	0.0000	159.0	3.35%	12.0	1.48%	motif file (matrix)	svg
206	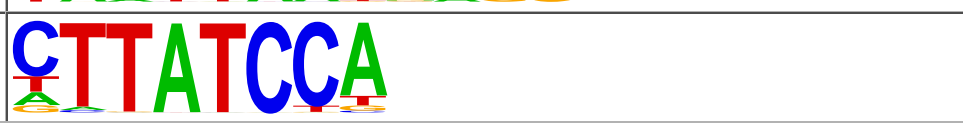	AT5G61620(MYBrelated)/colamp-AT5G61620-DAP-Seq(GSE60143)/Homer	1e-19	-4.502e+01	0.0000	666.0	14.04%	80.1	9.87%	motif file (matrix)	svg
207	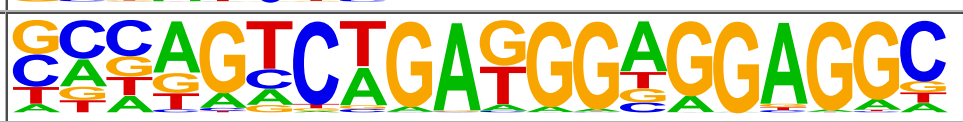	ZSCAN22(Zf)/HEK293-ZSCAN22.GFP-ChIP-Seq(GSE58341)/Homer	1e-19	-4.484e+01	0.0000	91.0	1.92%	5.2	0.65%	motif file (matrix)	svg
208	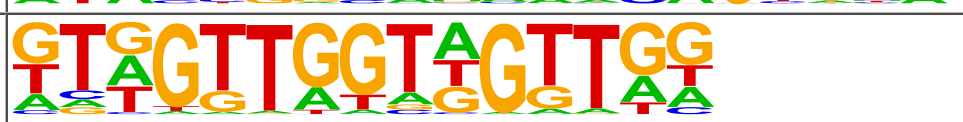	AT1G24250(Orphan)/col-AT1G24250-DAP-Seq(GSE60143)/Homer	1e-19	-4.423e+01	0.0000	149.0	3.14%	11.9	1.47%	motif file (matrix)	svg
209	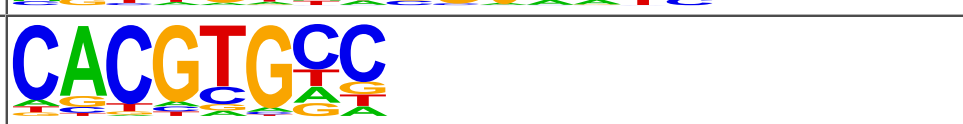	IBL1(bHLH)/Seedling-IBL1-ChIP-Seq(GSE51120)/Homer	1e-19	-4.416e+01	0.0000	2988.0	63.01%	459.7	56.60%	motif file (matrix)	svg
210	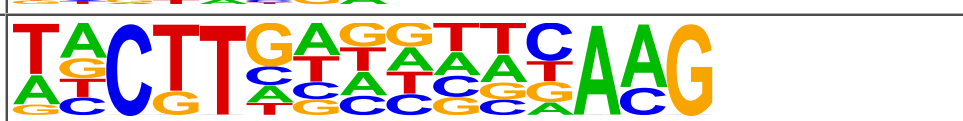	ANAC096(NAC)/colamp-ANAC096-DAP-Seq(GSE60143)/Homer	1e-19	-4.409e+01	0.0000	234.0	4.93%	21.7	2.67%	motif file (matrix)	svg
211	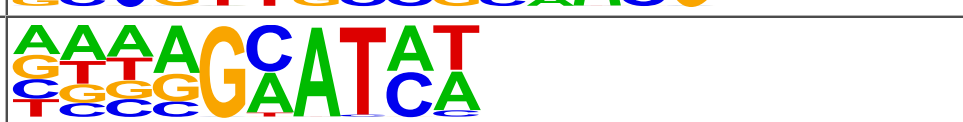	At3g24120(G2like)/col-At3g24120-DAP-Seq(GSE60143)/Homer	1e-18	-4.335e+01	0.0000	1397.0	29.46%	193.0	23.77%	motif file (matrix)	svg
212	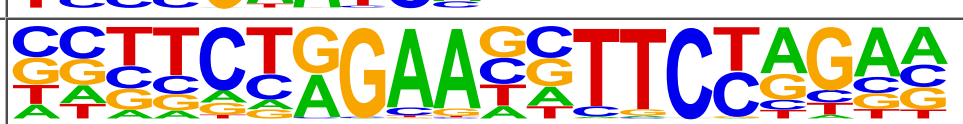	HRE(HSF)/HepG2-HSF1-ChIP-Seq(GSE31477)/Homer	1e-18	-4.316e+01	0.0000	100.0	2.11%	6.7	0.83%	motif file (matrix)	svg
213	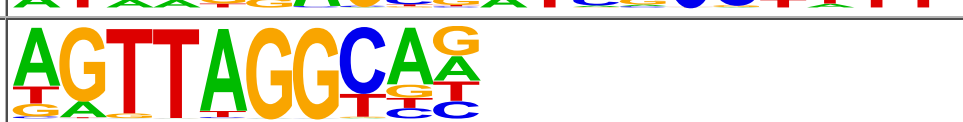	MYB116(MYB)/colamp-MYB116-DAP-Seq(GSE60143)/Homer	1e-18	-4.312e+01	0.0000	311.0	6.56%	31.9	3.93%	motif file (matrix)	svg
214	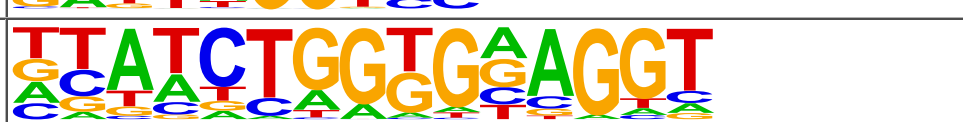	STOP1(C2H2)/colamp-STOP1-DAP-Seq(GSE60143)/Homer	1e-18	-4.292e+01	0.0000	272.0	5.74%	26.9	3.31%	motif file (matrix)	svg
215	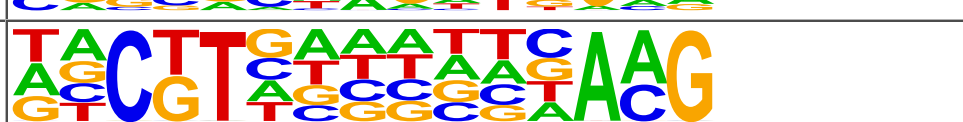	ANAC071(NAC)/col-ANAC071-DAP-Seq(GSE60143)/Homer	1e-18	-4.274e+01	0.0000	295.0	6.22%	29.3	3.61%	motif file (matrix)	svg
216	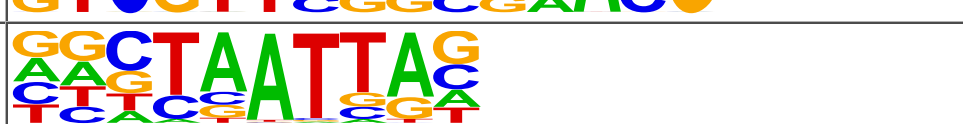	En1(Homeobox)/SUM149-EN1-ChIP-Seq(GSE120957)/Homer	1e-18	-4.233e+01	0.0000	754.0	15.90%	94.5	11.64%	motif file (matrix)	svg
217	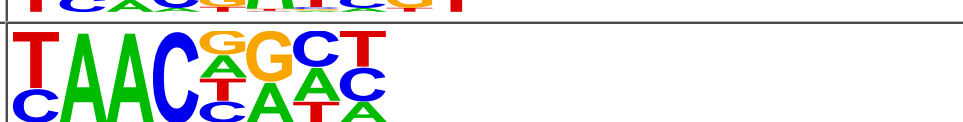	MYB101(MYB)/colamp-MYB101-DAP-Seq(GSE60143)/Homer	1e-18	-4.229e+01	0.0000	1011.0	21.32%	133.7	16.46%	motif file (matrix)	svg
218	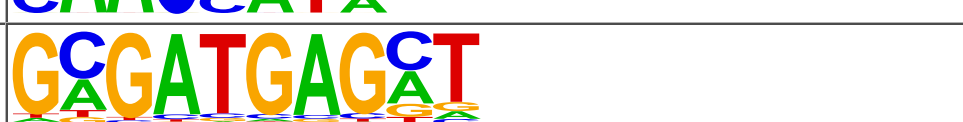	TOD6?/SacCer-Promoters/Homer	1e-18	-4.177e+01	0.0000	181.0	3.82%	15.5	1.91%	motif file (matrix)	svg
219	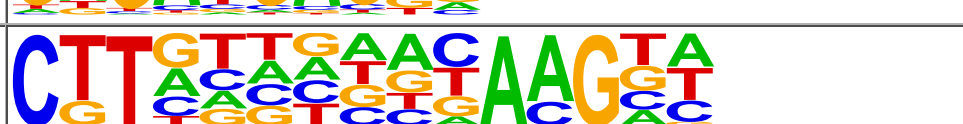	NTM2(NAC)/col-NTM2-DAP-Seq(GSE60143)/Homer	1e-18	-4.170e+01	0.0000	206.0	4.34%	18.9	2.33%	motif file (matrix)	svg
220	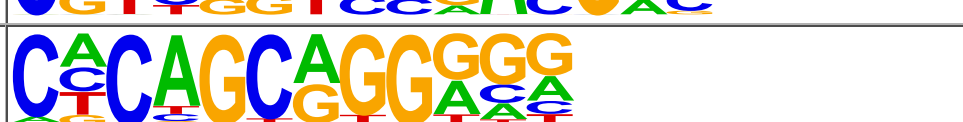	Zic2(Zf)/ESC-Zic2-ChIP-Seq(SRP197560)/Homer	1e-18	-4.146e+01	0.0000	989.0	20.86%	130.0	16.01%	motif file (matrix)	svg
221	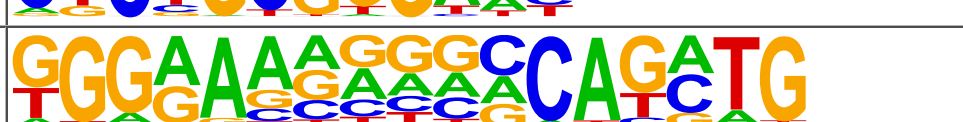	RBPJ:Ebox(?,bHLH)/Panc1-Rbpj1-ChIP-Seq(GSE47459)/Homer	1e-17	-4.141e+01	0.0000	238.0	5.02%	22.9	2.82%	motif file (matrix)	svg
222	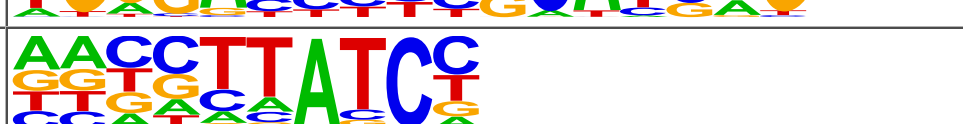	At5g08520(MYBrelated)/colamp-At5g08520-DAP-Seq(GSE60143)/Homer	1e-17	-4.138e+01	0.0000	419.0	8.84%	46.8	5.76%	motif file (matrix)	svg
223	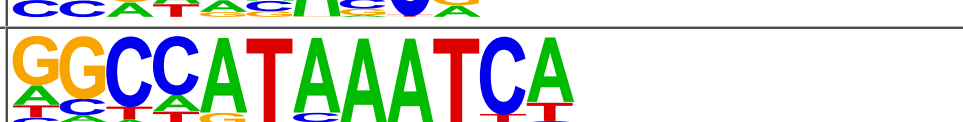	Hoxc9(Homeobox)/Ainv15-Hoxc9-ChIP-Seq(GSE21812)/Homer	1e-17	-4.082e+01	0.0000	145.0	3.06%	11.6	1.43%	motif file (matrix)	svg
224	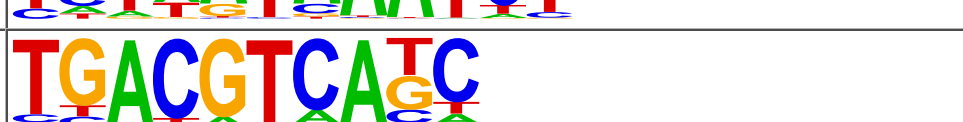	TGA1(bZIP)/colamp-TGA1-DAP-Seq(GSE60143)/Homer	1e-17	-4.019e+01	0.0000	212.0	4.47%	19.1	2.35%	motif file (matrix)	svg
225	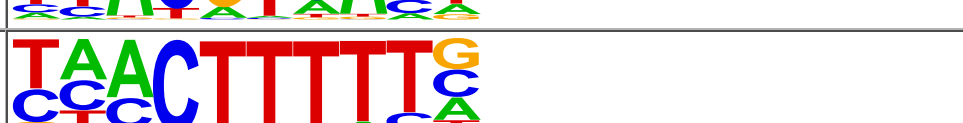	AT3G12130(C3H)/colamp-AT3G12130-DAP-Seq(GSE60143)/Homer	1e-17	-3.934e+01	0.0000	1175.0	24.78%	160.2	19.72%	motif file (matrix)	svg
226	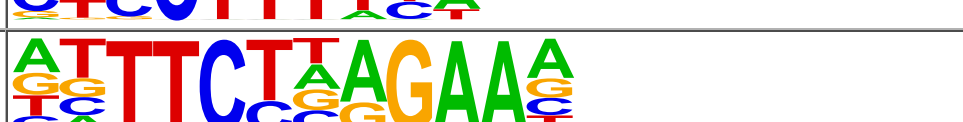	STAT5(Stat)/mCD4+-Stat5-ChIP-Seq(GSE12346)/Homer	1e-17	-3.915e+01	0.0000	143.0	3.02%	11.9	1.46%	motif file (matrix)	svg
227	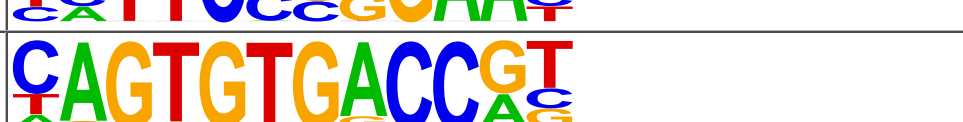	M1BP(Zf)/S2R+-M1BP-ChIP-Seq(GSE49842)/Homer	1e-16	-3.911e+01	0.0000	134.0	2.83%	10.9	1.34%	motif file (matrix)	svg
228	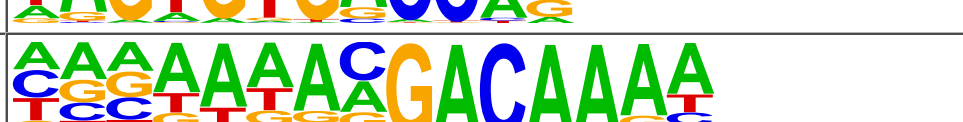	IDD2(C2H2)/colamp-IDD2-DAP-Seq(GSE60143)/Homer	1e-16	-3.808e+01	0.0000	85.0	1.79%	5.9	0.73%	motif file (matrix)	svg
229	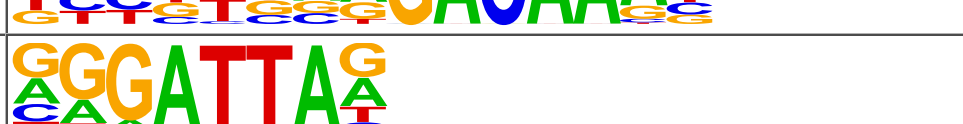	GSC(Homeobox)/FrogEmbryos-GSC-ChIP-Seq(DRA000576)/Homer	1e-16	-3.807e+01	0.0000	490.0	10.33%	57.6	7.09%	motif file (matrix)	svg
230	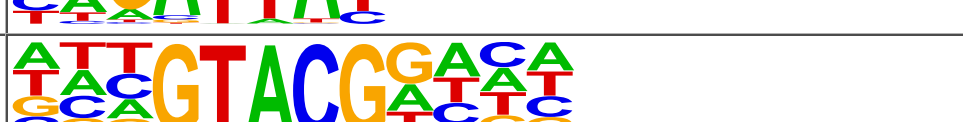	SPL5(SBP)/colamp-SPL5-DAP-Seq(GSE60143)/Homer	1e-16	-3.791e+01	0.0000	361.0	7.61%	39.7	4.88%	motif file (matrix)	svg
231	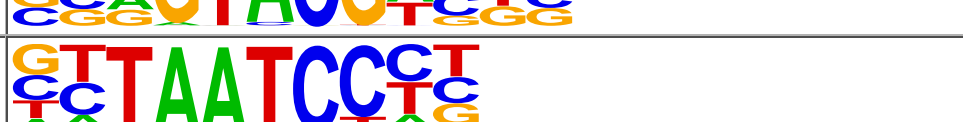	Otx2(Homeobox)/EpiLC-Otx2-ChIP-Seq(GSE56098)/Homer	1e-16	-3.776e+01	0.0000	368.0	7.76%	40.2	4.95%	motif file (matrix)	svg
232	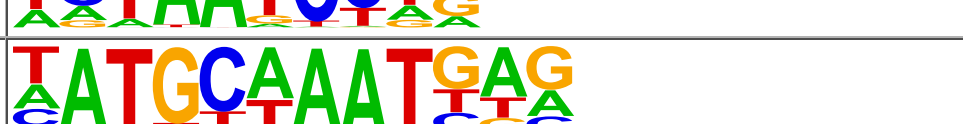	Oct6(POU,Homeobox)/NPC-Pou3f1-ChIP-Seq(GSE35496)/Homer	1e-16	-3.760e+01	0.0000	232.0	4.89%	22.6	2.78%	motif file (matrix)	svg
233	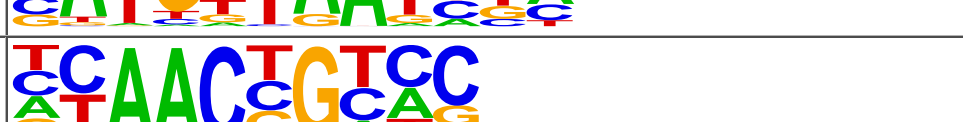	MYB77(MYB)/col-MYB77-DAP-Seq(GSE60143)/Homer	1e-16	-3.700e+01	0.0000	779.0	16.43%	100.6	12.39%	motif file (matrix)	svg
234	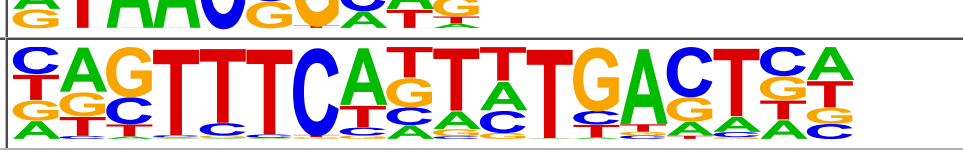	bZIP:IRF(bZIP,IRF)/Th17-BatF-ChIP-Seq(GSE39756)/Homer	1e-15	-3.648e+01	0.0000	284.0	5.99%	29.0	3.57%	motif file (matrix)	svg

235	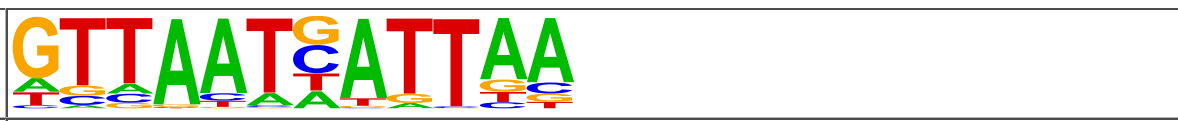	HNF1b(Homeobox)/PDAC-HNF1B-ChIP-Seq(GSE64557)/Homer	1e-15	-3.612e+01	0.0000	35.0	0.74%	1.9	0.24%	motif file (matrix)	svg
236	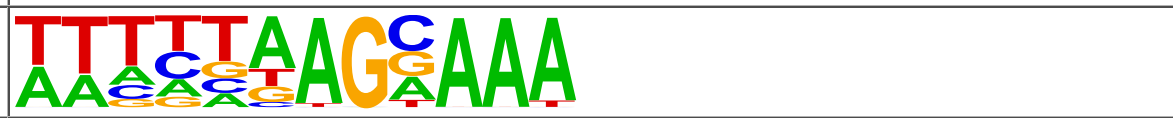	AT5G60130(ABI3VP1)/col-AT5G60130-DAP-Seq(GSE60143)/Homer	1e-15	-3.587e+01	0.0000	802.0	16.91%	104.5	12.87%	motif file (matrix)	svg
237	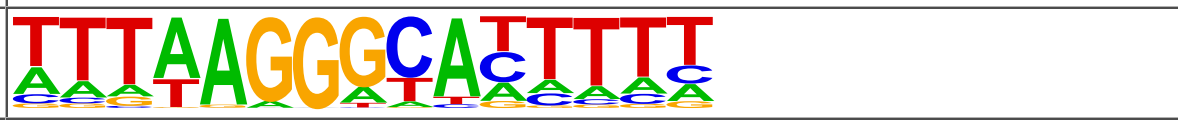	AT2G33550(Trihelix)/colamp-AT2G33550-DAP-Seq(GSE60143)/Homer	1e-15	-3.586e+01	0.0000	769.0	16.22%	99.8	12.29%	motif file (matrix)	svg
238	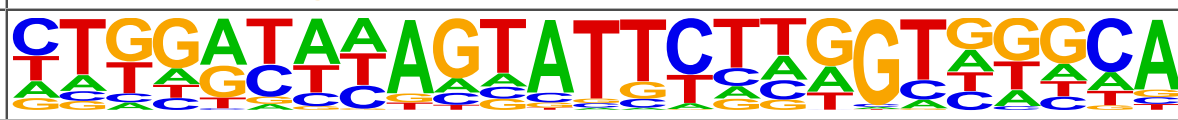	ZNF136(Zf)/HEK293-ZNF136.GFP-ChIP-Seq(GSE58341)/Homer	1e-15	-3.546e+01	0.0000	61.0	1.29%	3.3	0.40%	motif file (matrix)	svg
239	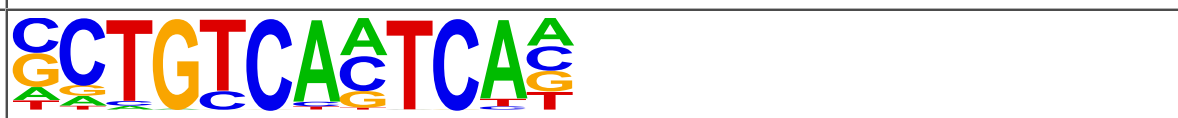	Pbx3(Homeobox)/GM12878-PBX3-ChIP-Seq(GSE32465)/Homer	1e-15	-3.527e+01	0.0000	102.0	2.15%	7.7	0.95%	motif file (matrix)	svg
240	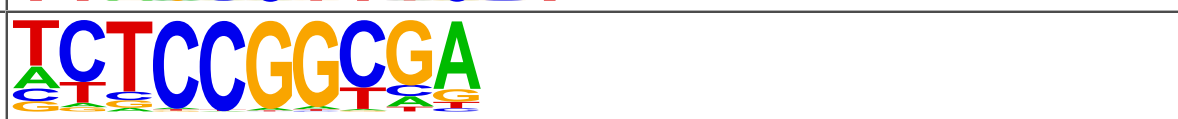	AT3G58630(Trihelix)/col-AT3G58630-DAP-Seq(GSE60143)/Homer	1e-15	-3.514e+01	0.0000	647.0	13.64%	81.5	10.03%	motif file (matrix)	svg
241	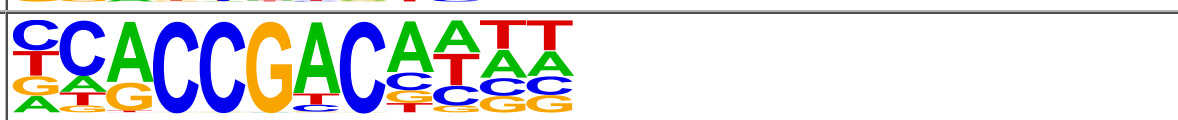	RAP21(AP2EREBP)/colamp-RAP21-DAP-Seq(GSE60143)/Homer	1e-15	-3.505e+01	0.0000	220.0	4.64%	21.2	2.61%	motif file (matrix)	svg
242	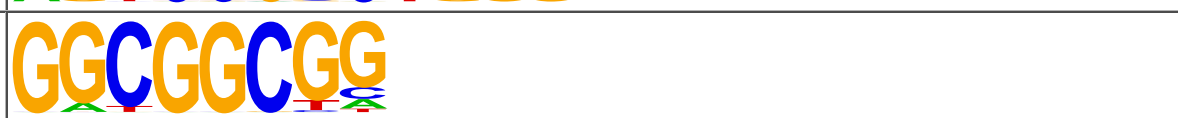	ESE1(AP2EREBP)/col-ESE1-DAP-Seq(GSE60143)/Homer	1e-15	-3.481e+01	0.0000	3504.0	73.89%	558.0	68.70%	motif file (matrix)	svg
243	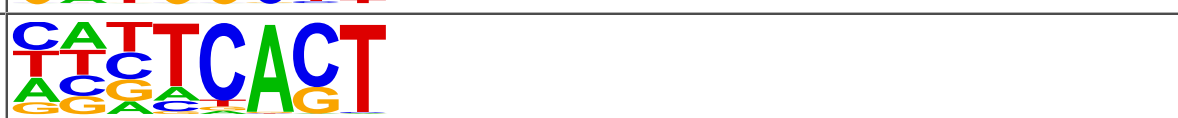	STZ(C2H2)/colamp-STZ-DAP-Seq(GSE60143)/Homer	1e-15	-3.480e+01	0.0000	2698.0	56.90%	415.9	51.20%	motif file (matrix)	svg
244	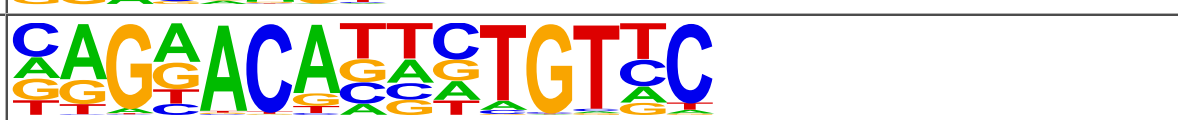	GRE(NR),IR3/RAW264.7-GRE-ChIP-Seq(Unpublished)/Homer	1e-14	-3.434e+01	0.0000	101.0	2.13%	8.0	0.98%	motif file (matrix)	svg
245	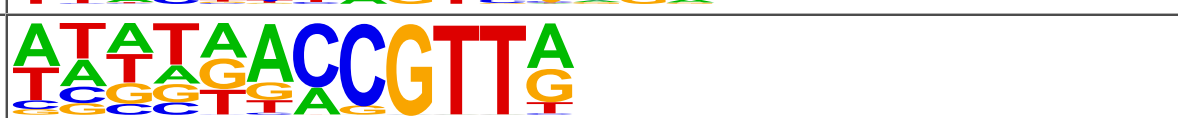	MYB3R1(MYB)/col-MYB3R1-DAP-Seq(GSE60143)/Homer	1e-14	-3.392e+01	0.0000	91.0	1.92%	6.4	0.79%	motif file (matrix)	svg
246	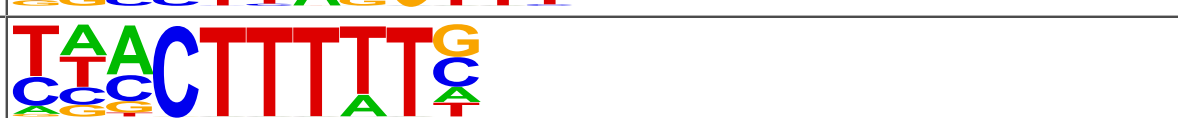	At3g45610(C2C2dof)/col-At3g45610-DAP-Seq(GSE60143)/Homer	1e-14	-3.366e+01	0.0000	854.0	18.01%	113.9	14.03%	motif file (matrix)	svg
247	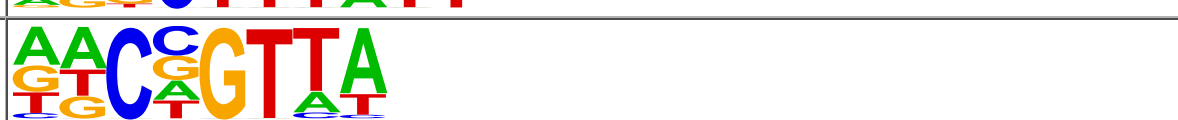	MYB65(MYB)/colamp-MYB65-DAP-Seq(GSE60143)/Homer	1e-14	-3.345e+01	0.0000	465.0	9.81%	55.1	6.78%	motif file (matrix)	svg
248	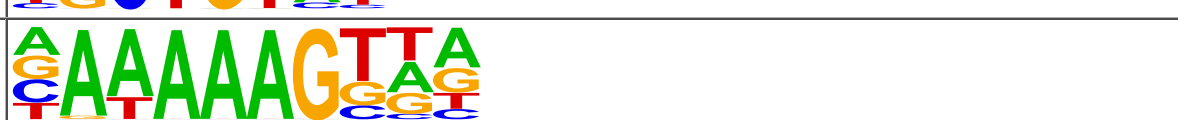	BBX31(Orphan)/col-BBX31-DAP-Seq(GSE60143)/Homer	1e-14	-3.327e+01	0.0000	794.0	16.74%	104.3	12.85%	motif file (matrix)	svg
249	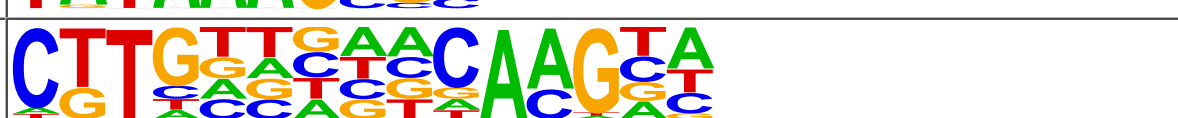	ANAC013(NAC)/col-ANAC013-DAP-Seq(GSE60143)/Homer	1e-14	-3.304e+01	0.0000	177.0	3.73%	16.8	2.07%	motif file (matrix)	svg
250	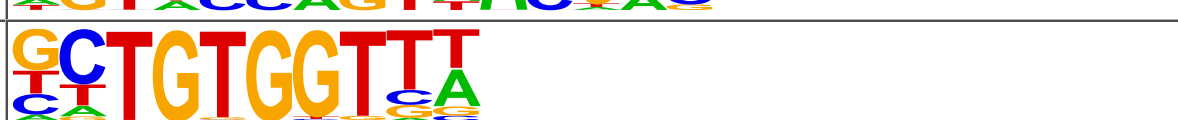	RUNX-AML(Runt)/CD4+-PolII-ChIP-Seq(Barski_et_al.)/Homer	1e-14	-3.280e+01	0.0000	292.0	6.16%	31.6	3.89%	motif file (matrix)	svg
251	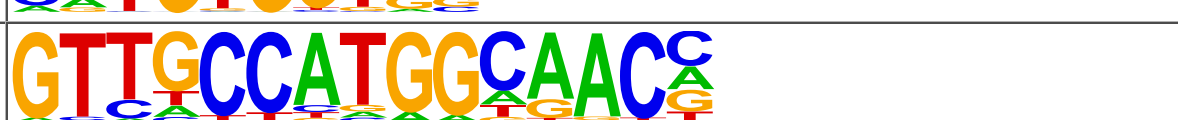	Rfx2(HTH)/LoVo-RFX2-ChIP-Seq(GSE49402)/Homer	1e-14	-3.250e+01	0.0000	99.0	2.09%	8.0	0.98%	motif file (matrix)	svg
252	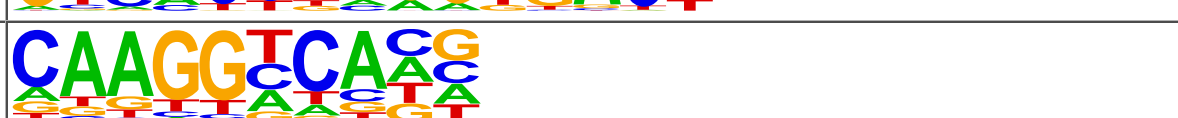	SF1(NR)/H295R-Nr5a1-ChIP-Seq(GSE44220)/Homer	1e-14	-3.245e+01	0.0000	269.0	5.67%	28.1	3.46%	motif file (matrix)	svg
253	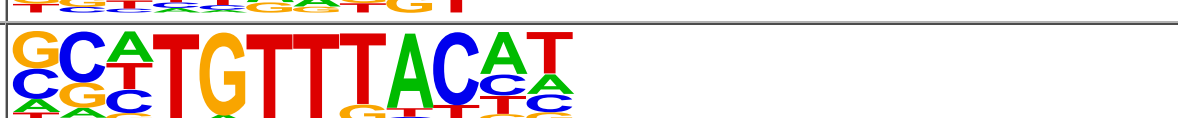	FOXK2(Forkhead)/U2OS-FOXK2-ChIP-Seq(E-MTAB-2204)/Homer	1e-13	-3.223e+01	0.0000	246.0	5.19%	26.0	3.20%	motif file (matrix)	svg
254	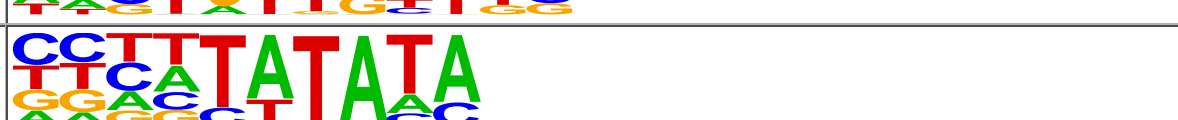	TATA-box/SacCer-Promoters/Homer	1e-13	-3.139e+01	0.0000	252.0	5.31%	26.9	3.31%	motif file (matrix)	svg
255	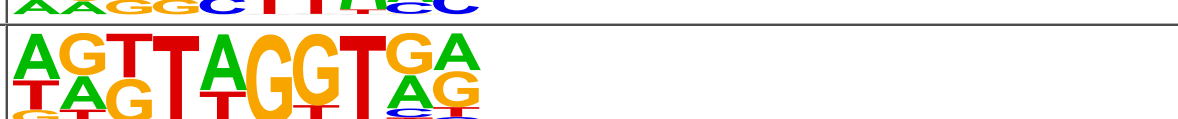	MYB49(MYB)/col-MYB49-DAP-Seq(GSE60143)/Homer	1e-13	-3.135e+01	0.0000	432.0	9.11%	51.8	6.38%	motif file (matrix)	svg
256	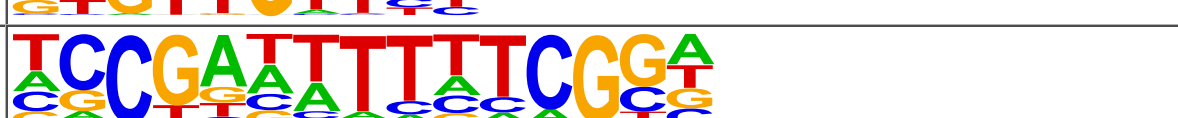	LBD2(LOBAS2)/colamp-LBD2-DAP-Seq(GSE60143)/Homer	1e-13	-3.133e+01	0.0000	340.0	7.17%	39.0	4.80%	motif file (matrix)	svg
257	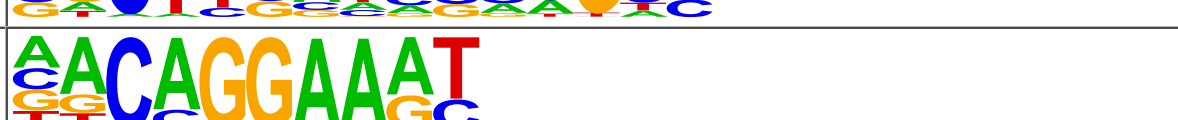	EWS:FLI1-fusion(ETS)/SK_N_MC-EWS:FLI1-ChIP-Seq(SRA014231)/Homer	1e-13	-3.112e+01	0.0000	473.0	9.97%	57.8	7.11%	motif file (matrix)	svg
258	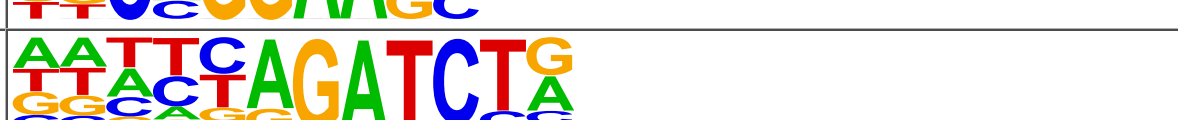	GATA11(C2C2gata)/col-GATA11-DAP-Seq(GSE60143)/Homer	1e-13	-3.112e+01	0.0000	190.0	4.01%	18.6	2.29%	motif file (matrix)	svg
259	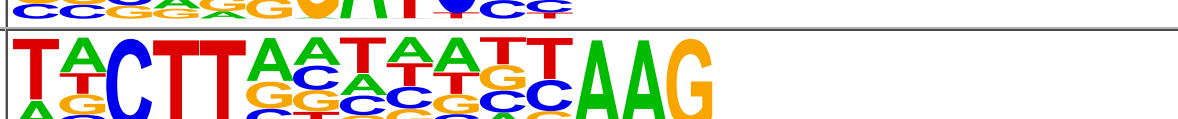	ANAC062(NAC)/colamp-ANAC062-DAP-Seq(GSE60143)/Homer	1e-13	-3.094e+01	0.0000	124.0	2.61%	10.3	1.27%	motif file (matrix)	svg
260	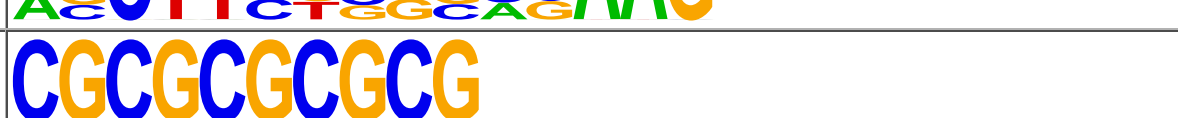	SeqBias: CG-repeat	1e-13	-3.091e+01	0.0000	3916.0	82.58%	635.1	78.19%	motif file (matrix)	svg
261	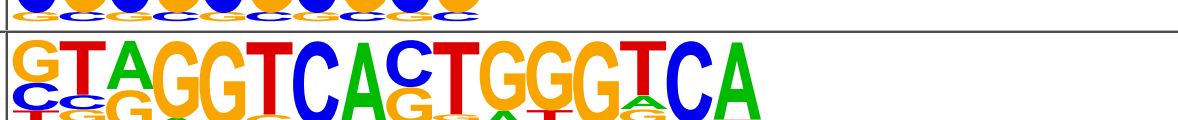	Reverb(NR),DR2/RAW-Reverba.biotin-ChIP-Seq(GSE45914)/Homer	1e-13	-3.085e+01	0.0000	68.0	1.43%	4.6	0.56%	motif file (matrix)	svg
262	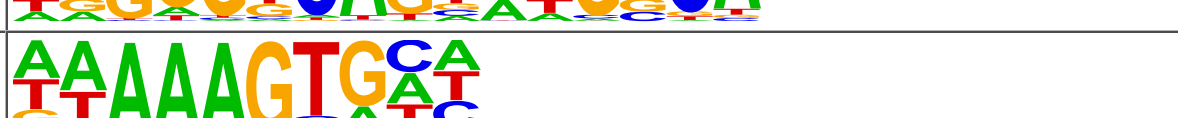	AT1G69570(C2C2dof)/col-AT1G69570-DAP-Seq(GSE60143)/Homer	1e-13	-3.044e+01	0.0000	553.0	11.66%	69.7	8.58%	motif file (matrix)	svg
263	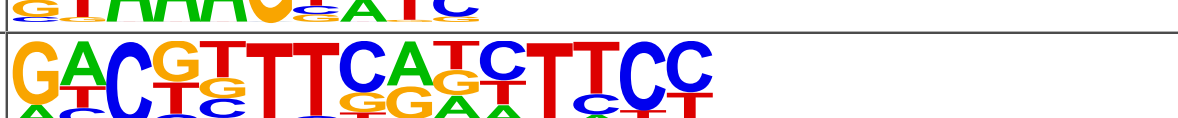	RKD2(RWPRK)/colamp-RKD2-DAP-Seq(GSE60143)/Homer	1e-13	-3.022e+01	0.0000	450.0	9.49%	54.5	6.71%	motif file (matrix)	svg
264	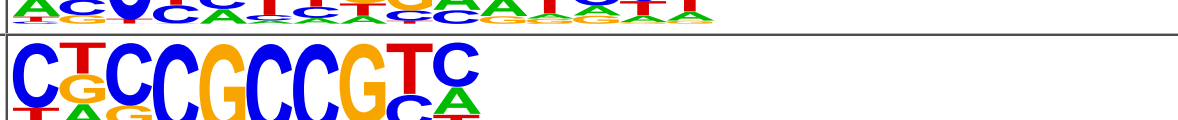	LEP(AP2EREBP)/col-LEP-DAP-Seq(GSE60143)/Homer	1e-13	-3.022e+01	0.0000	2544.0	53.65%	392.2	48.29%	motif file (matrix)	svg
265	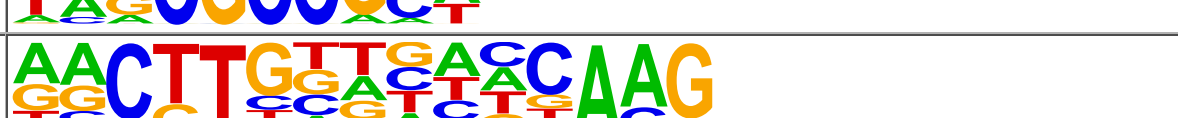	ANAC103(NAC)/col-ANAC103-DAP-Seq(GSE60143)/Homer	1e-12	-2.982e+01	0.0000	180.0	3.80%	17.5	2.15%	motif file (matrix)	svg
266	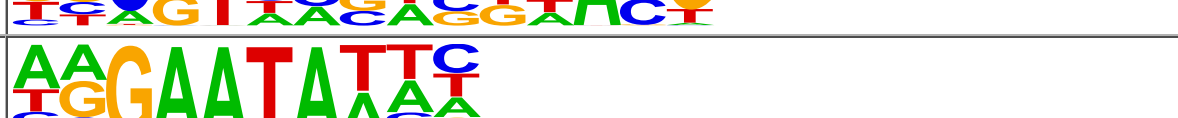	KANADI1(Myb)/Seedling-KAN1-ChIP-Seq(GSE48081)/Homer	1e-12	-2.901e+01	0.0000	488.0	10.29%	60.6	7.46%	motif file (matrix)	svg
267	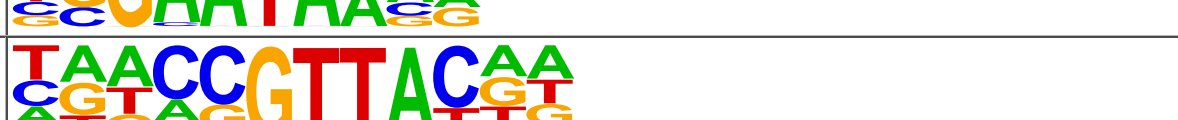	MYB119(MYB)/colamp-MYB119-DAP-Seq(GSE60143)/Homer	1e-12	-2.895e+01	0.0000	95.0	2.00%	7.9	0.97%	motif file (matrix)	svg
268	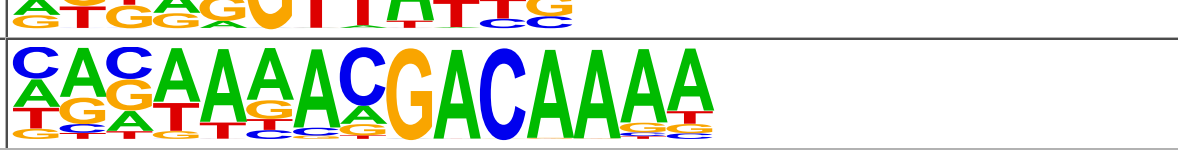	NUC(C2H2)/col-NUC-DAP-Seq(GSE60143)/Homer	1e-12	-2.869e+01	0.0000	66.0	1.39%	4.1	0.50%	motif file (matrix)	svg

269	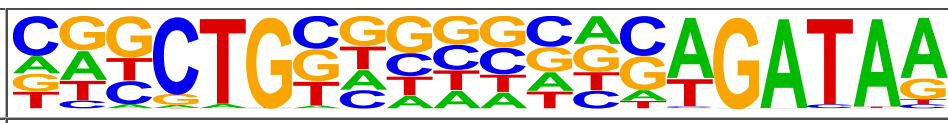	GATA:SCL(Zf,bHLH)/Ter119-SCL-ChIP-Seq(GSE18720)/Homer	1e-12	-2.866e+01	0.0000	44.0	0.93%	2.5	0.31%	motif file (matrix)	svg
270	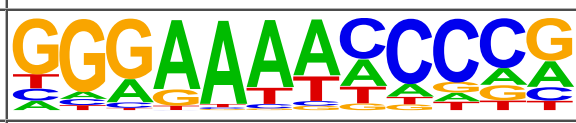	Dorsal(RHD)/Embryo-dl-ChIP-Seq(GSE65441)/Homer	1e-12	-2.864e+01	0.0000	121.0	2.55%	10.6	1.31%	motif file (matrix)	svg
271	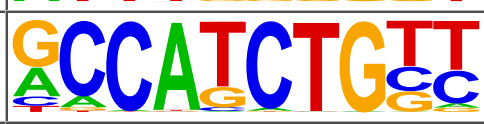	NeuroD1(bHLH)/Islet-NeuroD1-ChIP-Seq(GSE30298)/Homer	1e-12	-2.863e+01	0.0000	568.0	11.98%	72.9	8.98%	motif file (matrix)	svg
272	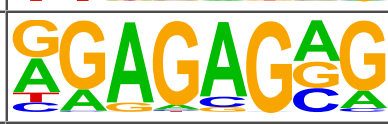	Trl(Zf)/S2-GAGAfactor-ChIP-Seq(GSE40646)/Homer	1e-12	-2.853e+01	0.0000	2947.0	62.15%	463.2	57.03%	motif file (matrix)	svg
273	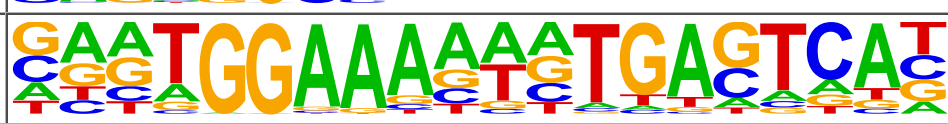	NFAT:AP1(RHD,bZIP)/Jurkat-NFATC1-ChIP-Seq(Jolma_et_al.)/Homer	1e-12	-2.830e+01	0.0000	129.0	2.72%	11.6	1.42%	motif file (matrix)	svg
274	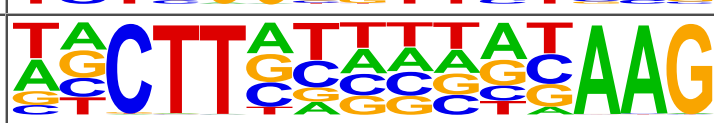	ANAC004(NAC)/colamp-ANAC004-DAP-Seq(GSE60143)/Homer	1e-12	-2.830e+01	0.0000	129.0	2.72%	11.9	1.46%	motif file (matrix)	svg
275	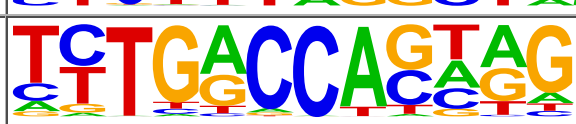	Bcl11a(Zf)/HSPC-BCL11A-ChIP-Seq(GSE104676)/Homer	1e-12	-2.804e+01	0.0000	375.0	7.91%	44.6	5.50%	motif file (matrix)	svg
276	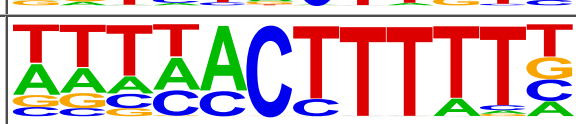	OBP4(C2C2dof)/col-OBP4-DAP-Seq(GSE60143)/Homer	1e-12	-2.792e+01	0.0000	672.0	14.17%	88.8	10.93%	motif file (matrix)	svg
277	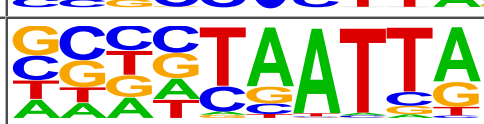	DLX1(Homeobox)/BasalGanglia-Dlx1-ChIP-seq(GSE124936)/Homer	1e-12	-2.774e+01	0.0000	552.0	11.64%	70.1	8.63%	motif file (matrix)	svg
278	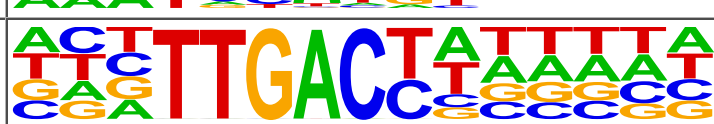	WRKY18(WRKY)/col-WRKY18-DAP-Seq(GSE60143)/Homer	1e-12	-2.766e+01	0.0000	625.0	13.18%	81.3	10.01%	motif file (matrix)	svg
279	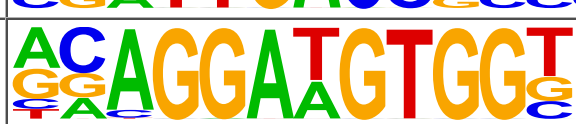	ETS:RUNX(ETS,Runt)/Jurkat-RUNX1-ChIP-Seq(GSE17954)/Homer	1e-12	-2.764e+01	0.0000	65.0	1.37%	4.9	0.61%	motif file (matrix)	svg
280	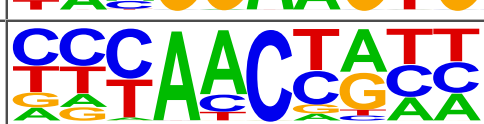	ATY13(MYB)/col-ATY13-DAP-Seq(GSE60143)/Homer	1e-11	-2.757e+01	0.0000	1892.0	39.90%	284.3	35.00%	motif file (matrix)	svg
281	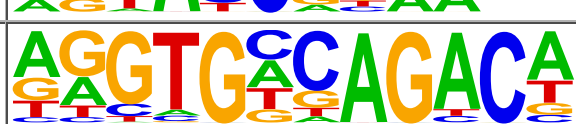	Tbox:Smad(T-box,MAD)/ESCd5-Smad2_3-ChIP-Seq(GSE29422)/Homer	1e-11	-2.755e+01	0.0000	111.0	2.34%	9.0	1.11%	motif file (matrix)	svg
282	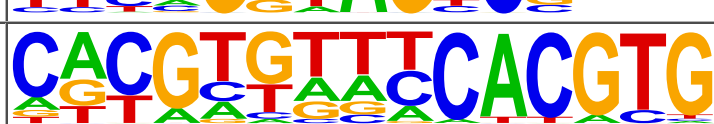	bHLH18(bHLH)/col-bHLH18-DAP-Seq(GSE60143)/Homer	1e-11	-2.741e+01	0.0000	30.0	0.63%	0.2	0.02%	motif file (matrix)	svg
283	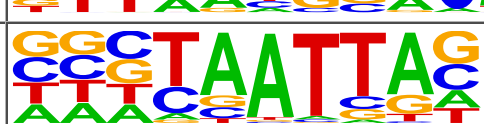	DLX2(Homeobox)/BasalGanglia-Dlx2-ChIP-seq(GSE124936)/Homer	1e-11	-2.707e+01	0.0000	590.0	12.44%	76.2	9.38%	motif file (matrix)	svg
284	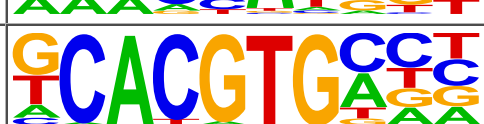	bHLHE40(bHLH)/HepG2-BHLHE40-ChIP-Seq(GSE31477)/Homer	1e-11	-2.681e+01	0.0000	365.0	7.70%	43.7	5.38%	motif file (matrix)	svg
285	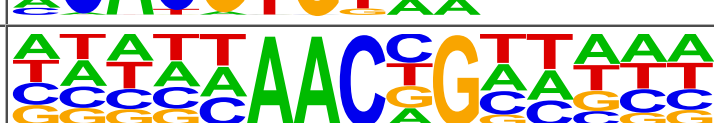	MYB73(MYB)/col-MYB73-DAP-Seq(GSE60143)/Homer	1e-11	-2.676e+01	0.0000	714.0	15.06%	95.8	11.80%	motif file (matrix)	svg
286	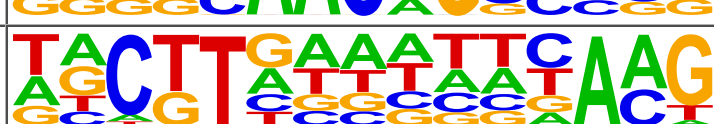	NST1(NAC)/colamp-NST1-DAP-Seq(GSE60143)/Homer	1e-11	-2.672e+01	0.0000	265.0	5.59%	29.2	3.60%	motif file (matrix)	svg
287	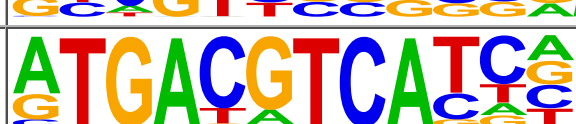	JunD(bZIP)/K562-JunD-ChIP-Seq/Homer	1e-11	-2.660e+01	0.0000	64.0	1.35%	4.5	0.55%	motif file (matrix)	svg
288	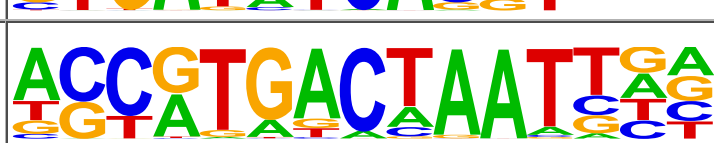	PAX3:FKHR-fusion(Paired,Homeobox)/Rh4-PAX3:FKHR-ChIP-Seq(GSE19063)/Homer	1e-11	-2.652e+01	0.0000	101.0	2.13%	8.9	1.09%	motif file (matrix)	svg
289	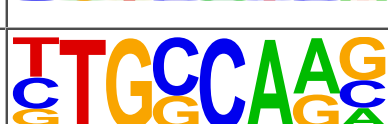	NF1-halfsite(CTF)/LNCaP-NF1-ChIP-Seq(Unpublished)/Homer	1e-11	-2.609e+01	0.0000	1176.0	24.80%	168.1	20.70%	motif file (matrix)	svg
290	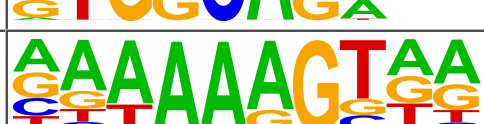	AT5G63260(C3H)/col-AT5G63260-DAP-Seq(GSE60143)/Homer	1e-11	-2.608e+01	0.0000	1038.0	21.89%	146.0	17.98%	motif file (matrix)	svg
291	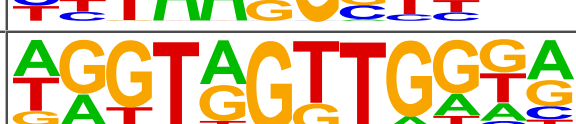	MYB94(MYB)/col-MYB94-DAP-Seq(GSE60143)/Homer	1e-11	-2.606e+01	0.0000	249.0	5.25%	27.8	3.42%	motif file (matrix)	svg
292	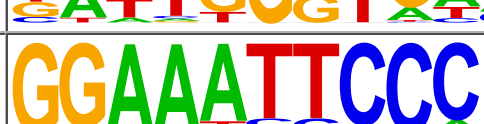	NFkB-p65-Rel(RHD)/ThioMac-LPS-Expression(GSE23622)/Homer	1e-11	-2.576e+01	0.0000	29.0	0.61%	0.5	0.06%	motif file (matrix)	svg
293	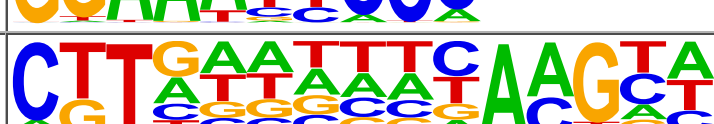	VND1(NAC)/col-VND1-DAP-Seq(GSE60143)/Homer	1e-11	-2.544e+01	0.0000	255.0	5.38%	28.8	3.54%	motif file (matrix)	svg
294	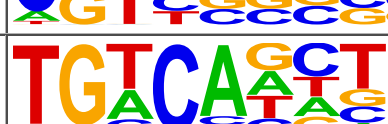	Tgif2(Homeobox)/mES-Tgif2-ChIP-Seq(GSE55404)/Homer	1e-11	-2.541e+01	0.0000	1616.0	34.08%	240.6	29.63%	motif file (matrix)	svg
295	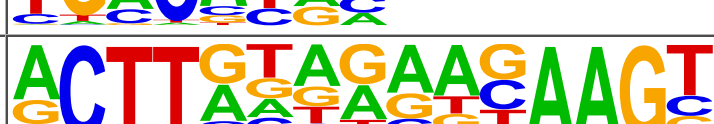	NTM1(NAC)/col-NTM1-DAP-Seq(GSE60143)/Homer	1e-11	-2.540e+01	0.0000	188.0	3.96%	19.2	2.36%	motif file (matrix)	svg
296	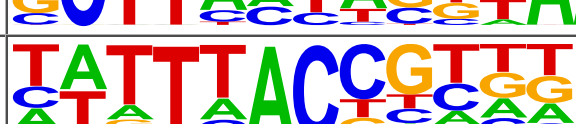	EMB1789(C3H)/col-EMB1789-DAP-Seq(GSE60143)/Homer	1e-10	-2.532e+01	0.0000	149.0	3.14%	14.2	1.74%	motif file (matrix)	svg
297	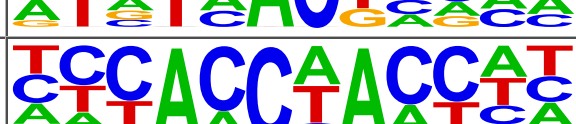	MYB13(MYB)/col-MYB13-DAP-Seq(GSE60143)/Homer	1e-10	-2.521e+01	0.0000	361.0	7.61%	43.7	5.38%	motif file (matrix)	svg
298	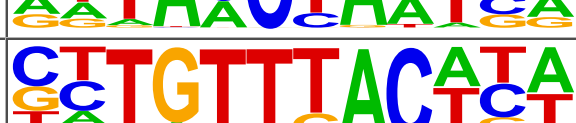	Foxa2(Forkhead)/Liver-Foxa2-ChIP-Seq(GSE25694)/Homer	1e-10	-2.497e+01	0.0000	290.0	6.12%	33.2	4.09%	motif file (matrix)	svg
299	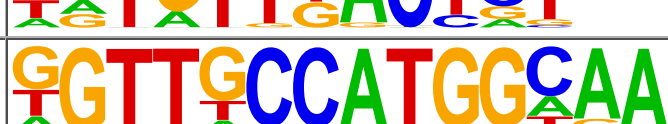	X-box(HTH)/NPC-H3K4me1-ChIP-Seq(GSE16256)/Homer	1e-10	-2.493e+01	0.0000	72.0	1.52%	5.1	0.63%	motif file (matrix)	svg
300	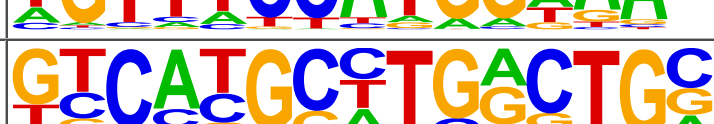	Pax8(Paired,Homeobox)/Thyroid-Pax8-ChIP-Seq(GSE26938)/Homer	1e-10	-2.442e+01	0.0000	359.0	7.57%	43.9	5.40%	motif file (matrix)	svg
301	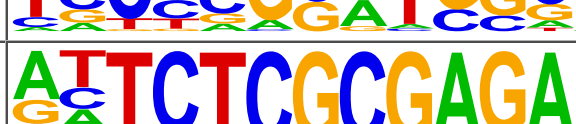	GFX(?)/Promoter/Homer	1e-10	-2.414e+01	0.0000	28.0	0.59%	0.0	0.00%	motif file (matrix)	svg
302	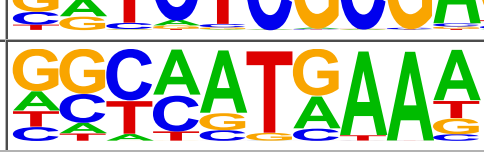	Hoxd10(Homeobox)/ChickenMSG-Hoxd10.Flag-ChIP-Seq(GSE86088)/Homer	1e-10	-2.390e+01	0.0000	507.0	10.69%	65.1	8.02%	motif file (matrix)	svg

303	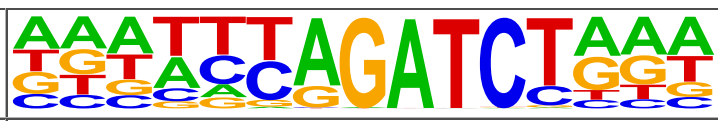	GATA1(C2C2gata)/colamp-GATA1-DAP-Seq(GSE60143)/Homer	1e-10	-2.389e+01	0.0000	170.0	3.58%	17.3	2.13%	motif file (matrix)	svg
304	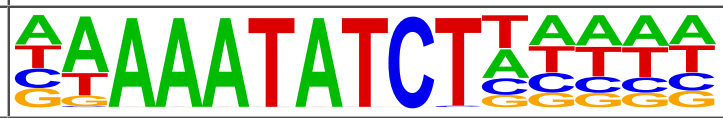	LCL1(MYBrelated)/colamp-LCL1-DAP-Seq(GSE60143)/Homer	1e-10	-2.385e+01	0.0000	80.0	1.69%	6.8	0.83%	motif file (matrix)	svg
305	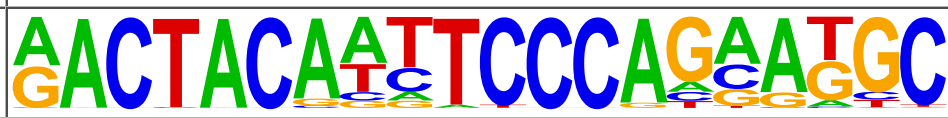	GFY-Staf(?,Zf)/Promoter/Homer	1e-10	-2.344e+01	0.0000	40.0	0.84%	2.7	0.34%	motif file (matrix)	svg
306	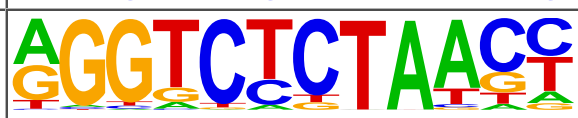	PRDM14(Zf)/H1-PRDM14-ChIP-Seq(GSE22767)/Homer	1e-10	-2.340e+01	0.0000	138.0	2.91%	13.0	1.60%	motif file (matrix)	svg
307	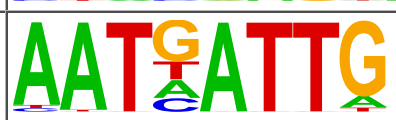	ATHB5(HB)/colamp-ATHB5-DAP-Seq(GSE60143)/Homer	1e-10	-2.328e+01	0.0000	279.0	5.88%	32.8	4.04%	motif file (matrix)	svg
308	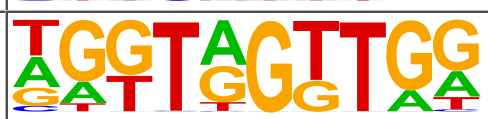	MYB96(MYB)/colamp-MYB96-DAP-Seq(GSE60143)/Homer	1e-10	-2.305e+01	0.0000	511.0	10.78%	66.1	8.13%	motif file (matrix)	svg
309	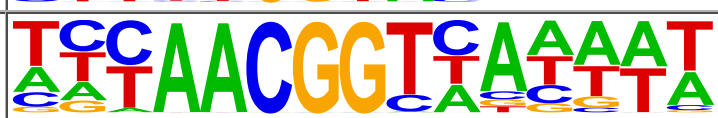	MYB3R5(MYB)/col-MYB3R5-DAP-Seq(GSE60143)/Homer	1e-9	-2.256e+01	0.0000	27.0	0.57%	0.6	0.07%	motif file (matrix)	svg
310	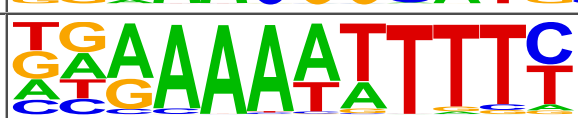	SFP1/SacCer-Promoters/Homer	1e-9	-2.256e+01	0.0000	27.0	0.57%	0.1	0.01%	motif file (matrix)	svg
311	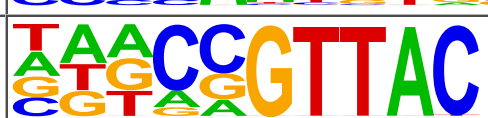	MYB98(MYB)/col-MYB98-DAP-Seq(GSE60143)/Homer	1e-9	-2.237e+01	0.0000	104.0	2.19%	9.9	1.21%	motif file (matrix)	svg
312	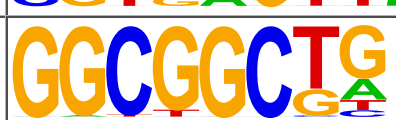	AT5G23930(mTERF)/col-AT5G23930-DAP-Seq(GSE60143)/Homer	1e-9	-2.206e+01	0.0000	3761.0	79.31%	613.2	75.50%	motif file (matrix)	svg
313	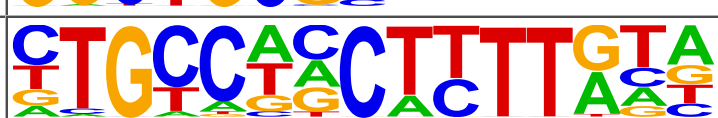	ZNF7(Zf)/HepG2-ZNF7.Flag-ChIP-Seq(Encode)/Homer	1e-9	-2.199e+01	0.0000	283.0	5.97%	33.1	4.08%	motif file (matrix)	svg
314	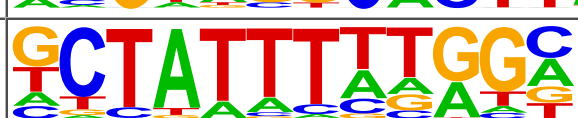	Mef2b(MADS)/HEK293-Mef2b.V5-ChIP-Seq(GSE67450)/Homer	1e-9	-2.197e+01	0.0000	311.0	6.56%	37.7	4.64%	motif file (matrix)	svg
315	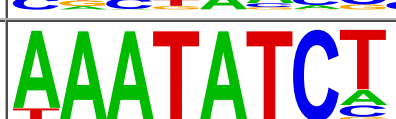	RVE1(MYBrelated)/col-RVE1-DAP-Seq(GSE60143)/Homer	1e-9	-2.188e+01	0.0000	366.0	7.72%	45.4	5.59%	motif file (matrix)	svg
316	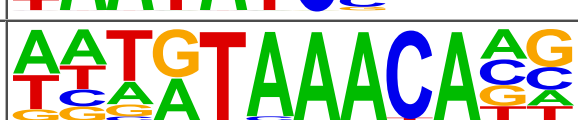	FoxL2(Forkhead)/Ovary-FoxL2-ChIP-Seq(GSE60858)/Homer	1e-9	-2.177e+01	0.0000	352.0	7.42%	43.5	5.35%	motif file (matrix)	svg
317	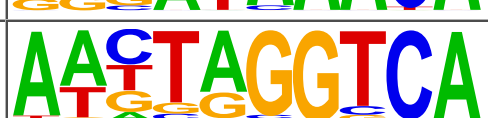	RORgt(NR)/EL4-RORgt.Flag-ChIP-Seq(GSE56019)/Homer	1e-9	-2.146e+01	0.0000	49.0	1.03%	3.2	0.40%	motif file (matrix)	svg
318	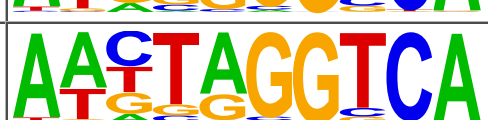	RORgt(NR)/EL4-RORgt.Flag-ChIP-Seq(GSE56019)/Homer	1e-9	-2.146e+01	0.0000	49.0	1.03%	3.2	0.40%	motif file (matrix)	svg
319	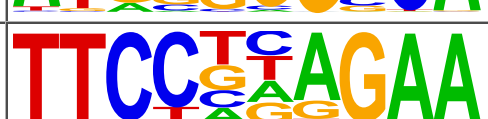	STAT6(Stat)/Macrophage-Stat6-ChIP-Seq(GSE38377)/Homer	1e-9	-2.139e+01	0.0000	173.0	3.65%	18.3	2.26%	motif file (matrix)	svg
320	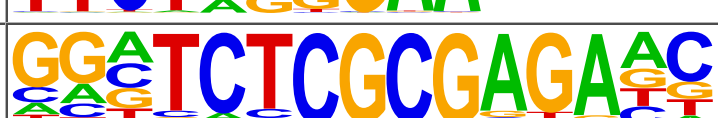	ZBTB33(Zf)/GM12878-ZBTB33-ChIP-Seq(GSE32465)/Homer	1e-9	-2.114e+01	0.0000	165.0	3.48%	17.8	2.19%	motif file (matrix)	svg
321	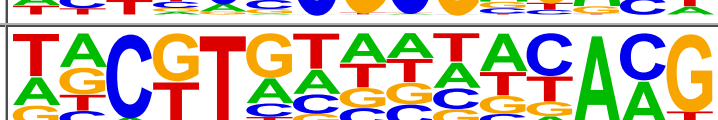	CUC3(NAC)/col-CUC3-DAP-Seq(GSE60143)/Homer	1e-9	-2.110e+01	0.0000	142.0	2.99%	14.8	1.82%	motif file (matrix)	svg
322	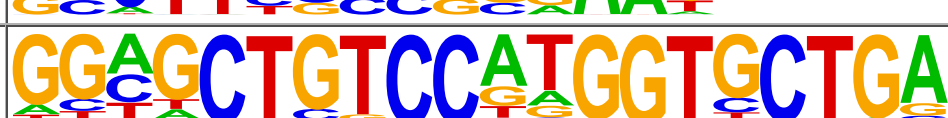	REST-NRSF(Zf)/Jurkat-NRSF-ChIP-Seq/Homer	1e-9	-2.102e+01	0.0000	26.0	0.55%	0.2	0.03%	motif file (matrix)	svg
323	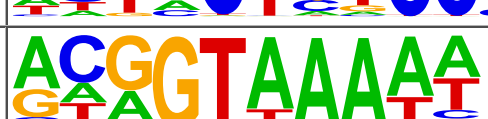	AT1G76880(Trihelix)/col-AT1G76880-DAP-Seq(GSE60143)/Homer	1e-9	-2.097e+01	0.0000	134.0	2.83%	13.3	1.63%	motif file (matrix)	svg
324	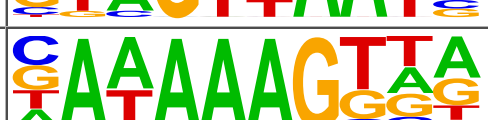	dof43(C2C2dof)/colamp-dof43-DAP-Seq(GSE60143)/Homer	1e-9	-2.097e+01	0.0000	719.0	15.16%	99.2	12.21%	motif file (matrix)	svg
325	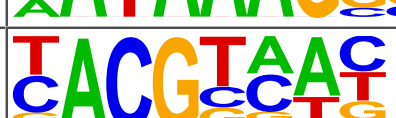	ATAF1(NAC)/col-ATAF1-DAP-Seq(GSE60143)/Homer	1e-9	-2.097e+01	0.0000	2026.0	42.72%	312.4	38.47%	motif file (matrix)	svg
326	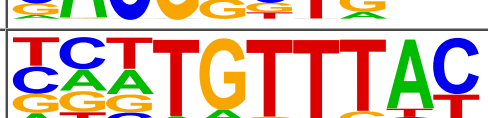	FOXK1(Forkhead)/HEK293-FOXK1-ChIP-Seq(GSE51673)/Homer	1e-9	-2.094e+01	0.0000	431.0	9.09%	55.9	6.88%	motif file (matrix)	svg
327	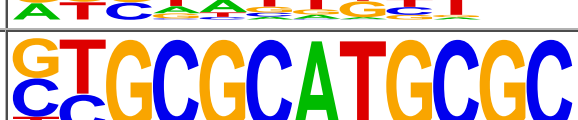	NRF(NRF)/Promoter/Homer	1e-8	-2.053e+01	0.0000	640.0	13.50%	87.1	10.72%	motif file (matrix)	svg
328	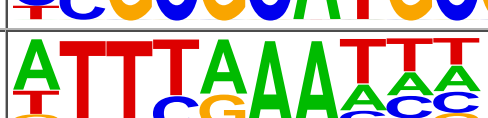	SOL1(CPP)/colamp-SOL1-DAP-Seq(GSE60143)/Homer	1e-8	-2.018e+01	0.0000	442.0	9.32%	57.4	7.07%	motif file (matrix)	svg
329	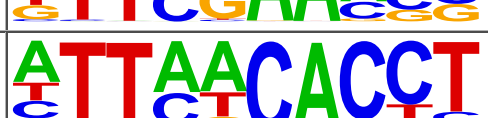	Eomes(T-box)/H9-Eomes-ChIP-Seq(GSE26097)/Homer	1e-8	-2.007e+01	0.0000	868.0	18.30%	123.6	15.22%	motif file (matrix)	svg
330	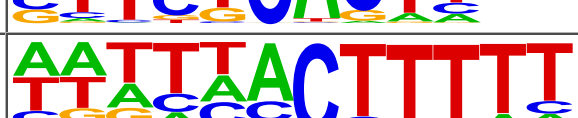	DAG2(C2C2dof)/col-DAG2-DAP-Seq(GSE60143)/Homer	1e-8	-1.995e+01	0.0000	715.0	15.08%	99.2	12.21%	motif file (matrix)	svg
331	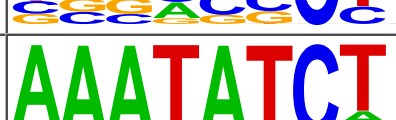	At4g01280(MYBrelated)/colamp-At4g01280-DAP-Seq(GSE60143)/Homer	1e-8	-1.961e+01	0.0000	291.0	6.14%	35.9	4.41%	motif file (matrix)	svg
332	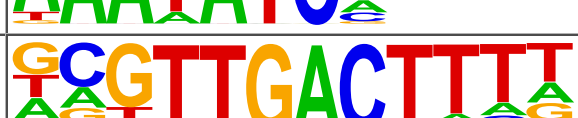	WRKY21(WRKY)/colamp-WRKY21-DAP-Seq(GSE60143)/Homer	1e-8	-1.951e+01	0.0000	25.0	0.53%	1.1	0.13%	motif file (matrix)	svg
333	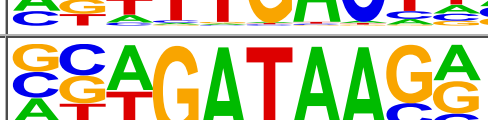	Gata4(Zf)/Heart-Gata4-ChIP-Seq(GSE35151)/Homer	1e-8	-1.949e+01	0.0000	366.0	7.72%	46.5	5.72%	motif file (matrix)	svg
334	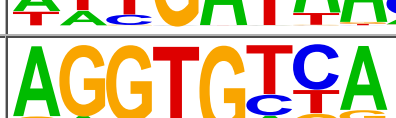	Tbx5(T-box)/HL1-Tbx5.biotin-ChIP-Seq(GSE21529)/Homer	1e-8	-1.940e+01	0.0000	1922.0	40.53%	296.5	36.51%	motif file (matrix)	svg
335	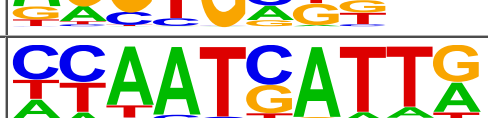	HAT2(Homeobox)/colamp-HAT2-DAP-Seq(GSE60143)/Homer	1e-8	-1.922e+01	0.0000	241.0	5.08%	28.6	3.53%	motif file (matrix)	svg
336	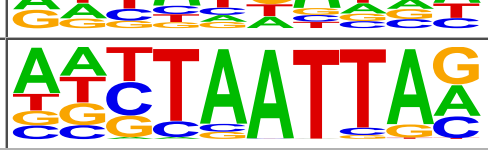	Lhx3(Homeobox)/Neuron-Lhx3-ChIP-Seq(GSE31456)/Homer	1e-8	-1.916e+01	0.0000	609.0	12.84%	84.0	10.34%	motif file (matrix)	svg

337	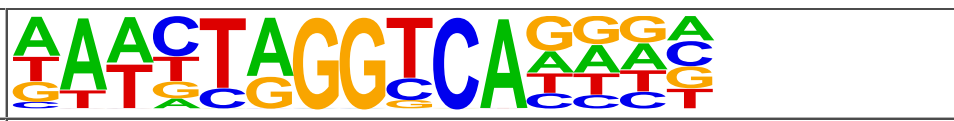	RORa(NR)/Liver-Rora-ChIP-Seq(GSE101115)/Homer	1e-8	-1.885e+01	0.0000	56.0	1.18%	4.9	0.60%	motif file (matrix)	svg
338	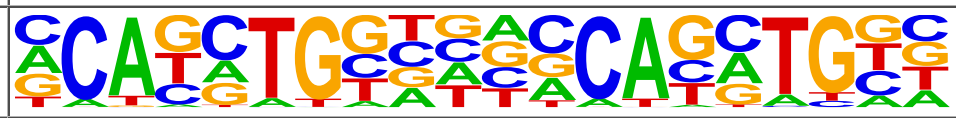	Twist(bHLH)/HMLE-TWIST1-ChIP-Seq(Chang_et_al)/Homer	1e-8	-1.885e+01	0.0000	56.0	1.18%	4.5	0.56%	motif file (matrix)	svg
339	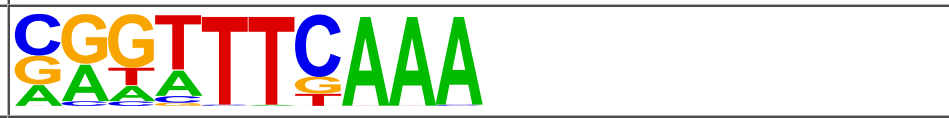	CHR(?)/Hela-CellCycle-Expression/Homer	1e-8	-1.873e+01	0.0000	197.0	4.15%	22.2	2.73%	motif file (matrix)	svg
340	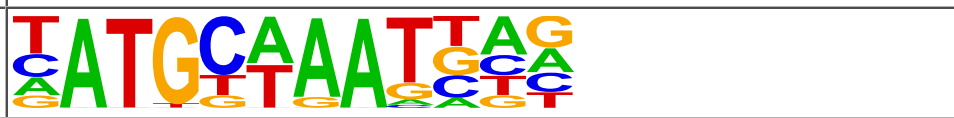	Brn1(POU,Homeobox)/NPC-Brn1-ChIP-Seq(GSE35496)/Homer	1e-8	-1.855e+01	0.0000	160.0	3.37%	17.6	2.17%	motif file (matrix)	svg
341	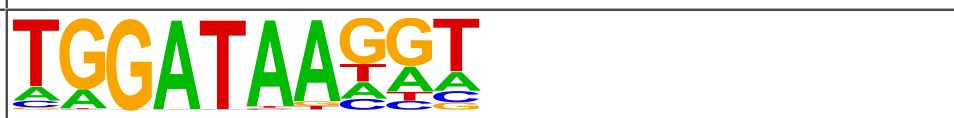	AT5G56840(MYBrelated)/colamp-AT5G56840-DAP-Seq(GSE60143)/Homer	1e-8	-1.847e+01	0.0000	522.0	11.01%	70.2	8.64%	motif file (matrix)	svg
342	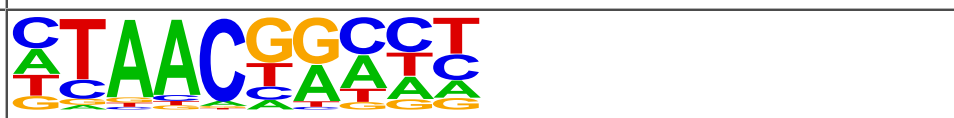	MYB56(MYB)/colamp-MYB56-DAP-Seq(GSE60143)/Homer	1e-7	-1.824e+01	0.0000	369.0	7.78%	47.6	5.86%	motif file (matrix)	svg
343	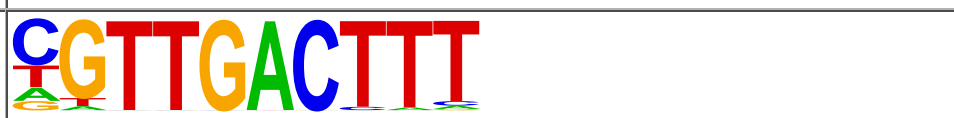	WRKY8(WRKY)/colamp-WRKY8-DAP-Seq(GSE60143)/Homer	1e-7	-1.804e+01	0.0000	24.0	0.51%	1.0	0.12%	motif file (matrix)	svg
344	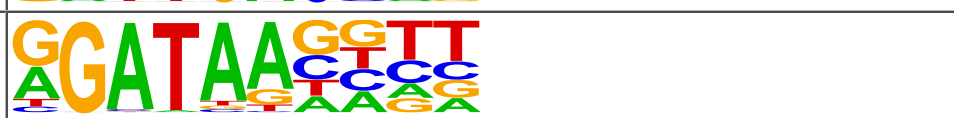	At1g49010(MYBrelated)/col-At1g49010-DAP-Seq(GSE60143)/Homer	1e-7	-1.760e+01	0.0000	699.0	14.74%	98.4	12.11%	motif file (matrix)	svg
345	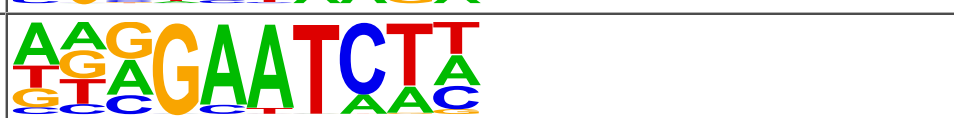	AT5G45580(G2like)/colamp-AT5G45580-DAP-Seq(GSE60143)/Homer	1e-7	-1.757e+01	0.0000	635.0	13.39%	88.3	10.87%	motif file (matrix)	svg
346	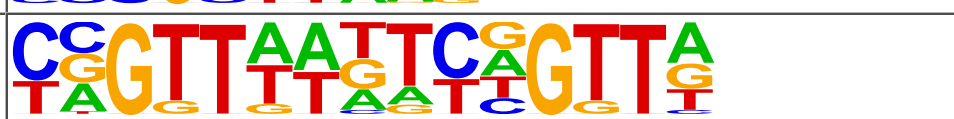	MYB44(MYB)/colamp-MYB44-DAP-Seq(GSE60143)/Homer	1e-7	-1.717e+01	0.0000	80.0	1.69%	7.0	0.87%	motif file (matrix)	svg
347	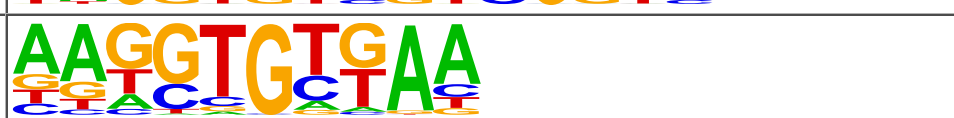	Tbr1(T-box)/Cortex-Tbr1-ChIP-Seq(GSE71384)/Homer	1e-7	-1.685e+01	0.0000	600.0	12.65%	83.2	10.24%	motif file (matrix)	svg
348	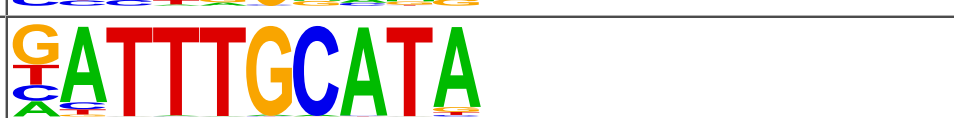	Oct11(POU,Homeobox)/NCIH1048-POU2F3-ChIP-seq(GSE115123)/Homer	1e-7	-1.677e+01	0.0000	149.0	3.14%	16.3	2.01%	motif file (matrix)	svg
349	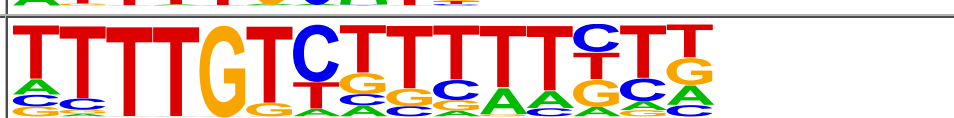	IDD5(C2H2)/colamp-IDD5-DAP-Seq(GSE60143)/Homer	1e-7	-1.650e+01	0.0000	410.0	8.65%	54.5	6.71%	motif file (matrix)	svg
350	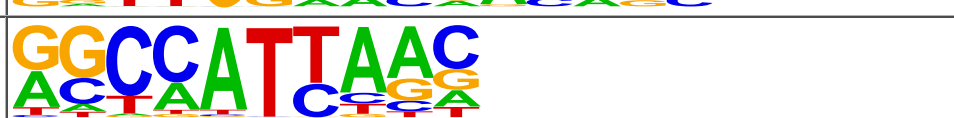	Nanog(Homeobox)/mES-Nanog-ChIP-Seq(GSE11724)/Homer	1e-7	-1.649e+01	0.0000	1994.0	42.05%	311.8	38.39%	motif file (matrix)	svg
351	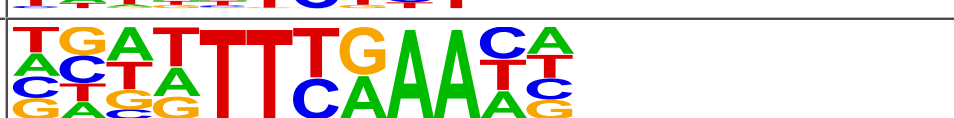	TCX2(CPP)/colamp-TCX2-DAP-Seq(GSE60143)/Homer	1e-7	-1.648e+01	0.0000	456.0	9.62%	61.6	7.58%	motif file (matrix)	svg
352	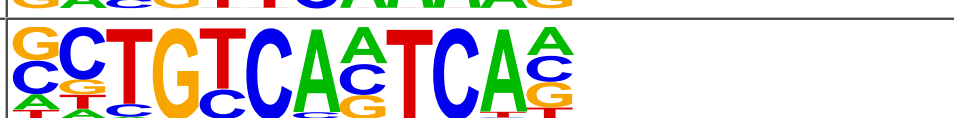	Pknox1(Homeobox)/ES-Prep1-ChIP-Seq(GSE63282)/Homer	1e-7	-1.647e+01	0.0000	126.0	2.66%	13.4	1.65%	motif file (matrix)	svg
353	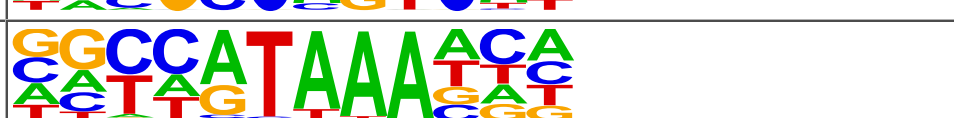	CDX4(Homeobox)/ZebrafishEmbryos-Cdx4.Myc-ChIP-Seq(GSE48254)/Homer	1e-7	-1.632e+01	0.0000	282.0	5.95%	35.2	4.34%	motif file (matrix)	svg
354	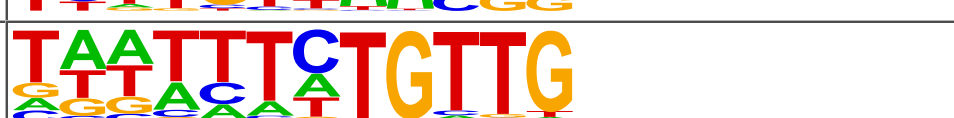	RAV1(RAV)/colamp-RAV1-DAP-Seq(GSE60143)/Homer	1e-7	-1.621e+01	0.0000	409.0	8.63%	54.2	6.68%	motif file (matrix)	svg
355	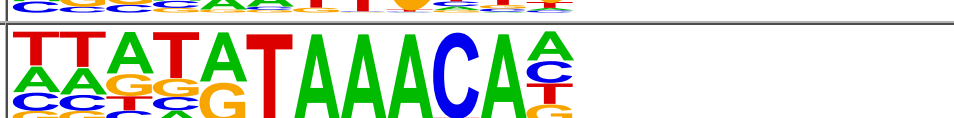	Foxf1(Forkhead)/Lung-Foxf1-ChIP-Seq(GSE77951)/Homer	1e-6	-1.609e+01	0.0000	402.0	8.48%	53.1	6.53%	motif file (matrix)	svg
356	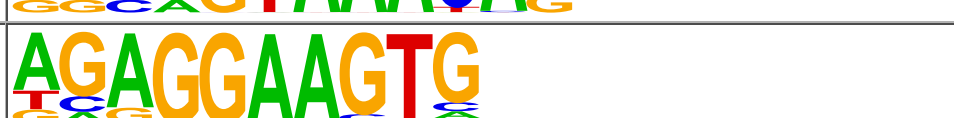	PU.1(ETS)/ThioMac-PU.1-ChIP-Seq(GSE21512)/Homer	1e-6	-1.595e+01	0.0000	335.0	7.06%	43.8	5.39%	motif file (matrix)	svg
357	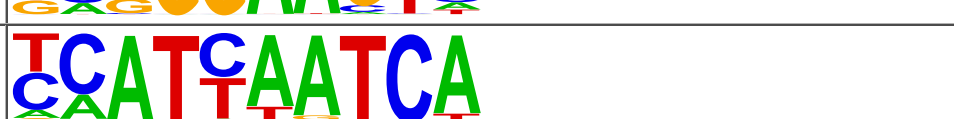	Pdx1(Homeobox)/Islet-Pdx1-ChIP-Seq(SRA008281)/Homer	1e-6	-1.584e+01	0.0000	301.0	6.35%	38.8	4.78%	motif file (matrix)	svg
358	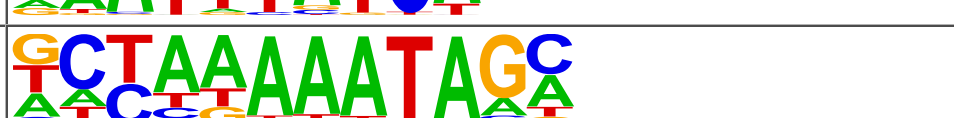	Mef2c(MADS)/GM12878-Mef2c-ChIP-Seq(GSE32465)/Homer	1e-6	-1.573e+01	0.0000	183.0	3.86%	21.9	2.69%	motif file (matrix)	svg
359	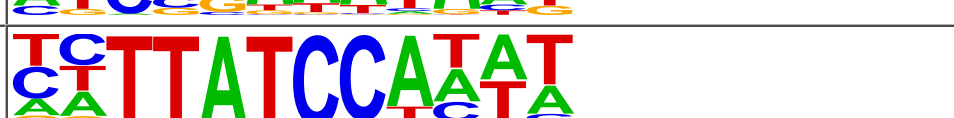	At1g74840(MYBrelated)/col100-At1g74840-DAP-Seq(GSE60143)/Homer	1e-6	-1.568e+01	0.0000	394.0	8.31%	53.0	6.52%	motif file (matrix)	svg
360	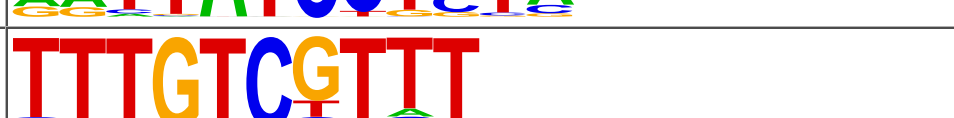	AtIDD11(C2H2)/colamp-AtIDD11-DAP-Seq(GSE60143)/Homer	1e-6	-1.565e+01	0.0000	197.0	4.15%	23.6	2.91%	motif file (matrix)	svg
361	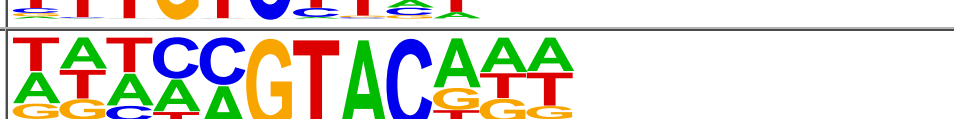	SPL15(SBP)/colamp-SPL15-DAP-Seq(GSE60143)/Homer	1e-6	-1.560e+01	0.0000	684.0	14.42%	97.1	11.96%	motif file (matrix)	svg
362	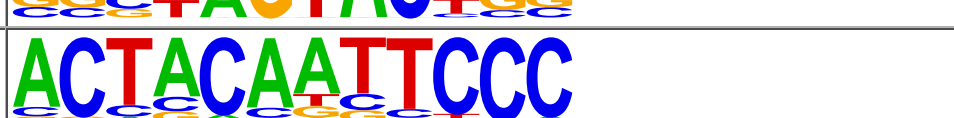	GFY(?)/Promoter/Homer	1e-6	-1.550e+01	0.0000	43.0	0.91%	3.7	0.45%	motif file (matrix)	svg
363	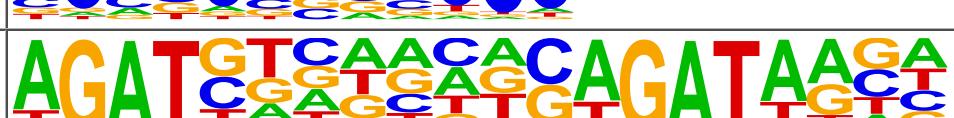	GATA3(Zf),DR8/iTreg-Gata3-ChIP-Seq(GSE20898)/Homer	1e-6	-1.526e+01	0.0000	33.0	0.70%	2.4	0.29%	motif file (matrix)	svg
364	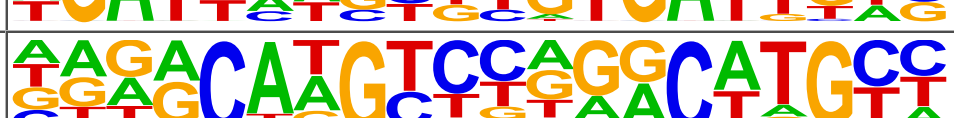	p73(p53)/Trachea-p73-ChIP-Seq(PRJNA310161)/Homer	1e-6	-1.523e+01	0.0000	22.0	0.46%	0.4	0.05%	motif file (matrix)	svg
365	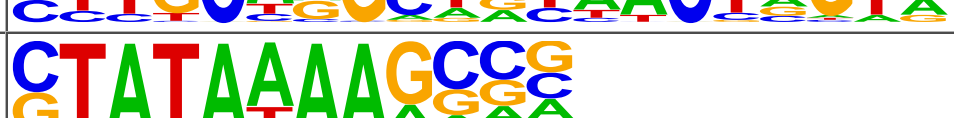	TATA-box/Drosophila-Promoters/Homer	1e-6	-1.523e+01	0.0000	22.0	0.46%	1.0	0.12%	motif file (matrix)	svg
366	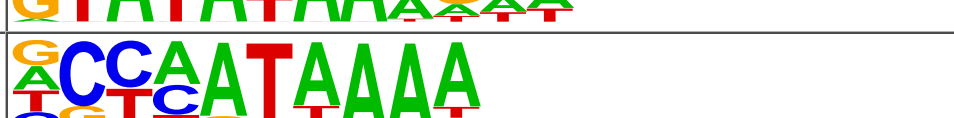	Hoxd13(Homeobox)/ChickenMSG-Hoxd13.Flag-ChIP-Seq(GSE86088)/Homer	1e-6	-1.521e+01	0.0000	606.0	12.78%	85.3	10.51%	motif file (matrix)	svg
367	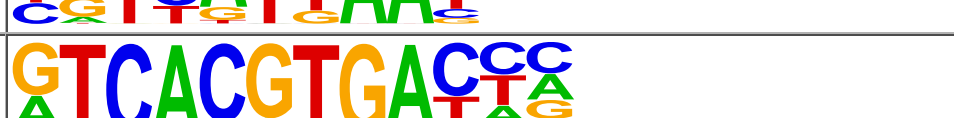	TFE3(bHLH)/MEF-TFE3-ChIP-Seq(GSE75757)/Homer	1e-6	-1.514e+01	0.0000	77.0	1.62%	7.6	0.93%	motif file (matrix)	svg
368	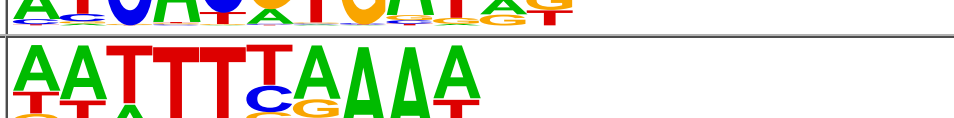	Unknown6/Drosophila-Promoters/Homer	1e-6	-1.504e+01	0.0000	312.0	6.58%	40.8	5.02%	motif file (matrix)	svg
369	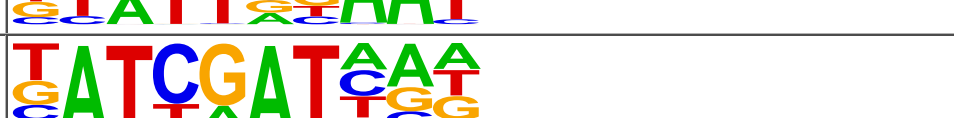	CUX1(Homeobox)/K562-CUX1-ChIP-Seq(GSE92882)/Homer	1e-6	-1.502e+01	0.0000	285.0	6.01%	36.2	4.46%	motif file (matrix)	svg
370	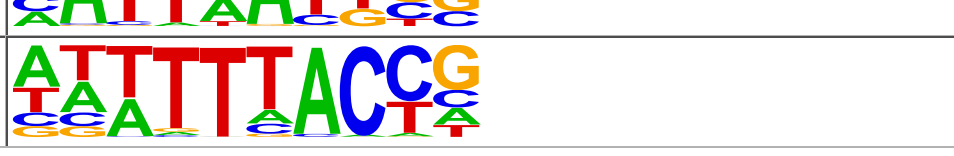	AT5G47660(Trihelix)/colamp-AT5G47660-DAP-Seq(GSE60143)/Homer	1e-6	-1.500e+01	0.0000	483.0	10.19%	66.3	8.17%	motif file (matrix)	svg

371	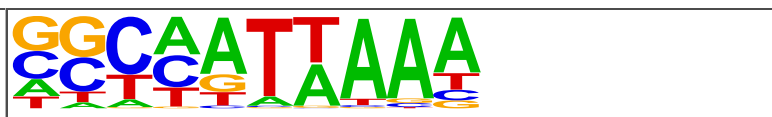	Unknown(Homeobox)/Limb-p300-ChIP-Seq/Homer	1e-6	-1.491e+01	0.0000	325.0	6.85%	42.5	5.23%	motif file (matrix)	svg
372	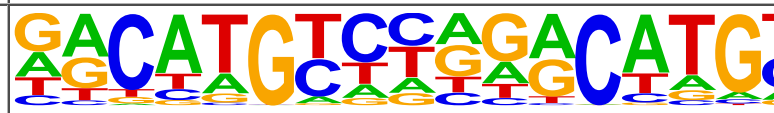	p53(p53)/Saos-p53-ChIP-Seq(GSE15780)/Homer	1e-6	-1.458e+01	0.0000	42.0	0.89%	3.8	0.47%	motif file (matrix)	svg
373	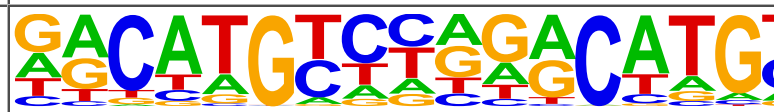	p53(p53)/Saos-p53-ChIP-Seq/Homer	1e-6	-1.458e+01	0.0000	42.0	0.89%	3.8	0.47%	motif file (matrix)	svg
374	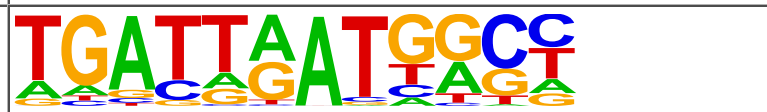	Hoxb4(Homeobox)/ES-Hoxb4-ChIP-Seq(GSE34014)/Homer	1e-6	-1.456e+01	0.0000	51.0	1.08%	4.0	0.50%	motif file (matrix)	svg
375	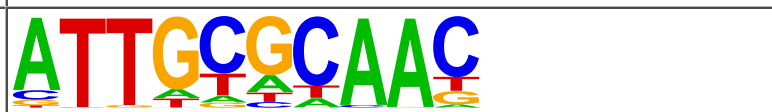	CEBP(bZIP)/ThioMac-CEBPb-ChIP-Seq(GSE21512)/Homer	1e-6	-1.447e+01	0.0000	263.0	5.55%	33.6	4.13%	motif file (matrix)	svg
376	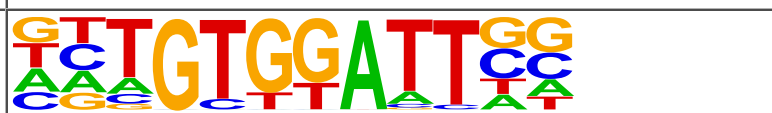	Foxh1(Forkhead)/hESC-FOXH1-ChIP-Seq(GSE29422)/Homer	1e-6	-1.445e+01	0.0000	290.0	6.12%	37.7	4.64%	motif file (matrix)	svg
377	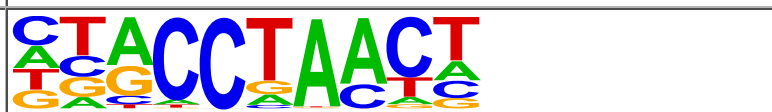	MYB62(MYB)/colamp-MYB62-DAP-Seq(GSE60143)/Homer	1e-6	-1.426e+01	0.0000	722.0	15.23%	104.5	12.87%	motif file (matrix)	svg
378	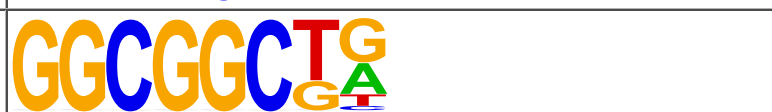	ERF1(AP2EREBP)/colamp-ERF1-DAP-Seq(GSE60143)/Homer	1e-6	-1.420e+01	0.0000	3287.0	69.32%	536.5	66.05%	motif file (matrix)	svg
379	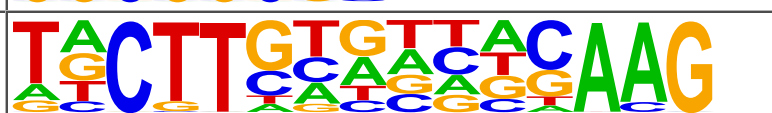	ANAC011(NAC)/col-ANAC011-DAP-Seq(GSE60143)/Homer	1e-6	-1.412e+01	0.0000	114.0	2.40%	12.2	1.50%	motif file (matrix)	svg
380	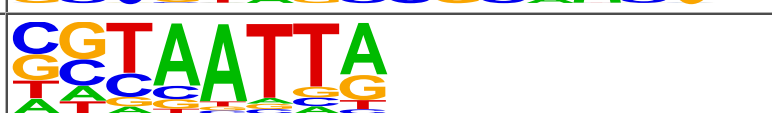	DLX5(Homeobox)/BasalGanglia-Dlx5-ChIP-seq(GSE124936)/Homer	1e-6	-1.410e+01	0.0000	309.0	6.52%	40.1	4.94%	motif file (matrix)	svg
381	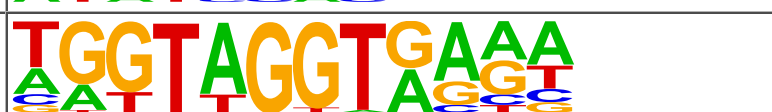	MYB40(MYB)/col-MYB40-DAP-Seq(GSE60143)/Homer	1e-6	-1.406e+01	0.0000	136.0	2.87%	15.1	1.85%	motif file (matrix)	svg
382	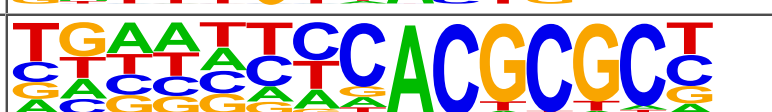	FAR1(FAR1)/col-FAR1-DAP-Seq(GSE60143)/Homer	1e-6	-1.393e+01	0.0000	594.0	12.53%	84.9	10.45%	motif file (matrix)	svg
383	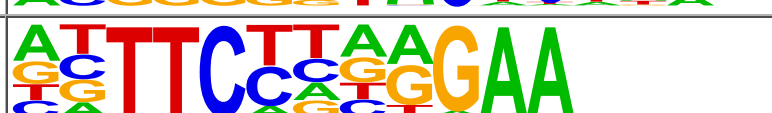	STAT6(Stat)/CD4-Stat6-ChIP-Seq(GSE22104)/Homer	1e-5	-1.372e+01	0.0000	164.0	3.46%	19.4	2.39%	motif file (matrix)	svg
384	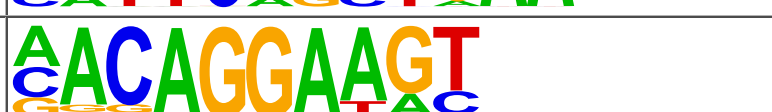	Ets1-distal(ETS)/CD4+-PolII-ChIP-Seq(Barski_et_al.)/Homer	1e-5	-1.341e+01	0.0000	149.0	3.14%	17.6	2.16%	motif file (matrix)	svg
385	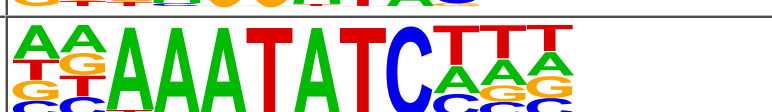	AT3G10113(MYBrelated)/col-AT3G10113-DAP-Seq(GSE60143)/Homer	1e-5	-1.340e+01	0.0000	320.0	6.75%	42.9	5.28%	motif file (matrix)	svg
386	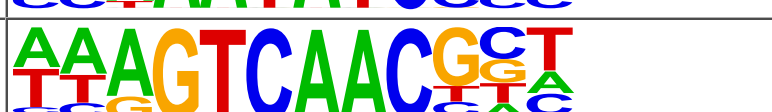	WRKY47(WRKY)/colamp-WRKY47-DAP-Seq(GSE60143)/Homer	1e-5	-1.321e+01	0.0000	74.0	1.56%	8.0	0.98%	motif file (matrix)	svg
387	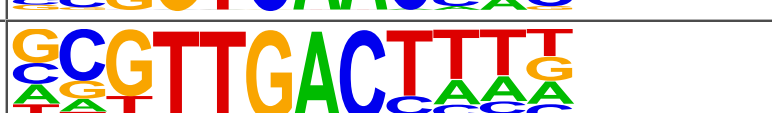	WRKY11(WRKY)/col-WRKY11-DAP-Seq(GSE60143)/Homer	1e-5	-1.317e+01	0.0000	31.0	0.65%	2.9	0.36%	motif file (matrix)	svg
388	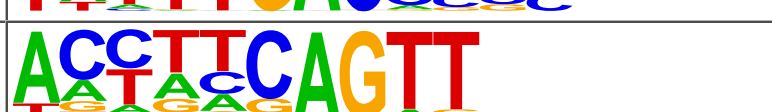	JGL(C2H2)/col-JGL-DAP-Seq(GSE60143)/Homer	1e-5	-1.313e+01	0.0000	641.0	13.52%	92.7	11.41%	motif file (matrix)	svg
389	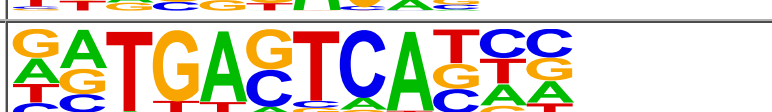	Fosl2(bZIP)/3T3L1-Fosl2-ChIP-Seq(GSE56872)/Homer	1e-5	-1.288e+01	0.0000	89.0	1.88%	9.4	1.15%	motif file (matrix)	svg
390	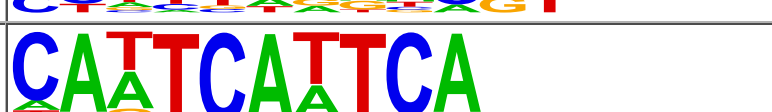	WUS1(Homeobox)/colamp-WUS1-DAP-Seq(GSE60143)/Homer	1e-5	-1.288e+01	0.0000	169.0	3.56%	20.2	2.49%	motif file (matrix)	svg
391	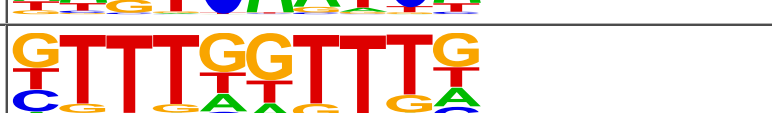	HuR(?)/HEK293-HuR-CLIP-Seq(GSE87887)/Homer	1e-5	-1.287e+01	0.0000	1384.0	29.19%	213.1	26.23%	motif file (matrix)	svg
392	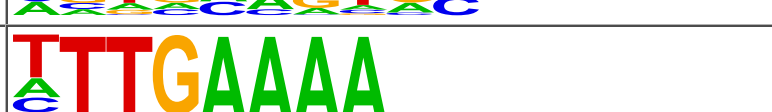	At2g41835(C2H2)/col-At2g41835-DAP-Seq(GSE60143)/Homer	1e-5	-1.283e+01	0.0000	258.0	5.44%	33.7	4.15%	motif file (matrix)	svg
393	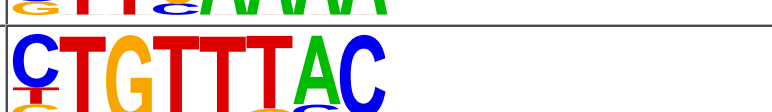	Foxo1(Forkhead)/RAW-Foxo1-ChIP-Seq(Fan_et_al.)/Homer	1e-5	-1.276e+01	0.0000	1012.0	21.34%	152.5	18.77%	motif file (matrix)	svg
394	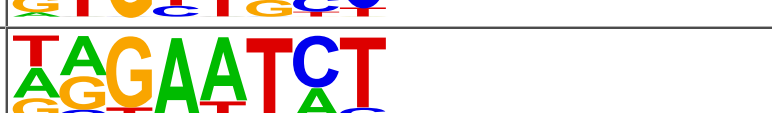	At3g04030(G2like)/col-At3g04030-DAP-Seq(GSE60143)/Homer	1e-5	-1.271e+01	0.0000	448.0	9.45%	62.7	7.72%	motif file (matrix)	svg
395	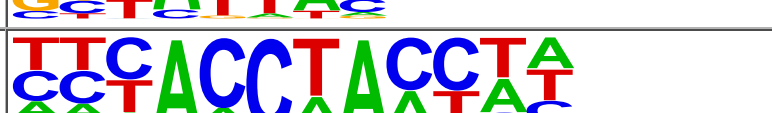	MYB74(MYB)/colamp-MYB74-DAP-Seq(GSE60143)/Homer	1e-5	-1.270e+01	0.0000	271.0	5.71%	35.6	4.38%	motif file (matrix)	svg
396	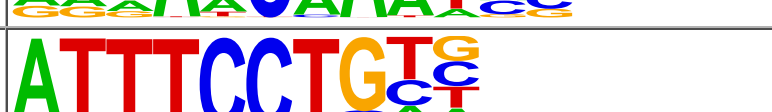	EWS:ERG-fusion(ETS)/CADO_ES1-EWS:ERG-ChIP-Seq(SRA014231)/Homer	1e-5	-1.267e+01	0.0000	370.0	7.80%	50.6	6.23%	motif file (matrix)	svg
397	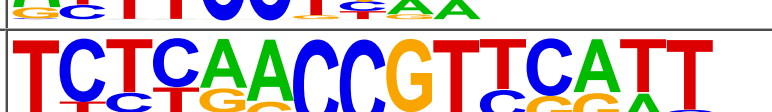	AT5G59990(C2C2COlike)/colamp-AT5G59990-DAP-Seq(GSE60143)/Homer	1e-5	-1.258e+01	0.0000	20.0	0.42%	1.3	0.16%	motif file (matrix)	svg
398	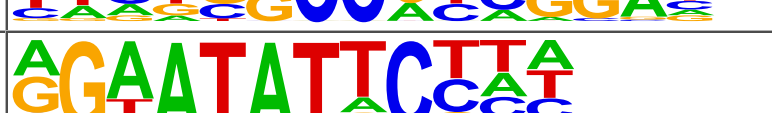	At5g29000(G2like)/col-At5g29000-DAP-Seq(GSE60143)/Homer	1e-5	-1.257e+01	0.0000	96.0	2.02%	10.5	1.29%	motif file (matrix)	svg
399	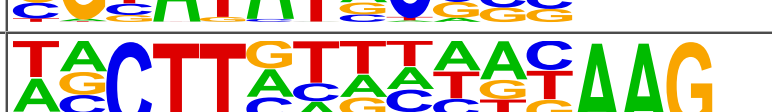	ANAC005(NAC)/col-ANAC005-DAP-Seq(GSE60143)/Homer	1e-5	-1.250e+01	0.0000	161.0	3.40%	19.5	2.40%	motif file (matrix)	svg
400	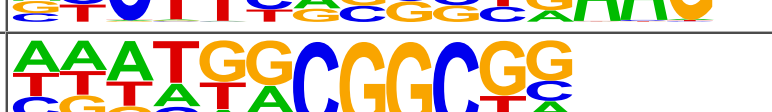	RAP26(AP2EREBP)/colamp-RAP26-DAP-Seq(GSE60143)/Homer	1e-5	-1.240e+01	0.0000	3457.0	72.90%	568.8	70.03%	motif file (matrix)	svg
401	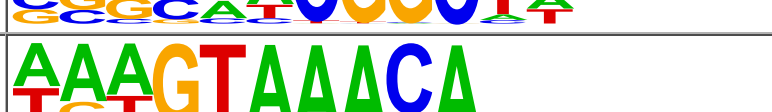	FOXA1(Forkhead)/LNCAP-FOXA1-ChIP-Seq(GSE27824)/Homer	1e-5	-1.213e+01	0.0000	452.0	9.53%	63.9	7.87%	motif file (matrix)	svg
402	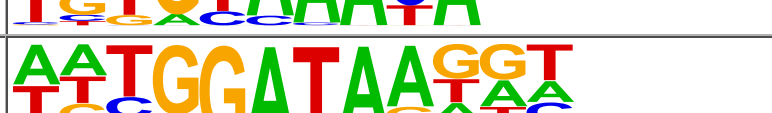	AT4G00250(GeBP)/col-AT4G00250-DAP-Seq(GSE60143)/Homer	1e-5	-1.210e+01	0.0000	289.0	6.09%	38.8	4.77%	motif file (matrix)	svg
403	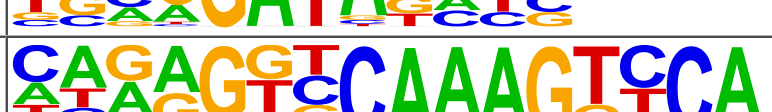	HNF4a(NR),DR1/HepG2-HNF4a-ChIP-Seq(GSE25021)/Homer	1e-5	-1.175e+01	0.0000	173.0	3.65%	21.2	2.61%	motif file (matrix)	svg
404	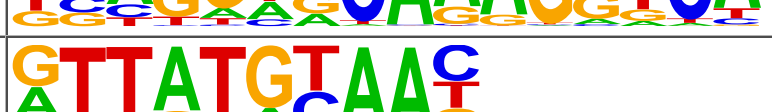	HLF(bZIP)/HSC-HLF.Flag-ChIP-Seq(GSE69817)/Homer	1e-5	-1.164e+01	0.0000	294.0	6.20%	39.6	4.88%	motif file (matrix)	svg

405	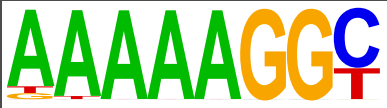	dof42(C2C2dof)/col-dof42-DAP-Seq(GSE60143)/Homer	1e-5	-1.163e+01	0.0000	353.0	7.44%	48.1	5.93%	motif file (matrix)	svg
406	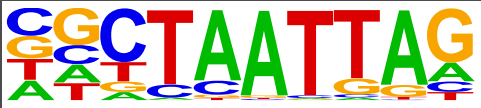	LHX9(Homeobox)/Hct116-LHX9.V5-ChIP-Seq(GSE116822)/Homer	1e-5	-1.159e+01	0.0000	488.0	10.29%	70.0	8.62%	motif file (matrix)	svg
407	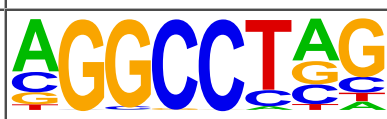	ZNF711(Zf)/SHSY5Y-ZNF711-ChIP-Seq(GSE20673)/Homer	1e-4	-1.149e+01	0.0000	2826.0	59.60%	459.9	56.62%	motif file (matrix)	svg
408	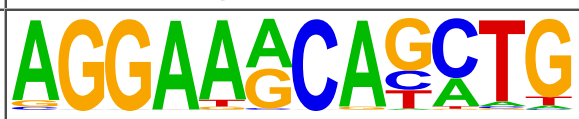	ETS:E-box(ETS,bHLH)/HPC7-Scl-ChIP-Seq(GSE22178)/Homer	1e-4	-1.147e+01	0.0000	47.0	0.99%	4.4	0.54%	motif file (matrix)	svg
409	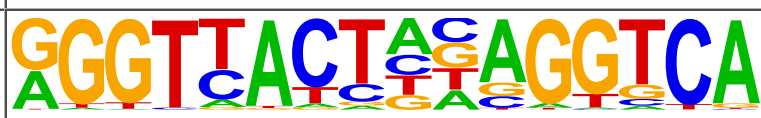	LXRE(NR),DR4/RAW-LXRb.biotin-ChIP-Seq(GSE21512)/Homer	1e-4	-1.133e+01	0.0000	19.0	0.40%	0.7	0.09%	motif file (matrix)	svg
410	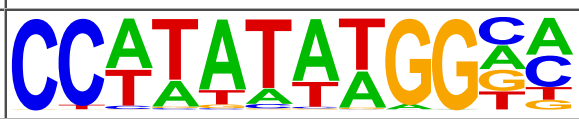	CArG(MADS)/PUER-Srf-ChIP-Seq(Sullivan_et_al.)/Homer	1e-4	-1.133e+01	0.0000	144.0	3.04%	17.3	2.13%	motif file (matrix)	svg
411	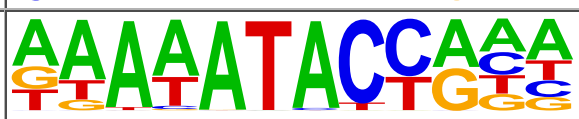	Unknown4/Drosophila-Promoters/Homer	1e-4	-1.114e+01	0.0000	38.0	0.80%	3.3	0.40%	motif file (matrix)	svg
412	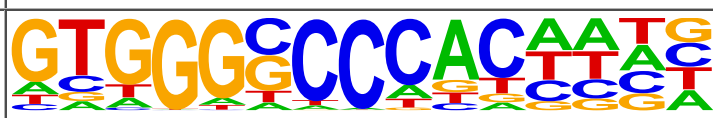	TCP7(TCP)/col-TCP7-DAP-Seq(GSE60143)/Homer	1e-4	-1.107e+01	0.0000	774.0	16.32%	115.1	14.17%	motif file (matrix)	svg
413	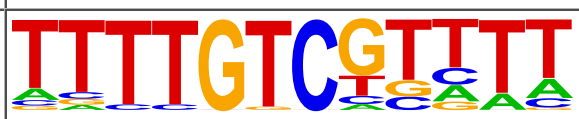	JKD(C2H2)/col-JKD-DAP-Seq(GSE60143)/Homer	1e-4	-1.095e+01	0.0000	136.0	2.87%	16.9	2.08%	motif file (matrix)	svg
414	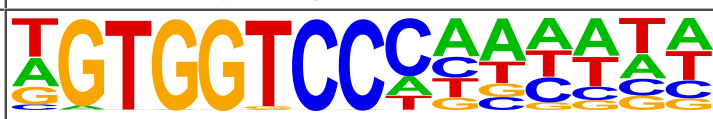	TCP3(TCP)/colamp-TCP3-DAP-Seq(GSE60143)/Homer	1e-4	-1.093e+01	0.0000	143.0	3.02%	17.0	2.10%	motif file (matrix)	svg
415	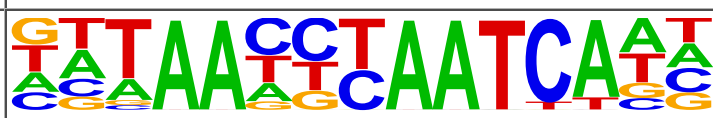	DUX4(Homeobox)/Myoblasts-DUX4.V5-ChIP-Seq(GSE75791)/Homer	1e-4	-1.075e+01	0.0001	46.0	0.97%	4.9	0.60%	motif file (matrix)	svg
416	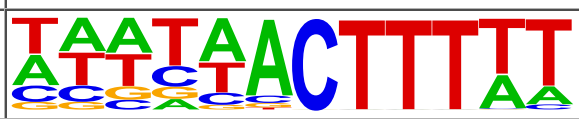	At4g38000(C2C2dof)/col-At4g38000-DAP-Seq(GSE60143)/Homer	1e-4	-1.072e+01	0.0001	522.0	11.01%	75.5	9.30%	motif file (matrix)	svg
417	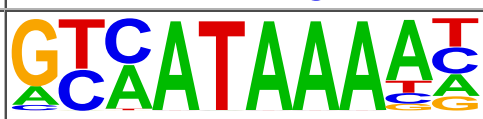	Cdx2(Homeobox)/mES-Cdx2-ChIP-Seq(GSE14586)/Homer	1e-4	-1.069e+01	0.0001	284.0	5.99%	38.4	4.73%	motif file (matrix)	svg
418	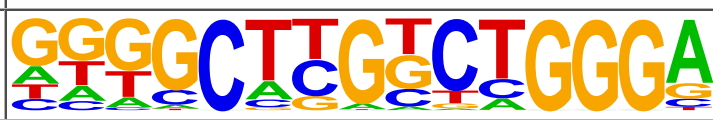	Zfp809(Zf)/ES-Zfp809-ChIP-Seq(GSE70799)/Homer	1e-4	-1.058e+01	0.0001	578.0	12.19%	84.4	10.39%	motif file (matrix)	svg
419	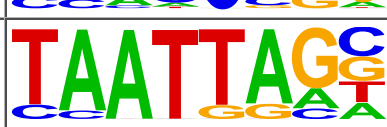	Lhx2(Homeobox)/HFSC-Lhx2-ChIP-Seq(GSE48068)/Homer	1e-4	-1.050e+01	0.0001	342.0	7.21%	47.1	5.79%	motif file (matrix)	svg
420	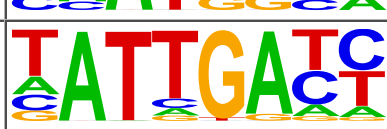	Hnf6b(Homeobox)/LNCaP-Hnf6b-ChIP-Seq(GSE106305)/Homer	1e-4	-1.046e+01	0.0001	270.0	5.69%	36.0	4.44%	motif file (matrix)	svg
421	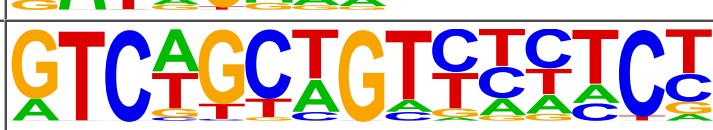	ZNF317(Zf)/HEK293-ZNF317.GFP-ChIP-Seq(GSE58341)/Homer	1e-4	-1.035e+01	0.0001	37.0	0.78%	3.9	0.48%	motif file (matrix)	svg
422	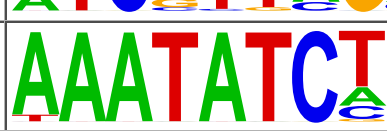	EPR1(MYBrelated)/colamp-EPR1-DAP-Seq(GSE60143)/Homer	1e-4	-1.029e+01	0.0001	223.0	4.70%	29.9	3.68%	motif file (matrix)	svg
423	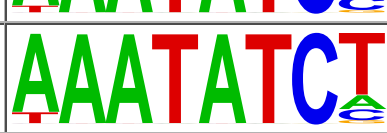	LHY1(MYBrelated)/col-LHY1-DAP-Seq(GSE60143)/Homer	1e-4	-1.029e+01	0.0001	223.0	4.70%	29.9	3.68%	motif file (matrix)	svg
424	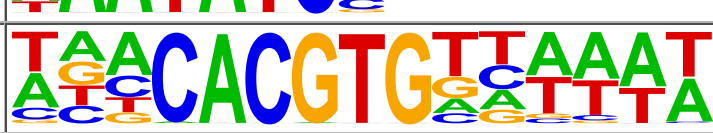	GT3a(Trihelix)/col-GT3a-DAP-Seq(GSE60143)/Homer	1e-4	-1.022e+01	0.0001	155.0	3.27%	19.9	2.45%	motif file (matrix)	svg
425	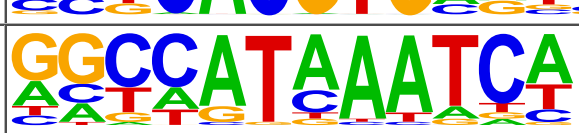	HOXA9(Homeobox)/HSC-Hoxa9-ChIP-Seq(GSE33509)/Homer	1e-4	-1.017e+01	0.0001	189.0	3.99%	24.7	3.04%	motif file (matrix)	svg
426	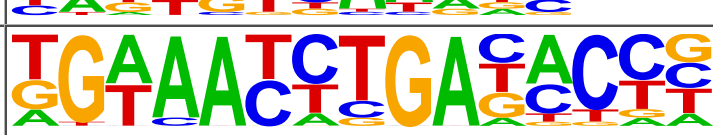	Six4(Homeobox)/MCF7-SIX4-ChIP-Seq(Encode)/Homer	1e-4	-1.013e+01	0.0001	18.0	0.38%	0.7	0.09%	motif file (matrix)	svg
427	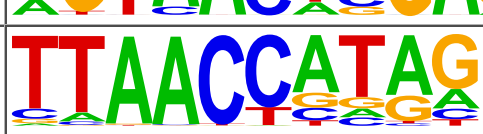	AT3G25990(Trihelix)/colamp-AT3G25990-DAP-Seq(GSE60143)/Homer	1e-4	-1.002e+01	0.0001	202.0	4.26%	26.9	3.31%	motif file (matrix)	svg
428	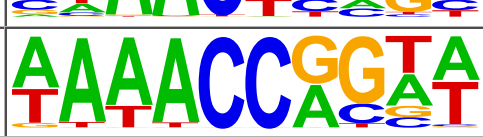	AT1G76870(Trihelix)/col-AT1G76870-DAP-Seq(GSE60143)/Homer	1e-4	-9.766e+00	0.0001	126.0	2.66%	15.7	1.93%	motif file (matrix)	svg
429	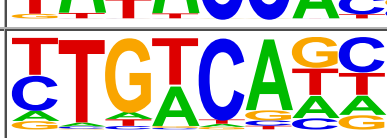	Tgif1(Homeobox)/mES-Tgif1-ChIP-Seq(GSE55404)/Homer	1e-4	-9.731e+00	0.0001	1344.0	28.34%	210.2	25.88%	motif file (matrix)	svg
430	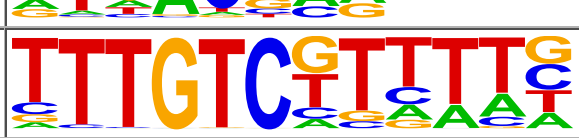	IDD7(C2H2)/col-IDD7-DAP-Seq(GSE60143)/Homer	1e-4	-9.707e+00	0.0001	201.0	4.24%	26.7	3.28%	motif file (matrix)	svg
431	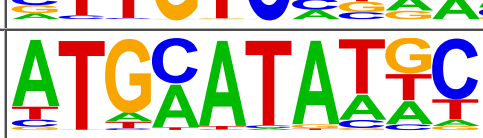	Pit1(Homeobox)/GCrat-Pit1-ChIP-Seq(GSE58009)/Homer	1e-4	-9.432e+00	0.0002	408.0	8.60%	58.6	7.21%	motif file (matrix)	svg
432	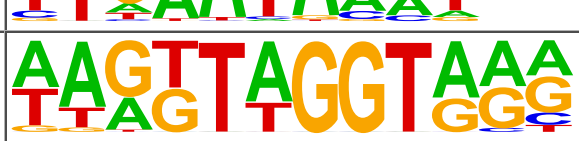	MYB4(MYB)/col200-MYB4-DAP-Seq(GSE60143)/Homer	1e-4	-9.381e+00	0.0002	266.0	5.61%	36.9	4.54%	motif file (matrix)	svg
433	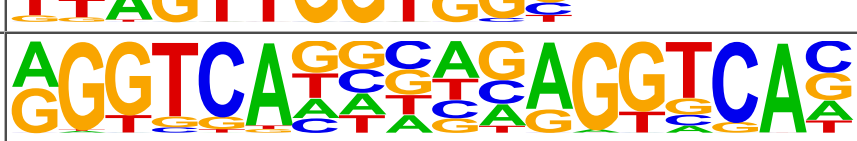	RAR:RXR(NR),DR5/ES-RAR-ChIP-Seq(GSE56893)/Homer	1e-3	-8.976e+00	0.0003	17.0	0.36%	0.1	0.01%	motif file (matrix)	svg
434	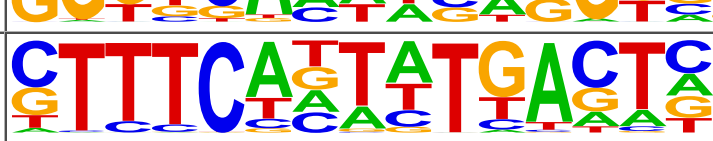	IRF:BATF(IRF:bZIP)/pDC-Irf8-ChIP-Seq(GSE66899)/Homer	1e-3	-8.826e+00	0.0003	35.0	0.74%	3.6	0.44%	motif file (matrix)	svg
435	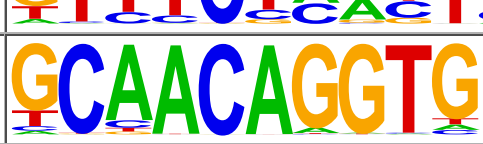	SCRT1(Zf)/HEK293-SCRT1.eGFP-ChIP-Seq(Encode)/Homer	1e-3	-8.808e+00	0.0003	198.0	4.18%	26.8	3.30%	motif file (matrix)	svg
436	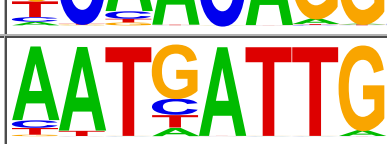	ATHB7(Homeobox)/col-ATHB7-DAP-Seq(GSE60143)/Homer	1e-3	-8.760e+00	0.0004	244.0	5.15%	33.1	4.08%	motif file (matrix)	svg
437	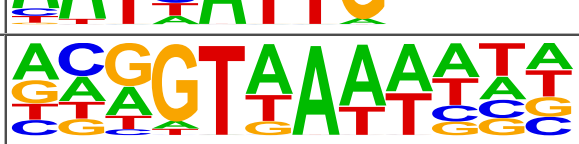	GT2(Trihelix)/colamp-GT2-DAP-Seq(GSE60143)/Homer	1e-3	-8.748e+00	0.0004	341.0	7.19%	48.6	5.99%	motif file (matrix)	svg
438	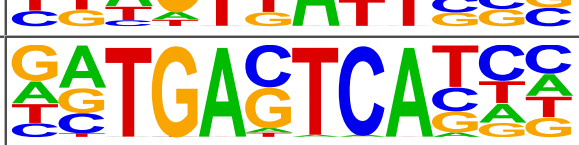	Jun-AP1(bZIP)/K562-cJun-ChIP-Seq(GSE31477)/Homer	1e-3	-8.668e+00	0.0004	66.0	1.39%	7.3	0.89%	motif file (matrix)	svg

439	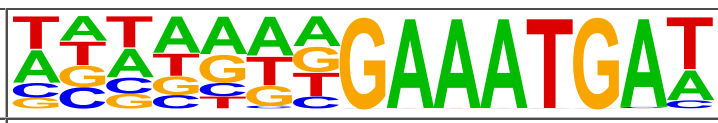	AT2G31460(REMB3)/col-AT2G31460-DAP-Seq(GSE60143)/Homer	1e-3	-8.661e+00	0.0004	130.0	2.74%	16.1	1.98%	motif file (matrix)	svg
440	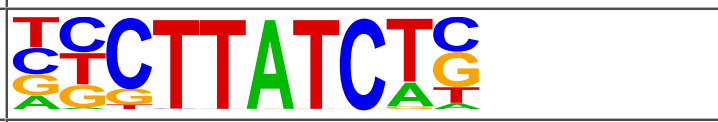	Gata2(Zf)/K562-GATA2-ChIP-Seq(GSE18829)/Homer	1e-3	-8.408e+00	0.0005	223.0	4.70%	30.7	3.78%	motif file (matrix)	svg
441	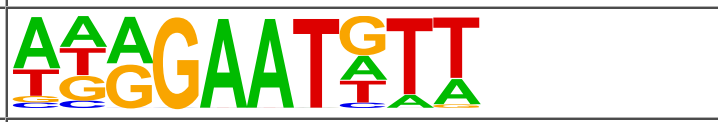	AT2G38300(G2like)/col-AT2G38300-DAP-Seq(GSE60143)/Homer	1e-3	-8.345e+00	0.0005	572.0	12.06%	85.2	10.50%	motif file (matrix)	svg
442	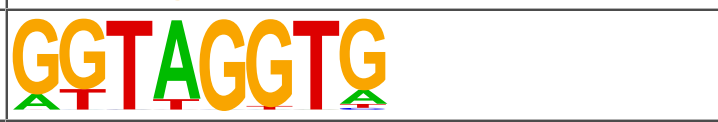	MYB99(MYB)/colamp-MYB99-DAP-Seq(GSE60143)/Homer	1e-3	-8.282e+00	0.0006	434.0	9.15%	63.7	7.84%	motif file (matrix)	svg
443	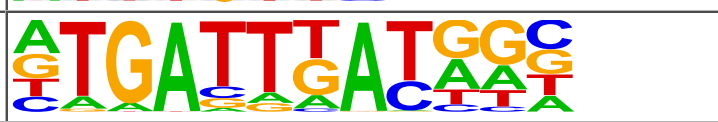	PBX2(Homeobox)/K562-PBX2-ChIP-Seq(Encode)/Homer	1e-3	-8.260e+00	0.0006	229.0	4.83%	31.7	3.90%	motif file (matrix)	svg
444	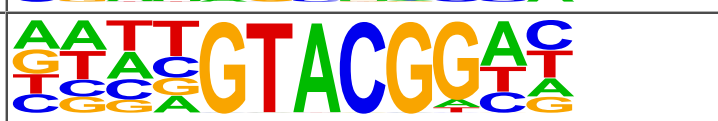	bHLH28(bHLH)/col-bHLH28-DAP-Seq(GSE60143)/Homer	1e-3	-8.243e+00	0.0006	242.0	5.10%	33.7	4.15%	motif file (matrix)	svg
445	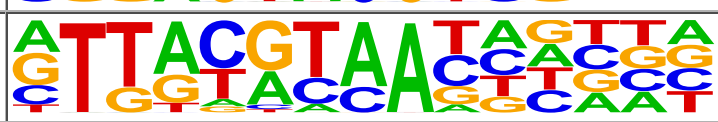	NFIL3(bZIP)/HepG2-NFIL3-ChIP-Seq(Encode)/Homer	1e-3	-8.239e+00	0.0006	255.0	5.38%	35.2	4.34%	motif file (matrix)	svg
446	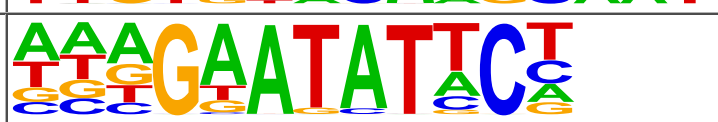	At2g03500(G2like)/col-At2g03500-DAP-Seq(GSE60143)/Homer	1e-3	-8.223e+00	0.0006	94.0	1.98%	11.5	1.41%	motif file (matrix)	svg
447	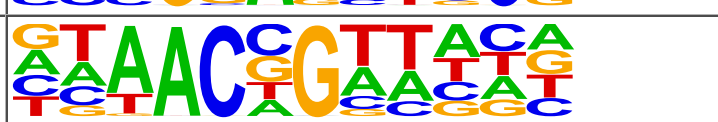	MYB81(MYB)/col-MYB81-DAP-Seq(GSE60143)/Homer	1e-3	-8.223e+00	0.0006	658.0	13.88%	99.2	12.21%	motif file (matrix)	svg
448	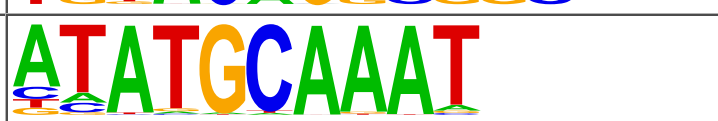	Oct2(POU,Homeobox)/Bcell-Oct2-ChIP-Seq(GSE21512)/Homer	1e-3	-8.212e+00	0.0006	176.0	3.71%	23.2	2.86%	motif file (matrix)	svg
449	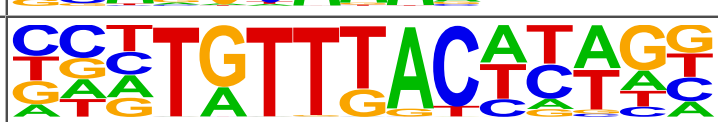	Foxa3(Forkhead)/Liver-Foxa3-ChIP-Seq(GSE77670)/Homer	1e-3	-8.205e+00	0.0006	101.0	2.13%	12.4	1.53%	motif file (matrix)	svg
450	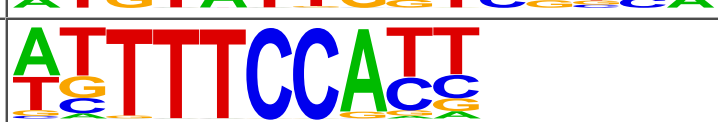	NFAT(RHD)/Jurkat-NFATC1-ChIP-Seq(Jolma_et_al.)/Homer	1e-3	-8.116e+00	0.0007	552.0	11.64%	82.5	10.15%	motif file (matrix)	svg
451	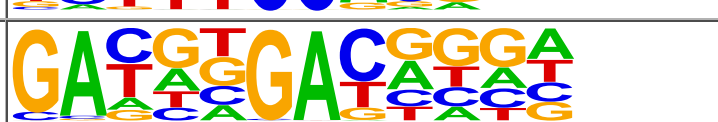	Knotted(Homeobox)/Corn-KN1-ChIP-Seq(GSE39161)/Homer	1e-3	-8.092e+00	0.0007	1361.0	28.70%	216.0	26.59%	motif file (matrix)	svg
452	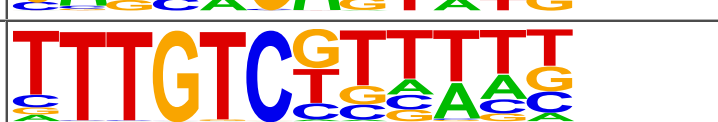	At5g66730(C2H2)/colamp-At5g66730-DAP-Seq(GSE60143)/Homer	1e-3	-8.022e+00	0.0007	162.0	3.42%	22.0	2.70%	motif file (matrix)	svg
453	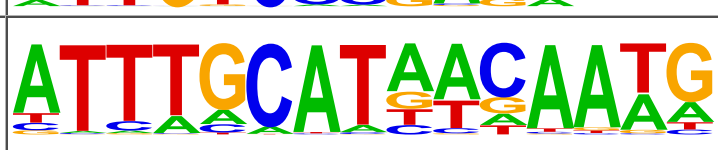	OCT4-SOX2-TCF-NANOG(POU,Homeobox,HMG)/mES-Oct4-ChIP-Seq(GSE11431)/Homer	1e-3	-8.003e+00	0.0007	72.0	1.52%	8.3	1.02%	motif file (matrix)	svg
454	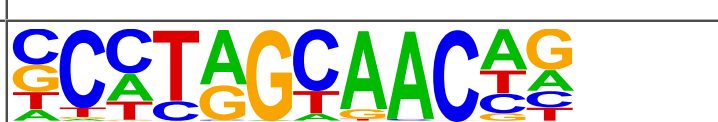	Rfx5(HTH)/GM12878-Rfx5-ChIP-Seq(GSE31477)/Homer	1e-3	-8.001e+00	0.0007	228.0	4.81%	31.2	3.84%	motif file (matrix)	svg
455	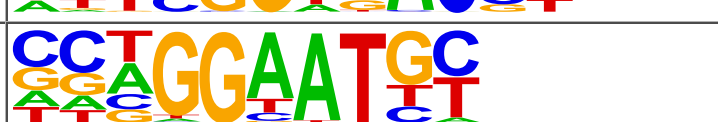	TEAD4(TEA)/Tropoblast-Tead4-ChIP-Seq(GSE37350)/Homer	1e-3	-7.982e+00	0.0008	445.0	9.38%	65.1	8.01%	motif file (matrix)	svg
456	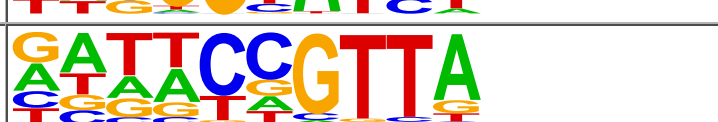	MYB105(MYB)/colamp-MYB105-DAP-Seq(GSE60143)/Homer	1e-3	-7.925e+00	0.0008	286.0	6.03%	40.1	4.94%	motif file (matrix)	svg
457	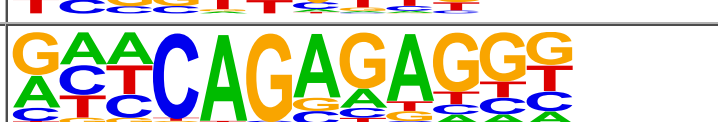	ZNF768(Zf)/Rajj-ZNF768-ChIP-Seq(GSE111879)/Homer	1e-3	-7.879e+00	0.0008	16.0	0.34%	0.7	0.08%	motif file (matrix)	svg
458	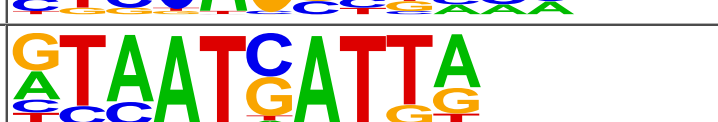	PHV(HB)/col-PHV-DAP-Seq(GSE60143)/Homer	1e-3	-7.861e+00	0.0008	114.0	2.40%	14.8	1.82%	motif file (matrix)	svg
459	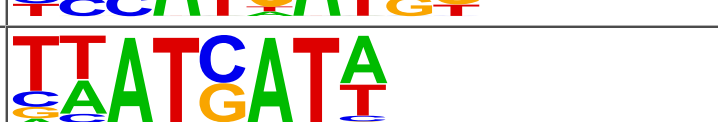	CRC(C2C2YABBY)/col-CRC-DAP-Seq(GSE60143)/Homer	1e-3	-7.823e+00	0.0009	419.0	8.84%	61.9	7.62%	motif file (matrix)	svg
460	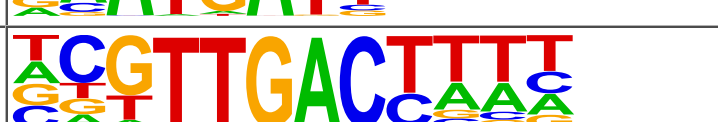	WRKY43(WRKY)/colamp-WRKY43-DAP-Seq(GSE60143)/Homer	1e-3	-7.814e+00	0.0009	100.0	2.11%	12.8	1.57%	motif file (matrix)	svg
461	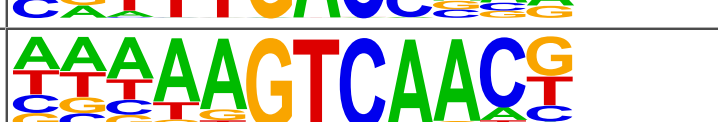	WRKY22(WRKY)/colamp-WRKY22-DAP-Seq(GSE60143)/Homer	1e-3	-7.565e+00	0.0011	187.0	3.94%	25.5	3.14%	motif file (matrix)	svg
462	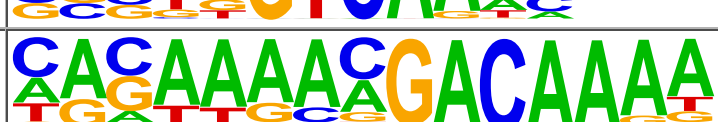	At1g14580(C2H2)/colamp-At1g14580-DAP-Seq(GSE60143)/Homer	1e-3	-7.556e+00	0.0011	120.0	2.53%	15.1	1.86%	motif file (matrix)	svg
463	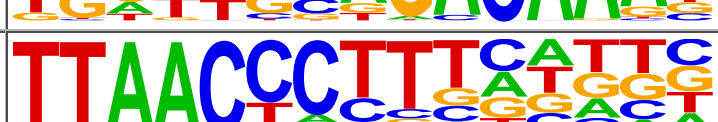	ZNF652/HepG2-ZNF652.Flag-ChIP-Seq(Encode)/Homer	1e-3	-7.543e+00	0.0012	71.0	1.50%	8.4	1.03%	motif file (matrix)	svg
464	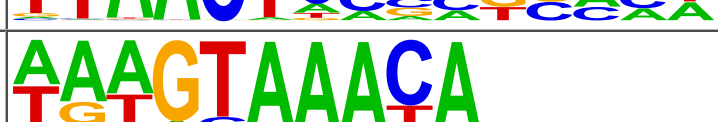	FOXA1(Forkhead)/MCF7-FOXA1-ChIP-Seq(GSE26831)/Homer	1e-3	-7.537e+00	0.0012	386.0	8.14%	56.5	6.95%	motif file (matrix)	svg
465	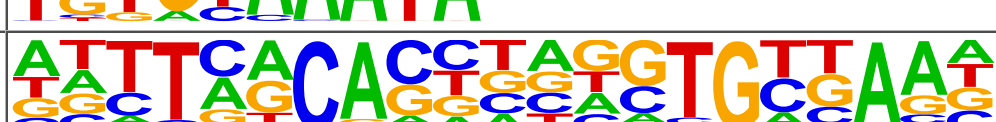	Brachyury(T-box)/Mesoendoderm-Brachyury-ChIP-exo(GSE54963)/Homer	1e-3	-7.467e+00	0.0012	78.0	1.64%	9.7	1.20%	motif file (matrix)	svg
466	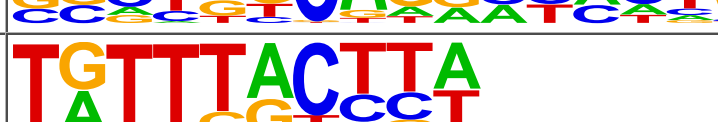	FOXM1(Forkhead)/MCF7-FOXM1-ChIP-Seq(GSE72977)/Homer	1e-3	-7.349e+00	0.0014	429.0	9.05%	63.3	7.79%	motif file (matrix)	svg
467	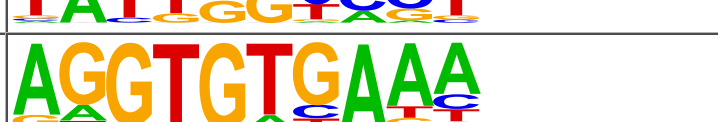	Tbx21(T-box)/GM12878-TBX21-ChIP-Seq(Encode)/Homer	1e-3	-7.265e+00	0.0015	416.0	8.77%	61.1	7.53%	motif file (matrix)	svg
468	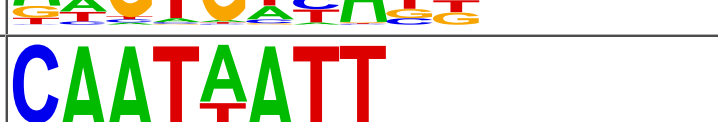	ATHB53(HB)/col-ATHB53-DAP-Seq(GSE60143)/Homer	1e-3	-7.236e+00	0.0015	166.0	3.50%	22.7	2.79%	motif file (matrix)	svg
469	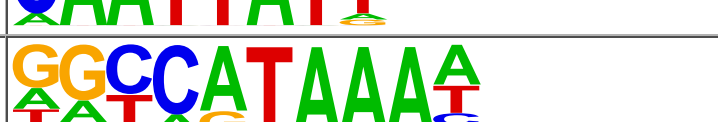	caudal(Homeobox)/Drosophila-Embryos-ChIP-Chip(modEncode)/Homer	1e-3	-7.212e+00	0.0016	270.0	5.69%	38.0	4.68%	motif file (matrix)	svg
470	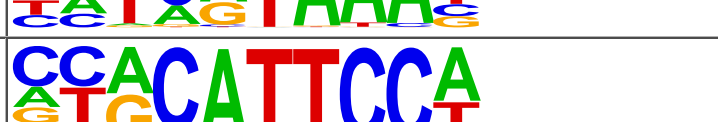	TEAD1(TEAD)/HepG2-TEAD1-ChIP-Seq(Encode)/Homer	1e-3	-7.196e+00	0.0016	503.0	10.61%	75.4	9.29%	motif file (matrix)	svg
471	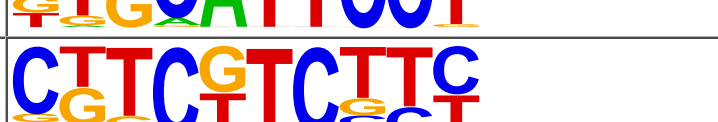	Unknown4/Arabidopsis-Promoters/Homer	1e-3	-7.189e+00	0.0016	1077.0	22.71%	169.9	20.92%	motif file (matrix)	svg
472	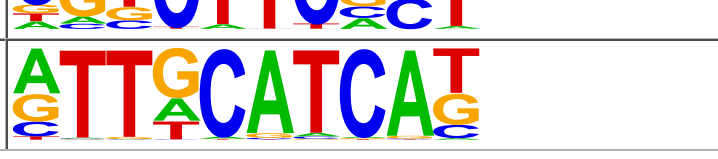	Chop(bZIP)/MEF-Chop-ChIP-Seq(GSE35681)/Homer	1e-3	-7.101e+00	0.0018	105.0	2.21%	13.9	1.71%	motif file (matrix)	svg

473	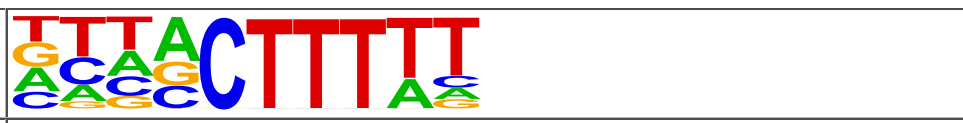	OBP3(C2C2dof)/col-OBP3-DAP-Seq(GSE60143)/Homer	1e-3	-6.921e+00	0.0021	1255.0	26.47%	199.5	24.56%	motif file (matrix)	svg
474	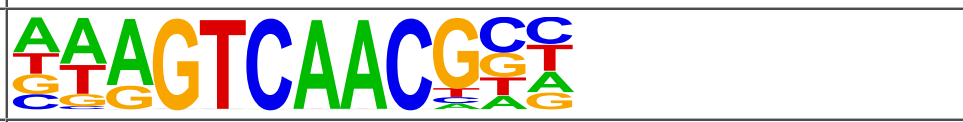	WRKY46(WRKY)/colamp-WRKY46-DAP-Seq(GSE60143)/Homer	1e-2	-6.887e+00	0.0022	24.0	0.51%	2.6	0.32%	motif file (matrix)	svg
475	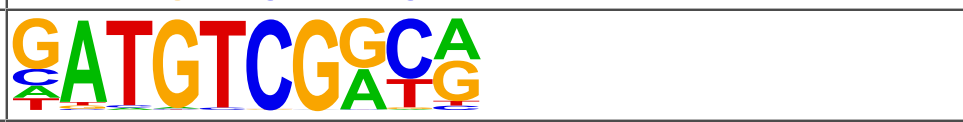	DDF2(AP2EREBP)/col-DDF2-DAP-Seq(GSE60143)/Homer	1e-2	-6.748e+00	0.0025	32.0	0.67%	3.0	0.37%	motif file (matrix)	svg
476	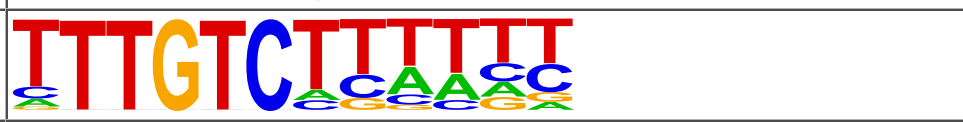	SGR5(C2H2)/colamp-SGR5-DAP-Seq(GSE60143)/Homer	1e-2	-6.663e+00	0.0027	312.0	6.58%	45.1	5.55%	motif file (matrix)	svg
477	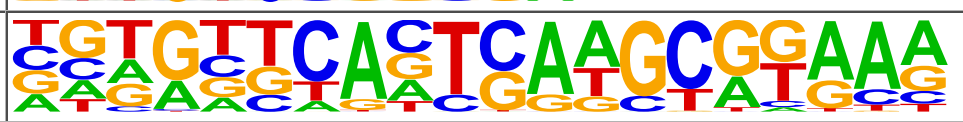	PAX6(Paired,Homeobox)/Forebrain-Pax6-ChIP-Seq(GSE66961)/Homer	1e-2	-6.663e+00	0.0027	69.0	1.46%	8.1	1.00%	motif file (matrix)	svg
478	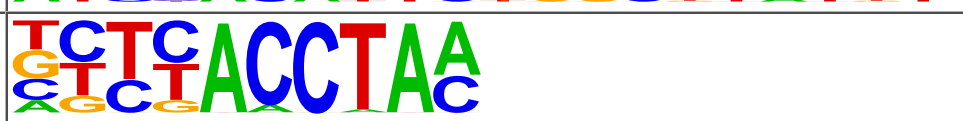	MYB41(MYB)/col-MYB41-DAP-Seq(GSE60143)/Homer	1e-2	-6.632e+00	0.0028	144.0	3.04%	19.3	2.37%	motif file (matrix)	svg
479	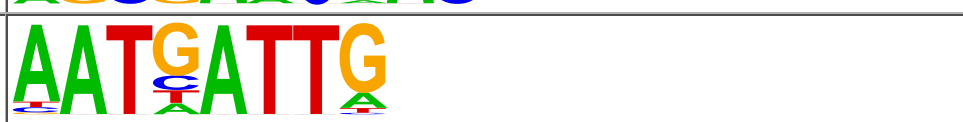	ATHB6(Homeobox)/col-ATHB6-DAP-Seq(GSE60143)/Homer	1e-2	-6.557e+00	0.0030	412.0	8.69%	61.5	7.58%	motif file (matrix)	svg
480	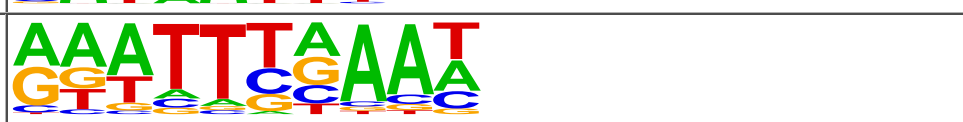	TSO1(CPP)/col-TSO1-DAP-Seq(GSE60143)/Homer	1e-2	-6.555e+00	0.0030	47.0	0.99%	5.9	0.73%	motif file (matrix)	svg
481	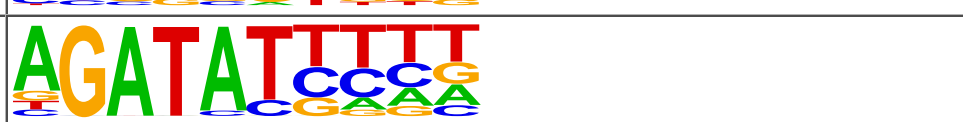	CCA(Myb)/Arabidopsis-CCA.GFP-ChIP-Seq(GSE70533)/Homer	1e-2	-6.345e+00	0.0037	367.0	7.74%	54.9	6.75%	motif file (matrix)	svg
482	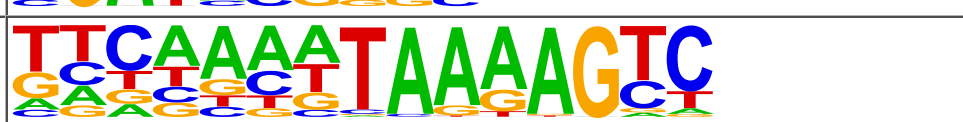	MYNN(Zf)/HEK293-MYNN.eGFP-ChIP-Seq(Encode)/Homer	1e-2	-6.292e+00	0.0039	89.0	1.88%	11.5	1.42%	motif file (matrix)	svg
483	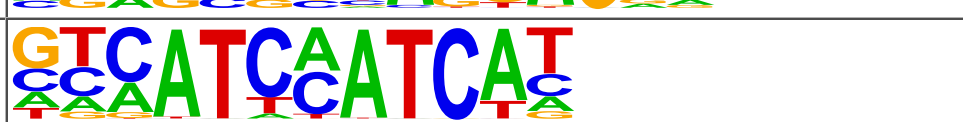	HOXA2(Homeobox)/mES-Hoxa2-ChIP-Seq(Donaldson_et_al.)/Homer	1e-2	-6.111e+00	0.0046	31.0	0.65%	3.6	0.44%	motif file (matrix)	svg
484	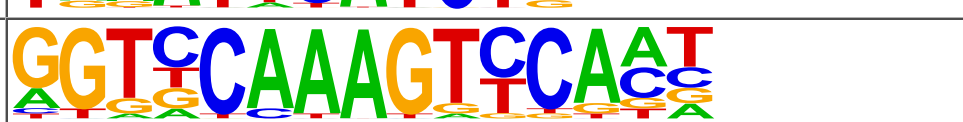	dHNF4(NR)/Fly-HNF4-ChIP-Seq(GSE73675)/Homer	1e-2	-5.860e+00	0.0059	14.0	0.30%	1.0	0.12%	motif file (matrix)	svg
485	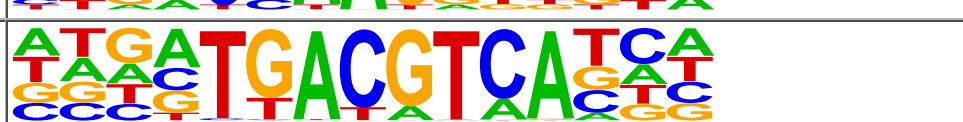	TGA5(bZIP)/col-TGA5-DAP-Seq(GSE60143)/Homer	1e-2	-5.854e+00	0.0059	60.0	1.27%	7.8	0.96%	motif file (matrix)	svg
486	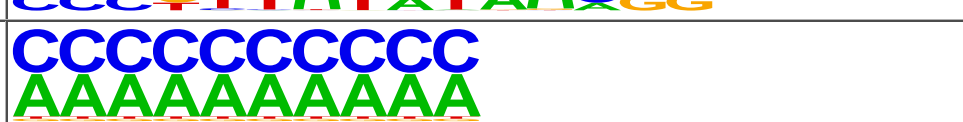	SeqBias: C/A-bias	1e-2	-5.843e+00	0.0060	4742.0	100.00%	811.7	99.93%	motif file (matrix)	svg
487	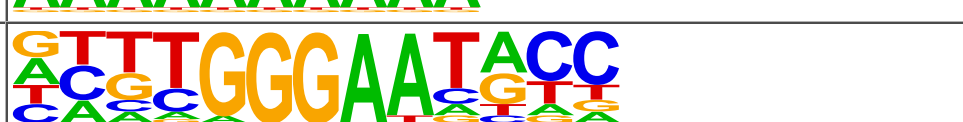	Ik-1	1e-2	-5.843e+00	0.0060	4742.0	100.00%	812.2	100.00%	motif file (matrix)	svg
488	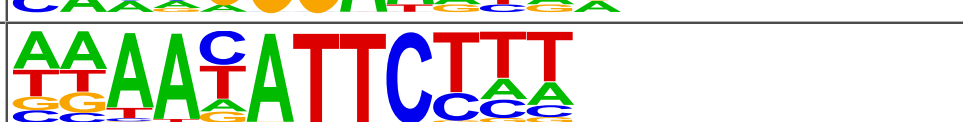	AT2G40260(G2like)/colamp-AT2G40260-DAP-Seq(GSE60143)/Homer	1e-2	-5.800e+00	0.0062	574.0	12.10%	88.5	10.89%	motif file (matrix)	svg
489	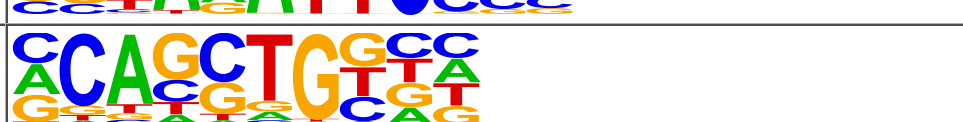	HEB(bHLH)/mES-Heb-ChIP-Seq(GSE53233)/Homer	1e-2	-5.730e+00	0.0067	1855.0	39.12%	302.1	37.19%	motif file (matrix)	svg
490	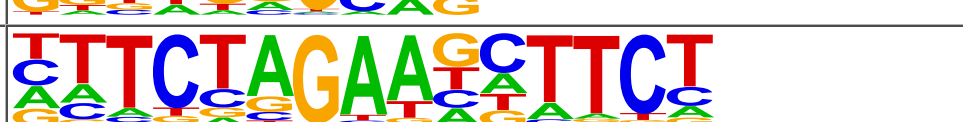	HSF3(HSF)/colamp-HSF3-DAP-Seq(GSE60143)/Homer	1e-2	-5.614e+00	0.0075	369.0	7.78%	55.5	6.84%	motif file (matrix)	svg
491	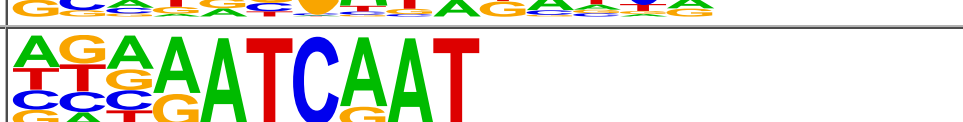	Cux2(Homeobox)/Liver-Cux2-ChIP-Seq(GSE35985)/Homer	1e-2	-5.561e+00	0.0079	127.0	2.68%	17.4	2.15%	motif file (matrix)	svg
492	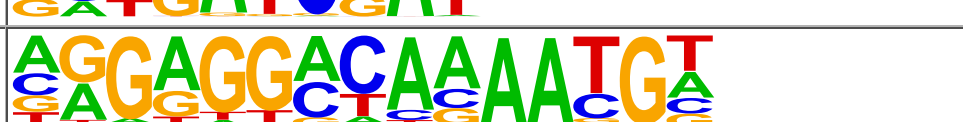	ZNF675(Zf)/HEK293-ZNF675.GFP-ChIP-Seq(GSE58341)/Homer	1e-2	-5.558e+00	0.0079	45.0	0.95%	5.4	0.66%	motif file (matrix)	svg
493	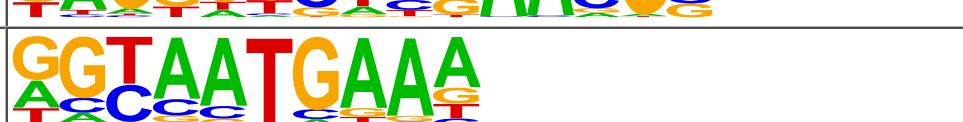	Hoxa10(Homeobox)/ChickenMSG-Hoxa10.Flag-ChIP-Seq(GSE86088)/Homer	1e-2	-5.466e+00	0.0086	198.0	4.18%	28.5	3.51%	motif file (matrix)	svg
494	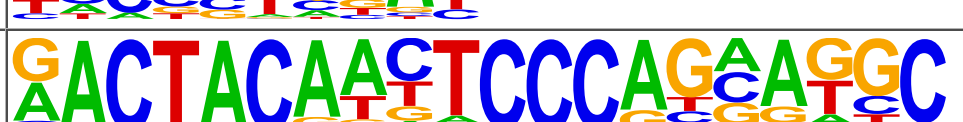	Ronin(THAP)/ES-Thap11-ChIP-Seq(GSE51522)/Homer	1e-2	-5.416e+00	0.0090	22.0	0.46%	2.6	0.32%	motif file (matrix)	svg
495	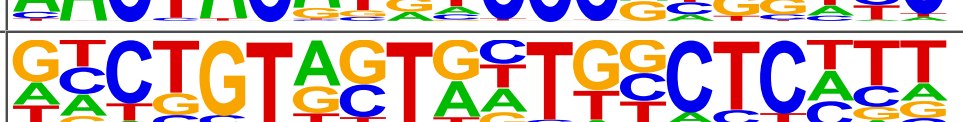	ZNF382(Zf)/HEK293-ZNF382.GFP-ChIP-Seq(GSE58341)/Homer	1e-2	-5.416e+00	0.0090	22.0	0.46%	2.3	0.28%	motif file (matrix)	svg
496	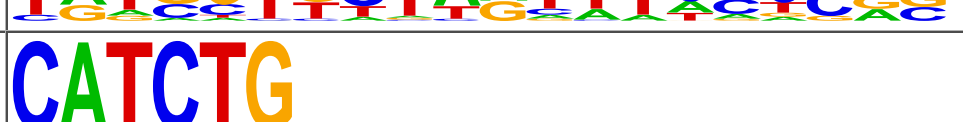	Tal1	1e-2	-5.293e+00	0.0102	686.0	14.47%	107.5	13.23%	motif file (matrix)	svg
497	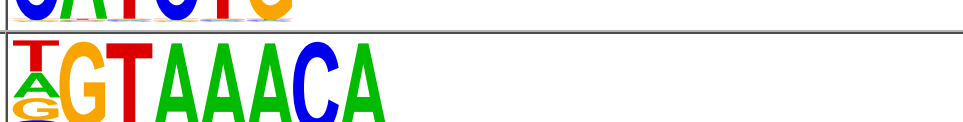	Foxo3(Forkhead)/U2OS-Foxo3-ChIP-Seq(E-MTAB-2701)/Homer	1e-2	-5.241e+00	0.0107	273.0	5.76%	40.9	5.03%	motif file (matrix)	svg
498	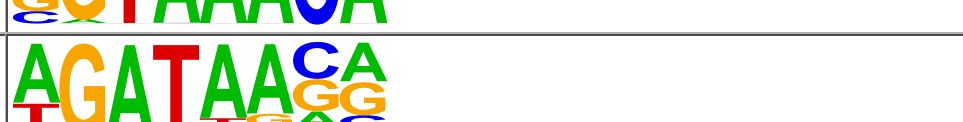	GATA3(Zf)/iTreg-Gata3-ChIP-Seq(GSE20898)/Homer	1e-2	-5.224e+00	0.0109	533.0	11.24%	82.6	10.17%	motif file (matrix)	svg
499	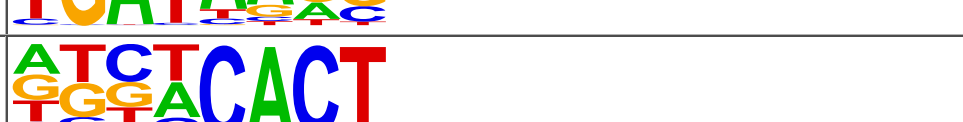	AZF1(C2H2)/colamp-AZF1-DAP-Seq(GSE60143)/Homer	1e-2	-5.160e+00	0.0116	2947.0	62.15%	490.1	60.34%	motif file (matrix)	svg
500	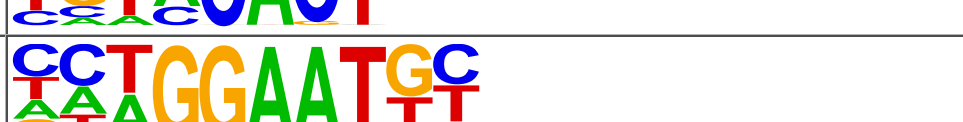	TEAD(TEA)/Fibroblast-PU.1-ChIP-Seq(Unpublished)/Homer	1e-2	-5.059e+00	0.0128	272.0	5.74%	40.0	4.93%	motif file (matrix)	svg
501	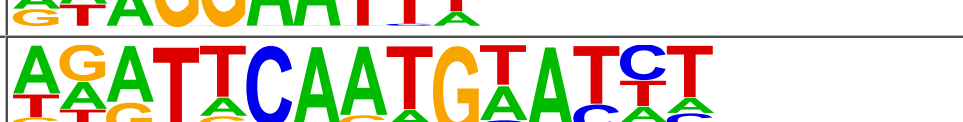	EIN3(EIL)/col-EIN3-DAP-Seq(GSE60143)/Homer	1e-2	-5.037e+00	0.0130	58.0	1.22%	7.8	0.96%	motif file (matrix)	svg
502	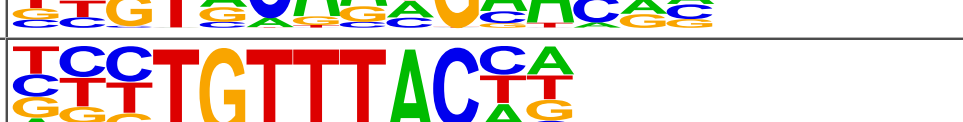	FOXP1(Forkhead)/H9-FOXP1-ChIP-Seq(GSE31006)/Homer	1e-2	-5.035e+00	0.0130	196.0	4.13%	28.2	3.47%	motif file (matrix)	svg
503	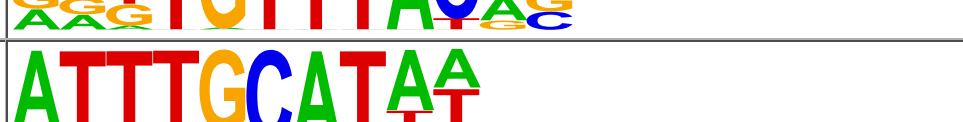	Oct4(POU,Homeobox)/mES-Oct4-ChIP-Seq(GSE11431)/Homer	1e-2	-5.020e+00	0.0132	234.0	4.93%	34.1	4.19%	motif file (matrix)	svg
504	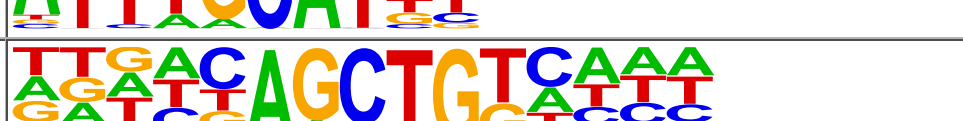	bZIP52(bZIP)/colamp-bZIP52-DAP-Seq(GSE60143)/Homer	1e-2	-4.895e+00	0.0149	628.0	13.24%	98.4	12.11%	motif file (matrix)	svg
505	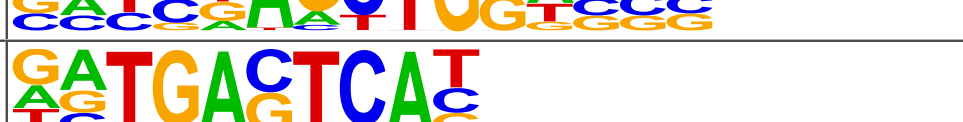	JunB(bZIP)/DendriticCells-Junb-ChIP-Seq(GSE36099)/Homer	1e-2	-4.883e+00	0.0151	131.0	2.76%	18.3	2.25%	motif file (matrix)	svg
506	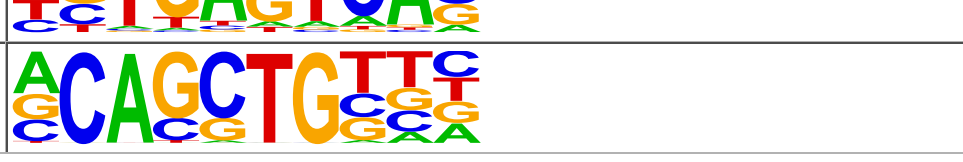	Ptf1a(bHLH)/Panc1-Ptf1a-ChIP-Seq(GSE47459)/Homer	1e-2	-4.880e+00	0.0151	2186.0	46.10%	360.6	44.40%	motif file (matrix)	svg

507	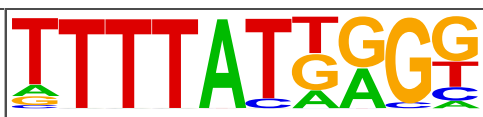	HOXB13(Homeobox)/ ProstateTumor-HOXB13-ChIP-Seq(GSE56288)/Homer	1e-2	-4.741e+00	0.0173	419.0	8.84%	64.6	7.95%	motif file (matrix)	svg
508	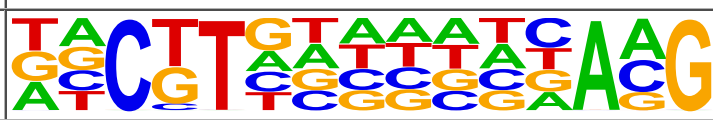	ANAC045(NAC)/col-ANAC045-DAP-Seq(GSE60143)/Homer	1e-2	-4.738e+00	0.0173	1132.0	23.87%	182.9	22.52%	motif file (matrix)	svg
509	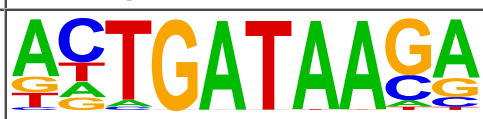	PQM-1(?)/cElegans-L3-ChIP-Seq(modEncode)/Homer	1e-2	-4.681e+00	0.0183	156.0	3.29%	22.8	2.81%	motif file (matrix)	svg
510	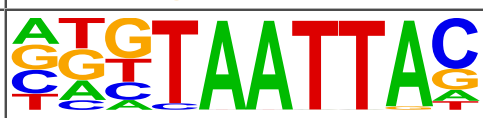	Dlx3(Homeobox)/Kerainocytes-Dlx3-ChIP-Seq(GSE89884)/Homer	1e-2	-4.625e+00	0.0193	213.0	4.49%	31.6	3.89%	motif file (matrix)	svg
511	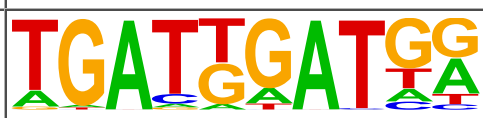	HOXA1(Homeobox)/mES-Hoxa1-ChIP-Seq(SRP084292)/Homer	1e-2	-4.622e+00	0.0194	84.0	1.77%	11.1	1.37%	motif file (matrix)	svg

Homer *de novo* Motif Results (KO_H3K36m2/)

Known Motif Enrichment Results
Gene Ontology Enrichment Results
If Homer is having trouble matching a motif to a known motif, try copy/pasting the matrix file into STAMP
More information on motif finding results: HOMER | Description of Results | Tips
Total target sequences = 1755
Total background sequences = 36534
* - possible false positive

Rank	Motif	P-value	log P-pvalue	% of Targets	% of Background	STD(Bg STD)	Best Match/Details	Motif File
1	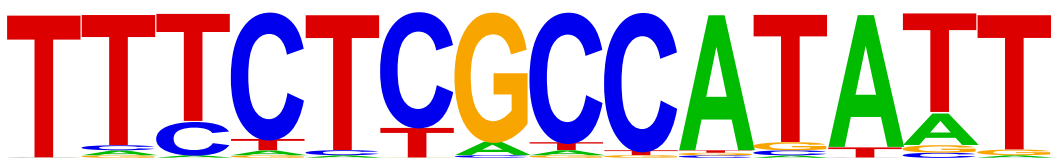	1e-294	-6.782e+02	5.75%	0.00%	132.0bp (0.0bp)	E2F7/MA0758.1/ Jaspar(0.709) More Information | Similar Motifs Found	motif file (matrix)
2	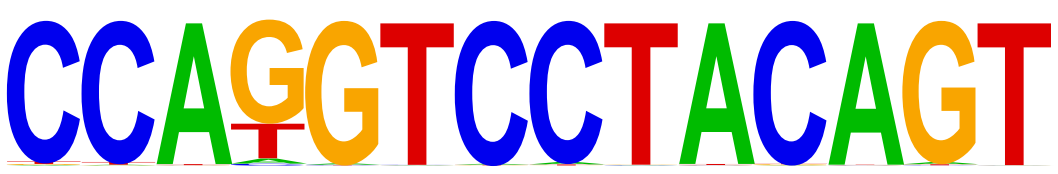	1e-294	-6.782e+02	5.75%	0.00%	130.6bp (35.6bp)	ZmHOX2a(1)(HD-HOX)/ Zea mays/ AthaMap(0.575) More Information | Similar Motifs Found	motif file (matrix)
3	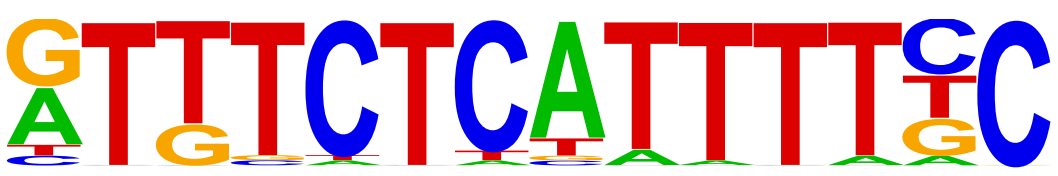	1e-291	-6.705e+02	5.70%	0.01%	132.5bp (181.6bp)	blmp-1/MA0537.1/ Jaspar(0.708) More Information | Similar Motifs Found	motif file (matrix)
4	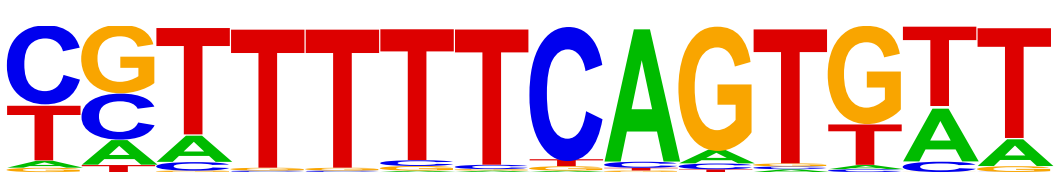	1e-212	-4.904e+02	4.79%	0.01%	138.8bp (41.1bp)	PABP(RRM)/ Drosophila_melanogaster-RNCMPT00139-PBM/ HughesRNA(0.717) More Information | Similar Motifs Found	motif file (matrix)
5	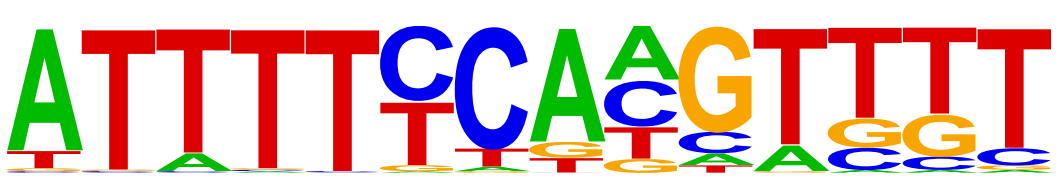	1e-186	-4.299e+02	5.58%	0.03%	139.5bp (120.2bp)	NFAT5/MA0606.1/ Jaspar(0.740) More Information | Similar Motifs Found	motif file (matrix)
6	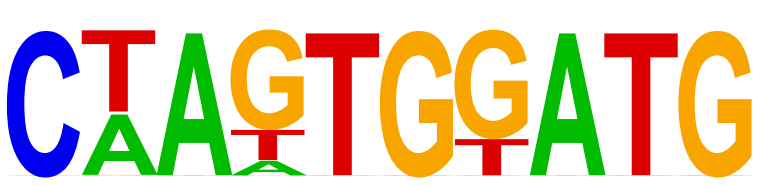	1e-185	-4.268e+02	14.53%	1.18%	140.6bp (154.5bp)	BHLH112/MA0961.1/ Jaspar(0.705) More Information | Similar Motifs Found	motif file (matrix)
7	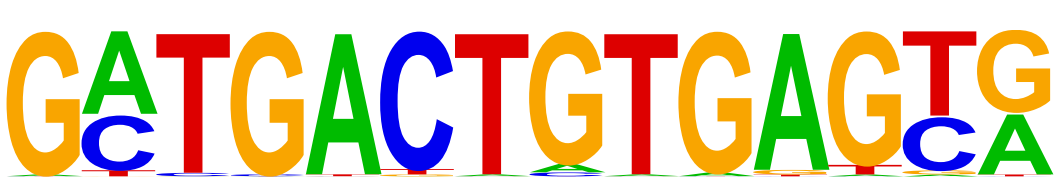	1e-168	-3.891e+02	11.91%	0.78%	140.0bp (150.2bp)	SIZF2/MA1405.1/ Jaspar(0.643) More Information | Similar Motifs Found	motif file (matrix)
8	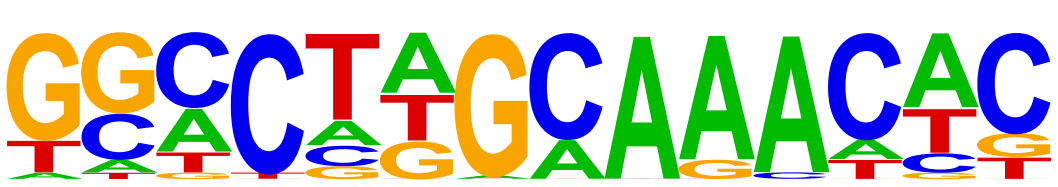	1e-168	-3.872e+02	14.25%	1.32%	137.5bp (146.1bp)	PHA-4(Forkhead)/ cElegans-Embryos-PHA4-ChIP-Seq(modEncode)/ Homer(0.705) More Information | Similar Motifs Found	motif file (matrix)
9	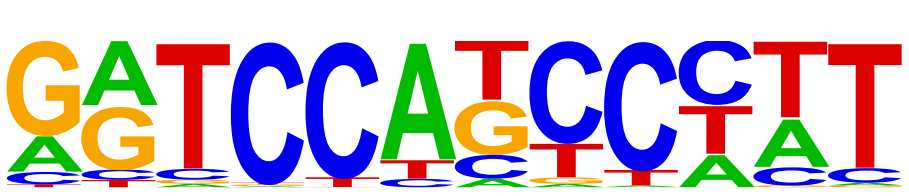	1e-155	-3.580e+02	14.53%	1.58%	140.0bp (143.5bp)	SRSF9(RRM)/ Homo_sapiens-RNCMPT00067-PBM/ HughesRNA(0.715) More Information | Similar Motifs Found	motif file (matrix)
10	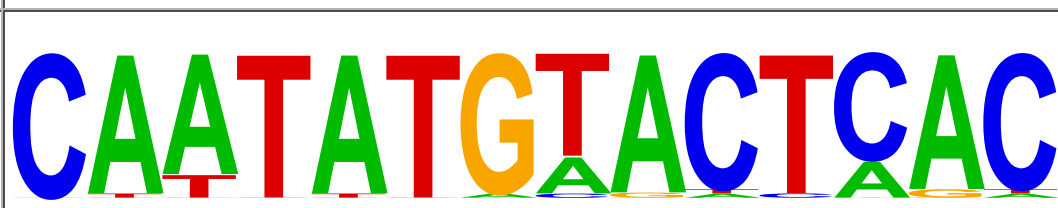	1e-151	-3.485e+02	13.56%	1.37%	133.1bp (134.3bp)	PB0096.1_Zfp187_1/ Jaspar(0.650) More Information | Similar Motifs Found	motif file (matrix)
11	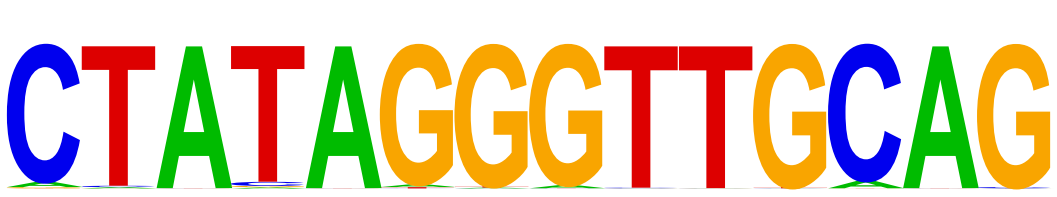	1e-150	-3.464e+02	10.37%	0.65%	128.4bp (140.0bp)	MUB(KH)/ Drosophila_melanogaster-RNCMPT00137-PBM/ HughesRNA(0.674) More Information | Similar Motifs Found	motif file (matrix)

12	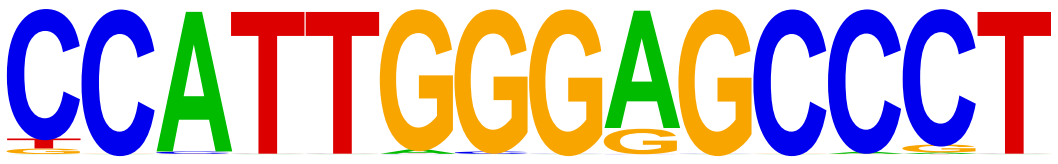	1e-148	-3.415e+02	10.77%	0.75%	139.6bp (149.4bp)	PLAGL2/MA1548.1/ Jaspar(0.617) More Information | Similar Motifs Found	motif file (matrix)
13	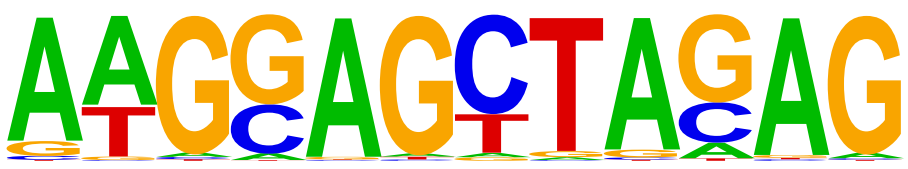	1e-146	-3.369e+02	13.85%	1.53%	120.6bp (137.1bp)	RBM5(RRM)/ Homo_sapiens-RNCMPT00154-PBM/ HughesRNA(0.625) More Information | Similar Motifs Found	motif file (matrix)
14	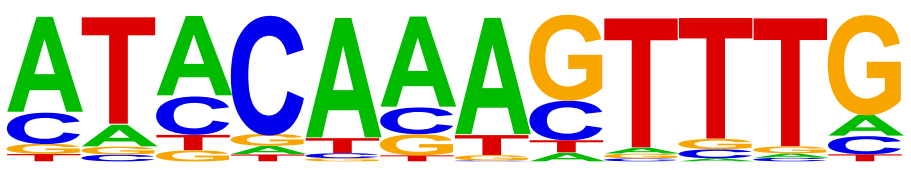	1e-144	-3.336e+02	15.67%	2.12%	121.2bp (138.2bp)	MET31/ Literature(Harbison)/ Yeast(0.723) More Information | Similar Motifs Found	motif file (matrix)
15	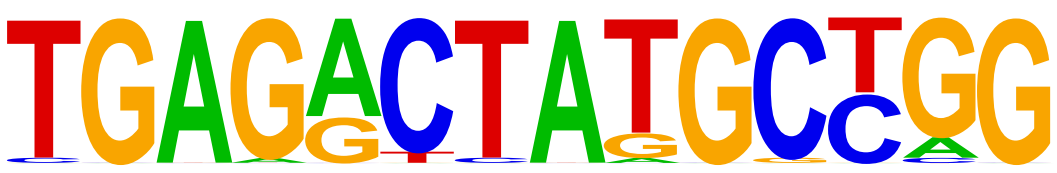	1e-139	-3.212e+02	10.09%	0.69%	145.2bp (149.0bp)	Six4/MA0204.1/ Jaspar(0.568) More Information | Similar Motifs Found	motif file (matrix)
16	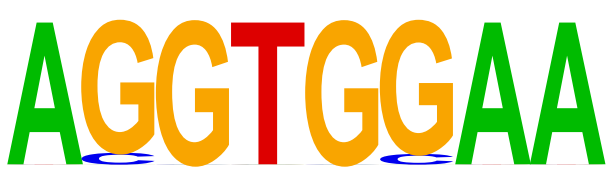	1e-137	-3.162e+02	20.11%	4.00%	141.0bp (387.7bp)	PF10_0068(RRM)/ Plasmodium_falciparum-RNCMPT00199-PBM/ HughesRNA(0.807) More Information | Similar Motifs Found	motif file (matrix)
17	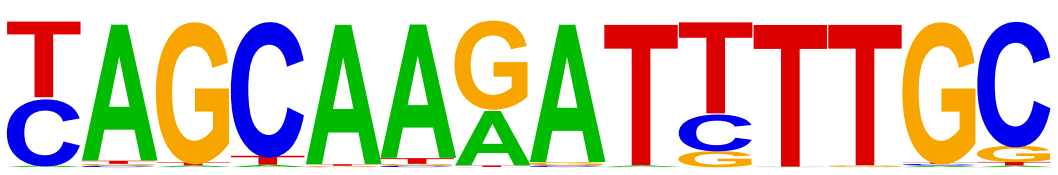	1e-136	-3.136e+02	12.25%	1.24%	138.0bp (144.8bp)	Ng_0261(RRM)/ Naegleria_gruberi-RNCMPT00261-PBM/ HughesRNA(0.651) More Information | Similar Motifs Found	motif file (matrix)
18	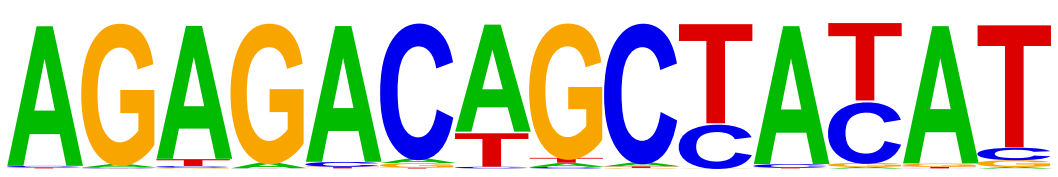	1e-134	-3.086e+02	10.48%	0.83%	138.2bp (142.6bp)	SRSF10(RRM)/ Homo_sapiens-RNCMPT00090-PBM/ HughesRNA(0.659) More Information | Similar Motifs Found	motif file (matrix)
19	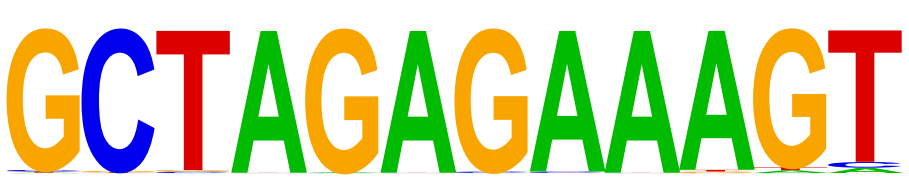	1e-131	-3.028e+02	10.37%	0.84%	139.0bp (147.2bp)	PRDM1/MA0508.3/ Jaspar(0.788) More Information | Similar Motifs Found	motif file (matrix)
20	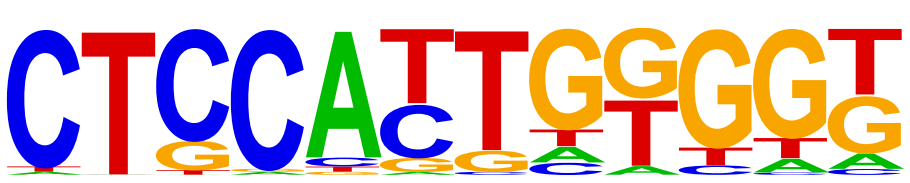	1e-131	-3.018e+02	14.42%	1.98%	144.3bp (146.5bp)	Pp_0237(RRM)/ Physcomitrella_patens-RNCMPT00237-PBM/ HughesRNA(0.702) More Information | Similar Motifs Found	motif file (matrix)
21	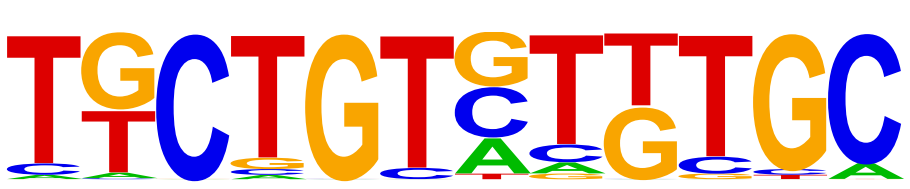	1e-126	-2.918e+02	9.52%	0.70%	141.8bp (146.6bp)	ENOX1(RRM)/ Homo_sapiens-RNCMPT00149-PBM/ HughesRNA(0.714) More Information | Similar Motifs Found	motif file (matrix)
22	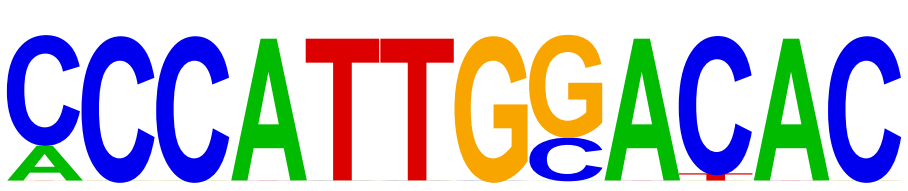	1e-126	-2.903e+02	9.12%	0.62%	135.4bp (153.9bp)	PB0132.1_Hbp1_2/ Jaspar(0.747) More Information | Similar Motifs Found	motif file (matrix)
23	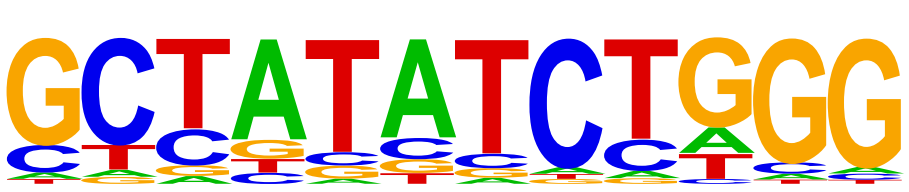	1e-118	-2.720e+02	9.97%	0.91%	138.6bp (144.4bp)	MF0008.1_MADS_class/ Jaspar(0.678) More Information | Similar Motifs Found	motif file (matrix)
24	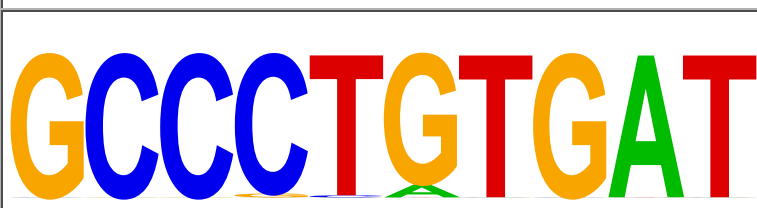	1e-113	-2.615e+02	7.92%	0.50%	142.5bp (146.6bp)	PB0040.1_Lef1_1/ Jaspar(0.700) More Information | Similar Motifs Found	motif file (matrix)

25	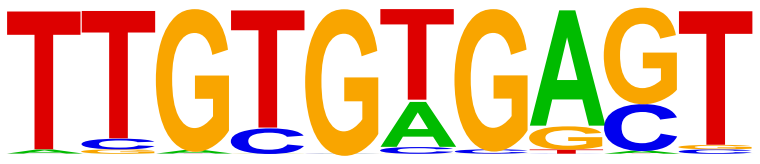	1e-105	-2.439e+02	9.91%	1.06%	147.6bp (145.0bp)	SUP-12(RRM)/ Caenorhabditis_elegans-RNCMPT00179-PBM/ HughesRNA(0.780) More Information | Similar Motifs Found	motif file (matrix)
26	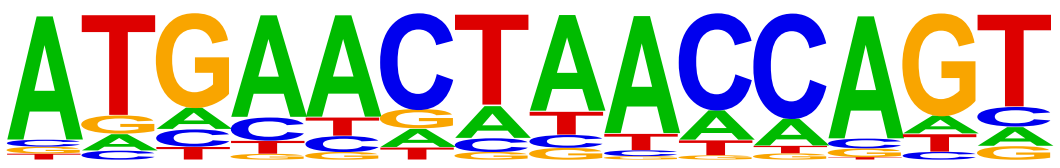	1e-104	-2.417e+02	20.74%	5.51%	147.7bp (146.6bp)	grh/dmmpmm(Bigfoot)/ fly(0.647) More Information | Similar Motifs Found	motif file (matrix)
27	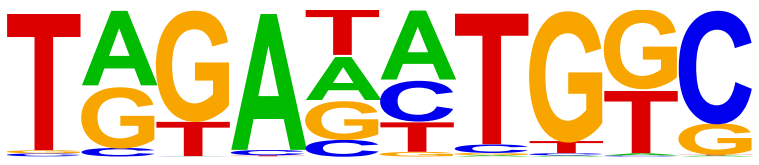	1e-97	-2.252e+02	7.75%	0.63%	133.1bp (143.4bp)	GATA10/MA1013.1/ Jaspar(0.699) More Information | Similar Motifs Found	motif file (matrix)
28	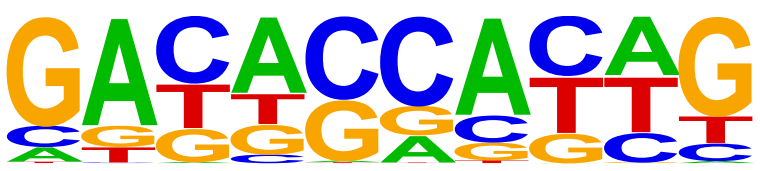	1e-96	-2.217e+02	8.66%	0.87%	134.4bp (308.9bp)	BRU-3(RRM)/ Drosophila_melanogaster-RNCMPT00122-PBM/ HughesRNA(0.643) More Information | Similar Motifs Found	motif file (matrix)
29	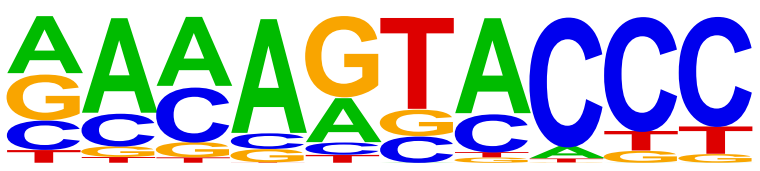	1e-93	-2.144e+02	15.73%	3.55%	133.3bp (317.1bp)	PB0152.1_Nkx3-1_2/ Jaspar(0.675) More Information | Similar Motifs Found	motif file (matrix)
30	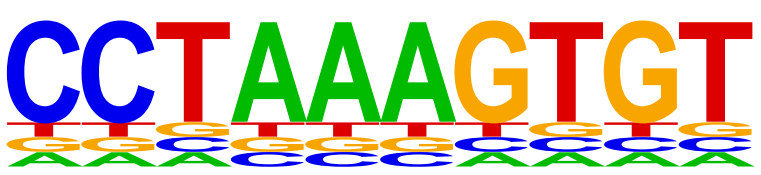	1e-92	-2.141e+02	3.30%	0.04%	138.9bp (112.6bp)	tin/ dmmpmm(SeSiMCMC)/ fly(0.757) More Information | Similar Motifs Found	motif file (matrix)
31	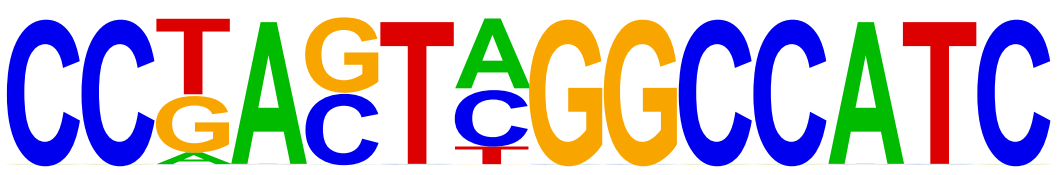	1e-84	-1.953e+02	8.26%	0.94%	109.8bp (141.0bp)	pho/MA1460.1/ Jaspar(0.692) More Information | Similar Motifs Found	motif file (matrix)
32	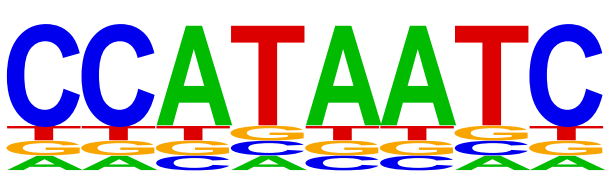	1e-82	-1.902e+02	8.32%	0.99%	130.8bp (139.2bp)	Gsc/ dmmpmm(Noyes_hd)/ fly(0.777) More Information | Similar Motifs Found	motif file (matrix)
33	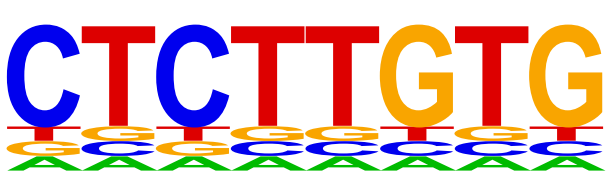	1e-69	-1.593e+02	9.34%	1.64%	145.6bp (139.6bp)	Tb_0217(RRM)/ Trypanosoma_brucei-RNCMPT00217-PBM/ HughesRNA(0.770) More Information | Similar Motifs Found	motif file (matrix)
34	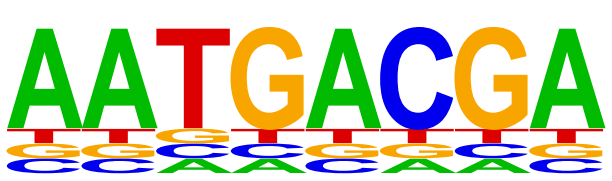	1e-47	-1.097e+02	2.74%	0.12%	130.7bp (157.8bp)	FXR1(KH)/ Homo_sapiens-RNCMPT00161-PBM/ HughesRNA(0.878) More Information | Similar Motifs Found	motif file (matrix)
35	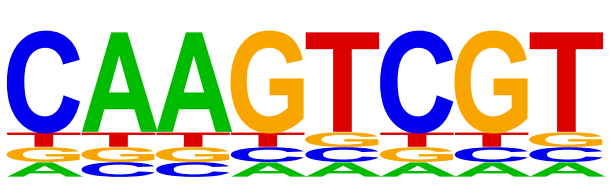	1e-44	-1.031e+02	2.39%	0.09%	125.3bp (212.4bp)	BHLH112/MA0961.1/ Jaspar(0.773) More Information | Similar Motifs Found	motif file (matrix)
36	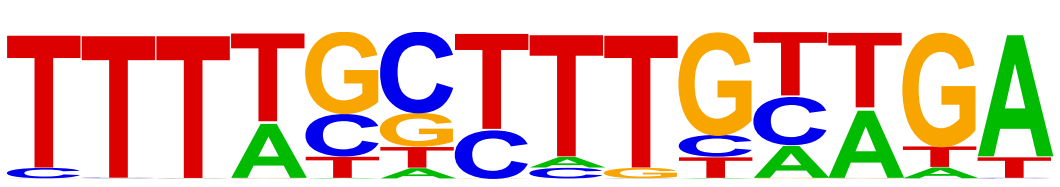	1e-40	-9.231e+01	4.96%	0.78%	131.3bp (139.0bp)	HuR(?)/HEK293-HuR-CLIP-Seq(GSE87887)/ Homer(0.688) More Information | Similar Motifs Found	motif file (matrix)
37	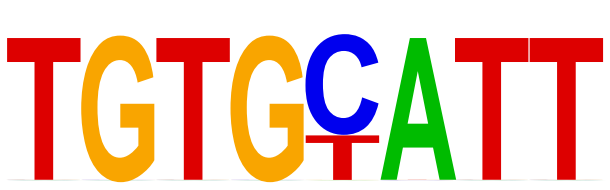	1e-27	-6.224e+01	5.47%	1.43%	129.3bp (171.5bp)	FOXH1/MA0479.1/ Jaspar(0.888) More Information | Similar Motifs Found	motif file (matrix)
38	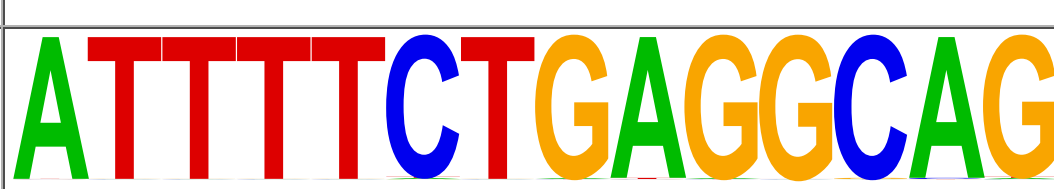	1e-13	-3.041e+01	0.51%	0.01%	119.7bp (78.9bp)	PABPC5(RRM)/ Homo_sapiens-RNCMPT00171-PBM/	motif file (matrix)

							HughesRNA(0.672)<br>More Information | Similar Motifs Found	

Homer Known Motif Enrichment Results (KO_H3K36m2)

Homer *de novo* Motif Results
Gene Ontology Enrichment Results
Known Motif Enrichment Results (txt file)
Total Target Sequences = 1756, Total Background Sequences = 36622

Rank	Motif	Name	P-value	log P-pvalue	q-value (Benjamini)	# Target Sequences with Motif	% of Targets Sequences with Motif	# Background Sequences with Motif	% of Background Sequences with Motif	Motif File	SVG
1	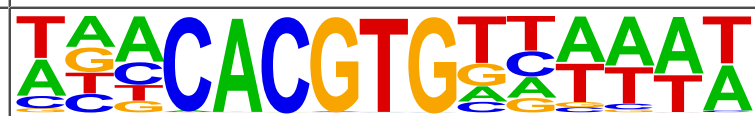	GT3a(Trihelix)/col-GT3a-DAP-Seq(GSE60143)/Homer	1e-24	-5.710e+01	0.0000	121.0	6.89%	842.3	2.30%	motif file (matrix)	svg
2	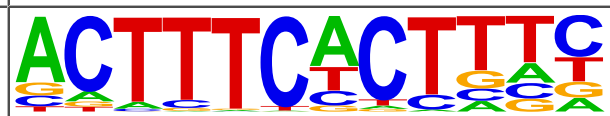	PRDM1(Zf)/Hela-PRDM1-ChIP-Seq(GSE31477)/Homer	1e-23	-5.456e+01	0.0000	316.0	18.01%	3669.6	10.02%	motif file (matrix)	svg
3	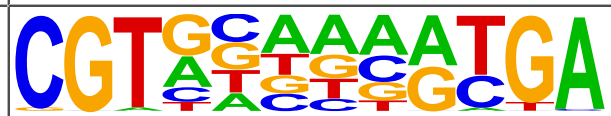	ABF1/SacCer-Promoters/Homer	1e-21	-4.950e+01	0.0000	168.0	9.57%	1542.2	4.21%	motif file (matrix)	svg
4	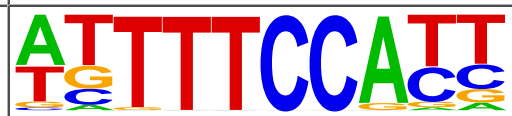	NFAT(RHD)/Jurkat-NFATC1-ChIP-Seq(Jolma_et_al.)/Homer	1e-20	-4.724e+01	0.0000	425.0	24.22%	5696.9	15.55%	motif file (matrix)	svg
5	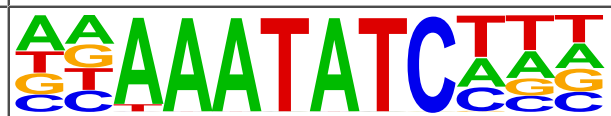	AT3G10113(MYBrelated)/col-AT3G10113-DAP-Seq(GSE60143)/Homer	1e-19	-4.425e+01	0.0000	303.0	17.26%	3714.7	10.14%	motif file (matrix)	svg
6	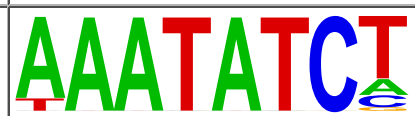	RVE1(MYBrelated)/col-RVE1-DAP-Seq(GSE60143)/Homer	1e-18	-4.240e+01	0.0000	337.0	19.20%	4332.2	11.82%	motif file (matrix)	svg
7	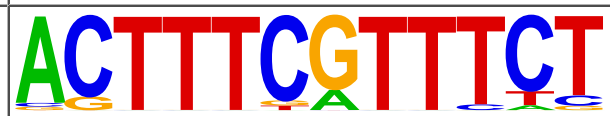	T1ISRE(IRF)/ThioMac-Ifnb-Expression/Homer	1e-16	-3.851e+01	0.0000	40.0	2.28%	151.4	0.41%	motif file (matrix)	svg
8	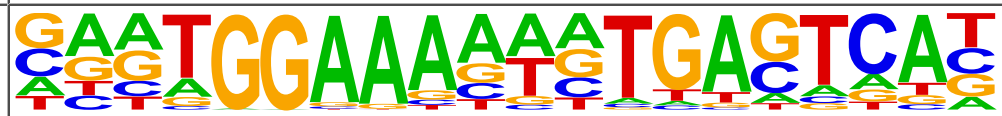	NFAT:AP1(RHD,bZIP)/Jurkat-NFATC1-ChIP-Seq(Jolma_et_al.)/Homer	1e-15	-3.560e+01	0.0000	107.0	6.10%	924.4	2.52%	motif file (matrix)	svg
9	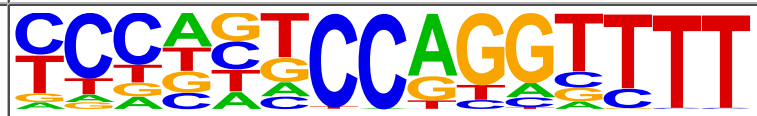	PRDM15(Zf)/ESC-Prdm15-ChIP-Seq(GSE73694)/Homer	1e-15	-3.514e+01	0.0000	458.0	26.10%	6712.0	18.32%	motif file (matrix)	svg
10	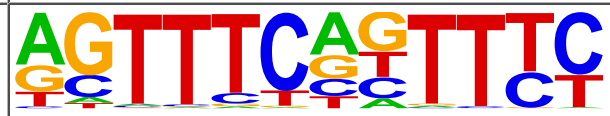	IRF3(IRF)/BMDM-Irf3-ChIP-Seq(GSE67343)/Homer	1e-14	-3.269e+01	0.0000	181.0	10.31%	2051.9	5.60%	motif file (matrix)	svg
11	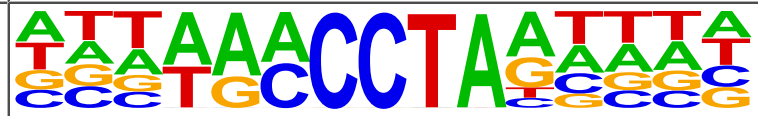	AT1G72740(MYBrelated)/colamp-AT1G72740-DAP-Seq(GSE60143)/Homer	1e-13	-3.115e+01	0.0000	405.0	23.08%	5908.6	16.13%	motif file (matrix)	svg
12	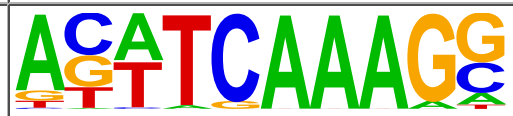	Tcf3(HMG)/mES-Tcf3-ChIP-Seq(GSE11724)/Homer	1e-13	-3.037e+01	0.0000	149.0	8.49%	1613.1	4.40%	motif file (matrix)	svg
13	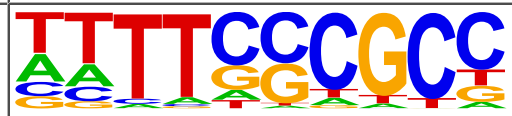	DEL2(E2FDP)/col-DEL2-DAP-Seq(GSE60143)/Homer	1e-12	-2.924e+01	0.0000	268.0	15.27%	3568.5	9.74%	motif file (matrix)	svg
14	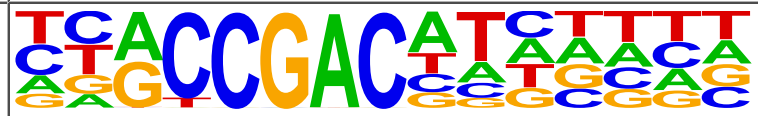	CBF2(AP2EREBP)/colamp-CBF2-DAP-Seq(GSE60143)/Homer	1e-12	-2.902e+01	0.0000	260.0	14.81%	3439.2	9.39%	motif file (matrix)	svg
15	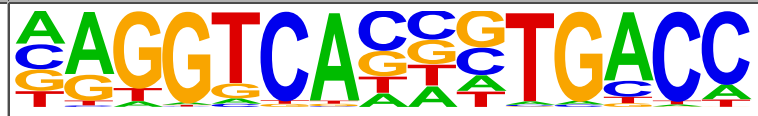	ERE(NR),IR3/MCF7-ERa-ChIP-Seq(Unpublished)/Homer	1e-12	-2.804e+01	0.0000	131.0	7.46%	1392.3	3.80%	motif file (matrix)	svg
16	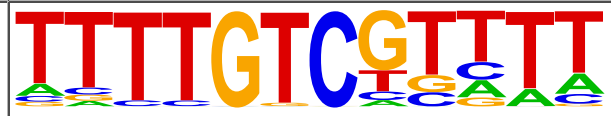	JKD(C2H2)/col-JKD-DAP-Seq(GSE60143)/Homer	1e-11	-2.573e+01	0.0000	115.0	6.55%	1202.6	3.28%	motif file (matrix)	svg
17	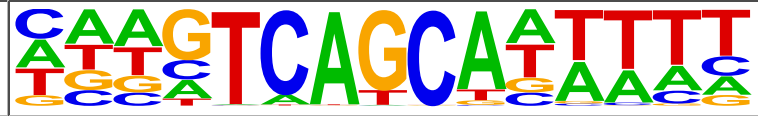	MafF(bZIP)/HepG2-MafF-ChIP-Seq(GSE31477)/Homer	1e-10	-2.365e+01	0.0000	136.0	7.75%	1566.4	4.28%	motif file (matrix)	svg
18	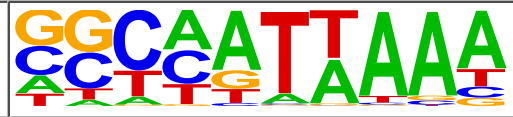	Unknown(Homeobox)/Limb-p300-ChIP-Seq/Homer	1e-10	-2.360e+01	0.0000	300.0	17.09%	4331.0	11.82%	motif file (matrix)	svg
19	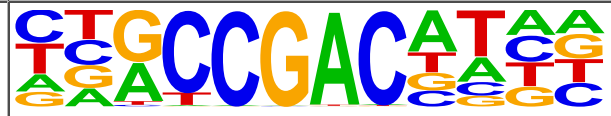	CBF3(AP2EREBP)/colamp-CBF3-DAP-Seq(GSE60143)/Homer	1e-9	-2.267e+01	0.0000	259.0	14.76%	3644.7	9.95%	motif file (matrix)	svg
20	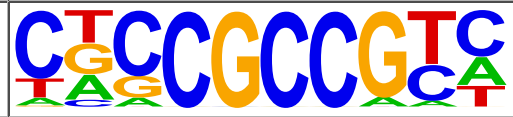	LEP(AP2EREBP)/col-LEP-DAP-Seq(GSE60143)/Homer	1e-9	-2.097e+01	0.0000	648.0	36.92%	11049.3	30.16%	motif file (matrix)	svg
21	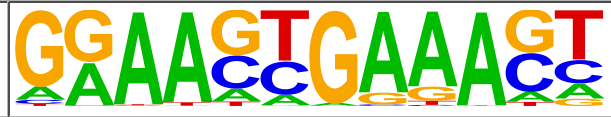	IRF8(IRF)/BMDM-IRF8-ChIP-Seq(GSE77884)/Homer	1e-9	-2.075e+01	0.0000	167.0	9.52%	2143.8	5.85%	motif file (matrix)	svg
22	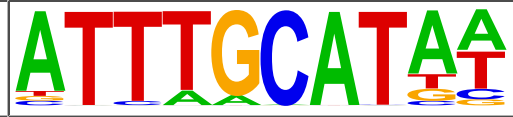	Oct4(POU,Homeobox)/mES-Oct4-ChIP-Seq(GSE11431)/Homer	1e-8	-2.060e+01	0.0000	226.0	12.88%	3148.3	8.59%	motif file (matrix)	svg
23	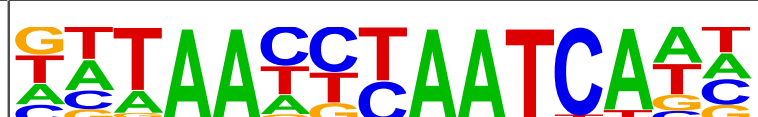	DUX4(Homeobox)/Myoblasts-DUX4.V5-ChIP-Seq(GSE75791)/Homer	1e-8	-1.996e+01	0.0000	53.0	3.02%	437.0	1.19%	motif file (matrix)	svg
24	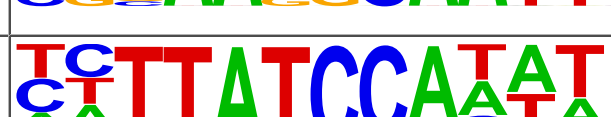	At1g74840(MYBrelated)/col100-At1g74840-DAP-Seq(GSE60143)/Homer	1e-8	-1.929e+01	0.0000	315.0	17.95%	4789.1	13.07%	motif file (matrix)	svg
25	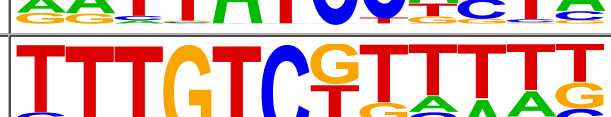	At5g66730(C2H2)/colamp-At5g66730-DAP-Seq(GSE60143)/Homer	1e-8	-1.923e+01	0.0000	135.0	7.69%	1666.0	4.55%	motif file (matrix)	svg
26	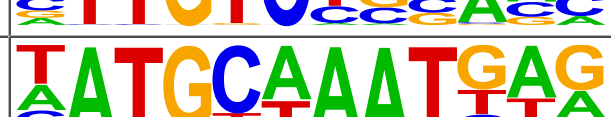	Oct6(POU,Homeobox)/NPC-Pou3f1-ChIP-Seq(GSE35496)/Homer	1e-8	-1.868e+01	0.0000	212.0	12.08%	2978.4	8.13%	motif file (matrix)	svg
27	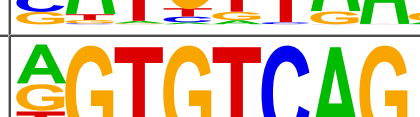	CELF2(RRM)/JSL1-CELF2-CLIP-Seq(GSE71264)/Homer	1e-7	-1.786e+01	0.0000	173.0	9.86%	2338.2	6.38%	motif file (matrix)	svg
28	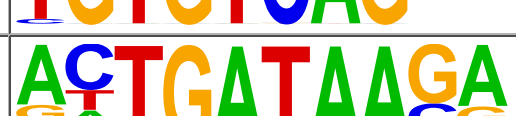	PQM-1(?)/cElegans-L3-ChIP-Seq(modEncode)/Homer	1e-7	-1.754e+01	0.0000	163.0	9.29%	2180.2	5.95%	motif file (matrix)	svg

29	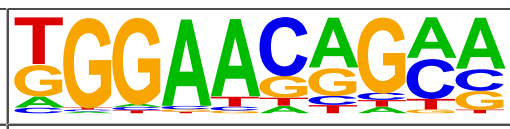	ZNF189(Zf)/HEK293-ZNF189.GFP-ChIP-Seq(GSE58341)/Homer	1e-7	-1.684e+01	0.0000	350.0	19.94%	5560.1	15.18%	motif file (matrix)	svg
30	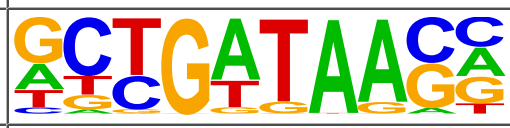	Unknown5/Drosophila-Promoters/Homer	1e-7	-1.681e+01	0.0000	226.0	12.88%	3300.3	9.01%	motif file (matrix)	svg
31	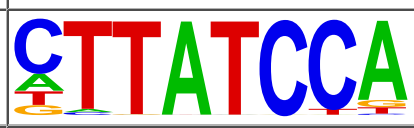	At5g47390(MYBrelated)/col-At5g47390-DAP-Seq(GSE60143)/Homer	1e-7	-1.659e+01	0.0000	455.0	25.93%	7565.6	20.65%	motif file (matrix)	svg
32	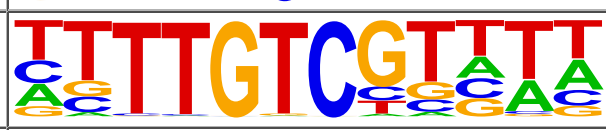	MGP(C2H2)/colamp-MGP-DAP-Seq(GSE60143)/Homer	1e-7	-1.653e+01	0.0000	115.0	6.55%	1419.5	3.87%	motif file (matrix)	svg
33	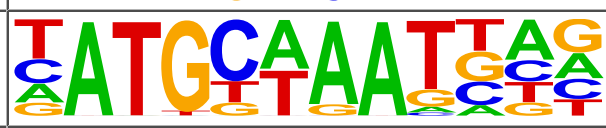	Brn1(POU,Homeobox)/NPC-Brn1-ChIP-Seq(GSE35496)/Homer	1e-7	-1.642e+01	0.0000	161.0	9.17%	2185.3	5.96%	motif file (matrix)	svg
34	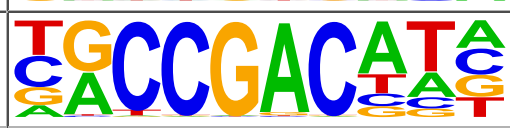	CBF1(AP2EREBP)/colamp-CBF1-DAP-Seq(GSE60143)/Homer	1e-7	-1.626e+01	0.0000	374.0	21.31%	6043.3	16.50%	motif file (matrix)	svg
35	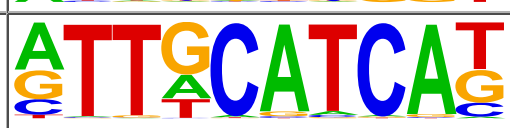	Chop(bZIP)/MEF-Chop-ChIP-Seq(GSE35681)/Homer	1e-6	-1.523e+01	0.0000	84.0	4.79%	964.3	2.63%	motif file (matrix)	svg
36	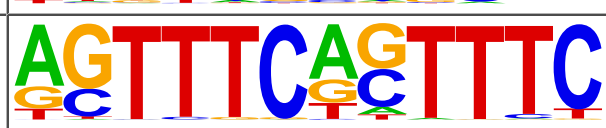	ISRE(IRF)/ThioMac-LPS-Expression(GSE23622)/Homer	1e-6	-1.509e+01	0.0000	48.0	2.74%	440.7	1.20%	motif file (matrix)	svg
37	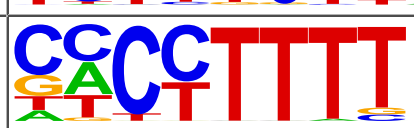	AT5G02460(C2C2dof)/col-AT5G02460-DAP-Seq(GSE60143)/Homer	1e-6	-1.478e+01	0.0000	711.0	40.51%	12750.2	34.80%	motif file (matrix)	svg
38	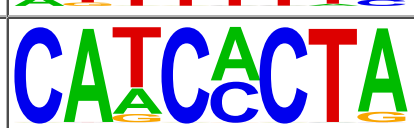	Unknown2/Drosophila-Promoters/Homer	1e-6	-1.419e+01	0.0000	455.0	25.93%	7723.1	21.08%	motif file (matrix)	svg
39	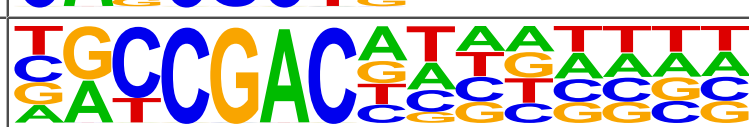	CBF4(AP2EREBP)/colamp-CBF4-DAP-Seq(GSE60143)/Homer	1e-6	-1.389e+01	0.0000	372.0	21.20%	6149.9	16.79%	motif file (matrix)	svg
40	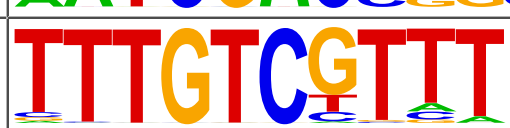	AtIDD11(C2H2)/colamp-AtIDD11-DAP-Seq(GSE60143)/Homer	1e-6	-1.388e+01	0.0000	148.0	8.43%	2056.8	5.61%	motif file (matrix)	svg
41	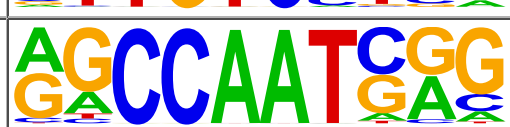	NFY(CCAAT)/Promoter/Homer	1e-6	-1.384e+01	0.0000	365.0	20.80%	6019.3	16.43%	motif file (matrix)	svg
42	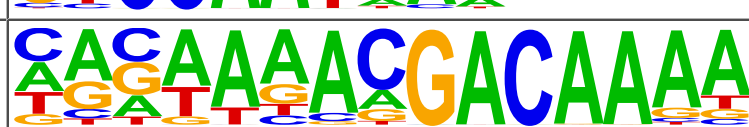	NUC(C2H2)/col-NUC-DAP-Seq(GSE60143)/Homer	1e-5	-1.376e+01	0.0000	61.0	3.48%	649.4	1.77%	motif file (matrix)	svg
43	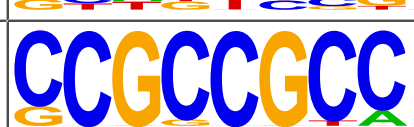	SHN3(AP2EREBP)/col-SHN3-DAP-Seq(GSE60143)/Homer	1e-5	-1.363e+01	0.0000	749.0	42.68%	13616.8	37.17%	motif file (matrix)	svg
44	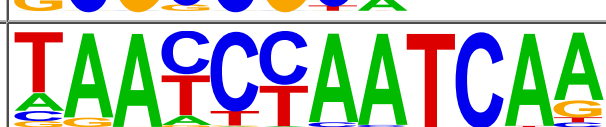	Duxbl(Homeobox)/NIH3T3-Duxbl.HA-ChIP-Seq(GSE119782)/Homer	1e-5	-1.330e+01	0.0000	49.0	2.79%	484.0	1.32%	motif file (matrix)	svg
45	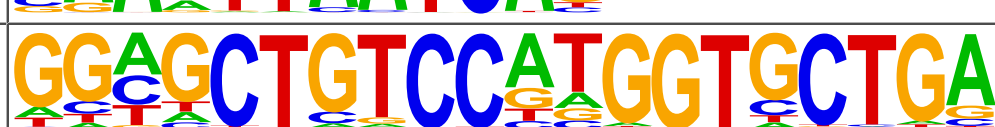	REST-NRSF(Zf)/Jurkat-NRSF-ChIP-Seq/Homer	1e-5	-1.291e+01	0.0001	7.0	0.40%	12.5	0.03%	motif file (matrix)	svg
46	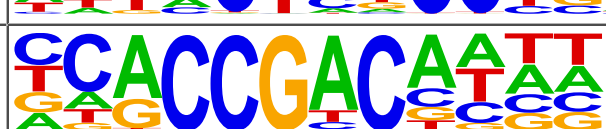	RAP21(AP2EREBP)/colamp-RAP21-DAP-Seq(GSE60143)/Homer	1e-5	-1.261e+01	0.0001	82.0	4.67%	1000.5	2.73%	motif file (matrix)	svg
47	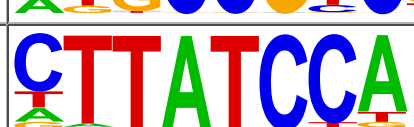	AT5G61620(MYBrelated)/colamp-AT5G61620-DAP-Seq(GSE60143)/Homer	1e-5	-1.253e+01	0.0001	478.0	27.24%	8292.3	22.63%	motif file (matrix)	svg
48	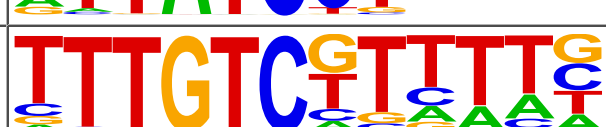	IDD7(C2H2)/col-IDD7-DAP-Seq(GSE60143)/Homer	1e-5	-1.251e+01	0.0001	159.0	9.06%	2303.0	6.29%	motif file (matrix)	svg
49	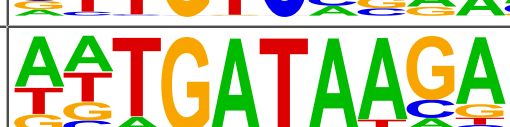	ELT-3(Gata)/cElegans-L1-ELT3-ChIP-Seq(modEncode)/Homer	1e-5	-1.230e+01	0.0001	198.0	11.28%	3006.4	8.21%	motif file (matrix)	svg
50	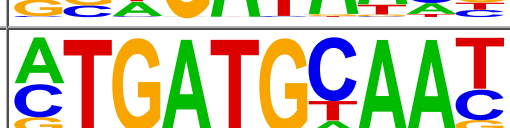	Atf4(bZIP)/MEF-Atf4-ChIP-Seq(GSE35681)/Homer	1e-5	-1.206e+01	0.0001	95.0	5.41%	1227.1	3.35%	motif file (matrix)	svg
51	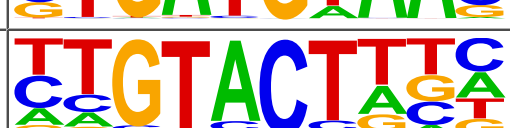	SPL11(SBP)/col100-SPL11-DAP-Seq(GSE60143)/Homer	1e-5	-1.195e+01	0.0001	226.0	12.88%	3533.8	9.65%	motif file (matrix)	svg
52	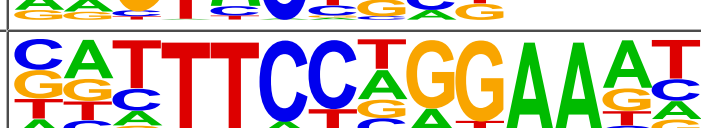	STAT1(Stat)/HelaS3-STAT1-ChIP-Seq(GSE12782)/Homer	1e-5	-1.194e+01	0.0001	121.0	6.89%	1667.3	4.55%	motif file (matrix)	svg
53	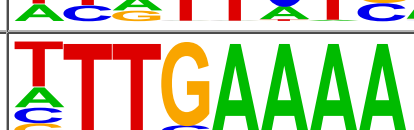	At2g41835(C2H2)/col-At2g41835-DAP-Seq(GSE60143)/Homer	1e-5	-1.187e+01	0.0001	210.0	11.97%	3245.6	8.86%	motif file (matrix)	svg
54	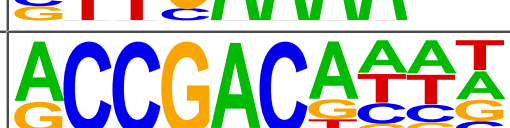	AT1G77200(AP2EREBP)/colamp-AT1G77200-DAP-Seq(GSE60143)/Homer	1e-5	-1.169e+01	0.0002	406.0	23.13%	6953.4	18.98%	motif file (matrix)	svg
55	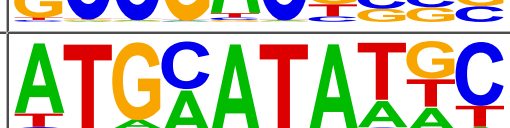	Pit1(Homeobox)/GCrat-Pit1-ChIP-Seq(GSE58009)/Homer	1e-5	-1.156e+01	0.0002	399.0	22.74%	6827.7	18.64%	motif file (matrix)	svg
56	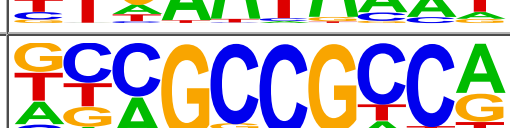	ERF5(AP2EREBP)/colamp-ERF5-DAP-Seq(GSE60143)/Homer	1e-4	-1.110e+01	0.0003	705.0	40.17%	12948.6	35.34%	motif file (matrix)	svg
57	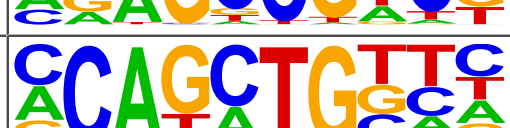	Twist2(bHLH)/Myoblast-Twist2.Ty1-ChIP-Seq(GSE127998)/Homer	1e-4	-1.102e+01	0.0003	624.0	35.56%	11318.1	30.89%	motif file (matrix)	svg
58	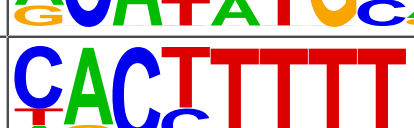	At1g64620(C2C2dof)/colamp-At1g64620-DAP-Seq(GSE60143)/Homer	1e-4	-1.025e+01	0.0006	477.0	27.18%	8457.3	23.08%	motif file (matrix)	svg
59	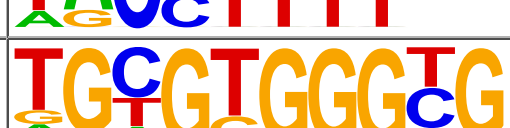	Egr1(Zf)/K562-Egr1-ChIP-Seq(GSE32465)/Homer	1e-4	-9.939e+00	0.0008	572.0	32.59%	10374.2	28.32%	motif file (matrix)	svg
60	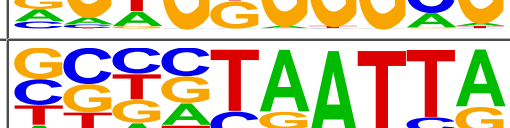	DLX1(Homeobox)/BasalGanglia-Dlx1-ChIP-seq(GSE124936)/Homer	1e-4	-9.850e+00	0.0009	425.0	24.22%	7469.0	20.39%	motif file (matrix)	svg
61	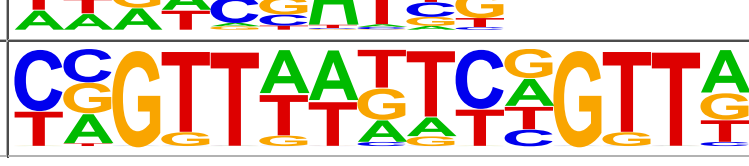	MYB44(MYB)/colamp-MYB44-DAP-Seq(GSE60143)/Homer	1e-4	-9.685e+00	0.0010	53.0	3.02%	620.4	1.69%	motif file (matrix)	svg

62	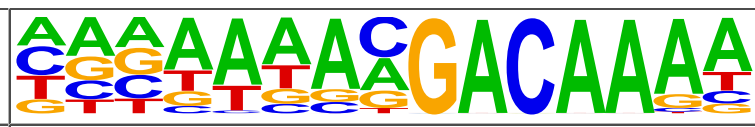	IDD2(C2H2)/colamp-IDD2-DAP-Seq(GSE60143)/Homer	1e-4	-9.579e+00	0.0011	72.0	4.10%	927.3	2.53%	motif file (matrix)	svg
63	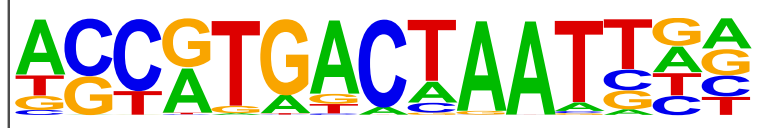	PAX3:FKHR-fusion(Paired,Homeobox)/Rh4-PAX3:FKHR-ChIP-Seq(GSE19063)/Homer	1e-4	-9.452e+00	0.0013	98.0	5.58%	1368.2	3.73%	motif file (matrix)	svg
64	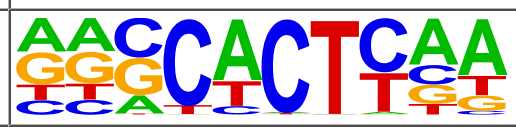	Nkx2.5(Homeobox)/HL1-Nkx2.5.biotin-ChIP-Seq(GSE21529)/Homer	1e-4	-9.354e+00	0.0014	687.0	39.15%	12754.7	34.81%	motif file (matrix)	svg
65	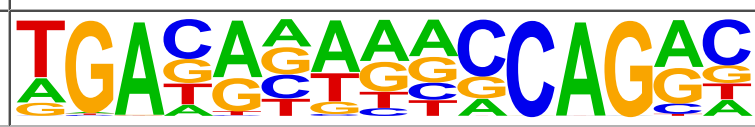	Hand2(bHLH)/Mesoderm-Hand2-ChIP-Seq(GSE61475)/Homer	1e-4	-9.331e+00	0.0014	179.0	10.20%	2816.2	7.69%	motif file (matrix)	svg
66	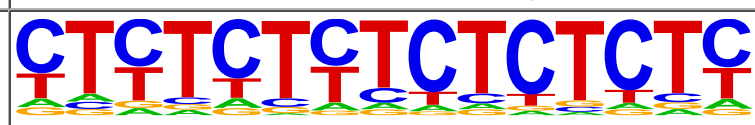	GAGA-repeat/SacCer-Promoters/Homer	1e-4	-9.304e+00	0.0014	1156.0	65.87%	22541.3	61.53%	motif file (matrix)	svg
67	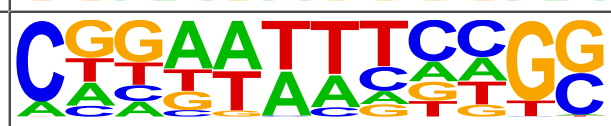	LBD18(LOBAS2)/colamp-LBD18-DAP-Seq(GSE60143)/Homer	1e-3	-9.103e+00	0.0017	760.0	43.30%	14272.3	38.96%	motif file (matrix)	svg
68	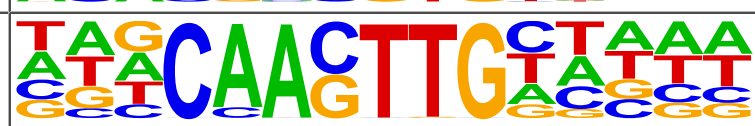	bHLH122(bHLH)/col100-bHLH122-DAP-Seq(GSE60143)/Homer	1e-3	-9.072e+00	0.0017	282.0	16.07%	4762.6	13.00%	motif file (matrix)	svg
69	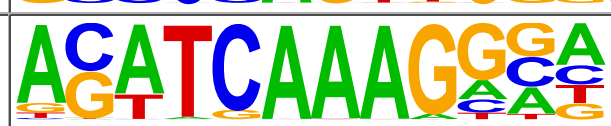	Tcf4(HMG)/Hct116-Tcf4-ChIP-Seq(SRA012054)/Homer	1e-3	-8.947e+00	0.0019	177.0	10.09%	2801.7	7.65%	motif file (matrix)	svg
70	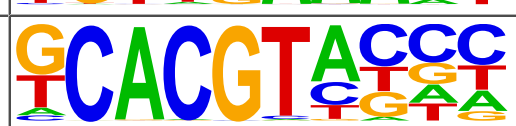	HIF2a(bHLH)/785_O-HIF2a-ChIP-Seq(GSE34871)/Homer	1e-3	-8.917e+00	0.0019	165.0	9.40%	2583.9	7.05%	motif file (matrix)	svg
71	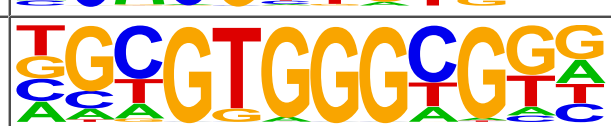	Egr2(Zf)/Thymocytes-Egr2-ChIP-Seq(GSE34254)/Homer	1e-3	-8.882e+00	0.0020	260.0	14.81%	4357.1	11.89%	motif file (matrix)	svg
72	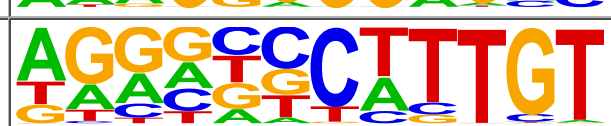	Sox9(HMG)/Limb-SOX9-ChIP-Seq(GSE73225)/Homer	1e-3	-8.833e+00	0.0020	299.0	17.04%	5105.4	13.94%	motif file (matrix)	svg
73	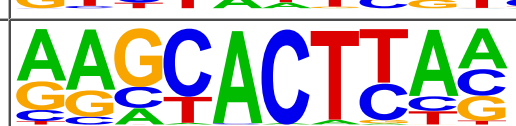	Nkx3.1(Homeobox)/LNCaP-Nkx3.1-ChIP-Seq(GSE28264)/Homer	1e-3	-8.777e+00	0.0021	708.0	40.34%	13243.6	36.15%	motif file (matrix)	svg
74	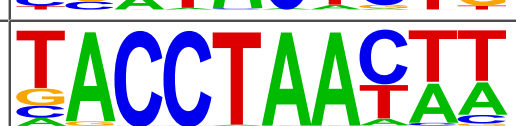	MYB27(MYB)/colamp-MYB27-DAP-Seq(GSE60143)/Homer	1e-3	-8.681e+00	0.0023	76.0	4.33%	1023.6	2.79%	motif file (matrix)	svg
75	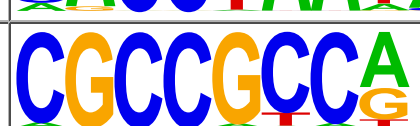	CRF4(AP2EREBP)/colamp-CRF4-DAP-Seq(GSE60143)/Homer	1e-3	-8.576e+00	0.0025	775.0	44.16%	14638.2	39.95%	motif file (matrix)	svg
76	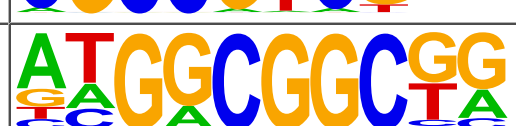	AT4G18450(AP2EREBP)/col-AT4G18450-DAP-Seq(GSE60143)/Homer	1e-3	-8.506e+00	0.0027	658.0	37.49%	12254.2	33.45%	motif file (matrix)	svg
77	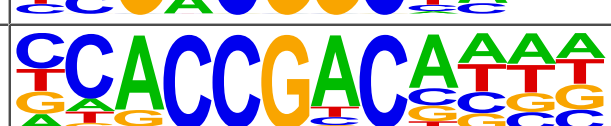	At1g22810(AP2EREBP)/colamp-At1g22810-DAP-Seq(GSE60143)/Homer	1e-3	-8.290e+00	0.0033	234.0	13.33%	3906.1	10.66%	motif file (matrix)	svg
78	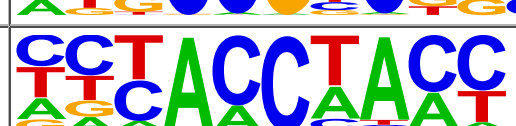	MYB61(MYB)/colamp-MYB61-DAP-Seq(GSE60143)/Homer	1e-3	-8.269e+00	0.0033	463.0	26.38%	8360.3	22.82%	motif file (matrix)	svg
79	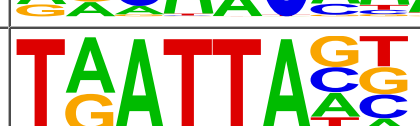	ATHB25(ZFHD)/colamp-ATHB25-DAP-Seq(GSE60143)/Homer	1e-3	-8.015e+00	0.0042	358.0	20.40%	6315.2	17.24%	motif file (matrix)	svg
80	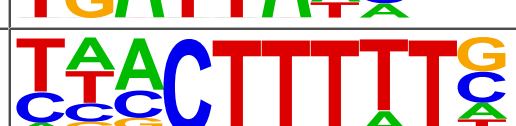	At3g45610(C2C2dof)/col-At3g45610-DAP-Seq(GSE60143)/Homer	1e-3	-7.976e+00	0.0043	520.0	29.63%	9526.0	26.00%	motif file (matrix)	svg
81	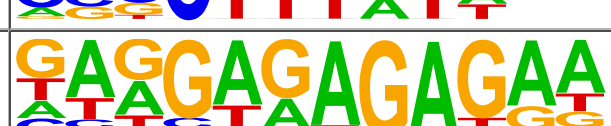	BPC1(BBRBPC)/colamp-BPC1-DAP-Seq(GSE60143)/Homer	1e-3	-7.872e+00	0.0047	248.0	14.13%	4202.7	11.47%	motif file (matrix)	svg
82	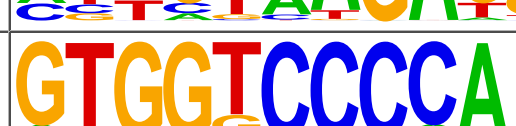	TCP17(TCP)/col-TCP17-DAP-Seq(GSE60143)/Homer	1e-3	-7.822e+00	0.0049	24.0	1.37%	228.0	0.62%	motif file (matrix)	svg
83	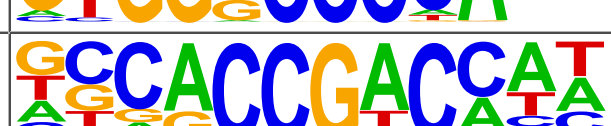	At4g28140(AP2EREBP)/colamp-At4g28140-DAP-Seq(GSE60143)/Homer	1e-3	-7.810e+00	0.0049	220.0	12.54%	3675.1	10.03%	motif file (matrix)	svg
84	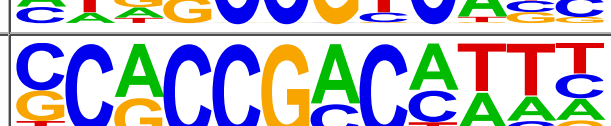	At5g65130(AP2EREBP)/colamp-At5g65130-DAP-Seq(GSE60143)/Homer	1e-3	-7.758e+00	0.0051	236.0	13.45%	3982.5	10.87%	motif file (matrix)	svg
85	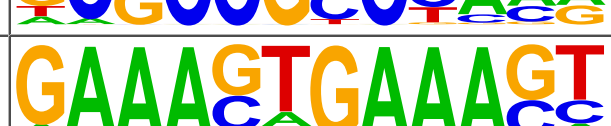	IRF1(IRF)/PBMC-IRF1-ChIP-Seq(GSE43036)/Homer	1e-3	-7.619e+00	0.0058	77.0	4.39%	1079.5	2.95%	motif file (matrix)	svg
86	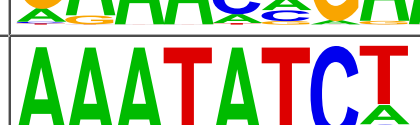	EPR1(MYBrelated)/colamp-EPR1-DAP-Seq(GSE60143)/Homer	1e-3	-7.473e+00	0.0066	175.0	9.97%	2853.1	7.79%	motif file (matrix)	svg
87	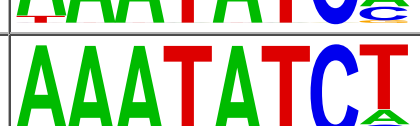	LHY1(MYBrelated)/col-LHY1-DAP-Seq(GSE60143)/Homer	1e-3	-7.473e+00	0.0066	175.0	9.97%	2853.1	7.79%	motif file (matrix)	svg
88	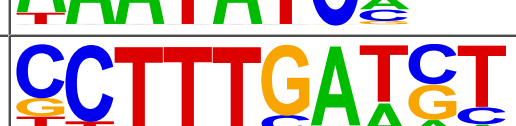	LEF1(HMG)/H1-LEF1-ChIP-Seq(GSE64758)/Homer	1e-3	-7.327e+00	0.0075	244.0	13.90%	4167.6	11.38%	motif file (matrix)	svg
89	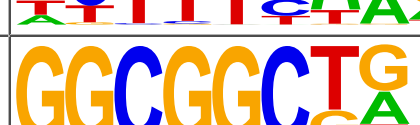	ERF1(AP2EREBP)/colamp-ERF1-DAP-Seq(GSE60143)/Homer	1e-3	-7.085e+00	0.0095	812.0	46.27%	15578.5	42.52%	motif file (matrix)	svg
90	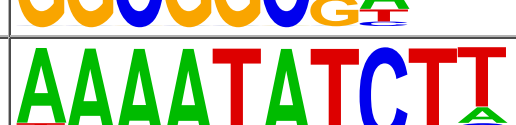	At3g09600(MYBrelated)/colamp-At3g09600-DAP-Seq(GSE60143)/Homer	1e-2	-6.801e+00	0.0124	216.0	12.31%	3673.2	10.03%	motif file (matrix)	svg
91	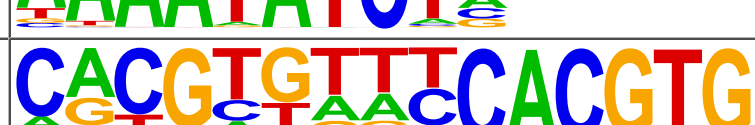	bHLH18(bHLH)/col-bHLH18-DAP-Seq(GSE60143)/Homer	1e-2	-6.766e+00	0.0127	12.0	0.68%	86.2	0.24%	motif file (matrix)	svg
92	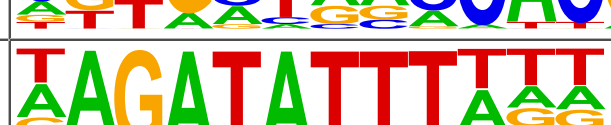	AT2G28920(ND)/col-AT2G28920-DAP-Seq(GSE60143)/Homer	1e-2	-6.733e+00	0.0130	144.0	8.21%	2324.6	6.34%	motif file (matrix)	svg
93	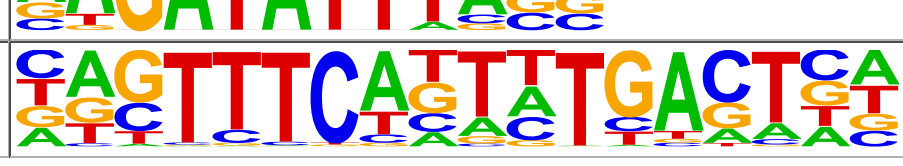	bZIP:IRF(bZIP,IRF)/Th17-BatF-ChIP-Seq(GSE39756)/Homer	1e-2	-6.650e+00	0.0140	174.0	9.91%	2889.5	7.89%	motif file (matrix)	svg

94	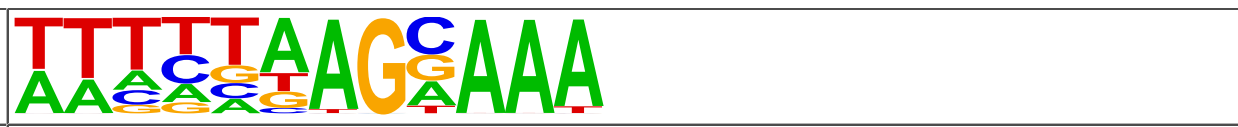	AT5G60130(ABI3VP1)/col-AT5G60130-DAP-Seq(GSE60143)/Homer	1e-2	-6.641e+00	0.0140	569.0	32.42%	10661.3	29.10%	motif file (matrix)	svg
95	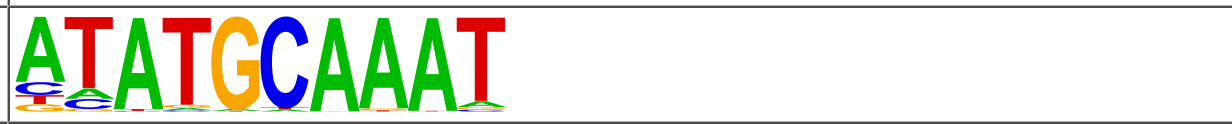	Oct2(POU,Homeobox)/Bcell-Oct2-ChIP-Seq(GSE21512)/Homer	1e-2	-6.620e+00	0.0141	142.0	8.09%	2294.6	6.26%	motif file (matrix)	svg
96	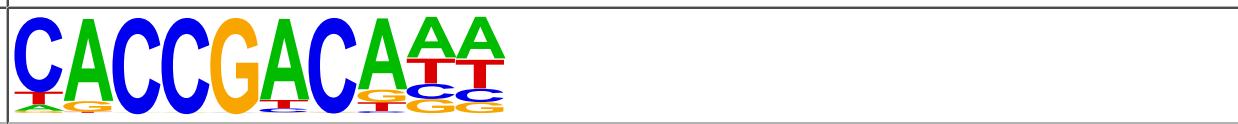	At4g31060(AP2EREBP)/colamp-At4g31060-DAP-Seq(GSE60143)/Homer	1e-2	-6.606e+00	0.0142	156.0	8.89%	2556.0	6.98%	motif file (matrix)	svg
97	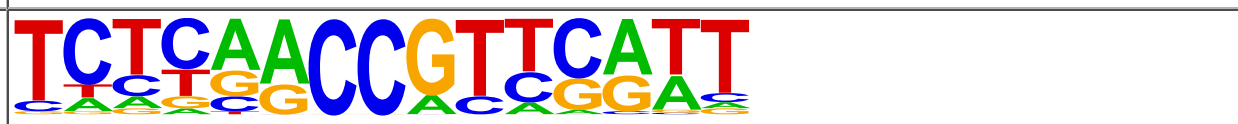	AT5G59990(C2C2COlike)/colamp-AT5G59990-DAP-Seq(GSE60143)/Homer	1e-2	-6.542e+00	0.0149	16.0	0.91%	139.5	0.38%	motif file (matrix)	svg
98	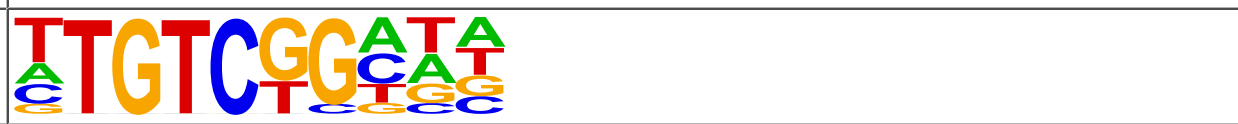	ARF2(ARF)/col-ARF2-DAP-Seq(GSE60143)/Homer	1e-2	-6.493e+00	0.0155	938.0	53.45%	18275.5	49.88%	motif file (matrix)	svg
99	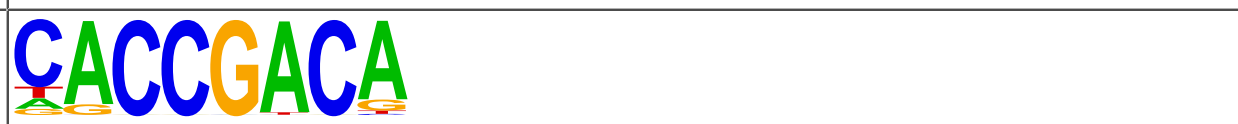	ERF38(AP2EREBP)/col-ERF38-DAP-Seq(GSE60143)/Homer	1e-2	-6.485e+00	0.0155	241.0	13.73%	4177.1	11.40%	motif file (matrix)	svg
100	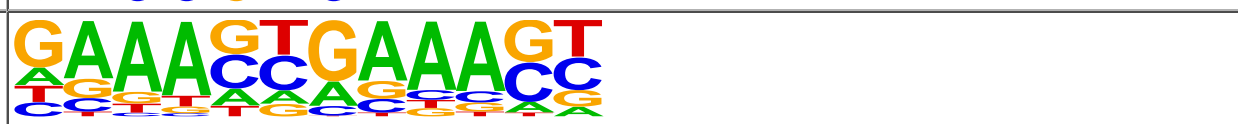	IRF2(IRF)/Erythroblas-IRF2-ChIP-Seq(GSE36985)/Homer	1e-2	-6.446e+00	0.0160	49.0	2.79%	648.6	1.77%	motif file (matrix)	svg
101	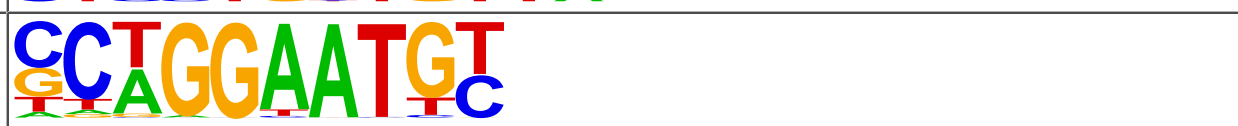	TEAD2(TEA)/Py2T-Tead2-ChIP-Seq(GSE55709)/Homer	1e-2	-6.175e+00	0.0207	195.0	11.11%	3321.7	9.07%	motif file (matrix)	svg
102	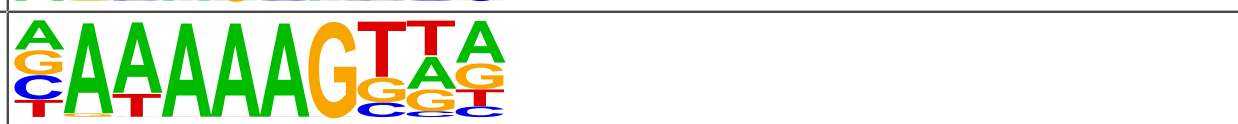	BBX31(Orphan)/col-BBX31-DAP-Seq(GSE60143)/Homer	1e-2	-6.101e+00	0.0221	501.0	28.55%	9353.4	25.53%	motif file (matrix)	svg
103	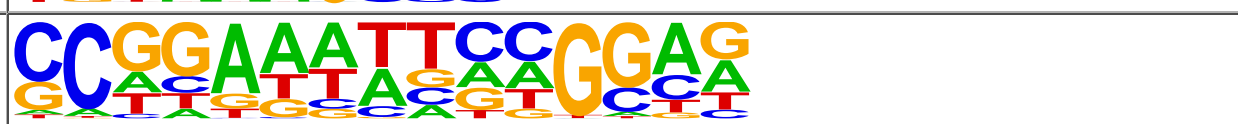	ASL18(LOBAS2)/colamp-ASL18-DAP-Seq(GSE60143)/Homer	1e-2	-5.902e+00	0.0267	688.0	39.20%	13180.2	35.97%	motif file (matrix)	svg
104	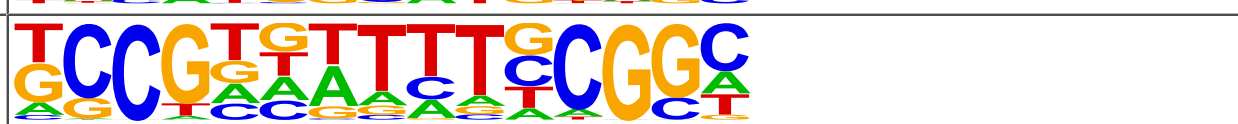	LBD13(LOBAS2)/colamp-LBD13-DAP-Seq(GSE60143)/Homer	1e-2	-5.821e+00	0.0287	648.0	36.92%	12372.3	33.77%	motif file (matrix)	svg
105	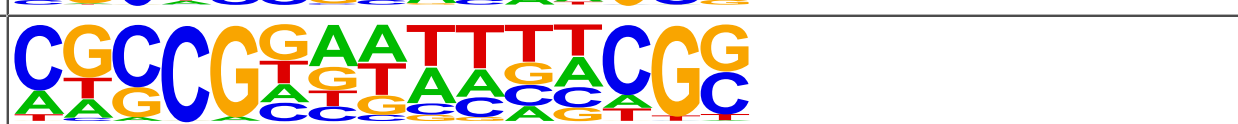	LOB(LOBAS2)/col-LOB-DAP-Seq(GSE60143)/Homer	1e-2	-5.758e+00	0.0303	292.0	16.64%	5234.4	14.29%	motif file (matrix)	svg
106	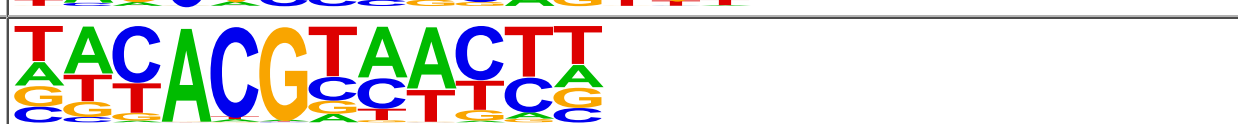	ANAC047(NAC)/colamp-ANAC047-DAP-Seq(GSE60143)/Homer	1e-2	-5.739e+00	0.0306	176.0	10.03%	2993.4	8.17%	motif file (matrix)	svg
107	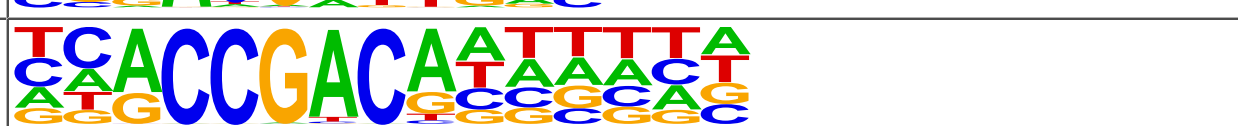	AT3G16280(AP2EREBP)/colamp-AT3G16280-DAP-Seq(GSE60143)/Homer	1e-2	-5.701e+00	0.0314	145.0	8.26%	2410.7	6.58%	motif file (matrix)	svg
108	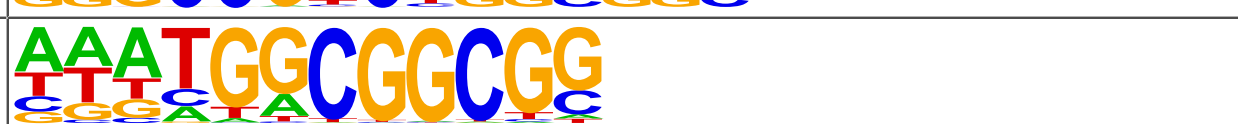	ERF9(AP2EREBP)/colamp-ERF9-DAP-Seq(GSE60143)/Homer	1e-2	-5.699e+00	0.0314	542.0	30.88%	10231.2	27.93%	motif file (matrix)	svg
109	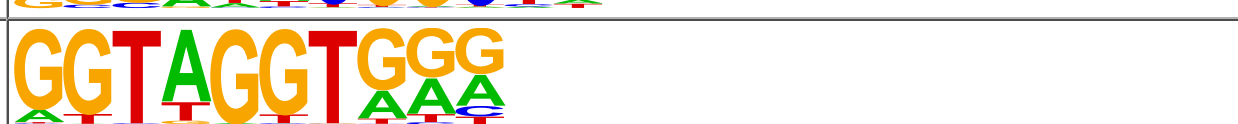	MYB17(MYB)/colamp-MYB17-DAP-Seq(GSE60143)/Homer	1e-2	-5.670e+00	0.0318	195.0	11.11%	3361.3	9.17%	motif file (matrix)	svg
110	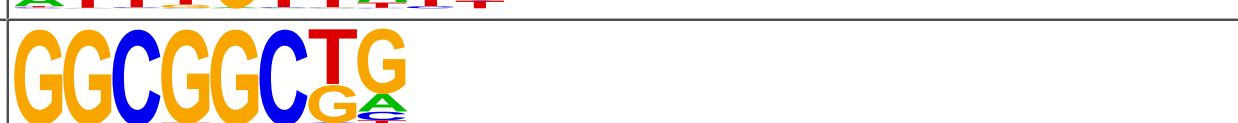	ERF2(AP2EREBP)/colamp-ERF2-DAP-Seq(GSE60143)/Homer	1e-2	-5.599e+00	0.0339	844.0	48.09%	16442.9	44.88%	motif file (matrix)	svg
111	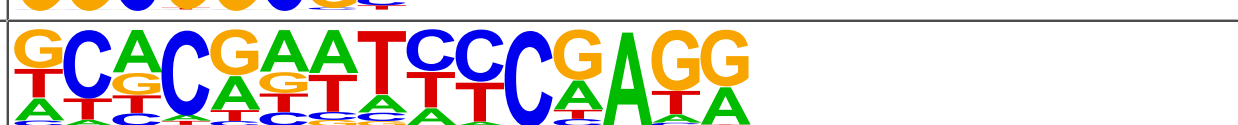	AIL7(AP2EREBP)/colamp-AIL7-DAP-Seq(GSE60143)/Homer	1e-2	-5.516e+00	0.0364	170.0	9.69%	2896.9	7.91%	motif file (matrix)	svg
112	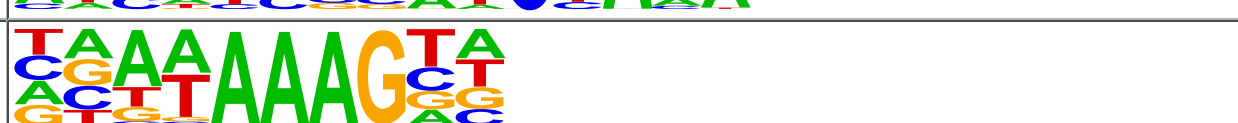	dof45(C2C2dof)/col-dof45-DAP-Seq(GSE60143)/Homer	1e-2	-5.436e+00	0.0391	728.0	41.48%	14067.6	38.40%	motif file (matrix)	svg
113	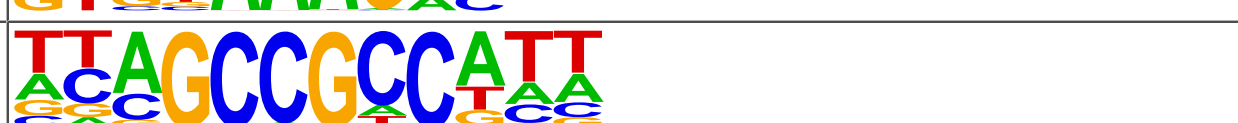	ERF13(AP2EREBP)/colamp-ERF13-DAP-Seq(GSE60143)/Homer	1e-2	-5.330e+00	0.0431	847.0	48.26%	16544.7	45.16%	motif file (matrix)	svg
114	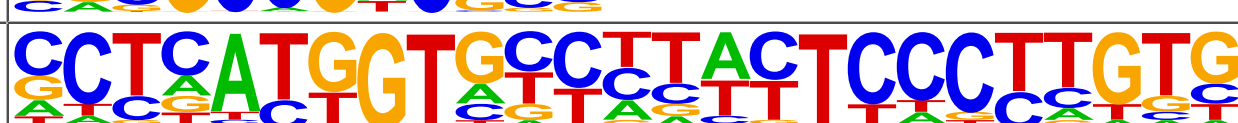	ZNF41(Zf)/HEK293-ZNF41.GFP-ChIP-Seq(GSE58341)/Homer	1e-2	-5.196e+00	0.0489	27.0	1.54%	327.5	0.89%	motif file (matrix)	svg
115	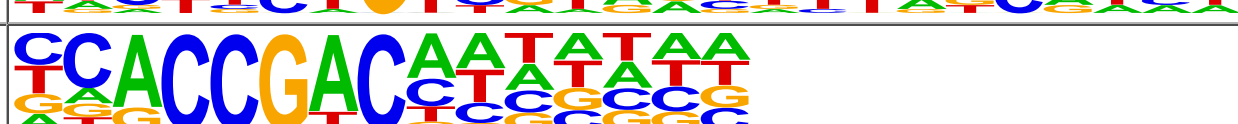	DEAR3(AP2EREBP)/colamp-DEAR3-DAP-Seq(GSE60143)/Homer	1e-2	-5.171e+00	0.0497	137.0	7.81%	2297.8	6.27%	motif file (matrix)	svg
116	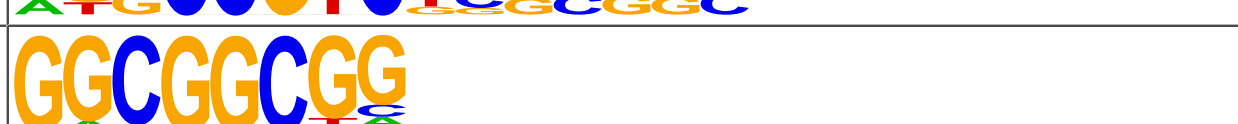	ESE1(AP2EREBP)/col-ESE1-DAP-Seq(GSE60143)/Homer	1e-2	-5.068e+00	0.0546	865.0	49.29%	16958.4	46.29%	motif file (matrix)	svg
117	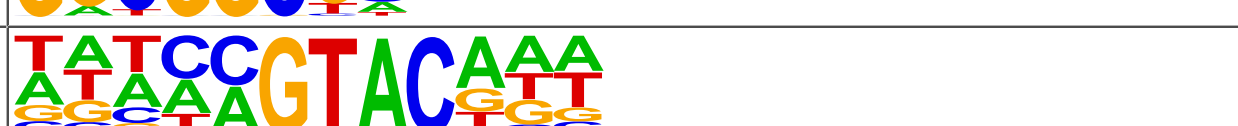	SPL15(SBP)/colamp-SPL15-DAP-Seq(GSE60143)/Homer	1e-2	-5.067e+00	0.0546	432.0	24.62%	8093.5	22.09%	motif file (matrix)	svg
118	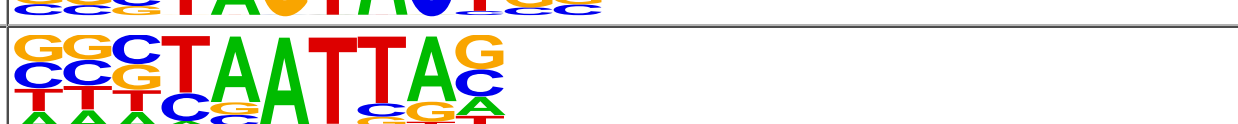	DLX2(Homeobox)/BasalGanglia-Dlx2-ChIP-seq(GSE124936)/Homer	1e-2	-5.033e+00	0.0556	439.0	25.01%	8238.6	22.49%	motif file (matrix)	svg
119	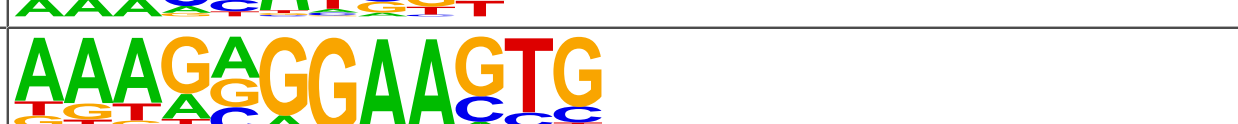	SpiB(ETS)/OCILY3-SPIB-ChIP-Seq(GSE56857)/Homer	1e-2	-4.954e+00	0.0596	89.0	5.07%	1418.1	3.87%	motif file (matrix)	svg
120	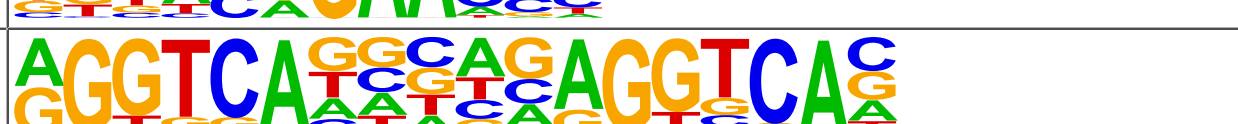	RAR:RXR(NR),DR5/ES-RAR-ChIP-Seq(GSE56893)/Homer	1e-2	-4.940e+00	0.0600	10.0	0.57%	82.4	0.22%	motif file (matrix)	svg
121	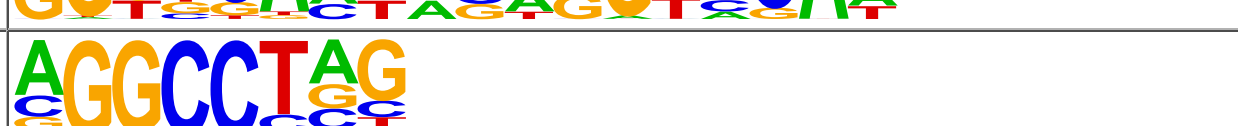	ZNF711(Zf)/SHSY5Y-ZNF711-ChIP-Seq(GSE20673)/Homer	1e-2	-4.832e+00	0.0663	908.0	51.74%	17891.2	48.83%	motif file (matrix)	svg
122	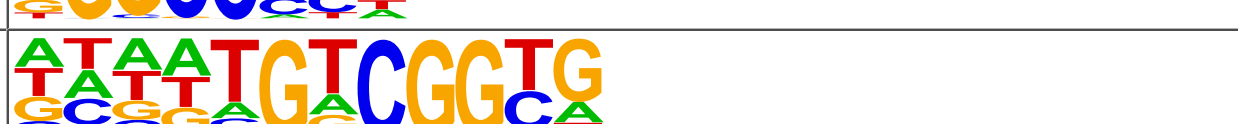	AT1G71450(AP2EREBP)/col-AT1G71450-DAP-Seq(GSE60143)/Homer	1e-2	-4.817e+00	0.0667	847.0	48.26%	16623.4	45.37%	motif file (matrix)	svg
123	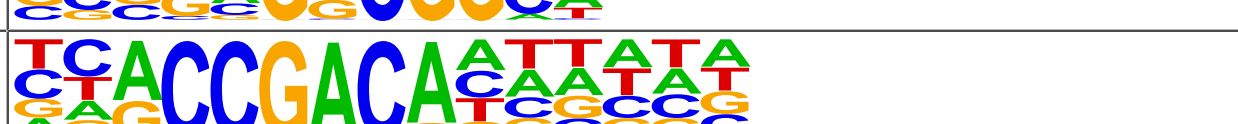	At2g44940(AP2EREBP)/colamp-At2g44940-DAP-Seq(GSE60143)/Homer	1e-2	-4.787e+00	0.0682	64.0	3.65%	974.8	2.66%	motif file (matrix)	svg
124	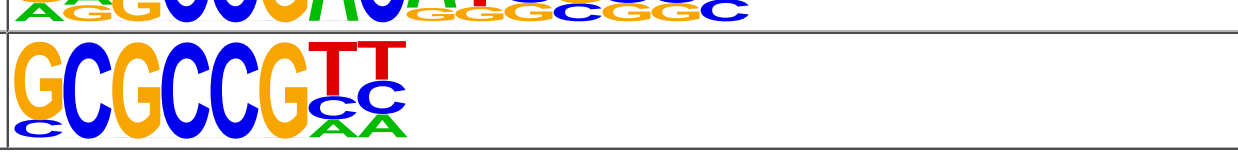	PUCHI(AP2EREBP)/colamp-PUCHI-DAP-Seq(GSE60143)/Homer	1e-2	-4.688e+00	0.0747	855.0	48.72%	16810.3	45.88%	motif file (matrix)	svg
