# Supplementary material for: Kdm2a histone demethylase ablation restores anti-tumor immunogenicity in liver cancer
Source: Br J Cancer. 2026 Aug 19;135(10):1587–602. doi: 10.1038/s41416-026-03566-z (PMC13740323; doi:10.1038/s41416-026-03566-z)
Supplement: Supplementary file 2 [file 41416_2026_3566_MOESM1_ESM.docx]

**Supplementary materials**

**Figures**

**Supplementary Figure 1. KDM2A mRNA extracted from FFPE tissue specimens of human HCC**

**Supplementary Figure 1 legend. A,** representative images of histological staining of human HCC grade 2 with H&E and antibodies against KDM2A, CD163, CD80, CD4, CD8. Scale bar, 200 µm. B, fold change of KDM2A mRNA extracted from FFPE tissue specimens of human HCC with different histological grading. PT= peritumoral tissue. Data shown as mean±SD, *p<0.01

**Supplementary Figure 2. KDM2A inhibition disrupts mitochondrial function and activates glycolysis.**

**Supplementary Figure 2 legend. A**, differential expression gene heatmap. All the differentially expressed genes in the comparison ex vivo KDM2A depleted Hepa1-6 and control Hepa1-6 were pooled as the differential gene set. We used the mainstream hierarchical clustering to cluster the fpkm values of genes, and homogenized the row (Z-score). The genes with similar expression patterns in the heatmap were gathered. Red color indicates genes with high expression levels, and black color indicates genes with low expression levels. **B,C** GSEA analysis for glycolysis and reactive oxygen species biosynthesis in ex vivo KDM2A depleted vs control Hepa1-6 cells. FDR= false discovery rate, NES=normalized enrichment score. **D**, enzymes of glycolysis pathway are displayed as heatmap. Color represents increased genes, indicated as log2(FC) (p-adj<0.05) in ex vivo KDM2A depleted Hepa1-6 vs scramble cells. **E**, KEGG enrichment results in ex vivo KDM2A depleted Hepa1-6, 20 upregulated KEGG pathways were selected for display. The abscissa is the ratio of the number of differential genes linked with the KEGG pathway to the total number of differential genes. The ordinate is KEGG Pathway. The size of a point represents the number of genes annotated to a specific KEGG pathway. The color from red (most significant) to purple (no significant) represents the significant level of the enrichment. **F**, a comprehensive KEGG pathview analysis with data integration and visualization relative to oxidative phosphorylation with downregulated genes (green boxes) belonging to NADH dehydrogenase (complex I of electron transport chain)

**Supplementary Figure 3. KDM2A depletion results in cell growth inhibition and downregulation of inflammatory genes in Huh7 cells.**

**Supplementary Figure 3 legend. A,** Time course of Huh7 cells growth. KDM2A depleted Huh7 cells and scrambled Huh7 cells were plated in culture dishes and grown for up 6 days. Results are expressed as mean±SEM.* p<0.01, **p<0.001 as determined by unpaired Student's t-test. **B**, Growth rate of Huh7 cells transfected in transient with scramble siRNA (CTL) and siRNA directed against KDM2A (KDM2A_siRNA) at different concentrations. After 3 days post-transfection, cells were stained with crystal violet. **C**, Volcano plot was used to infer the overall distribution of differentially expressed genes. The x-axis shows the log_2_ fold change in gene expression between KDM2A depleted Huh7 cells and control Huh7 cells, and the y-axis shows the statistical significance of the differences. Red dots represent up-regulation genes and green dots represent down-regulation genes. The dashed lines indicate the threshold line for differential gene screening criteria. **D**, GSEA analysis in KDM2A depleted versus control Huh7 cells. FDR=false discovery rate, NES= normalized enrichment score. **E**, Gene Ontology (GO) enrichment analysis of downregulated genes in KDM2A depleted Huh7 cells versus control, the most significant 30 GO terms were selected for display. In the figure, the abscissa is the ratio of the number of differential genes linked with the GO terms to the total number of differential genes, and the ordinate is GO terms. The size of a point represents the number of genes annotated to a specific GO terms, and the color from red to purple represents the significant level of the enrichment. The GO relative to inflammatory pathways are identified below a red line

**Supplementary Figure 4. Genome wide distribution of H3K36me2 DNA binding peaks**

**Supplementary Figure 4 legend. A, B** distribution of peak summits relative to transcription start site (TSS) predicting the binding sites of proteins on genes. ChIPSeeker software has been used to obtain the relevant genes for each peak, and calculate the number of peaks based on their distance from TSS. **C**, **D** summary of genome wide distribution of peaks. The number of the peaks mapping to the chromosome reflects the distribution of the protein binding sites. Horizontal axis is the coordinate of the peaks in the chromosome. Vertical is the chromosomes. Every blue bar represents a peak**. E**,**F** Heatmap of signal distribution around TSS (**E**) and gene body (**F**). The computeMatrix module of DeepTools software is used to statistically analyze the signals in the 3000bp upstream and downstream regions of the transcription start site (TSS) and gene body.

**Supplementary Figure 5. Redistribution of H3K36me2 upon KDM2A depletion modulates cellular functions.**

**Supplementary Figure 5 legend. A**, overall distribution of the different peak expressed as fold change and FDR in the form of a volcano plot. The upward peak is represented by a red dot, the downward peak is represented by a blue dot, and the non differential peak identified by a dashed line is represented by a green dot. The horizontal axis is the fold changes in signal intensity of the peak, the vertical axis represents the statistical significance of the degree of difference between the two samples. **B**, Venn diagram between the peaks of Hepa1-6shKDM2A (KD_H3K36me2_IP) and Hepa1-6shCTL (CTL_H3K36me2_IP). If the overlap between two peak regions has at least 1 bp, it is considered that the two peaks intersect. **C**, GO enrichment analysis of differentially expressed peak-associated genes among the downregulated genes in KDM2A-depleted Hepa1-6 cells (KD) vs control (CTL). **D**, KEGG enrichment analysis of differentially expressed peak-associated genes among the downregulated genes in KDM2A depleted Hepa1-6 cells (KD) vs control (CTL). **E, F** plot of KEGG enrichment results, selected the 20 most significant KEGG pathways and draw a bar chart for display. The horizontal axis in the figure represents the KEGG pathway, and the vertical axis represents the significance level of pathway enrichment. The higher the value, the more significant it is. The value on the column represents the number of expressed genes enriched in this pathway.

**Immunoblotting raw data**

**FIGURE 1**

**FIGURE 4**

**FIGURE 5**

**FIGURE 6**

**FIGURE 7**
